# Synthesis and Evaluation of (Bis)benzyltetrahydroisoquinoline Alkaloids as Antiparasitic Agents

**DOI:** 10.1021/jacsau.4c00007

**Published:** 2024-02-12

**Authors:** Ana Sozanschi, Hannah Asiki, Maiara Amaral, Erica V. de Castro Levatti, Andre G. Tempone, Richard J. Wheeler, Edward A. Anderson

**Affiliations:** † Chemistry Research Laboratory, Department of Chemistry, University of Oxford, 12 Mansfield Road, Oxford OX1 3TA, U.K.; ‡ Peter Medawar Building for Pathogen Research, Nuffield Department of Medicine, University of Oxford, Oxford , OX1 3SY, U.K.; § Laboratory of Pathophysiology, Butantan Institute, Av. Vital Brazil, 1500, 05503-900 São Paulo, Brazil; ∥ Instituto de Medicina Tropical, Faculdade de Medicina, Universidade de São Paulo, 05403-000 São Paulo, Brazil

**Keywords:** benzyltetrahydroisoquinoline, alkaloids, leishmaniasis, chagas disease, parasites, neglected tropical disease, natural products

## Abstract

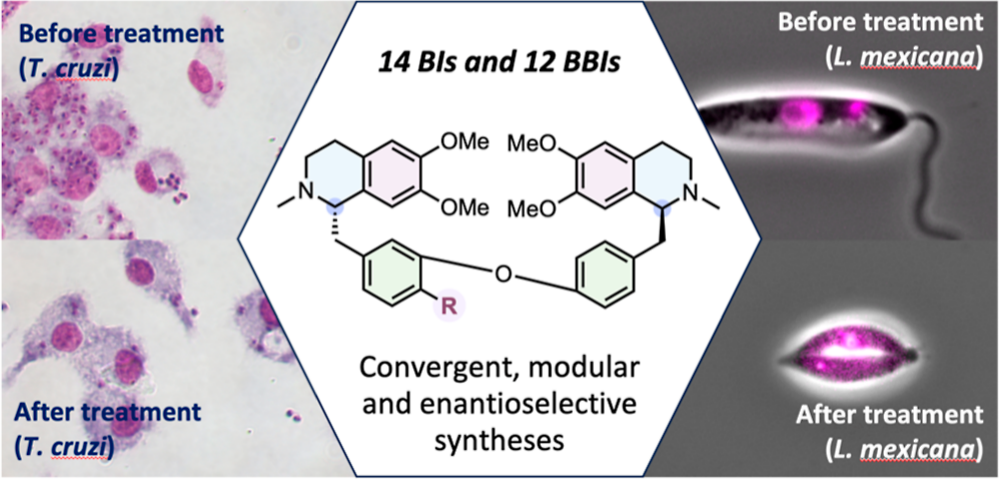

Visceral leishmaniasis and Chagas disease are neglected tropical diseases (NTDs) that severely impact the developing world. With current therapies suffering from poor efficacy and safety profiles as well as emerging resistance, new drug leads are direly needed. In this work, 26 alkaloids (9 natural and 17 synthetic) belonging to the benzyltetrahydroisoquinoline (BI) family were evaluated against both the pro/trypomastigote and amastigote forms of the parasites *Leishmania infantum* and *Trypanosoma cruzi*, the causative agents of these diseases. These alkaloids were synthesized via an efficient and modular enantioselective approach based on Bischler-Napieralski cyclization/Noyori asymmetric transfer hydrogenation to build the tetrahydroisoquinoline core. The bis-benzyltetrahydroisoquinoline (BBI) alkaloids were prepared using an Ullmann coupling of two BI units to form the biaryl ether linkage, which enabled a comprehensive survey of the influence of BI stereochemistry on bioactivity. Preliminary studies into the mechanism of action against *Leishmania mexicana* demonstrate that these compounds interfere with the cell cycle, potentially through inhibition of kinetoplast division, which may offer opportunities to identify a new target/mechanism of action. Three of the synthesized alkaloids showed promising druglike potential, meeting the Drugs for Neglected Disease *initiative* (DND*i*) criteria for a hit against Chagas disease.

## Introduction

Leishmaniases are a group of protozoan neglected diseases caused by over 20 species of the *Leishmania* genus that are transmitted via infected sandflies and affect roughly 12 million people worldwide.^1,2^ The wide range of clinical manifestations, asymptomatic cases, and poor diagnosis suggests that the incidence numbers are likely significantly higher than estimated.^3^ Visceral leishmaniasis (VL), the most severe form of the disease, is caused by *Leishmania donovani* and *Leishmania infantum* and is lethal unless treated, with up to 65,000 deaths annually,^4^ thus being the deadliest parasitic disease after malaria.^5^ The fatality of the disease is attributed to the parasite spreading to and irreversibly damaging vital organs such as the liver, spleen, and bone marrow.^6^ First line treatments are limited to pentavalent antimonials, liposomal amphotericin B, and miltefosine.^7−9^ However, all drugs currently in use suffer from drawbacks such as long and expensive treatment courses and associated toxicity.^10,11^ The related protozoan *Trypanosoma cruzi* causes Chagas disease, which affects over 8 million people, predominantly in South America.^12^ Similarly to leishmaniasis, this disease can be difficult to diagnose due to asymptomatic patients that become chronically infected in the absence of treatment.^12^ Chagas disease ultimately leads to cardiac failure, although gastrointestinal involvement (megacolon, mega-esophagus) can also be part of the clinical manifestation.^13,14^ In terms of treatment, Chagas disease suffers from similar limitations as VL: only two drugs are available (nifurtimox and benznidazole), with an even more limited pipeline of potential new therapeutics.^15,16^

Controlling and eradicating these diseases is further impeded by the emergence of parasite drug resistance; this typically appears as a consequence of poor patience compliance and the use of monotherapies as opposed to a combination of antiparasitic drugs.^4,15,17^ As such, there is an urgent need for novel drug leads that are efficient, safe, and readily accessible and elicit their therapeutic effect via novel targets.

**Figure 1:**
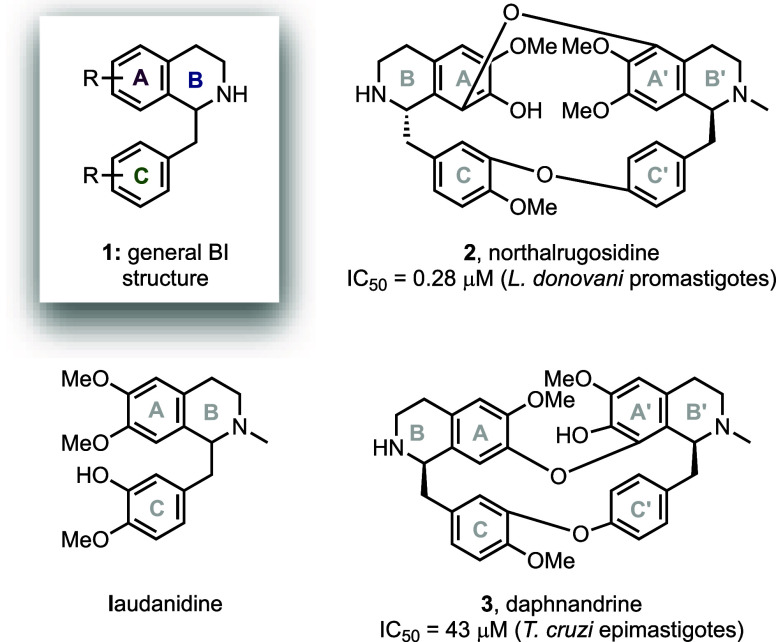
General structure of a BI alkaloid (1), example of BI laudanidine (this work), BBIs 2 and 3, and their reported antiparasitic activity.

Benzyltetrahydroisoquinoline (BI) and bis-benzyltetrahydroisoquinoline (BBI) natural products (e.g., Figure 1) belong to a large subclass of isoquinoline alkaloids found in plants in the tropical and subtropical regions. This family features one (BI) or two (BBI) 1,2,3,4-tetrahydroisoquinoline moieties to which a substituted benzyl group is attached; in the case of BBIs, there is at least one biaryl ether linkage joining the two BI units together.^18^ Many alkaloids in this family have been investigated due to their interesting pharmacological profiles, including antimicrobial and anticancer properties.^19,20^ Macrocyclic BBIs have been sporadically investigated as antiparasitic agents over the last half century, with alkaloids such as northalrugosidine (**2**) and daphnandrine (**3**) shown to be active against extracellular *L. donovani* promastigotes and *T. cruzi* epimastigotes, respectively.^21−25^

However, since much of this work has been performed on the readily cultured extracellular insect life cycle stages of the parasites, relying on simpler assays that in the case of neglected tropical diseases rarely yield viable drug candidates, BBIs remain to be rigorously investigated as potential antiparasitic agents.^26^ To the best of our knowledge, the simpler BIs themselves have not been investigated for antileishmanial or antitrypanosomal properties. Building on our previous studies of the structurally related aporphine alkaloids,^27^ we now report a modular and enantioselective synthesis of 14 BIs and 12 nonmacrocyclic BBIs, alongside a comprehensive examination of their biological activity (and the influence of BI/BBI stereochemistry) on both the extra- and intracellular forms of *L. infantum* and *T. cruzi*. The resulting structure–activity relationships (SAR) reveal the importance of stereochemistry in relation to bioactivity. Finally, preliminary studies into the mechanism of action of BI alkaloids on *Leishmania mexicana* are described.

## Results and Discussion

**Scheme 1:**
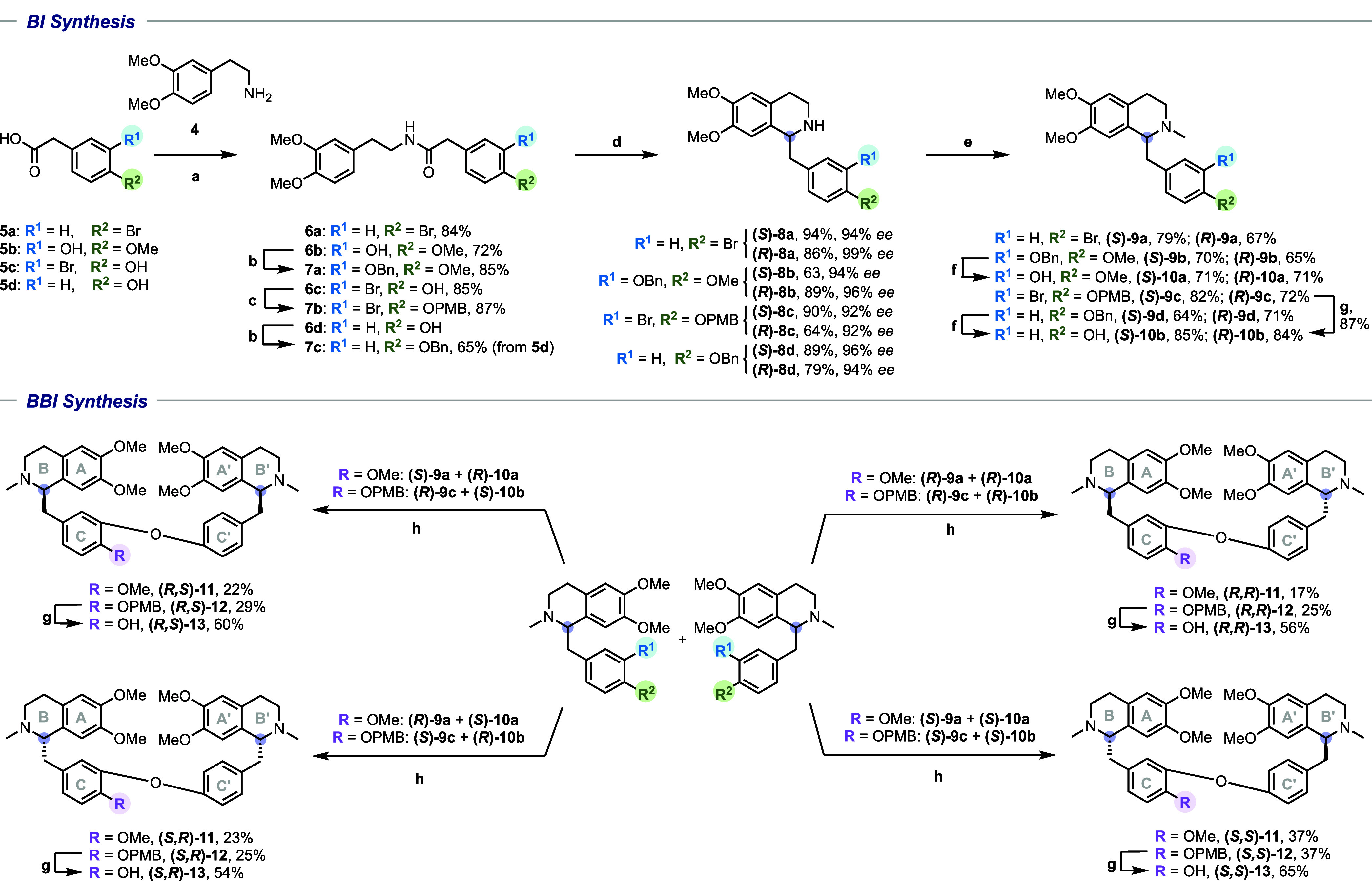
Synthesis of BI and BBI Alkaloids Reagents and conditions: (a) EDC·HCl, HOBt, Et_3_N, CH_2_Cl_2_, rt; (b) BnBr, TBAI, K_2_CO_3_, acetone, rt; (c) PMBCl, K_2_CO_3_, DMF, 80 °C; (d) 1. Tf_2_O, 2-Clpyr., CH_2_Cl_2_, −78 °C to rt; 2. RuCl[(*R,R*) or (*S,S*)TsDPEN](*p*-cymene), HCO_2_H/Et_3_N (5:2), DMF, 0 °C to rt; (e) H_2_CO, NaBH_4_, MeOH, 0 °C to rt; (f) H_2_, Pd/C (10 wt %), AcOH, MeOH, rt. (g) H_2_, Pd/C (10 wt %), Et_3_N, MeOH, rt; (h) CuO, K_2_CO_3_, pyridine, 140 °C.

The strategy to access our BI/BBI library is shown in Scheme 1. These particular compounds were selected due to the commercial availability of many building blocks, streamlining the synthesis, and also due to their *pseudo*symmetry, which enabled a modular and convergent approach. That acyclic BBIs have not yet been evaluated for antileishmanial and antitrypanosomal activity, as well as the question of the influence of their stereochemistry, offered an additional basis for this selection.

The synthesis commenced (Scheme 1) with the coupling of homoveratrylamine **4** to phenylacetic acid derivatives **5a**–**d** using EDC·HCl/HOBt, which gave amides **6a**–**c** in good yields (72–85%). Since free phenols are incompatible with the cyclodehydration conditions for dihydroisoquinoline (DHIQ) synthesis developed by Movassaghi and Hill,^28^ phenols **6b** and **6d** were protected as benzyl ethers **7a** and **7c** (61–65% over two steps from **6b,d**), while phenol **6c** was protected as the PMB ether **7b** (87%), which introduces an element of structural diversity on the C ring. Subsequent Bischler–Napieralski cyclization^28^ (BN)/Noyori asymmetric transfer hydrogenation^29^ (ATH) afforded the enantioenriched BIs **8a**–**d** in good to excellent yields and high enantioselectivity (63–94% over two steps, >92% *ee*). The 3,4-DHIQs resulting from the BN cyclization step are known to be air-sensitive, and as such were immediately subjected to the reduction step.^30^ Amines **8a**–**d** were then N-methylated, with subsequent Pd-catalyzed debenzylation of the two enantiomers of **9b** and **9d** affording the four natural BI targets (+)-laudanidine [(*S*)**-10a**, 71%], (−)-laudanidine [(*R*)**-10a**, 71%], (+)-armepavine [(*S*)**-10b**, 85%], and (−)-armepavine [(*R*)**-10b**, 84%].

With a selection of BIs in hand, bromo-BIs (*S*)**-9a** and (*R*)**-9a** were each subjected to copper-catalyzed Ullman coupling with phenols (*S*)**-10a** and (*R*)**-10a** (Scheme 1b) to afford four diastereomers of **11** in modest yields (17–37%). The same approach was employed in the synthesis of the four diastereomers of **12** from bromo-BIs (*S*)**-9c**/(*R*)**-9c** and phenols (*S*)**-10b**/(*R*)**-10b** (25–37%). The moderate yields of these Ullman couplings likely reflect the relatively electron-rich nature of the bromide coupling partners and the steric hindrance afforded by their *ortho-*substituents. Finally, PMB deprotection of the diastereomers of **12** afforded (−)-dauricine [(*R,R*)**-13**, 56%], (+)-dauricine [(*S,S*)**-3**, 65%], and unnatural stereoisomers (*R,S*)**-13** and (*S,R*)**-13** (60% and 54%). For BBIs **12**, we also implemented a divergent route whereby phenolic BIs (*S*)- and (*R*)**-10b** were prepared from the common precursor BIs (*S*)**-9c** and (*R*)**-9c** via tandem debromination/PMB deprotection (Scheme 1, conditions g), from which the desired phenols (*S*)**-10b** and (*R*)**-10b** were obtained in good yields (87% for both). Initial conditions tested for this transformation resulted in the formation of (*S*)- and (*R*)**-10c**, which were also taken forward for evaluation against the parasites (see the Supporting Information for synthetic details). While both routes afford (*R,R*)**-13** in similar overall yields (12% from **6d** vs 11% from **6c**), the use of the common precursors **10** improves the synthesis efficiency by removing the need for a discrete starting material, thus reducing the overall step count by 5. In summary, four natural and ten non-natural BI alkaloids and five natural and seven non-natural BBI alkaloids were synthesized.

The *Leishmania* parasite adopts two major morphological classes during its life-cycle stages: promastigotes in the insect vector and amastigotes in mammalian hosts.^31^ The DND*i* suggests that phenotypic screening is the best method to discover antileishmanial drug hits, since there is a lack of validated targets, and known targets have failed to deliver drug candidates.^26^ These phenotypic assays are best performed on intracellular amastigotes, which are the clinically relevant form of the parasite and mimic the in vivo conditions to which a drug would be exposed. Nonetheless, assays based on the promastigote form can also provide information on the direct action of the compounds on the cells.

Table 1 depicts the antileishmanial (*L. infantum*) activity evaluation performed against both the amastigote form (evaluated microscopically using an ex vivo intracellular model of mice macrophages) and the promastigote one (using an MTT colorimetric method).^32,33^ The cytotoxicity was evaluated against NCTC cells using an MTT colorimetric method and the selectivity index was determined using the ratio between the CC_50_ values (NCTC cells) and the IC_50_ values (amastigotes).^32^ Ten out of the 14 BIs tested showed potent activity (IC_50_ < 10 μM) against *L. infantum* promastigotes. In general, the (*R*)-enantiomers displayed superior activity, and were less toxic to mammalian cells, than the enantiomeric (*S*)-series. With respect to the substituents on the C ring, larger, hydrophobic groups conferred greater bioactivity (e.g., **9b**–**d**), whereas small, hydrophilic groups such as a hydroxyl group afford modest to poor bioactivity. The most active BI of this series was (*R*)**-9d** (IC_50_ = 1.1 ± 0.5 μM). However, all BIs showed significant mammalian cytotoxicity apart from the phenols [(*S*)- and (*R*)**-10a, (***S*)- and (*R*)**-10b]**.

**Table 1:** Bioactivity of BIs and BBIs against *L. infantum* and *T. cruzi* and Mammalian Cytotoxicity^a^

	*L. infantum* IC_50_			*T. cruzi* IC_50_			
compound	promastigotes (μM ± SD)	amastigotes (μM ± SD)	SI	trypomastigotes (μM ± SD)	amastigotes (μM ± SD)	SI	CC_50_ (μM ± SD)
(*S*)**-9a**	18.4 ± 2.1	13.3 ± 0.9	3.2	14.4 ± 5.5	25.1 ± 3.1	1.7	42.5 ± 1.5
(*R*)**-9a**	15.5 ± 5.8	23.4 ± 3.4	3.7	28.3 ± 11.7	31.8 ± 4.3	2.7	86.8 ± 2.9
(*S*)**-10b**, (+)-armepavine	36.2 ± 5.2	NA	ND	123.6 ± 2.3	NA	ND	>200
*(R*)**-10b**, (–)-armepavine	4.8 ± 0.8	NA	ND	106.8 ± 1.8	NA	ND	>200
(*S*)**-9d**	3.7 ± 0.2	7.5 ± 2.2	<1	7.6 ± 0.4	3.2 ± 0.5	2.0	6.6 ± 1.5
(*R*)**-9d**	1.1 ± 0.5	9.4 ± 0.5	3.0	16.5 ± 6.1	6.2 ± 5.9	4.5	28.1 ± 0.5
(*S*)**-10c**	4.8 ± 0.2	NA	ND	20.4 ± 11.4	NA	ND	5.8 ± 0.5
(*R*)**-10c**	2.4 ± 0.9	9.3 ± 0.4	3.0	25.3 ± 2.1	NA	ND	28.1 ± 5.5
(*S*)**-9c**	5.2 ± 1.3	NA	ND	11.1 ± 1.7	3.3 ± 0.01	1.9	6.3 ± 2.3
(*R*)**-9c**	3.5 ± 1.2	NA	ND	8.5 ± 3.1	NA	ND	13.4 ± 1.3
(*S*)**-10a**, (+)-laudanidine	95.4 ± 2.0	NA	ND	>150	5.9 ± 0.9	>34	>200
(*R*)**-10a**, (–)-laudanidine	30.2 ± 4.8	NA	ND	>150	2.6 ± 0.3	>77	>200
(*S*)**-9b**	8.6 ± 0.8	26.7 ± 2.0	1.8	57.7 ± 2.3	12.2 ± 0.5	3.9	47.1 ± 11.2
(*R*)**-9b**	3.3 ± 1.6	13.1 ± 0.8	4.7	72.3 ± 3.0	4.3 ± 0.4	13	57.3 ± 3.3
(*R,S*)**-11**	0.6 ± 0.1	NA	ND	6.5 ± 2.1	NA	ND	7.3 ± 0.7
(*S,R*)**-11**,(−)-*O,O′-*dimethylgrisabine	0.8 ± 0.01	NA	ND	2.6 ± 2.9	2.2 ± 0.7	4.7	10.2 ± 1.4
(*R,R*)**-11**,(−)-*O*-methyldauricine	0.7 ± 0.1	NA	ND	7.2 ± 0.6	1.5 ± 0.2	7.8	11.4 ± 4.8
(*S,S*)**-11**,(+)-*O*-methylthalibrine	0.7 ± 0.01	NA	ND	3.7 ± 3.6	NA	ND	11.4 ± 0.2
(*R,S*)**-13**	3.2 ± 1.7	NA	ND	10.1 ± 3.9	2.8 ± 0.6	4.8	13.3 ± 1.6
(*S,R*)**-13**	5.7 ± 1.3	3.6 ± 0.3	4.4	4.2 ± 4.8	4.4 ± 0.4	3.6	15.6 ± 1.6
(*R,R*)**-13**, (–)-dauricine	3.2 ± 1.1	3.7 ± 0.6	1.9	3.7 ± 2.9	NA	ND	7.0 ± 1.0
(*S,S*)**-13**, (+)-dauricine	5.5 ± 0.6	NA	ND	5.1 ± 4.8	2.3 ± 1.1	7.0	15.9 ± 0.8
(*R,S*)**-12**	0.4 ± 0.2	NA	ND	1.3 ± 0.7	NA	ND	2.7 ± 1.6
(*S,R*)**-12**	0.7 ± 0.1	NA	ND	1.7 ± 0.3	NA	ND	4.7 ± 1.7
(*R,R*)**-12**	1.1 ± 0.5	NA	ND	7.3 ± 1.5	NA	ND	13.9 ± 1.6
(*S,S*)**-12**	1.1 ± 0.5	NA	ND	5.6 ± 2.1	NA	ND	20.8 ± 5.4
miltefosine	ND	6.5 ± 3.0	18	ND	ND	ND	119.7 ± 4.2
benznidazole	ND	ND	ND	12.8 ± 0.7	5.0 ± 1.5	>40	>200

a IC_50_: 50% inhibitory concentration; SD: standard deviation; CC_50_: 50% cytotoxic concentration; NA: not active (for *L. infantum* amastigotes IC_50_ > 150 μM; for *T. cruzi* amastigotes IC_50_ > 100 μM); ND: not determined. SI: selectivity index (CC_50_/IC_50_ amastigotes).

As mentioned above, the phenotypic intracellular amastigote assay is more representative of a compound’s potential to become an antiparasitic hit compound, and the activities obtained from this assay differ significantly from those obtained from the extracellular promastigote assay. Half of the tested BIs were inactive against *L. infantum* amastigotes, and only three compounds [(*S*)**-9d**, (*R*)**-9d** and (*R*)**-10c]** showed promising activity (IC_50_ < 10 μM). An anomalous result was obtained for (*R*)**-10c** as its enantiomer was found to be inactive, which is in contrast to all other findings for enantiomeric pairs. While differences were observed between the bioactivity of enantiomer pairs, no consistent trend was seen. Nonetheless, it would not be expected that the stereogenic centers in any of these BIs/BBIs would be susceptible to epimerization within the parasite, underlining the likely importance of stereochemistry on biological activity. Considering the criteria established by the DND*i* for a hit against VL (IC_50_ < 10 μM, and selectivity index (SI, the ratio between CC_50_ and IC_50_) > 10), none of the tested BIs present an optimal efficacy despite reasonable bioactivity.^26^ The three compounds that meet the potency threshold fall short, as they are too toxic to mammalian cells. For the BBI alkaloids, all compounds were found to be highly potent against *L. infantum* promastigotes, with the majority demonstrating submicromolar activity (e.g., BBIs **11**, (*R,S*)*-***12** and (*S,R*)**-12**). While the stereochemistry at C1 and C1′ affects the bioactivity, no obvious trend was found. Despite this potent activity in the extracellular assay, the PMB- and methoxy-substituted BBIs proved to be inactive in the intracellular amastigote assay. This may be due to the compounds being unable to cross multiple cell membranes or the toxicity exerted on the macrophages at the tested concentration. Only the phenolic BBIs (−)-dauricine ((*R,R*)**-13**) and one of its diastereomers (*S,R*)**-13** were active against the amastigote form; unfortunately, low CC_50_ values were again observed, rendering these compounds unsuitable as drug hits due to the low selectivity index (SI < 10).

*T. cruzi* also assumes several cell morphologies during its lifecycle, which differ between the insect vector and mammalian host. In the latter environment, the parasite transitions between extracellular bloodstream trypomastigotes and intracellular amastigotes; both forms are clinically relevant, particularly during the acute stage of Chagas disease.^34^ In this work, antitrypanosomal activity was tested against both the amastigote form (evaluated microscopically using an ex vivo intracellular model using mice macrophages) and the trypomastigote form (using the resazurin in vitro assay).^33,35^ Only two BIs ((*S*)**-9d** and (*R*)**-9c**) exhibited activity against extracellular trypomastigotes with IC_50_ values below 10 μM; however, both compounds also proved significantly cytotoxic. Phenolic compounds (**10a** and **10b**) demonstrated poor activity, and no trend was observed for one enantiomer series that was consistently superior to the other across the BI compound library. The results obtained from the intracellular amastigote assay were more encouraging, with three compounds meeting the DND*i* criteria for a hit against Chagas disease: (−)-laudanidine [(*R*)**-10a**], (+)-laudanidine [(*S*)**-10a**] and (*R*)**-9b** were found to be active at concentrations below 10 μM, display SIs >10, and can be synthesized in less than 8 steps. (−)-Laudanidine [(*R*)**-10a**] shows twice the selectivity of the current ‘gold standard’ drug benznidazole. Once again, the BBIs tested were found to display potent activity against extracellular *T. cruzi* trypomastigotes, with around half also showing potency in the amastigote assay. Unlike the BIs, lower CC_50_ values resulted in low SI values, likely rendering these dimeric species unfit as a hit scaffold. In silico predictions of pharmacokinetic profiles were carried out using the SwissADME webtool (see the Supporting Information for details).^36^ Based on the profile obtained for (*R*)**-10a**, BI compounds are predicted to have good oral bioavailability, whereas BBIs (*R,R*)**-11** and (*S,R*)**-13** are predicted to be inferior drug candidates due to decreased oral bioavailability.

**Figure 2:**
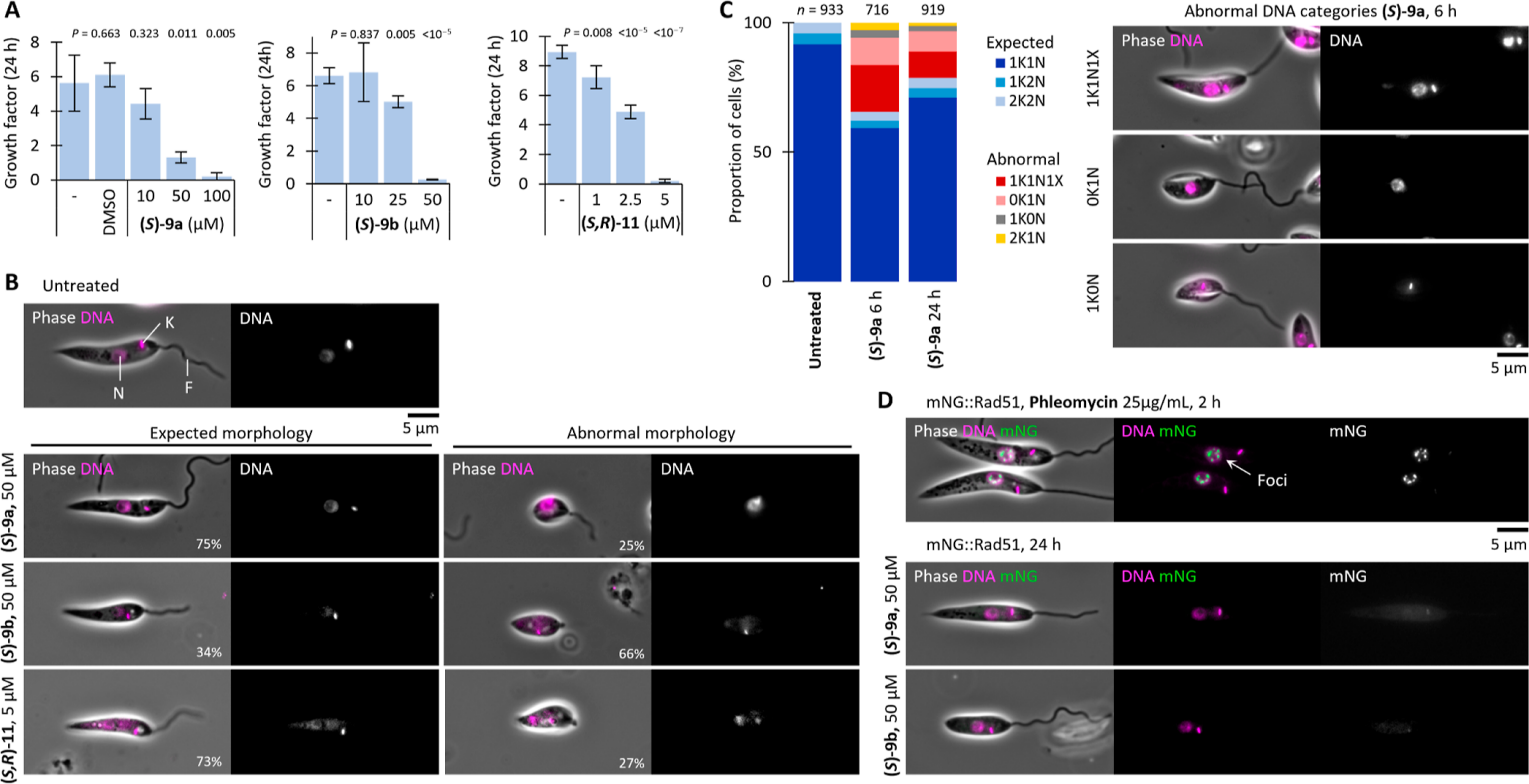
(A) 24-h growth factor, mean ± SD from *n* = 3 repeats. Statistical significance was derived from a two-tailed *t*-test versus untreated cells (−). (B) Characteristic light microscopy images showing the normal *L. mexicana* morphology (N = nucleus, K = kinetoplast, F = flagellum) and result of compound treatment from *n* > 80 cells. N and K stained here with Hoechst 33342 (magenta). (C) Counts of the cell cycle stage during growth in promastigote culture. *n* = number of cells counted, representative example from *n* = 2 repeats. Representative images of abnormal DNA categories 1K1N1X, 0K1N, and 1K0N were taken from cells treated with (***S***)-**9a**, 50 μM, 6 h. (D) Representative light microscopy images of mNeonGreen tagged RAD51 cell line (mNG::RAD51) under different treatment conditions. One replicate, representative images of *n* > 300 cells per condition.

Preliminary investigations into the mechanism of action for compounds (*S*)**-9a**, (*S*)**-9b** and (*S,R*)**-11** were carried out using *L. mexicana* promastigotes. Light microscopy-based techniques were employed to further explore compound activity in a different, related *Leishmania* species. Activity against *L. mexicana* was established by quantifying inhibition of growth over 24 h in a promastigote culture (Figure 2A). For compounds (*S*)**-9a** and (*S*)**-9b**, concentrations of 50 μM are sufficient to inhibit growth over 24 h, while BBI (*S,R*)**-11** was found to be 10-fold more potent, which reflects the findings in *L. infantum*. A solvent-only control using 0.1% (v/v) DMSO led to no significant effects. Cell cultures were examined by light microscopy to identify any morphological changes resulting from compound treatment (Figure 2B). The typical *Leishmania* promastigote cell morphology comprises an elongated cell body tapered at the posterior end, a flagellum (F) often of comparable length to the cell body, and two DNA containing organelles: the nucleus (N) and the kinetoplast (K).^37^ Cells treated with the BI and BBI compounds develop a changed morphology with a spherical cell body or shortened flagellum. This effect is observed in 25% of the population for compound (*S*)**-9a**, to a greater extent (66%) for (*S*)**-9b**, and 27% for compound (*S,R*)**-11**. An abnormal number and position of Ks and Ns were often observed, with Ks and Ns sometimes less well-defined by Hoechst 33342 staining.

To investigate whether the morphological changes are a consequence of cell cycle interference, we analyzed the cell cycle over a 24 h period. Untreated *L. mexicana* promastigotes typically have a doubling time of 7.1 h, with each of the DNA-containing organelles replicating once in the order nucleus (N) then kinetoplast (K), before cytokinesis, leading to two daughter cells.^37,38^ This organelle division occurs in the final 10% of the cell cycle, which leads to a heterogeneous population of cells with majority possessing the configuration 1K1N and only a small percentage of the population in 1K2N or 2K2N. Cells treated with (*S*)**-9a** at 50 μM were studied after 6 and 24 h of treatment, and found to deviate from the typical cell cycle, giving rise to abnormal categories of cells with incorrectly replicated organelles (Figure 2C). For around 15% of the cells in 1K1N there was the presence of an eXtra structure (1K1N1X) that could not be clearly assigned as K or N, generally toward the posterior end of the cell body. More prominently, the proportion of cells in the dividing stages (Non-1K1N) doubled from the expected 10%, with the results at the two time points being similar suggesting that the abnormalities do not accumulate over time.

In the related species *Trypanosoma brucei*, it has been shown that the replication and division cycles of the kinetoplast, nucleus, and cytokinesis occur independently of one other with minimal to no cross-talk between the subcycles, which can lead to cells failing in one stage but continuing replication of the others.^39,40^ We observed 10% of treated cells in the 0K1N configuration, while 3% are in 1K0N or 2K1N, which are considered abnormal categories not present in untreated cells. This suggests a failure of kinetoplast duplication but not of the nucleus, which upon cytokinesis would lead to 1K1N and 1N daughter cells. The small number of cells in a 2K1N configuration could arise from occasional failure of nucleus division, and daughter cells being 1K1N and 1K0N.^41,42^

Some BI alkaloids have been shown to intercalate DNA, and induce double stranded DNA breaks (DSBs) in cancer cell lines.^43^ DSBs often lead to cytotoxicity, which could be an explanation for the observed compound activity.^44−46^*Leishmania* employ the RAD51 DNA recombinase protein as part of its DNA repair machinery to fix naturally occurring nuclear DSBs via homologous recombination.^47−50^ Using a CRISPR/Cas9 gene editing strategy,^51^ we generated a genetically modified cell line with mNeonGreen tagged RAD51. This would allow observation of fluorescent RAD51 protein recruitment to foci in the nucleus corresponding to DSB accumulation, as has been shown in *T. brucei*.^52,53^ Phleomycin was used as a positive control to induce DSBs,^54^ which led to the expected observation of accumulation of RAD51 as green dots in the nucleus in 80% of cells from as early as 2 h posttreatment (Figure 2D). In contrast, compounds (*S*)**-9a** and (*S*)**-9b** demonstrated no RAD51 nuclear foci for up to 24 h. We can therefore conclude that the BI compounds tested are unlikely to act via DSBs (albeit this methodology is sensitive only to DSBs in nuclear DNA), and another mechanism of cell cycle disruption is responsible for the breakdown in the healthy cell cycle.

## Conclusions

While much progress has been made in the past few years and a promising pipeline of novel drugs exists now for VL, there remains a severe lack of drug leads in clinical trials against Chagas disease.^16^ Drug discovery and development for NTDs continues to be hindered by limited allocated resources, the lack of validated targets, limited knowledge of the mechanism of action of currently approved treatments, and limitations imposed by phenotypic screening. This work highlights a modular and efficient enantioselective approach that enables the synthesis of 14 BI and 12 linear BBI alkaloids. Key transformations included a Bischler-Napieralski cyclization followed by Noyori asymmetric transfer hydrogenation and, in the case of BBIs, a copper-catalyzed Ullmann cross coupling to form the biaryl ether bridge. We also demonstrated the use of Pd-mediated hydrogenation that allows the synthesis of the phenolic BI from the bromo-BI coupling partner, thus shortening the overall synthesis by five steps. These alkaloids were evaluated for antiparasitic activity against both the intracellular and extracellular forms of *L. infantum* and *T. cruzi*. The importance of stereochemistry was explored by testing both enantiomers of the BIs and all four stereoisomers of the BBI alkaloids, which revealed some correlation between the activity and enantiomeric series against *L. infantum* promastigotes. The BBI alkaloids were found to be highly potent antiparasitic compounds, but also highly cytotoxic, likely rendering them unsuitable as drug candidate hit compounds. However, the BIs (+)-laudanidine ((*S*)**-10a**), (−)-laudanidine ((*R*)**-10a**) and (*R*)**-9b** conformed to the DND*i* criteria for a hit compound against *T. cruzi*, having potent activity and good selectivity against the parasite in comparison to mammalian cells. Finally, preliminary studies of the mechanism of action for the BI alkaloids in *L. mexicana* revealed interference with the cell cycle, which appeared most likely to be due to inhibition of kinetoplast division. Nuclear double stranded DNA breaks are not induced as part of the compound mechanism, although kinetoplast DSBs could be responsible. Overall, this comprehensive assessment of BIs and acyclic BBIs offers a depth of information on the antiparasitic activity against various forms of the parasite. Further work may enable the discovery of a new target for the potential design of antiparasitic agents using nontoxic scaffolds, as well as focusing on the wider physicochemical and pharmacokinetic profiles of the more active BIs from this study.

## Supporting Information Available

The pharmacokinetic profile in silico prediction, experimental procedures, complete characterization data, biological assays and copies of ^1^H and ^13^C NMR spectra, as well as chiral SFC (supercritical fluid chromatography) chromatograms (PDF)

## Abbreviations

VL: visceral leishmaniasis
L.: *Leishmania*
T.: *Trypanosoma*
BI: benzyltetrahydroisoquinoline
BBI: bis-benzyltetrahydroisoquinoline
NTD: neglected tropical disease
DND*i*: Drugs for Neglected Diseases initiative
SAR: structure–activity relationship
NA: not active
ND: not determined
SI: selectivity index
SD: standard deviation
DNA: DNA
DSB: double-stranded DNA breaks
IC_50_: 50% inhibitory concentration
CC_50_: 50% cytotoxic concentration
ATH: asymmetric transfer hydrogenation
MTT: 3-(4,5-dimethylthiazol-2-yl)-2,5-diphenyltetrazolium bromide
EDC: 1-ethyl-3-(−3-(dimethylamino)propyl)carbodiimide
HOBt: hydroxybenzotriazole
PMB: *para*-methoxybenzene
RAD51: DNA repair protein
mNG: mNeonGreen

## References

[ref1] Burza S.; Croft S. L.; Boelaert M. Leishmaniasis. Lancet 2018, 392 (10151), 951–970. 10.1016/S0140-6736(18)31204-2. 30126638

[ref2] Balana-Fouce R.; Perez Pertejo M. Y.; Dominguez-Asenjo B.; Gutierrez-Corbo C.; Reguera R. M. Walking a tightrope: drug discovery in visceral leishmaniasis. Drug Discov. Today 2019, 24 (5), 1209–1216. 10.1016/j.drudis.2019.03.007. 30876846

[ref3] de Souza M. L.; Dos Santos W. M.; de Sousa A.; Ferraz L. R. d. M.; da Costa L. A. G.; Silva E. O.; Rolim Neto P. J. Cutaneous leishmaniasis: new oral therapeutic approaches under development. Int. J. Dermatol. 2022, 61 (1), 89–98. 10.1111/ijd.15902. 34510406

[ref4] Capela R.; Moreira R.; Lopes F. An Overview of Drug Resistance in Protozoal Diseases. Int. J. Mol. Sci. 2019, 20 (22), 5748 10.3390/ijms20225748. 31731801 PMC6888673

[ref5] Sangshetti J. N.; Kalam Khan F. A.; Kulkarni A. A.; Arote R.; Patil R. H. Antileishmanial drug discovery: comprehensive review of the last 10 years. RSC Adv. 2015, 5 (41), 32376–32415. 10.1039/C5RA02669E.

[ref6] Ready P. D. Epidemiology of visceral leishmaniasis. Clin. Epidemiol. 2014, 6, 147–154. 10.2147/CLEP.S44267. 24833919 PMC4014360

[ref7] Frezard F.; Demicheli C.; Ribeiro R. R. Pentavalent antimonials: new perspectives for old drugs. Molecules 2009, 14 (7), 2317–2336. 10.3390/molecules14072317. 19633606 PMC6254722

[ref8] Jha T. K.; Sundar S.; Thakur C. P.; Bachmann P.; Karbwang J.; Fischer C.; Voss A.; Berman J. Miltefosine, an Oral Agent, for the Treatment of Indian Visceral Leishmaniasis. N. Engl. J. Med. 1999, 341 (24), 1795–1800. 10.1056/NEJM199912093412403. 10588964

[ref9] Kumari S.; Kumar V.; Tiwari R. K.; Ravidas V.; Pandey K.; Kumar A. Amphotericin B: A drug of choice for Visceral Leishmaniasis. Acta Trop. 2022, 235, 106661 10.1016/j.actatropica.2022.106661. 35998680

[ref10] Alvar J.; den Boer M.; Dagne D. A. Towards the elimination of visceral leishmaniasis as a public health problem in east Africa: reflections on an enhanced control strategy and a call for action. Lancet Global Health 2021, 9 (12), e1763–e1769. 10.1016/s2214-109x(21)00392-2. 34798033 PMC8609279

[ref11] Wijnant G.-J.; Dumetz F.; Dirkx L.; Bulté D.; Cuypers B.; Van Bocxlaer K.; Hendrickx S. Tackling Drug Resistance and Other Causes of Treatment Failure in Leishmaniasis. Front. Trop. Dis. 2022, 3, 837460 10.3389/fitd.2022.837460.

[ref12] Perez-Molina J. A.; Molina I. Chagas disease. Lancet 2018, 391 (10115), 82–94. 10.1016/S0140-6736(17)31612-4. 28673423

[ref13] Echavarria N. G.; Echeverria L. E.; Stewart M.; Gallego C.; Saldarriaga C. Chagas Disease: Chronic Chagas Cardiomyopathy. Curr. Probl. Cardiol. 2021, 46 (3), 100507 10.1016/j.cpcardiol.2019.100507. 31983471

[ref14] WHO Expert Committee on the Control of Chagas Disease 2000: Brasilia, Brazil: World Health Organization. Control of Chagas Disease: Second Report of the WHO Expert Committee, 2002.

[ref15] Sales Junior P. A.; Molina I.; Fonseca Murta S. M.; Sanchez-Montalva A.; Salvador F.; Correa-Oliveira R.; Carneiro C. M. Experimental and Clinical Treatment of Chagas Disease: A Review. Am. J. Trop. Med. Hyg. 2017, 97 (5), 1289–1303. 10.4269/ajtmh.16-0761. 29016289 PMC5817734

[ref16] De Rycker M.; Wyllie S.; Horn D.; Read K. D.; Gilbert I. H. Anti-trypanosomatid drug discovery: progress and challenges. Nat. Rev. Microbiol. 2023, 21 (1), 35–50. 10.1038/s41579-022-00777-y. 35995950 PMC9395782

[ref17] Haldar A. K.; Sen P.; Roy S. Use of antimony in the treatment of leishmaniasis: current status and future directions. Mol. Biol. Int. 2011, 2011, 571242 10.4061/2011/571242. 22091408 PMC3196053

[ref18] Weber C.; Opatz T. Bisbenzylisoquinoline Alkaloids. Alkaloids: Chem. Biol. 2019, 81, 1–114. 10.1016/bs.alkal.2018.07.001. 30685048

[ref19] Otshudi A. L.; Apers S.; Pieters L.; Claeys M.; Pannecouque C.; De Clercq E.; Van Zeebroeck A.; Lauwers S.; Frederich M.; Foriers A. Biologically active bisbenzylisoquinoline alkaloids from the root bark of *Epinetrum villosum*. J. Ethnopharmacol. 2005, 102 (1), 89–94. 10.1016/j.jep.2005.05.021. 15996841

[ref20] Lv J. J.; Xu M.; Wang D.; Zhu H. T.; Yang C. R.; Wang Y. F.; Li Y.; Zhang Y. J. Cytotoxic bisbenzylisoquinoline alkaloids from *Stephania epigaea*. J. Nat. Prod. 2013, 76 (5), 926–932. 10.1021/np400084t. 23621840

[ref21] Kumar A.; Chowdhury S. R.; Sarkar T.; Chakrabarti T.; Majumder H. K.; Jha T.; Mukhopadhyay S. A new bisbenzylisoquinoline alkaloid isolated from *Thalictrum foliolosum*, as a potent inhibitor of DNA topoisomerase IB of *Leishmania donovani*. Fitoterapia 2016, 109, 25–30. 10.1016/j.fitote.2015.11.021. 26625837

[ref22] Camacho M. d. R.; Phillipson J. D.; Croft S. L.; Rock P.; Marshall S. J.; Schiff P. L. In vitro activity of *Triclisia patens* and some bisbenzylisoquinoline alkaloids against *L**eishmania donovani* and *Trypanosoma brucei brucei*. Phytother Res. 2002, 16 (5), 432–436. 10.1002/ptr.929. 12203262

[ref23] Fournet A.; Munoz V.; Manjon A. M.; Angelo A.; Hocquemiller R.; Cortes D.; Cave A.; Bruneton J. Activité antiparasitaire d’alcaloïdes bisbenzylisoquinoleiques I: activité in vitro sur des promastigotes de trois souches de leishmania. J. Ethnopharmacol. 1988, 24, 327–335. 10.1016/0378-8741(88)90162-6. 3253497

[ref24] Naman C. B.; Gupta G.; Varikuti S.; Chai H.; Doskotch R. W.; Satoskar A. R.; Kinghorn A. D. Northalrugosidine Is a Bisbenzyltetrahydroisoquinoline Alkaloid from *Thalictrum alpinum* with in Vivo Antileishmanial Activity. J. Nat. Prod. 2015, 78 (3), 552–556. 10.1021/np501028u. 25629555 PMC4394985

[ref25] Fournet A.; Manjon A. M.; Muñoz V.; Angelo A.; Bruneton J.; Hocquemiller R.; Cortes D.; Cavé A. Activité antiparasit aire d’alcaloïdes bisbenzylisoquinoléiques. II: Activité in vitro sur des epimastigotes de trois souches typifiees de *Trypanosoma cruzi*. J. Ethnopharmacol. 1988, 24, 337–343. 10.1016/0378-8741(88)90163-8. 3075676

[ref26] Don R.; Ioset J. R. Screening strategies to identify new chemical diversity for drug development to treat kinetoplastid infections. Parasitology 2014, 141 (1), 140–146. 10.1017/S003118201300142X. 23985066

[ref27] Pieper P.; McHugh E.; Amaral M.; Tempone A. G.; Anderson E. A. Enantioselective synthesis and anti-parasitic properties of aporphine natural products. Tetrahedron 2020, 76 (2), 130814–130822. 10.1016/j.tet.2019.130814.

[ref28] Movassaghi M.; Hill D. H. A Versatile Cyclodehydration Reaction for the Synthesis of Isoquinoline and Beta-Carboline Derivatives. Org. Lett. 2008, 10, 3485–3488. 10.1021/ol801264u. 18642832 PMC2692836

[ref29] Uematsu N.; Fujii A.; Hashiguchi S.; Ikariya T.; Noyori R. Asymmetric Transfer Hydrogenation of Imines. J. Am. Chem. Soc. 1996, 118 (20), 4916–4917. 10.1021/ja960364k.

[ref30] Blank N.; Opatz T. Enantioselective synthesis of tetrahydroprotoberberines and bisbenzylisoquinoline alkaloids from a deprotonated α-aminonitrile. J. Org. Chem. 2011, 76 (23), 9777–9784. 10.1021/jo201871c. 22004161

[ref31] Sunter J.; Gull K. Shape, form, function and Leishmania pathogenicity: from textbook descriptions to biological understanding. Open Biol. 2017, 7 (9), 170165 10.1098/rsob.170165. 28903998 PMC5627057

[ref32] Amaral M.; Asiki H.; Sear C. E.; Singh S.; Pieper P.; Haugland M. M.; Anderson E. A.; Tempone A. G. Biological activity and structure-activity relationship of dehydrodieugenol B analogues against visceral leishmaniasis. RSC Med. Chem. 2023, 14 (7), 1344–1350. 10.1039/D3MD00081H. 37484568 PMC10357944

[ref33] Martins L. F.; Mesquita J. T.; Pinto E. G.; Costa-Silva T. A.; Borborema S. E.; Galisteo Junior A. J.; Neves B. J.; Andrade C. H.; Shuhaib Z. A.; Bennett E. L.; et al. Analogues of Marine Guanidine Alkaloids Are in Vitro Effective against *Trypanosoma cruzi* and Selectively Eliminate *Leishmania (L.) infantum* Intracellular Amastigotes. J. Nat. Prod. 2016, 79 (9), 2202–2210. 10.1021/acs.jnatprod.6b00256. 27586460

[ref34] Tyler K. M.; Olson C. L.; Engman D. M. The Life Cycle Of *Trypanosoma Cruzi*. American Trypanosomiasis; World Class Parasites, 2003; pp 1–11.

[ref35] Ferreira D. D.; Sousa F. S.; Costa-Silva T. A.; Reimao J. Q.; Torrecilhas A. C.; Johns D. M.; Sear C. E.; Honorio K. M.; Lago J. H. G.; Anderson E. A.; et al. Dehydrodieugenol B derivatives as antiparasitic agents: Synthesis and biological activity against *Trypanosoma cruzi*. Eur. J. Med. Chem. 2019, 176, 162–174. 10.1016/j.ejmech.2019.05.001. 31103897

[ref36] Daina A.; Michielin O.; Zoete V. SwissADME: a free web tool to evaluate pharmacokinetics, drug-likeness and medicinal chemistry friendliness of small molecules. Sci. Rep. 2017, 7, 42717 10.1038/srep42717. 28256516 PMC5335600

[ref37] Wheeler R. J.; Gluenz E.; Gull K. The cell cycle of Leishmania: morphogenetic events and their implications for parasite biology. Mol. Microbiol. 2011, 79 (3), 647–662. 10.1111/j.1365-2958.2010.07479.x. 21255109 PMC3166656

[ref38] Ambit A.; Woods K. L.; Cull B.; Coombs G. H.; Mottram J. C. Morphological Events during the Cell Cycle of *Leishmania major*. Eukaryot. Cell 2011, 10 (11), 1429–1438. 10.1128/ec.05118-11. 21926331 PMC3209043

[ref39] Ploubidou A.; Robinson D. R.; Docherty R. C.; Ogbadoyi E. O.; Gull K. Evidence for novel cell cycle checkpoints in trypanosomes: kinetoplast segregation and cytokinesis in the absence of mitosis. J. Cell Sci. 1999, 112 (24), 4641–4650. 10.1242/jcs.112.24.4641. 10574712

[ref40] Wheeler R. J.; Gull K.; Sunter J. D. Coordination of the Cell Cycle in Trypanosomes. Annu. Rev. Microbiol. 2019, 73 (1), 133–154. 10.1146/annurev-micro-020518-115617. 31500537

[ref41] Grant K. M.; Dunion M. H.; Yardley V.; Skaltsounis A.-L.; Marko D.; Eisenbrand G.; Croft S. L.; Meijer L.; Mottram J. C. Inhibitors of *Leishmania mexicana* CRK3 Cyclin-Dependent Kinase: Chemical Library Screen and Antileishmanial Activity. Antimicrob. Agents Chemother. 2004, 48 (8), 3033–3042. 10.1128/aac.48.8.3033-3042.2004. 15273118 PMC478496

[ref42] Duncan S. M.; Myburgh E.; Philipon C.; Brown E.; Meissner M.; Brewer J.; Mottram J. C. Conditional gene deletion with DiCre demonstrates an essential role for CRK3 in *Leishmania mexicana* cell cycle regulation. Mol. Microbiol. 2016, 100 (6), 931–944. 10.1111/mmi.13375. 26991545 PMC4913733

[ref43] Inoue N.; Terabayashi T.; Takiguchi-Kawashima Y.; Fujinami D.; Matsuoka S.; Kawano M.; Tanaka K.; Tsumura H.; Ishizaki T.; Narahara H.; et al. The benzylisoquinoline alkaloids, berberine and coptisine, act against camptothecin-resistant topoisomerase I mutants. Sci. Rep. 2021, 11 (1), 7718 10.1038/s41598-021-87344-2. 33833336 PMC8032691

[ref44] Bennett C. B.; Westmoreland T. J.; Snipe J. R.; Resnick M. A. A Double-Strand Break within a Yeast Artificial Chromosome (YAC) Containing Human DNA Can Result in YAC Loss, Deletion, or Cell Lethality. Mol. Cell. Biol. 1996, 16 (8), 4414–4425. 10.1128/MCB.16.8.4414. 8754842 PMC231440

[ref45] Cannan W. J.; Pederson D. S. Mechanisms and Consequences of Double-Strand DNA Break Formation in Chromatin. J. Cell. Physiol. 2016, 231 (1), 3–14. 10.1002/jcp.25048. 26040249 PMC4994891

[ref46] da Silva M. S. DNA Double-Strand Breaks: A Double-Edged Sword for Trypanosomatids. Front. Cell Dev. Biol. 2021, 9, 669041 10.3389/fcell.2021.669041. 33937271 PMC8085331

[ref47] McKean P. G.; Keen J. K.; Smith D. F.; Benson F. E. Identification and characterisation of a RAD51 gene from *Leishmania major*. Mol. Biochem. Parasitol. 2001, 115 (2), 209–216. 10.1016/S0166-6851(01)00288-2. 11420107

[ref48] Passos-Silva D. G.; Rajão M. A.; Nascimento De Aguiar P. H.; Vieira-Da-Rocha J. P.; Machado C. R.; Furtado C. Overview of DNA repair in *Trypanosoma cruzi*, *Trypanosoma brucei*, and *Leishmania major*. J. Nucleic Acids 2010, 2010, 840768 10.4061/2010/840768. 20976268 PMC2952945

[ref49] Genois M. M.; Paquet E. R.; Laffitte M. C. N.; Maity R.; Rodrigue A.; Ouellette M.; Masson J. Y. DNA repair pathways in trypanosomatids: From DNA repair to drug resistance. Microbiol. Mol. Biol. Rev. 2014, 78 (1), 40–73. 10.1128/mmbr.00045-13. 24600040 PMC3957735

[ref50] Genois M.-M.; Plourde M.; Éthier C.; Roy G.; Poirier G. G.; Ouellette M.; Masson J.-Y. Roles of Rad51 paralogs for promoting homologous recombination in *Leishmania infantum*. Nucleic Acids Res. 2015, 43 (5), 2701–2715. 10.1093/nar/gkv118. 25712090 PMC4357719

[ref51] Beneke T.; Madden R.; Makin L.; Valli J.; Sunter J.; Gluenz E. A CRISPR Cas9 high-throughput genome editing toolkit for kinetoplastids. R. Soc. Open Sci. 2017, 4 (5), 170095 10.1098/rsos.170095. 28573017 PMC5451818

[ref52] Proudfoot C.; McCulloch R. Distinct roles for two RAD51 -related genes in *Trypanosoma brucei* antigenic variation. Nucleic Acids Res. 2005, 33 (21), 6906–6919. 10.1093/nar/gki996. 16326865 PMC1301600

[ref53] Glover L.; McCulloch R.; Horn D. Sequence homology and microhomology dominate chromosomal double-strand break repair in African trypanosomes. Nucleic Acids Res. 2008, 36 (8), 2608–2618. 10.1093/nar/gkn104. 18334531 PMC2377438

[ref54] Moore C. W. Cleavage of Cellular and Extracellular Saccharomyces cerevisiae DNA by Bleomycin and Phleomycin. Cancer Res. 1989, 49 (24 Pt 1), 6935–6940. 2479473

