# Supplementary material for: Synthesis and Evaluation of (Bis)benzyltetrahydroisoquinoline Alkaloids as Antiparasitic Agents
Source: JACS Au. 2024 Feb 12;4(2):847–54. doi: 10.1021/jacsau.4c00007 (PMC10900488; doi:10.1021/jacsau.4c00007)
Supplement: Supplementary file 1 — au4c00007_si_001.pdf [file au4c00007_si_001.pdf]

Synthesis and evaluation of (bis)benzyltetrahydroisoquinoline alkaloids as anti-parasitic agents

Ana Sozanschi[a], Hannah Asiki [a,b], Maiara Amaral[c,d], Erica V. de Castro Levatti[c] , Andre G Tempone*[c], Richard J. Wheeler*[b], Edward A. Anderson*[a]

[a] Chemistry Research Laboratory, Department of Chemistry, University of Oxford, 12 Mansfield Road, Oxford, OX1 3TA, UK
[b] Peter Medawar Building for Pathogen Research, Nuffield Department of Medicine, University of Oxford, OX1 3SY, UK
[c] Laboratory of Pathophysiology, Butantan Institute, Av. Vital Brazil, 1500, 05503-900, São Paulo, Brazil
[d] Instituto de Medicina Tropical, Faculdade de Medicina, Universidade de São Paulo, 05403-000, São Paulo, Brazil

*Corresponding authors:
Edward A. Anderson, Email: edward.anderson@chem.ox.ac.uk.
Richard J. Wheeler, Email: richard.wheeler@ndm.ox.ac.uk.
Andre G. Tempone, Email: andre.tempone@butantan.gov.br.

Supporting Information

Table of Contents

1. *In silico* prediction of the physicochemical and pharmacokinetic profile 2
2. Biological Evaluation 3
   General Experimental Considerations 3
   Light microscopy 3
   Animals 3
   Parasites and mammalian cell maintenance 3
   Biological activity assays 4
   Cell cycle studies 5
   Generation of mNG::RAD51 tagged cell line 5
   mNG::RAD51 nuclear foci studies 5
3. Optimization of the tandem dehalogenation/PMB deprotection 6
4. Synthesis of Boc-protected BIs for ee measurement by chiral SFC 6
5. Chemical Synthesis 7
   Materials/procedures 7
   General Experimental Procedures 8
   Experimental Procedures 10
6. NMR Spectra 43
7. References 100

1. *In silico* prediction of the physicochemical and pharmacokinetic profile

A prediction of the Absorption, Distribution, Metabolism and Excretion (ADME) properties was generated using the SwissADME Webtool (Table S1).[1] This was done in order analyse whether the hit compounds demonstrate a drug-like pharmacokinetic profile. The compounds selected were BI **(*R*)-9b** and **BBIs (*S,R*)-13** against *L. infantum* and BI (–)-laudanidine and BBI **(*R,R*)-11** against *T. cruzi* as these compounds demonstrated the most potent activity and the least mammalian cytotoxicity. The data obtained shows that compounds **(*R*)-9b**, **(*S,R*)-13** and **(*R,R*)-11** have a poor pharmacokinetic profile. The best hit (–)-laudanidine shows on the other hand a promising pharmacokinetic profile and good bioavailability.

Table S1. Predicted physicochemical and pharmacokinetic profile for hit compounds.

	**(*R*)-9b**	**(*S,R*)-13**	(–)-laudanidine **(*R*)-10a**	(–)-O-methyldauricine **(*R,R*)-11**
Formula	$C_{27}H_{31}NO_4$	$C_{38}H_{44}N_2O_6$	$C_{20}H_{25}NO_4$	$C_{39}H_{46}N_2O_6$
Molecular Weight (g mol-1)	433.54	624.77	343.42	638.79
Heavy Atoms	32	46	25	47
Aromatic Heavy Atoms	18	24	12	24
Fractions Csp3	0.33	0.37	0.40	0.38
Rotatable Bonds	8	10	5	11
H-bond Acceptors (HBA)	5	8	5	8
H-bond donors (HBD)	0	1	1	0
Molar Refractivity	130.43	188.02	101.48	192.49
TPSA (Å2)	40.16	72.86	51.16	61.86
Consensus LogP	4.42	5.56	2.95	5.84
LogS (ESOL) [2]	–5.67	–7.66	–4.10	–7.88
Solubility (mg ml^{-1})	2.15e-06	1.36e-05	2.74e-02	8.40e-06
Solubility Class	Moderate	Poor	Moderate	Poor
GI Absorption	High	High	High	High
BBB permeant	Yes	No	Yes	No
P-gp substrate	Yes	No	No	No
CYP1A2 inhibitor	No	No	No	No
CYP2C19 inhibitor	No	No	No	No
CYP2C9 inhibitor	Yes	No	No	No
CYP2D6 inhibitor	Yes	No	Yes	No
CYP3A4 inhibitor	Yes	No	Yes	No
LogKp (cm s^{-1}) (skin permeation)	–5.28	–5.35	–6.02	–5.21
Lipinski violations [3]	0	1	0	1
Ghose [4]	No, 1 violation	No, 3 violations	Yes	No, 4 violations
Veber [5]	Yes	Yes	Yes	No, 1 violation
Egan [6]	Yes	Yes	Yes	Yes
Muegge [7]	No, 1 violation	No, 2 violations	Yes	No, 2 violations
PAINS [8]	0 alerts	0 alerts	0 alerts	0 alerts
Brenk [9]	0 alerts	0 alerts	0 alerts	0 alerts
Leadlikeness [10]	No	No	Yes	No

MW = molecular weight. TPSA = topological polar surface area. GI = gastrointestinal. BBB = blood brain barrier. P-gp = permeability glycoprotein; CYP = cytochrome P450. PAINS = pan assay interference compounds.

A quick visual comparison is made possible by the Webtool generating the bioavailability radars (Figure S1). If the radar plot falls completely within the pink area, the compounds is predicted to have good oral bioavailability.

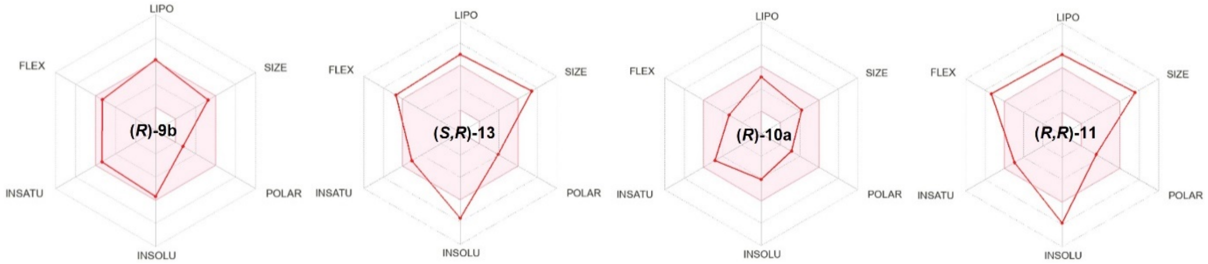

Figure S1. Bioavailability radars for hit compounds as generated by the SwissADME Webtool.

2. Biological Evaluation

General Experimental Considerations

MTT was obtained from Molecular Probes® (Invitrogen™). Foetal calf serum (FCS, 10500064), M199 medium (M199 containing Earle's salts and L-glutamine, 31100019), were purchased from Gibco. Hemin (H9039) was obtained from Sigma Aldrich. The FilterMax F5 Multi-Mode Microplate Reader spectrofluorimeter (Molecular Devices) was used to obtain all absorbance readings.

Light microscopy

Microscopy was performed with a widefield fluorescence microscope (Axio Observer A1, Zeiss) using a 63× NA 1.4 Plan-Apochromat oil immersion objective lens and a 120V metal halide fluorescence light source (Zeiss, HXP 120V) with a Hamamatsu ORCA-Flash4.0 camera. Standard fluorescence microscopy was performed with a Green (ThorLabs, MDF-GFP2) and/or Blue (Zeiss, 49) filter cube.

For microscopy of cells adhered to glass, promastigotes (1×10^8 cells) were harvested from a log-phase culture by centrifugation at 800g for 3 min, washed three times in Phosphate Buffered Saline (PBS) to improve adhesion to the glass slide and increase cell density. Hoescht 33342 (1µg mL^{-1}) was used in the first wash. The cells were re-suspended in 20 to 50 µL PBS and 1 µL was placed on a microscope slide, a coverslip was applied and immediately imaged.

Animals

The animal breeding facility of the Institute Adolfo Lutz SP provided the golden hamsters (*Mesocricetus auratus*) and BALB/c mice. The animals were maintained in a controlled environment in sterilised cages. All procedures involving the animals were done according to the Guidelines for Care and Use of Laboratory Animals of the Brazilian National Council of Animal Experimentation (COBEA). The approval of these procedures was obtained from the Animal Ethic Committee (CEUA IMT-USP 000404A) from the University of São Paulo and Instituto Adolfo Lutz – Secretary of Health of São Paulo State (CEUA 05/2018).

Parasites and mammalian cell maintenance

Leishmania (L.) mexicana promastigotes (expressing Cas9 and T7 RNA polymerase, derived from the World Health Organisation strain MNYC/BZ/62/M379)[11] were maintained in M199 medium supplemented with 10% v/v foetal calf serum, 5 mM HEPES·NaOH (pH 7.4), 26 mM $NaHCO_3$ and 5 µg mL^{-1} hemin at 28 °C. Sub-culturing was used to maintain a continuous log-phase culture with a density between 1×10^5 and 1×10^7 cells mL^{-1}.

Leishmania (L.) infantum (MHOM/BR/1972/LD) promastigotes were maintained in M199 medium supplemented with 0.25% hemin, 10% fetal bovine serum and 5% human urine at 24 °C.

Leishmania (L.) infantum (MHOM/BR/1972/LD) amastigotes were obtained from golden hamsters (*Mesocricetus auratus*) spleens following 60-70 days of infection *via* differential centrifugation. The Stauber method was used to determine the number of parasites.[12]

Trypanosoma (T.) cruzi trypomastigotes (Y strain) were maintained in Rhesus monkey kidney cells (LLC-MK2, ATCC CCL 7). Cultivation was performed in 2% fetal calf serum supplemented RPMI-1640 medium. The trypomastigotes were maintained in a humidified incubator at 37 °C and with 5% CO_2.

Murine conjunctive fibroblasts NCTC clone 929 (ATCC) were maintained in 10% foetal calf serum supplemented RPMI-1640 medium (pH 7.2) at 37 °C in a humidified incubator with 5% CO_2.

Peritoneal macrophages were obtained via washing the peritoneal cavity of BALB/c mice with 10% foetal calf serum supplemented RPMI-1640 medium (pH 7.2). The macrophages were maintained in a humidified incubator at 37 °C and with 5% CO_2.

Biological activity assays

Antipromastigote Assay (*L. infantum*)

L. infantum promastigotes (1 × 10^6 cells/well) were added to a 96-well plates. Treatment of the cells with selected compounds (serially diluted 0.14 to 200 µM) was performed in M199 medium supplemented with 0.25% hemin and 10% fetal bovine serum for 48 h at 24 °C. The MTT colorimetric assay was used to determine the activity (IC_{50}) of the compounds. Miltefosine was used as a standard drug and untreated promastigotes were used as a negative control. The half inhibitory concentration (IC_{50}) was determined from a sigmoidal regression of the concentration-response curves generated in GraphPadPrism7.

Antiamastigote Assay (*L. infantum*)

Peritoneal macrophages (1 × 10^5/well) were added to a 16-well slide chambers (NUNC) and incubated with 5% CO_2 at 37 °C. The cells were subsequently infected with *L. infantum* amastigotes (10:1 amastigotes/macrophage) for 24 h. Treatment of the cells with selected compounds (serially diluted 1.2 to 150 µM) was performed in a humidified incubator for 96 h at 37 °C and with 5% CO_2. Untreated cells were used as a negative control and miltefosine was used as a standard drug. The treated cells were fixed on slides with methanol, stained with Giemsa and observed under a light microscope.[13] The infection index was used in order to determine the half inhibitory concentration (IC_{50}) (no. infected macrophages × no. amastigotes / total no. macrophages).[14]

Antitrypomastigote Assay (*T. cruzi*)

Trypomastigotes derived from LLC-MK2 cells were added to 96-well plates. Treatment of the cells with selected compounds (serially diluted 1.2 to 150 µM) was performed in a humidified incubator in RPMI 1640 medium for 24 h at 37 °C and with 5% CO_2. Resazurin (0.011% in PBS) was added subsequently to verify parasite viability. Benznidazole was used as a standard drug. The optical density was determined at 570 nm using the FilterMax F5 (Molecular Devices).

Antiamastigote Assay (*T. cruzi*)

Peritoneal macrophages (1 × 10^5 cells/well) obtained from the peritoneal cavity of BALB/C mice were added to 16-well chambers (NUNC, Thermo Fischer Scientific). The cells were infected with trypomastigotes (10:1 parasites/macrophage) for 2 h. Then treatment of the infected macrophages with selected compounds (serially diluted 1.2 to 100 µM) was performed in a humidified incubator for 48 h at 37 °C and with 5% CO_2. The treated cells were fixed on slides using methanol, stained with Giemsa and observed under a light microscope (EVOS M5000, Thermo, USA) with digital image equipment. The infection index was used in order to determine the half inhibitory concentration (IC_{50}) (no. infected macrophages × no. amastigotes / total no. macrophages).

Mammalian toxicity

NCTC cells (6 × 10^4/well) were added to a 96-well plate. Treatments of the cells with selected compounds (serially diluted 1.5 to 200 µM) was performed in a humidified incubator for 96 h at 37 °C and with 5% CO_2. The MTT colorimetric method was used to determine the 50% cytotoxic concentration (CC_{50}) and the selectivity index (CC_{50} against NCTC cells/IC_{50} against amastigotes).[15]

Growth studies

L. mexicana promastigotes having a density of 1 × 10^6 cells mL^{-1} in M199 medium (5 mL) were treated with 5 µL of compound in DMSO (0.1% final DMSO carryover) and incubated for 24 h at 28 °C. The culture density was measured using an Improved Neubauer haemocytometer and all growth study experiments were executed at least three times. Statistical significance was derived from a two-tailed T-test.

Statistical analysis

The Graph Pad Prism 5 software was used to perform sigmoid dose-response curves for the determination of IC_{50} and CC_{50} values.

Cell cycle studies

Promastigotes (1 × 10^6 cells mL^{-1}) in 5 mL of M199 medium were treated with 5 µL of compound **(*S*)-9a** dissolved in DMSO giving a final concentration of 50 µM. At 6 h and 24 h post-treatment, a 1 mL aliquot was taken for imaging. The cell cycle experiment was preformed twice independently.

Generation of mNG::RAD51 tagged cell line

The single-stranded DNA-binding protein RAD51, associated with DNA Repair, LmxM.28.0550 was tagged with endogenous fluorescent protein mNeonGreen (mNG) at the *N*-terminus. Tagging constructs and single guide RNA (sgRNA) templates generated using the PCR method[11], with the pLPOT (mNG/Puro) plasmid as the template.[16] Reagents used for PCR are from the Expand High Fidelity PCR System dNTPack (Roche). Primers for constructs and sgRNA were designed using LeishGEdit (http://www.leishGEdit.net).

To amplify pPLOT plasmid: 1µL of each gene-specific forward and reverse primer (100 µM stock), 25 ng pLPOT plasmid, 1µL deoxyribonucleotide triphosphates (dNTP, 10 mM stock), 1µL DMSO in 20 µL H_2O were combined with 5µL $MgCl_2$ (10 ×), 19 µL H_2O and 0.5 µL HiFi Polymerase. PCR was at 94 °C for 5 min, followed by 35 cycles at 94 °C (15 sec), 65 °C (15 sec), 72 °C (2 min) then finally 72 °C (1 min).

To amplify sgRNA templates: 1µL dNTP (10 mM stock), 5µL $MgCl_2$ (10 ×), 1µL G00 sgRNA scaffold (100 µM stock), 0.5 µL HiFi Polymerase and 1µL gene-specific 5′sgRNA in 41 µL H2O were combined. PCR was at 94 °C for 30 sec, followed by 30 cycles at 94 °C (30 sec), 60 °C (30 sec), 72 °C (30 sec) then finally 72 °C (2 min). 1 µL of each PCR product was run on a 1% agarose gel to confirm the presence of expected amplified plasmid or template. Combined PCR products were precipitated for 20 min with 10 µL 3M sodium acetate and 300 µL EtOH, pelleted at 14000 min^{-1} 30 min, washed once with 70% EtOH, re-pelleted and resuspended in 10 µL H_2O.

Promastigotes (1 × 10^7 cells) were combined with PCR products and transfected using the Amaxa Nucleofector II.[17] Electroporation was performed using 2 mm cuvettes on the X-100 program, followed by immediate suspension in pre-warmed M199 medium. Successful transfectants were selected with 20 µg mL^{-1} Puromycin 15 h after transfection, taking 4 days to reach stable growth in culture.

mNG::RAD51 nuclear foci studies

mNG::RAD51 promastigotes (5 × 10^6 cells mL^{-1}) in 7 mL of M199 medium were treated with 7 µL of compound **(*S*)-9a** or **(*S*)-9b** dissolved in DMSO giving a final concentration of 50 µM. Phleomycin was used as a positive control at a final concentration of 25 µg mL^{-1}. At T = 0, 15, 30, 60 min, and 2, 6, 24 h post-treatment a 0.8 mL aliquot was taken for imaging.

3. Optimization of the tandem dehalogenation/PMB deprotection

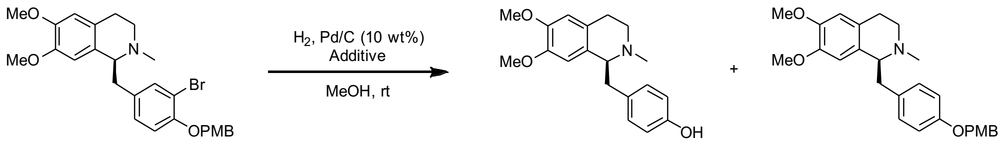

(*S*)-**9c** (*S*)-**10b** (*S*)-**10c**

Entry	H_2 balloon	Pd/C loading	Additive	Time	Temp.	Yield [a]
1	Triple balloon	10 mol%	-	48 h	rt	Incomplete conversion
2	Triple balloon	10 mol%	-	24 h	40 °C	73% (S)-10c
3	Triple balloon	14 mol%	Et_3N (1.5 equiv.)	48 h	rt	Mixture
4	Triple balloon	14 mol%	Et_3N (24 equiv.)	48 h	rt	Mixture
5	Triple balloon	10 mol%	AcOH (2.2 equiv.)	24 h	rt	>99% **(*S*)-10c**
6 [c]	Gas bag [b]	14 mol%	Et_3N (10 equiv.)	30	rt	90% **(*S*)-10b**

[a] Yields of isolated products. [b] H_2 gas was bubbled through the reaction mixture for the stated time; the gas bag was purchased from Sigma Aldrich with a capacity of 1 gallon. [c] General Procedure F.

4. Synthesis of Boc-protected BIs for ee measurement by chiral SFC

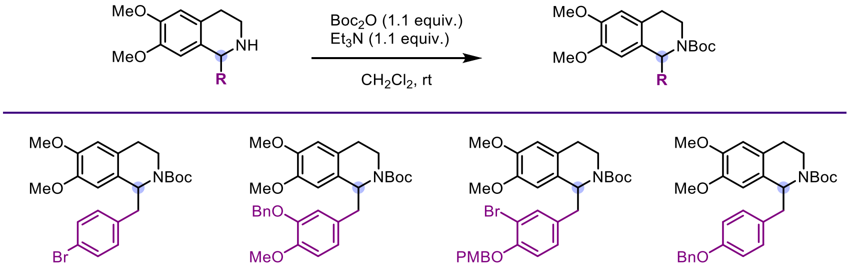

rac-**S1**, **96%**
(*S*)-S1, **86%** 94% *ee*
(*R*)-S1, **69%** 99% *ee*

rac-**S2**, **55%**
(*S*)-S2, **70%** 94% *ee*
(*R*)-S2, **51%** 96% *ee*

rac-**S3**, **71%**
(*S*)-S3, **39%** 96% *ee*
(*R*)-S3, **85%** 92% *ee*

rac-**S4**, **84%**
(*S*)-S4, **90%** 96% *ee*
(*R*)-S4, **76%** 94% *ee*

Compounds **S1** – **S4** were made from the corresponding secondary amines *via* **General Procedure H**, as to allow separation of enantiomers and *ee* measurement by chiral SFC. Associated chromatograms can be found in **Section 5** of the Supporting Information document.

5. Chemical Synthesis

Materials/procedures

All air- or moisture-sensitive reactions were performed under a nitrogen atmosphere, with anhydrous solvents and in oven-dried glassware or glassware dried under vacuum and heated using a heat gun. Commercial reagents were obtained from Acros Organics, Alfa Aesar, Fluorochem, Fisher Scientific, Merck, Sigma Aldrich or TCI and were used without any further purification unless otherwise stated. Anhydrous solvents were collected fresh from an MBraun SPS-800 solvent purification system. Additionally, for Ullmann cross-couplings, pyridine was distilled from CaH_2 and stored over NaOH pellets for no longer than 7 days. RuCl[TsDPEN(*p*-cymene)] was stored and weighed out in the glove box under a nitrogen atmosphere. Heating was performed using an oil bath. The gas bag with a capacity of 1 gallon was purchased from Sigma Aldrich.

Infrared spectra were obtained in $CHCl_3$ as the solvent using a Bruker Tensor 27 Fourier FT-IR spectrometer. Samples were prepared as a thin film on a diamond PIKE Miracle ATR module and the absorbance maxima (ν_{max}) are reported in cm^{-1}.

High-resolution mass spectra (HRMS) were recorded by the Departmental Mass Spectrometry Service, University of Oxford on a Thermo Exactive or Bruker MicroTOF Mass Spectrometer. Values are calculated to 4 decimal places from the molecular formula, and all values reported are within a tolerance of 5 ppm.

^{1}H (proton) and ^{13}C (carbon) NMR spectra were recorded on Bruker AVIII HD 400, AVIII HD 500, AVII 500 and AVIII HD 600 spectrometers. Chemical shifts are quoted in parts per million (ppm) to the nearest 0.01 ppm for ^{1}H NMR spectra, and 0.1 ppm for ^{13}C NMR spectra. NMR spectra were calibrated to the residual solvent peak stated: $CDCl_3$ (δ_H = 7.26 / δ_C = 77.16) or methanol-d_4 (δ_H = 3.31 / δ_C = 49.00). Peak multiplicities are defined as s (singlet), d (doublet), dd (doublet of doublets), dt (double triplet), t (triplet), q (quartet), m (multiplet) and br (broad). Coupling constants (*J*) are reported to the nearest 0.1 Hz.

Optical rotations were obtained using a Perkin Elmer 241 Polarimeter (using the sodium D line, 589 nm) with a path length of 1 dm at 25 °C. $[\alpha]_D^{25}$ are reported in units of 10^{-1} deg cm^2 g^{-1} and the concentrations (c) are reported in g/100 mL.

Column chromatography refers to normal phase column chromatography and was performed on silica gel (Merck, Si 60, 0.040 – 0.063 mm) using the solvent system stated and under a positive pressure. Preparative thin-layer chromatography was performed on silica gel GF uniplates with fluorescent indicator, (500 µm, 20 × 20 cm) from Miles Scientific (Analtech).

Analytical thin-layer chromatography (TLC) was used to monitor reaction progress and performed on pre-coated aluminium-backed plates (Merck Kieselgel 60 F254 plates). Visualisation was performed using ultraviolet light (254 nm) and/or by staining with potassium permanganate, vanillin, ninhydrin, phosphomolybdic acid (PMA) or Hanessian′s stain (CAM).

Chiral supercritical fluid chromatography (SFC) separations were conducted on a Waters Acquity UPC2 system using Waters Empower software. The Chiralpak® column IB (150 × 3 mm, 3 µm particle size) was used at 1500 PSI, 30 ºC, flow: 1.5 ml/min under the gradient specified in the text. The solvents used were of HPLC grade (obtained from Fisher Scientific, Sigma Aldrich or Rathburn).

No unexpected or unusually high safety hazards were encountered.

General Experimental Procedures

General Procedure A: Amide coupling

Adapted from the procedure of Mäder et al.[18] To a solution of carboxylic acid (1.0 equiv.) in anhydrous CH_2Cl_2 (3.3 mL/mmol of carboxylic acid) at 0 ºC under an inert atmosphere was added 1-ethyl-3-(3-dimethylaminopropyl) carbodiimide hydrochloride (1.5 equiv.) and hydroxybenzotriazole hydrate (0.5 equiv.) and the mixture was stirred for 10 min. Amine (1.1 equiv.) and triethylamine (1.1 equiv.) were added to the mixture at 0 ºC, then the mixture was warmed to room temperature and stirred for 18 h. The reaction mixture was washed sequentially with H_2O, HCl (aq., 1 M) and $NaHCO_3$ (aq., sat.). The combined organic extracts were washed with brine, dried ($MgSO_4$), filtered and concentrated *in vacuo*. Flash column chromatography afforded the desired amides.

General Procedure B: Bischler-Napieralski reaction

Adapted from the procedure of Movassaghi et al.[19] Trifluoromethanesulfonic anhydride (1.1 equiv.) was added via syringe over 1 min to a stirred solution of amide (1.0 equiv.) and 2-chloropyridine (1.2 equiv.) in anhydrous CH_2Cl_2 (5 mL/mmol of amide) at −78 ºC under an inert atmosphere. After 20 min the reaction mixture was warmed to room temperature and stirred for 1 h. Triethylamine (1.0 equiv.) was then added to neutralise the triflate salts. The mixture was concentrated *in vacuo* and immediately submitted to the next step without further purification.

General Procedure C: $NaBH_4$ reduction of imines

To a solution of crude imine in EtOH (5 mL/mmol of imine) at 0 ºC under an inert atmosphere was added sodium borohydride (3.0 equiv.). The mixture was warmed to room temperature and stirred for 18 h. The mixture was then cooled to 0 ºC and H_2O was added until gas evolution ceased. The mixture was extracted with CH_2Cl_2 (× 3), and the combined organic extracts were dried ($MgSO_4$), filtered and concentrated *in vacuo*. Purification by flash column chromatography afforded the desired amines.

General Procedure D: Asymmetric transfer hydrogenation (ATH) of imines

Adapted from the procedure of Uematsu et al.[20] To a flask containing the crude imine (1.0 equiv.) under an inert atmosphere was added RuCl[(*R,R*)-TsDPEN](p-cymene) or RuCl[(*S,S*)-TsDPEN](p-cymene) (0.07 equiv.) as a solution in anhydrous DMF (5 mL/mmol of amide) and the mixture was cooled to 0 ºC. An azeotrope mixture of $HCO_2H{:}Et_3N$ (5:2 v/v, 0.5 mL/mmol imine) was added slowly, then the mixture was warmed to room temperature and stirred for 18 h. The reaction was quenched by addition of $NaHCO_3$ (aq., sat.,) and the aqueous phase was extracted with EtOAc (×3). The combined organic extracts were washed with H_2O (×3) and brine (×2), dried ($MgSO_4$), filtered and concentrate *in vacuo*. Flash column chromatography afforded the desired tetrahydroisoquinolines.

General Procedure E: *N*-Methylation

Adapted from the procedure of Blank et al.[21] To a solution of tetrahydroisoquinoline (1.0 equiv.) in MeOH (20 mL/mmol of tetrahydrosioquinoline) was added formalin (37% aq., 28 equiv.) and the resulting mixture was stirred for 20 min. The reaction mixture was then cooled to 0 ºC for 10 min and sodium borohydride (3.0 equiv.) was added portion-wise over two min. The mixture was warmed to room temperature and stirred for 1 h. The mixture was concentrated in vacuo, diluted with CH_2Cl_2, washed with NaOH (aq., 1 M), dried (Na_2SO_4), filtered and concentrated *in vacuo*. Flash column chromatography afforded the desired *N*-methyl tetrahydroisoquinolines.

General Procedure F: Pd/C catalysed hydrogenation

To an oven-dried flask containing Pd/C (10% wt., 0.14 – 0.20 equiv.) under an inert atmosphere, was added anhydrous MeOH and the flask was evacuated and backfilled with hydrogen (×3). The substrate (1.0 equiv.) was then added as a solution in MeOH followed by the addition of triethylamine (10 equiv.). Using a gas bag,

hydrogen was continuously bubbled through the reaction mixture by having the needle submerged in the reaction mixture and a second exit needle for the stated time. The gas bag was refilled as needed. Upon completion, the reaction mixture was filtered through a pad of Celite® and the filter cake was washed with CH_2Cl_2/MeOH (8:2). The filtrate was concentrated *in vacuo* to afford the desired phenols. No further purification was necessary.

General Procedure G: Ullmann cross-coupling

To a flask containing bromide (1.0 equiv.) and phenol (1.1 equiv.) was added potassium carbonate (2.5 equiv.) and the flask was evacuated and backfilled with nitrogen (×3). After addition of anhydrous pyridine (0.65 – 1.6 mL/mmol bromide), the mixture was heated to 120 °C. Cupric oxide (2.0 equiv.) was added under a flow of nitrogen and the mixture was heated to 140 °C for 24 h. The reaction mixture was cooled to room temperature, diluted with CH_2Cl_2 and the organic phase was washed with ammonia (aq., 35%, ×4). The combined organic extracts were washed with brine, dried ($MgSO_4$), filtered and concentrated *in vacuo*. The pyridine was removed azeotropically with toluene. Purification preparative TLC (CH_2Cl_2/7N ammonia solution in MeOH, 90:5) afforded the desired BBIs.

General Procedure H: *N*-Boc protection

To a solution of tetrahydroisoquinoline (1.0 equiv.) in anhydrous CH_2Cl_2 (6 mL/mmol of tetrahydroisoquinoline) at room temperature under an inert atmosphere was added di-tert-butyl dicarbonate (1.2 equiv.) and triethylamine (1.2 equiv.), and the reaction mixture was stirred for 4 h. Upon completion, the mixture was concentrated *in vacuo*. Flash column chromatography afforded the desired *N*-Boc protected tetrahydroisoquinolines.

Experimental Procedures

2-(3-Bromo-4-hydroxyphenyl)acetic acid (5c)

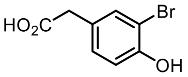

Adapted from the procedure of Weicheng et al.[22] To a solution of 4-hydroxyphenylacetic acid **5d** (5.00 g, 32.9 mmol, 1.0 equiv.) and *p*-toluensulfonic acid (6.56 g, 34.5 mmol, 1.05 equiv.) in acetonitrile (66 mL) at −15 ºC (1:2 w/w NaCl/ice cooling bath) was added *N*-bromosuccinimide (5.85 g, 32.9 mmol, 1.0 equiv.) and the mixture was stirred at −15 ºC for 2.5 h. The reaction mixture was then filtered and concentrated *in vacuo*. The residue was diluted with EtOAc (100 mL), washed sequentially with $Na_2S_2O_4$ (aq., sat., 2 × 100 mL) and H_2O (2 × 70 mL). The combined organic extracts were dried (Na_2SO_4), filtered and concentrated *in vacuo* to afford the title compound as a white solid (7.27 g, 31.5 mmol, 96%). No further purification was necessary.

^{1}H NMR (400 MHz, methanol-*d*4) δ 7.38 (d, *J* = 2.2 Hz, 1H), 7.06 (dd, *J* = 8.3, 2.2 Hz, 1H), 6.83 (d, *J* = 8.3 Hz, 1H), 4.97 (s, br, 1H), 3.49 (s, 2H).
^{13}C NMR (101 MHz, methanol-*d*4) δ 174.2, 152.9, 133.4, 129.2, 127.2, 115.7, 109.2, 39.1.
Spectroscopic data is in agreement with that reported previously.[21]

2-(4-Bromophenyl)-*N*-(3,4-dimethoxyphenethyl)acetamide (6a)

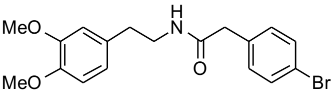

2-(3,4-Dimethoxyphenyl)ethan-1-amine **4** (860 µL, 5.12 mmol), 4-bromophenylactic acid **5a** (1.00 g, 4.65 mmol), triethylamine (710 µL, 5.12 mmol), hydroxybenzotriazole hydrate (315 mg, 2.33 mmol) and 1-ethyl-3-(3-dimethylaminopropyl)carbodiimide hydrochloride (1.34 g, 6.98 mmol) in anhydrous CH_2Cl_2 (16 mL) were submitted to **General Procedure A**. Purification by flash column chromatography (dry loading, EtOAc) afforded the title compound as an off-white amorphous solid (1.52 g, 4.40 mmol, 95%).

^{1}H NMR (400 MHz, $CDCl_3$) δ 7.43 (d, *J* = 8.4 Hz, 1H), 7.05 (d, *J* = 8.4 Hz, 1H), 6.73 (d, *J* = 8.1 Hz, 1H), 6.61 (d, *J* = 2.1 Hz, 1H), 6.51 (dd, *J* = 8.1, 2.1 Hz, 1H), 5.30 (s, br, 1H), 3.87 (s, 3H), 3.83 (s, 3H), 3.50 – 3.40 (m, 4H), 2.68 (t, *J* = 6.8 Hz, 2H).
^{13}C NMR (101 MHz, $CDCl_3$) δ 170.3, 149.3, 147.9, 133.9, 132.2(2C), 131.2(2C), 131.1, 121.5, 120.7, 111.9, 111.4, 56.1, 56.0, 43.4, 40.8, 35.1.
Spectroscopic data is in agreement with that reported previously.[18]

***N*-(3,4-Dimethoxyphenethyl)-2-(3-hydroxy-4-methoxyphenyl)acetamide (6b)**

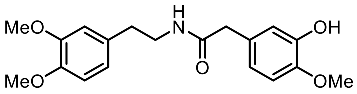

2-(3,4-Dimethoxyphenyl)ethan-1-amine **4** (860 µL, 5.58 mmol), 3-hydroxy-4-methoxyphenylacetic acid **5b** (1.00 g, 4.65 mmol), triethylamine (713 µL, 5.12 mmol), hydroxybenzotriazole hydrate (315 mg, 2.33 mmol) and 1-ethyl-3-(3-dimethylaminopropyl)carbodiimide hydrochloride (1.34 g, 6.98 mmol) in anhydrous CH_2Cl_2 (16 mL) were submitted to **General Procedure A**. Purification by trituration (cold EtOAc) afforded the title compound as an off-white amorphous solid (1.36 g, 3.95 mmol, 85%).

^{1}H NMR (400 MHz, $CDCl_3$) δ 6.78 – 6.70 (m, 3H), 6.65 – 6.53 (m, 3H), 5.97 (s, br, 1H), 5.49 (s, br, 1H), 3.87 (s, 3H), 3.84 (s, 3H), 3.81 (s, 3H), 3.47 – 3.37 (m, 4H), 2.66 (t, *J* = 6.9 Hz, 2H).

^{13}**C NMR** (101 MHz, $CDCl_3$) δ 171.5, 149.0, 147.7, 146.1(2C), 131.2, 127.8, 121.0, 120.8, 115.9, 111.9, 111.4, 111.2, 56.0, 55.9, 55.9, 43.2, 40.8, 35.1.
Spectroscopic data is in agreement with that reported previously.[23]

2-(3-Bromo-4-hydroxyphenyl)-N-(3,4-dimethoxyphenethyl)acetamide (6c)

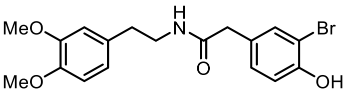

2-(3,4-Dimethoxyphenyl)ethan-1-amine **4** (375 mg, 2.07 mmol), 2-(3-bromo-4-hydroxy phenyl)acetic acid **5c** (435 mg, 1.88 mmol), triethylamine (289 µL, 2.07 mmol), hydroxybenzotriazole hydrate (127 mg, 0.940 mmol) and 1-ethyl-3-(3-dimethylaminopropyl) carbodiimide hydrochloride (540 mg, 2.82 mmol) in anhydrous CH_2Cl_2 (6.3 mL) were submitted to **General Procedure A**. Purification by flash column chromatography (EtOAc) afforded the title compound as an off-white amorphous solid (630 mg, 1.60 mmol, 85%).

m.p. 146 – 147 ºC.
^{1}**H NMR** (500 MHz, $CDCl_3$) δ 7.29 (d, *J* = 2.1 Hz, 1H), 7.00 (dd, *J* = 8.3, 2.1 Hz, 1H), 6.94 (d, *J* = 8.3 Hz, 1H), 6.74 (d, *J* = 8.2 Hz, 1H), 6.62 (d, *J* = 2.0 Hz, 1H), 6.55 (dd, *J* = 8.2, 2.0 Hz, 1H), 5.76 (s, br, 1H), 5.36 (s, br, 1H), 3.86 (s, 3H), 3.83 (s, 3H), 3.46 (q, *J* = 6.7 Hz, 2H), 3.42 (s, 2H), 2.69 (t, *J* = 6.7 Hz, 2H).
^{13}**C NMR** (126 MHz, $CDCl_3$) δ 170.7, 151.8, 149.2, 147.9, 132.9, 131.1, 130.3, 128.4, 120.8, 116.7, 111.8, 111.4, 110.6, 56.1, 56.0, 42.6, 40.9, 35.1.
IR (film) v_{max}/cm^{-1} 2957, 2837, 1646, 1515, 1464, 1261, 1236, 1157, 1141, 1028, 912.
HRMS (ESI$^+$) $C_{18}H_{20}Br^{79}NO_4$ found $[M+H]^+$ at 394.0649, expected at 394.0648.
Some spectroscopic data has been reported previously.[24]

2-(3-(Benzyloxy)-4-methoxyphenyl)-*N*-(3,4-dimethoxyphenethyl)acetamide (7a)

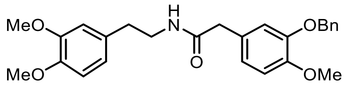

To a solution of *N*-(3,4-dimethoxyphenethyl)-2-(3-hydroxy-4-methoxyphenyl)acetamide **6b** (500 mg, 1.45 mmol, 1.0 equiv.) in acetone (7.5 mL) were added potassium carbonate (301 mg, 2.18 mmol, 1.5 equiv.), tetrabutylammonium iodide (27.0 mg, 73.1 µmol, 0.05 equiv.) and benzyl bromide (210 µL, 1.74 mmol, 1.2 equiv.) and the mixture was stirred for 30 h. The mixture was concentrated *in vacuo* and the residue was dissolved in CH_2Cl_2 (10 mL). The organic phase was washed sequentially with HCl (aq., 1M, 10 mL), NaOH (aq., 1M, 10 mL), brine (15 mL), dried ($MgSO_4$), filtered and concentrated *in vacuo*. Purification by flash column chromatography (dry loading, CH_2Cl_2/acetone, 9:1) afforded the title compound as a white solid (621 mg, 1.43 mmol, 85%).

m.p. 85 – 87 ºC.
^{1}**H NMR** (500 MHz, $CDCl_3$) δ 7.42 (d, *J* = 7.2 Hz, 2H), 7.38 – 7.33 (m, 2H), 7.31 – 7.27 (m, 1H), 6.82 (d, *J* = 8.1 Hz, 1H), 6.73 – 6.68 (m, 3H), 6.60 (d, *J* = 2.0 Hz, 1H), 6.51 (dd, *J* = 8.1, 2.0 Hz, 1H), 5.30 (s, br, 1H), 5.10 (s, 2H), 3.89 (s, 3H), 3.82 (s, 6H), 3.42 (s, 2H), 3.39 (q, *J* = 6.9 Hz, 2H), 2.62 (t, *J* = 6.9 Hz, 2H).
^{13}**C NMR** (151 MHz, $CDCl_3$) δ 171.2, 149.17, 149.15, 148.5, 147.8, 137.0, 131.2, 128.7(2C), 128.1, 127.5(2C), 127.2, 122.4, 120.7, 115.4, 112.3, 111.9, 111.3, 71.1, 56.2, 56.0, 55.9, 43.5, 40.8, 35.2.
IR (film) v_{max}/cm^{-1} 3295, 2933, 2835, 2360, 1646, 1589, 1513, 1454.
HRMS (ESI$^+$) $C_{26}H_{29}NO_5$ found $[M+H]^+$ at 436.2118, expected at 436.2117.

2-(3-Bromo-4-((4-methoxybenzyl)oxy)phenyl)-*N*-(3,4-dimethoxyphenethyl)acetamide (7b)

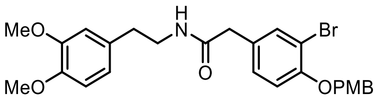

To a solution of 2-(3-bromo-4-hydroxyphenyl)-*N*-(3,4-dimethoxyphenethyl)acetamide **3c** (3.30 g, 8.37 mmol, 1.0 equiv.) and potassium carbonate (2.89 g, 20.9 mmol, 2.5 equiv.) in DMF (12 mL) was added 4-methoxybenzyl chloride (1.36 mL, 10.0 mmol, 1.2 equiv.). The reaction mixture was heated to 80 ºC for 18 h. Upon completion, the mixture was diluted with EtOAc (40 mL) and washed sequentially with $NaHCO_3$ (aq., sat., 10 mL), HCl (aq., 1 M, 15 mL) and H_2O (2 × 10 mL). The combined organic extracts were washed with brine (20 mL), dried ($MgSO_4$), filtered and concentrated *in vacuo*. Purification by flash column chromatography (dry-loading, EtOAc/pentane, 2:1 → 1:0) afforded the title compound as an off-white solid (3.75 g, 7.29 mmol, 87%).

m.p. 113 – 114 ºC.
^{1}H NMR (500 MHz, $CDCl_3$) δ 7.42 – 7.36 (m, 3H), 7.03 (dd, *J* = 8.4, 2.2 Hz, 1H), 6.92 (d, *J* = 8.7 Hz, 2H), 6.86 (d, *J* = 8.4 Hz, 1H), 6.74 (d, *J* = 8.1 Hz, 1H), 6.62 (d, *J* = 2.1 Hz, 1H), 6.54 (dd, *J* = 8.1, 2.1 Hz, 1H), 5.37 (s, br, 1H), 5.07 (s, 2H), 3.84 (s, 3H), 3.83 (s, 3H), 3.81 (s, 3H), 3.45 (q, *J* = 6.7 Hz, 2H), 3.42 (s, 2H), 2.68 (t, *J* = 6.7 Hz, 2H).
^{13}C NMR (126 MHz, $CDCl_3$) δ 170.6, 159.6, 154.5, 149.2, 147.8, 134.3, 131.1, 129.5, 128.9(2C), 128.7, 128.5, 120.8, 114.3, 114.2(2C), 113.0, 111.8, 111.4, 70.9, 56.05, 55.98, 55.4, 42.7, 40.8, 35.2.
IR (film) ν_{max}/cm^{-1} 2937, 2835, 1648, 1312, 1515, 1494, 1246, 1029, 735.
HRMS (ESI$^+$) $C_{26}H_{28}Br^{79}NO_5$ found $[M+H]^+$ at 514.1244, expected at 514.1224.

2-(4-(Benzyloxy)phenyl)-N-(3,4-dimethoxyphenethyl)acetamide (7c)

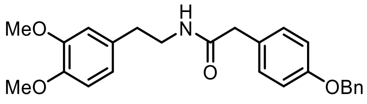

2-(3,4-Dimethoxyphenyl)ethan-1-amine **4** (2.44 mL, 14.5 mmol), 4-hydroxyphenylacetic acid **5d** (2.00 g, 13.1 mmol), triethylamine (2.20 mL, 15.7 mmol), hydroxybenzotriazole hydrate (885 mg, 6.55 mmol) and 1-ethyl-3-(3-dimethylaminopropyl) carbodiimide hydrochloride (3.77 g, 19.7 mmol) in anhydrous CH_2Cl_2 (66 mL) were submitted to **General Procedure A**. To a solution of the crude amide in acetone (66 mL) was added potassium carbonate (2.72 g, 19.7 mmol, 1.5 equiv.), tetrabutylammonium iodide (242 mg, 0.655 mmol, 0.05 equiv.) and benzyl bromide (1.87 mL, 15.7 mmol, 1.2 equiv.), and the mixture stirred for 24 h. The reaction mixture was concentrated *in vacuo.* The residue was diluted with CH_2Cl_2 (30 mL) and washed sequentially with HCl (aq., 1 M, 10 mL) and $NaHCO_3$ (aq., sat., 10 mL). The combined organic extracts were washed with brine (15 mL), dried ($MgSO_4$), filtered and concentrated *in vacuo*. Purification by flash column chromatography (dry loading, CH_2Cl_2/acetone, 9:1) afforded the title compound as a white solid (3.47 g, 1.43 mmol, 65% over two steps).

^{1}H NMR (500 MHz, $CDCl_3$) δ 7.44 (d, *J* = 7.0 Hz, 2H), 7.41 – 7.37 (m, 2H), 7.36 – 7.31 (m, 1H), 7.07 (d, *J* = 8.6 Hz, 2H), 6.91 (d, *J* = 8.6 Hz, 2H), 6.72 (d, *J* = 8.1 Hz, 1H), 6.61 (d, *J* = 2.0 Hz, 1H), 6.55 (dd, *J* = 8.1, 2.0 Hz, 1H), 5.35 (s, br, 1H), 5.06 (s, 2H), 3.84 (s, 3H) 3.83 (s, 3H), 3.47 (s, 2H), 3.44 (q, *J* = 6.8 Hz, 2H), 2.67 (t, *J* = 6.8 Hz, 2H).
^{13}C NMR (126 MHz, $CDCl_3$) δ 171.4, 158.2, 149.2, 147.8, 137.0, 131.3, 130.7(2C), 128.8(2C), 128.2, 127.6(2C), 127.2, 120.7, 115.5(2C), 111.9, 111.4, 70.2, 56.1, 56.0, 43.1, 40.9, 35.2.
Spectroscopic data is in agreement with that reported previously.[25]

1-(4-Bromobenzyl)-6,7-dimethoxy-1,2,3,4-tetrahydroisoquinoline (rac-8a)

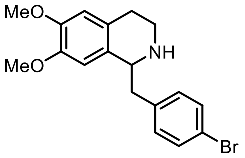

2-(4-Bromophenyl)-*N*-(3,4-dimethoxyphenethyl)acetamide **6a** (60 mg, 0.16 mmol), 2-chloropyridine (18 µL, 0.19 mmol), and trifluoromethanesulfonic anhydride (30 µL, 0.17 mmol) in CH_2Cl_2 (0.8 mL) were submitted to **General Procedure B**.

The crude imine and sodium borohydride (18 mg, 0.48 mmol) in EtOH (0.8 mL) were submitted to **General Procedure C**. Purification by flash column chromatography (EtOAc/pentane/7N ammonia solution in MeOH, 7:1:0.1 → 1:0:0.1) afforded the title compound as a yellow oil (31 mg, 86 µmol, 53% over 2 steps).

^{1}H NMR (500 MHz, $CDCl_3$) δ 7.44 (d, *J* = 8.3 Hz, 1H), 7.13 (d, *J* = 8.3 Hz, 1H), 6.54 (s, 1H), 6.06 (s, 1H), 4.13 (dd, *J* = 9.4, 4.5 Hz, 1H), 3.86 (s, 3H), 3.81 (s, 3H), 3.23 – 3.11 (m, 2H), 2.97 – 2.85 (m, 2H), 2.80 – 2.62 (m, 2H), 2.00 (s, br, 1H).

^{13}C NMR (126 MHz, $CDCl_3$) δ 147.7, 147.2, 138.2, 131.8(2C), 131.3(2C), 130.1, 127.4, 120.5, 112.0, 109.5, 56.8, 56.1, 56.0, 42.3, 40.8, 29.5.

Spectroscopic data is in agreement with that reported previously.[26]

(*S*)-1-(4-Bromobenzyl)-6,7-dimethoxy-1,2,3,4-tetrahydroisoquinoline ((*S*)-8a)

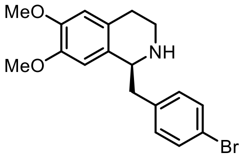

2-(4-Bromophenyl)-*N*-(3,4-dimethoxyphenethyl)acetamide **6a** (0.25 g, 0.66 mmol), 2-chloropyridine (70 µL, 0.79 mmol) and trifluoromethanesulfonic anhydride (0.12 mL, 0.73 mmol) in anhydrous CH_2Cl_2 (3.3 mL) were submitted to **General Procedure B**.

The crude imine, RuCl[(*R,R*)-TsDPEN](*p*-cymene) (29 mg, 0.046 mmol) and HCO_2H/Et_3N (5:2 v/v, 0.33 mL) in anhydrous DMF (3.3 mL) were submitted to **General Procedure D**. Purification by flash column chromatography (EtOAc/pentane/7N ammonia solution in MeOH, 7:1:0.1 → 1:0:0.1) afforded the title compound as a yellow oil (0.22 g, 0.62 mmol, 94% over 2 steps, 94% *ee*, after Boc derivatisation).

^{1}H NMR (400 MHz, $CDCl_3$) δ 7.44 (d, *J* = 8.3 Hz, 1H), 7.13 (d, *J* = 8.3 Hz, 2H), 6.59 (2s, 2H), 4.12 (dd, *J* = 9.5, 4.4 Hz, 1H), 3.86 (s, 3H), 3.82 (s, 3H), 3.24 – 3.10 (m, 2H), 2.98 – 2.82 (m, 2H), 2.80 – 2.63 (m, 2H), 1.64 (s, br, 1H).

^{13}C NMR (101 MHz, $CDCl_3$) δ 147.7, 147.2, 138.3, 131.8(2C), 131.3(2C), 130.3, 127.6, 120.4, 112.0, 109.5, 56.9, 56.1, 56.0, 42.4, 40.8, 29.6.

$[\alpha]_D^{25}$ –12.8 (c. 1.2, $CHCl_3$). Ref[21] $[\alpha]_D^{25}$ –8.5 (c. 1.0, $CHCl_3$), 95% *ee.*

Spectroscopic data is in agreement with that reported previously.[26]

(*R*)-1-(4-Bromobenzyl)-6,7-dimethoxy-1,2,3,4-tetrahydroisoquinoline ((*R*)-8a)

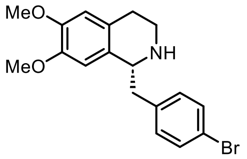

2-(4-Bromophenyl)-*N*-(3,4-dimethoxyphenethyl)acetamide **6a** (600 mg, 1.59 mmol), 2-chloropyridine (180 µL, 1.91 mmol) and trifluoromethanesulfonic anhydride (290 µL, 1.74 mmol) in anhydrous CH_2Cl_2 (8.0 mL) were submitted to **General Procedure B**.
The crude imine, RuCl[(*S,S*)-TsDPEN](*p*-cymene) (71.0 mg, 0.11 mmol) and HCO_2H/Et_3N (5:2 v/v, 0.81 mL) in anhydrous DMF (8.0 mL) were submitted to **General Procedure D**. Purification by flash column chromatography (EtOAc/pentane/7N ammonia solution in MeOH, 7:1:0.1 → 1:0:0.1) afforded the title compound as a yellow oil (494 mg, 1.36 mmol, 86% over 2 steps, 99% *ee* after Boc derivatisation).

^{1}H NMR (400 MHz, $CDCl_3$) δ 7.45 (d, *J* = 8.3 Hz, 1H), 7.13 (d, *J* = 8.3 Hz, 2H), 6.59 (s, 2H, H5), 4.13 (dd, *J* = 9.5, 4.4 Hz, 1H), 3.86 (s, 3H), 3.82 (s, 3H), 3.24 – 3.11 (m, 2H), 2.98 – 2.83 (m, 2H), 2.79 – 2.64 (m, 2H), 1.68 (s, br, 1H).
^{13}C NMR (126 MHz, $CDCl_3$) δ 147.7, 147.2, 138.3, 131.8(2C), 131.3(2C), 130.3, 127.6, 120.5, 112.0, 109.5, 56.9, 56.1, 56.0, 42.4, 40.9, 29.6.
IR (film) ν_{max}/cm^{-1} 2997, 2931, 1610, 1514, 1487, 1464, 1448.
HRMS (ESI$^+$) $C_{18}H_{20}Br^{79}NO_2$ found $[M+H]^+$ at 362.0748, expected at 362.0750.
$[\alpha]_D^{25}$ +13.5 (c. 1.11, $CHCl_3$).

1-(3-(Benzyloxy)-4-methoxybenzyl)-6,7-dimethoxy-1,2,3,4-tetrahydroisoquinoline (rac-8b)

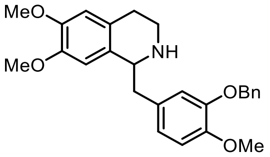

2-(3-(Benzyloxy)-4-methoxyphenyl)-*N*-(3,4-dimethoxyphenethyl)acetamide **7a** (87 mg, 0.20 mmol), 2-chloropyridine (20 µL, 0.24 mmol) and trifluoromethanesulfonic anhydride (40 µL, 0.22 mmol) in anhydrous CH_2Cl_2 (1.0 mL) were submitted to **General Procedure B**.
The crude imine and sodium borohydride (23 mg, 0.60 mmol) in EtOH (1.0 mL) were submitted to **General Procedure C**. Purification by flash column chromatography (EtOAc/pentane/7N ammonia solution in MeOH, 7:1:0.1 → 1:0:0.1) afforded the title compound as a yellow oil (60 mg, 0.14 mmol, 72% over 2 steps).

^{1}H NMR (400 MHz, $CDCl_3$) δ 7.47 – 7.39 (m, 2H), 7.38 – 7.33 (m, 2H), 7.31 – 7.25 (m, 1H), 6.85 (d, *J* = 8.1 Hz, 1H), 6.80 – 6.75 (m, 2H), 6.61 (s, 1H), 6.58 (s, 1H), 5.13 (d, *J* = 2.8 Hz, 2H), 4.04 (dd, *J* = 9.2, 4.2 Hz, 1H), 3.88 (s, 3H), 3.85 (s, 3H), 3.82 (s, 3H), 3.21 – 3.05 (m, 2H), 2.88 – 2.59 (m, 4H), 1.72 (s, br, 1H).
^{13}C NMR (101 MHz, $CDCl_3$) δ 148.5, 148.1, 147.5, 147.1, 137.2, 131.5, 130.6, 128.6(2C), 127.9, 127.6, 127.4(2C), 122.2, 115.4, 112.1, 111.9, 109.4, 71.1, 56.9, 56.2, 56.1, 56.0, 42.2, 41.0, 29.7.
IR (film) ν_{max}/cm^{-1} 2951, 2833, 2360, 2341, 1609, 1588, 1514, 1261.
HRMS (ESI$^+$) $C_{26}H_{29}NO_4$ found $[M+H]^+$ at 420.2165, expected at 410.2169.
Some spectroscopic data has been reported previously.[27]

(*S*)-1-(3-(Benzyloxy)-4-methoxybenzyl)-6,7-dimethoxy-1,2,3,4-tetrahydroisoquinoline ((*S*)-8b)

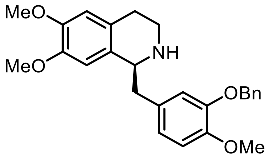

2-(3-(Benzyloxy)-4-methoxyphenyl)-*N*-(3,4-dimethoxyphenethyl)acetamide **7a** (0.40 g, 0.92 mmol), 2-chloropyridine (0.10 mL, 1.1 mmol) and trifluoromethanesulfonic anhydride (0.17 mL, 1.0 mmol) in anhydrous CH_2Cl_2 (4.6 mL) were submitted to **General Procedure B**.

The crude imine, RuCl[(*R,R*)-TsDPEN](*p*-cymene) (41 mg, 64 μmol), HCO_2H/Et_3N (5:2 v/v, 0.46 mL) in anhydrous DMF (4.6 mL) were submitted to **General Procedure D**. Purification by flash column chromatography (EtOAc/pentane/7N ammonia solution in MeOH, 7:1:0.1 → 1:0:0.1) afforded the title compound as a yellow oil (0.24 g, 0.58 mmol, 63% over 2 steps, 94% *ee* after Boc derivatisation).

^{1}H NMR (400 MHz, $CDCl_3$) δ 7.45 – 7.40 (m, 2H), 7.38 – 7.32 (m, 2H), 7.31 – 7.27 (m, 1H), 6.85 (d, *J* = 8.1 Hz, 1H), 6.81 – 6.75 (m, 2H), 6.61 (s, 1H), 6.58 (s, 1H), 5.14 – 5.11 (m, 2H), 4.04 (dd, *J* = 9.2, 4.4 Hz, 1H), 3.88 (s, 3H), 3.85 (s, 3H), 3.82 (s, 3H), 3.21 – 3.05 (m, 2H), 2.88 – 2.61 (m, 4H), 1.62 (s, br, 1H).
^{13}C NMR (101 MHz, $CDCl_3$) δ 148.6, 148.1, 147.6, 147.1, 137.3, 131.5, 130.7, 128.6(2C), 127.9, 127.6, 127.4(2C), 122.3, 115.6, 112.2, 112.0, 109.5, 71.1, 57.0, 56.2, 56.1, 56.0, 42.2, 41.0, 29.7.
IR (film) ν_{max}/cm^{-1} 2950, 2833, 1609, 1589, 1513, 1260.
HRMS (ESI$^+$) $C_{26}H_{29}NO_4$ found [M+H]$^+$ at 420.2164, expected at 420.2169.
$[\alpha]_D^{25}$ –21.8 (c. 0.99, $CHCl_3$).

(*R*)-1-(3-(Benzyloxy)-4-methoxybenzyl)-6,7-dimethoxy-1,2,3,4-tetrahydroisoquinoline ((*R*)-8b)

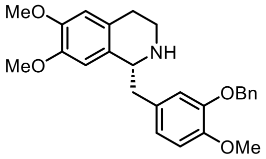

2-(3-(Benzyloxy)-4-methoxyphenyl)-*N*-(3,4-dimethoxyphenethyl)acetamide **7a** (1.0 g, 2.3 mmol), 2-chloropyridine (0.26 mL, 2.8 mmol) and trifluoromethanesulfonic anhydride (0.43 mL, 2.5 mmol) in anhydrous CH_2Cl_2 (12 mL) were submitted to **General Procedure B**.

The crude imine, RuCl[(*S,S*)-TsDPEN](*p*-cymene) (0.10 g, 0.16 mmol), HCO_2H/Et_3N (5:2 v/v, 1.2 mL) in anhydrous DMF (12 mL) were submitted to **General Procedure D**. Purification by flash column chromatography (EtOAc/pentane/7N ammonia solution in MeOH, 7:1:0.1 → 1:0:0.1) afforded the title compound as a yellow oil (0.86 g, 2.1 mmol, 89% over 2 steps, 96% *ee* after Boc derivatisation).

^{1}H NMR (500 MHz, $CDCl_3$) δ 7.46 – 7.39 (m, 2H, H16), 7.38 – 7.32 (m, 2H, H17), 7.31 – 7.27 (m, 1H, H18), 6.85 (d, *J* = 8.1 Hz, 1H, H13), 6.83 – 6.74 (m, 2H, H10, H14), 6.61 (s, 1H, H5), 6.58 (s, 1H, H8), 5.13 (d, *J* = 3.9 Hz, 2H, H15), 4.04 (dt, *J* = 13.0, 6.6 Hz, 1H, H1), 3.88 (s, 3H, H19), 3.85 (s, 3H, H21), 3.81 (s, 3H, H20), 3.16 – 3.06 (m, 2H, $H3_A$, $H\alpha_A$), 2.87 – 2.69 (m, 4H, $H3_B$, $H\alpha_B$, H4), 1.93 (s, br, 1H, H2).
^{13}C NMR (126 MHz, $CDCl_3$) δ 148.5, 148.1, 147.5, 147.1, 137.2, 131.4, 130.5, 128.6(2C), 127.9, 127.5, 127.4(2C), 122.3, 115.5, 112.1, 111.9, 109.5, 71.1, 56.9, 56.2, 56.1, 55.9, 42.1, 41.0, 29.6.
IR (film) ν_{max}/cm^{-1} 2933, 2834, 1609, 1589, 1513, 1262.
HRMS (ESI$^+$) $C_{26}H_{29}NO_4$ found [M+H]$^+$ at 420.2162, expected at 420.2169.
$[\alpha]_D^{25}$ +23.6 (c. 1.29, $CHCl_3$).

1-(3-Bromo-4-((4-methoxybenzyl)oxy)benzyl)-6,7-dimethoxy-1,2,3,4-tetrahydroisoquinoline (rac-8c)

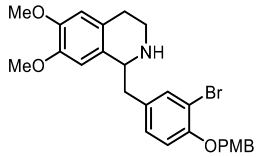

2-(3-Bromo-4-((4-methoxybenzyl)oxy)phenyl)-*N*-(3,4-dimethoxyphenethyl)acetamide **7b** (0.10 g, 0.19 mmol), 2-chloropyridine (22 μL, 0.23 mmol) and trifluoromethanesulfonic anhydride (35 μL, 0.21 mmol) in anhydrous CH_2Cl_2 (1 mL) were submitted to **General Procedure B**.
The crude imine and sodium borohydride (14 mg, 0.38 mmol) in EtOH (1 mL) were submitted to **General Procedure C**. Purification by flash column chromatography (EtOAc/pentane/7N ammonia solution in MeOH, 7:1:0.1 → 1:0:0.1) afforded the title compound as a yellow oil (70 mg, 0.14 mmol, 74%).

^{1}H NMR (500 MHz, $CDCl_3$) δ 7.47 (d, J = 2.2 Hz, 1H), 7.39 (d, J = 8.6 Hz, 2H), 7.10 (dd, J = 8.3, 2.2 Hz, 1H), 6.91 (d, J = 8.6 Hz, 2H), 6.89 (d, J = 8.3 Hz, 1H), 6.59 (2s, 2H), 5.06 (s, 2H), 4.09 (dd, J = 9.5, 4.3 Hz, 1H), 3.85 (s, 3H), 3.81 (2s, 6H), 3.19 (dt, J = 11.7, 5.6 Hz, 1H), 3.11 (dd, J = 13.8, 4.3 Hz, 1H), 2.95 – 2.88 (m, 1H), 2.85 – 2.64 (m, 3H), 2.03 (s, br, 1H).
^{13}C NMR (126 MHz, $CDCl_3$) δ 159.5, 153.8, 147.6, 147.1, 134.1, 133.2, 130.2, 129.3, 128.8(2C), 128.7, 127.4, 114.2, 114.0(2C), 112.7, 111.9, 109.4, 70.9, 56.9, 56.0, 55.9, 55.3, 41.7, 40.7, 29.5.
IR (film) ν_{max}/cm^{-1} 2952, 2935, 2835, 1612, 1587, 1515, 1494, 1464, 1247, 1175, 730.
HRMS (ESI$^+$) $C_{26}H_{28}Br^{79}NO_4$ found $[M+H]^+$ at 498.1262, expected at 498.1275.

(*S*)-1-(3-Bromo-4-((4-methoxybenzyl)oxy)benzyl)-6,7-dimethoxy-1,2,3,4-tetrahydroisoquinoline ((*S*)-8c)

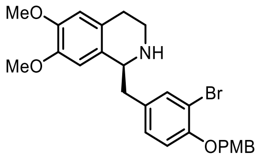

2-(3-Bromo-4-((4-methoxybenzyl)oxy)phenyl)-N-(3,4-dimethoxyphenethyl)acetamide **7b** (1.00 g, 1.94 mmol), 2-chloropyridine (220 μL, 2.33 mmol) and trifluoromethanesulfonic anhydride (360 μL, 2.14 mmol) in anhydrous CH_2Cl_2 (10 mL) were submitted to **General Procedure B**.
The crude imine, RuCl[(*R,R*)-TsDPEN](*p*-cymene) (87 mg, 0.14 mmol) and HCO_2H/Et_3N (5:2 v/v, 1.00 mL) in anhydrous DMF (10 mL) were submitted to **General Procedure D**. Purification by flash column chromatography (EtOAc/pentane/7N ammonia solution in MeOH, 7:1:0.1 → 1:0:0.1) afforded the title compound as an amber oil (0.868 g, 1.74 mmol, 90% over 2 steps, 96% *ee* after Boc derivatisation).

^{1}H NMR (500 MHz, $CDCl_3$) δ 7.48 (d, J = 2.1 Hz, 1H, H10), 7.40 (d, J = 8.5 Hz, 2H, H16), 7.11 (dd, J = 8.3, 2.1 Hz, 1H, H14), 6.92 (d, J = 8.5 Hz, 2H, H17), 6.90 (d, J = 8.3 Hz, 1H, H13), 6.60 (2s, 2H, H5, H8), 5.08 (s, 2H, H15), 4.09 (dd, J = 9.7, 4.3 Hz, 1H, H1), 3.86 (s, 3H, H20), 3.82 (2s, 6H, H18, H19), 3.19 (dt, J = 12.0, 5.4 Hz, 1H, $H3_A$), 3.12 (dd, J = 13.8, 4.3 Hz, 1H, $H\alpha_A$), 2.93 (ddd, J = 12.0, 7.0, 5.4 Hz, 1H, $H3_B$), 2.85 – 2.64 (m, 3H, $H\alpha_B$, H4), 1.72 (s, br, 1H, H2).
^{13}C NMR (126 MHz, $CDCl_3$) δ 159.6, 153.9, 147.7, 147.2, 134.2, 133.3, 130.4, 129.4, 128.9, 128.8(2C), 127.5, 114.3, 114.1(2C), 112.8, 112.0, 109.5, 71.0, 57.0, 56.1, 56.0, 55.4, 41.8, 40.9, 29.6.
IR (film) ν_{max}/cm^{-1} 2934, 2834, 1612, 1587, 1515, 1494, 1464, 1247, 1175.
HRMS (ESI$^+$) $C_{26}H_{28}Br^{79}NO_4$ found $[M+H]^+$ at 498.1266, expected at 498.1275.
$[\alpha]_D^{25}$ –3.8 (c. 0.86, $CHCl_3$).

(*R*)-1-(3-Bromo-4-((4-methoxybenzyl)oxy)benzyl)-6,7-dimethoxy-1,2,3,4-tetrahydroisoquinoline ((*R*)-8c)

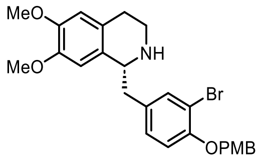

2-(3-Bromo-4-((4-methoxybenzyl)oxy)phenyl)-*N*-(3,4-dimethoxyphenethyl)acetamide **7b** (1.00 g, 1.94 mmol), 2-chloropyridine (220 µL, 2.33 mmol) and trifluoromethanesulfonic anhydride (360 µL, 2.14 mmol) in anhydrous CH_2Cl_2 (10 mL) were submitted to **General Procedure B**.
The crude imine, RuCl[(*S,S*)-TsDPEN](*p*-cymene) (87 mg, 0.14 mmol) and HCO_2H/Et_3N (5:2 v/v, 1.00 mL) in anhydrous DMF (10 mL) were submitted to **General Procedure D**. Purification by flash column chromatography (EtOAc/pentane/7N ammonia solution in MeOH, 7:1:0.1 → 1:0:0.1) afforded the title compound as an amber oil (638 mg, 1.28 mmol, 66% over 2 steps, 92% *ee* after Boc derivatisation).

^{1}H NMR (400 MHz, $CDCl_3$) δ 7.48 (d, *J* = 2.1 Hz, 1H), 7.40 (d, *J* = 8.7 Hz, 2H), 7.11 (dd, *J* = 8.3, 2.1 Hz, 1H), 6.97 – 6.86 (m, 3H), 6.60 (2s, 2H), 5.08 (s, 2H), 4.09 (dd, *J* = 9.6, 4.3 Hz, 1H), 3.86 (s, 3H), 3.82 (2s, 6H), δ 3.19 (dt, *J* = 11.7, 5.6 Hz, 1H), 3.11 (dd, *J* = 14.0, 4.3 Hz, 1H), 2.98 – 2.86 (m, 1H), 2.86 – 2.63 (m, 3H), 1.70 (s, br, 1H).
^{13}C NMR (126 MHz, $CDCl_3$) δ 159.6, 153.9, 147.7, 147.2, 134.2, 133.3, 130.4, 129.4, 128.9(2C), 128.8, 127.5, 114.3, 114.1(2C), 112.8, 112.0, 109.5, 71.0, 57.0, 56.1, 56.0, 55.4, 41.8, 40.8, 29.6.
IR (film) ν_{max}/cm^{-1} 2934, 2834, 1612, 1587, 1515, 1494, 1464, 1248, 1175.
HRMS (ESI$^+$) $C_{26}H_{28}Br^{79}NO_4$ found $[M+H]^+$ at 512.1426, expected at 512.1431.
$[\alpha]_D^{25}$ +3.6 (c. 1.28, $CHCl_3$).

1-(4-(Benzyloxy)benzyl)-6,7-dimethoxy-1,2,3,4-tetrahydroisoquinoline (rac-8d)

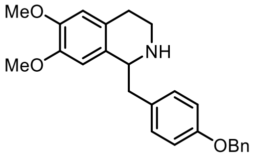

2-(4-(Benzyloxy)phenyl)-*N*-(3,4-dimethoxyphenethyl)acetamide **7c** (0.10 g, 0.25 mmol), 2-chloropyridine (28 µL, 0.30 mmol) and trifluoromethanesulfonic anhydride (46 µL, 0.27 mmol) in anhydrous CH_2Cl_2 (1.2 mL) were submitted to **General Procedure B**.
The crude imine and sodium borohydride (18.6 mg, 0.493 mmol) in EtOH (1.2 mL) were submitted to **General Procedure C**. Purification by flash column chromatography (EtOAc/pentane/7N ammonia solution in MeOH, 7:1:0.1 → 1:0:0.1) afforded the title compound as a yellow oil (24 mg, 61 µmol, 25%).

^{1}H NMR (500 MHz, $CDCl_3$) δ 7.44 (d, *J* = 7.0 Hz, 2H), 7.42 – 7.36 (m, 2H), 7.36 – 7.30 (m, 1H), 7.16 (d, *J* = 8.6 Hz, 2H), 6.94 (d, *J* = 8.6 Hz, 2H), 6.60 (s, 1H), 6.59 (s, 1H), 5.06 (s, 2H), 4.10 (dd, *J* = 9.5, 4.5 Hz, 1H), 3.86 (s, 3H), 3.80 (s, 3H), 3.24 – 3.17 (m, 1H), 3.14 (dd, *J* = 13.8, 4.5 Hz, 1H), 2.97 – 2.83 (m, 2H), 2.80 – 2.65 (m, 2H), 2.17 (s, br, 1H).
^{13}C NMR (126 MHz, $CDCl_3$) δ 157.7, 147.6, 147.1, 137.2, 131.4, 130.5(2), 130.4, 128.7(2C), 128.1, 127.6(2C), 127.3, 115.1(2C), 111.9, 109.6, 70.2, 57.1, 56.1, 56.0, 41.9, 40.8, 29.5.
Spectroscopic data is in agreement with that reported previously.[25]

(*S*)-1-(4-(Benzyloxy)benzyl)-6,7-dimethoxy-1,2,3,4-tetrahydroisoquinoline ((*S*)-8d)

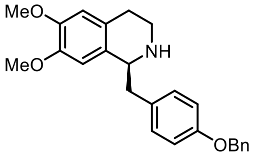

2-(4-(Benzyloxy)phenyl)-*N*-(3,4-dimethoxyphenethyl)acetamide **7c** (500 mg, 1.23 mmol), 2-chloropyridine (140 µL, 1.48 mmol) and trifluoromethanesulfonic anhydride (230 µL, 1.36 mmol) in anhydrous CH_2Cl_2 (6.0 mL) were submitted to **General Procedure B**.
The crude imine, RuCl[(*R,R*)-TsDPEN](*p*-cymene) (55 mg, 86 µmol) and HCO_2H/Et_3N (5:2 v/v, 0.60 mL) in anhydrous DMF (6.0 mL) were submitted to **General Procedure D**. Purification by flash column chromatography (EtOAc/pentane/7N ammonia solution in MeOH, 7:1:0.1 → 1:0:0.1) afforded the title compound as an amber oil (444 mg, 1.14 mmol, 93% over 2 steps, 96% *ee* after Boc derivatisation).

^{1}H NMR (500 MHz, $CDCl_3$) δ 7.44 (d, *J* = 7.0 Hz, 2H), 7.42 – 7.36 (m, 2H), 7.36 – 7.30 (m, 1H), 7.17 (d, *J* = 8.6 Hz, 2H), 6.95 (d, *J* = 8.6 Hz, 2H), 6.63 (s, 1H), 6.59 (s, 1H), 5.06 (s, 2H), 4.10 (dd, *J* = 9.5, 4.4 Hz, 1H), 3.86 (s, 3H), 3.81 (s, 3H), 3.23 – 3.17 (m, 1H), 3.15 (dd, *J* = 13.7, 4.4 Hz, 1H), 2.97 – 2.82 (m, 2H), 2.80 – 2.65 (m, 2H), 1.69 (s, br, 1H).
^{13}C NMR (126 MHz, $CDCl_3$) δ 157.6, 147.5, 147.1, 137.2, 131.5, 130.8, 130.5(2C), 128.7(2C), 128.1, 127.6(2C), 127.5, 115.1(2C), 112.0, 109.6, 70.2, 57.1, 56.1, 56.0, 42.0, 40.9, 29.7.
$[\alpha]_D^{25}$ +6.3 (c. 0.63, $CHCl_3$).
Spectroscopic data is in agreement with that reported previously.[25]
Note: Spectroscopic data is reported for **rac-8d**.

(*R*)-1-(4-(Benzyloxy)benzyl)-6,7-dimethoxy-1,2,3,4-tetrahydroisoquinoline ((*R*)-8d)

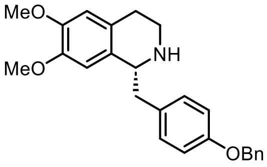

2-(4-(Benzyloxy)phenyl)-*N*-(3,4-dimethoxyphenethyl)acetamide **7c** (500 mg, 1.23 mmol), 2-chloropyridine (140 µL, 1.48 mmol) and trifluoromethanesulfonic anhydride (230 µL, 1.36 mmol) in anhydrous CH_2Cl_2 (6.0 mL) were submitted to **General Procedure B**.
The crude imine, RuCl[(*S,S*)-TsDPEN](*p*-cymene) (55 mg, 86 µmol) and HCO_2H/Et_3N (5:2 v/v, 0.60 mL) in anhydrous DMF (6.0 mL) were submitted to **General Procedure D**. Purification by flash column chromatography (EtOAc/pentane/7N ammonia solution in MeOH, 7:1:0.1 → 1:0:0.1) afforded the title compound as an amber oil (454 mg, 1.16 mmol, 94% over 2 steps, 94% *ee* after Boc derivatisation).

^{1}H NMR (500 MHz, $CDCl_3$) δ 7.47 – 7.41 (m, 2H), 7.41 – 7.35 (m, 2H), 7.35 – 7.30 (m, 1H), 7.17 (d, *J* = 8.6 Hz, 2H), 6.95 (d, *J* = 8.6 Hz, 2H), 6.63 (s, 1H), 6.59 (s, 1H), 5.06 (s, 2H), 4.10 (dd, *J* = 9.4, 4.4 Hz, 1H), 3.86 (s, 3H), 3.81 (s, 3H), 3.25 – 3.18 (m, 1H), 3.15 (dd, *J* = 13.8, 4.4 Hz, 1H), 2.97 – 2.81 (m, 2H), 2.80 – 2.65 (m, 2H), 1.66 (s, br, 1H).
^{13}C NMR (126 MHz, $CDCl_3$) δ 157.6, 147.6, 147.1, 137.3, 131.6, 130.8, 130.5(2C), 128.7(2C), 128.1, 127.6(2C), 127.5, 115.1(2C), 112.0, 109.6, 70.2, 57.1, 56.1, 56.0, 42.0, 40.9, 29.7.
$[\alpha]_D^{25}$ –6.9 (c. 0.49, $CHCl_3$).
Spectroscopic data is in agreement with that reported previously.[25]

(*S*)-1-(4-Bromobenzyl)-6,7-dimethoxy-2-methyl-1,2,3,4-tetrahydroisoquinoline ((*S*)-9a)

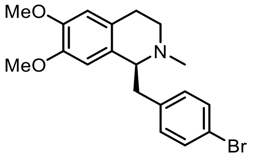

(*S*)-1-(4-Bromobenzyl)-6,7-dimethoxy-1,2,3,4-tetrahydroisoquinoline **(*S*)-8a** (0.10 g, 0.29 mmol), formalin (37% aq., 0.60 mL, 8.1 mmol) and sodium borohydride (33 mg, 0.87 mmol) in MeOH (1.9 mL) were submitted to **General Procedure E**. Purification by flash column chromatography (EtOAc/MeOH, 98:2) afforded the title compound as a pale-yellow oil (84 mg, 0.23 mmol, 79%).

^{1}H NMR (400 MHz, $CDCl_3$) δ 7.34 (d, *J* = 8.3 Hz, 2H), 6.95 (d, *J* = 8.3 Hz, 2H), 6.54 (s, 1H), 6.06 (s, 1H), 3.83 (s, 3H), 3.68 (dd, *J* = 7.2, 5.4 Hz, 1H), 3.61 (s, 3H), 3.21 – 3.04 (m, 2H), 2.85 – 2.68 (m, 3H), 2.57 – 2.51 (m, 1H), 2.50 (s, 3H).
^{13}C NMR (101 MHz, $CDCl_3$) δ 147.4, 146.5, 139.0, 131.7(2C), 131.1(2C), 128.9, 126.3, 119.8, 111.3, 110.9, 64.7, 55.8, 55.6, 47.0, 42.8, 40.6, 25.6.
$[\alpha]_D^{25}$ +49.5 (c. 1.06, $CHCl_3$).
Spectroscopic data is in agreement with that reported previously.[21]

(*R*)-1-(4-Bromobenzyl)-6,7-dimethoxy-2-methyl-1,2,3,4-tetrahydroisoquinoline ((*R*)-9a)

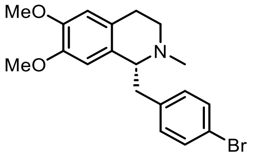

(*R*)-1-(4-Bromobenzyl)-6,7-dimethoxy-1,2,3,4-tetrahydroisoquinoline **(*R*)-8a** (494 mg, 1.36 mmol), formalin (37% aq., 2.80 mL, 37.6 mmol) and sodium borohydride (155 mg, 4.08 mmol) in MeOH (13.6 mL) were submitted to **General Procedure E**. Purification by flash column chromatography (EtOAc/MeOH, 98:2) afforded the title compound as a pale-yellow oil (325 mg, 0.884 mmol, 65%).

^{1}H NMR (500 MHz, $CDCl_3$) δ 7.35 (d, *J* = 8.3 Hz, 2H, H11), 6.96 (d, *J* = 8.3 Hz, 2H, H10), 6.54 (s, 1H, H5), 6.06 (s, 1H, H8), 3.83 (s, 3H, H16), 3.68 (dd, *J* = 7.3, 5.4 Hz, 1H, H1), 3.61 (s, 3H, H15), 3.20 – 3.05 (m, 2H, $H3_A$, $H\alpha_A$), 2.85 – 2.69 (m, 3H, $H\alpha_B$, $H4_A$, $H3_B$), 2.57 – 2.51 (m, 1H, $H4_B$), 2.50 (s, 3H, H2).
^{13}C NMR (126 MHz, $CDCl_3$) δ 147.4, 146.5, 139.0, 131.7(2C), 131.1(2C), 128.9, 126.4, 119.8, 111.3, 110.9, 64.7, 55.8, 55.7, 47.0, 42.8, 40.6, 25.6.
IR (film) ν_{max}/cm^{-1} 2933, 2832, 2794, 1610, 1514, 1487.
HRMS (ESI^+) $C_{19}H_{22}Br^{79}NO_2$ found $[M+H]^+$ at 376.0903, expected at 376.0907.
$[\alpha]_D^{25}$ –50.6 (c. 1.01, $CHCl_3$).

(*S*)-1-(3-(Benzyloxy)-4-methoxybenzyl)-6,7-dimethoxy-2-methyl-1,2,3,4-tetrahydroisoquinoline ((*S*)-9b), (+)-*O*-benzyllaudanidine

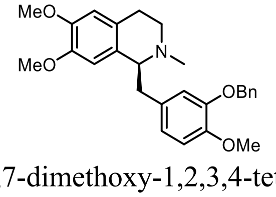

(*S*)-1-(3-(Benzyloxy)-4-methoxybenzyl)-6,7-dimethoxy-1,2,3,4-tetrahydroisoquinoline **(*S*)-8b** (0.20 g, 0.48 mmol), formalin (37% aq., 1.0 mL, 13 mmol) and sodium borohydride (55 mg, 1.4 mmol) in MeOH (9.6 mL) were submitted to **General Procedure E**. Purification by flash column chromatography (EtOAc/MeOH, 98:2) afforded the title compound as a yellow oil (0.18 g, 0.40 mmol, 84%).

^{1}H NMR (500 MHz, $CDCl_3$) δ 7.46 – 7.39 (m, 2H), 7.34 (dd, *J* = 8.5, 6.8 Hz, 2H), 7.31 – 7.24 (m, 1H), 6.77 (d, *J* = 8.1 Hz, 1H), 6.67 (d, *J* = 2.1 Hz, 1H), 6.61 (dd, *J* = 8.1, 2.1 Hz, 1H), 6.54 (s, 1H), 6.01 (s, 1H), 5.07 (d, *J* = 1.6 Hz, 2H), 3.85 (s, 3H), 3.83 (s, 3H), 3.63 – 3.58 (m, 1H), 3.57 (s, 3H), 3.16 – 3.03 (m, 2H), 2.85 – 2.67 (m, 3H), 2.59 – 2.50 (m, 1H), 2.48 (s, 3H).
^{13}C NMR (126 MHz, $CDCl_3$) δ 148.2, 147.9, 147.3, 146.4, 137.4, 132.6, 129.4, 128.6(2C), 127.9, 127.4(2C), 126.2, 122.7, 116.0, 111.7, 111.2, 111.1, 71.1, 64.9, 56.2, 55.9, 55.7, 47.1, 42.8, 40.8, 25.7.
IR (film) ν_{max}/cm^{-1} 2934, 2833, 1608, 1588, 1514.
HRMS (ESI$^+$) $C_{27}H_{31}NO_4$ found $[M+H]^+$ at 434.2331, expected at 434.2326.
$[\alpha]_D^{25}$ +71.1 (c. 1.19, $CHCl_3$).
Spectroscopic data is in agreement with that reported previously.[28]
Note: Spectroscopic data is reported for **rac-9b**.

(*R*)-1-(3-(Benzyloxy)-4-methoxybenzyl)-6,7-dimethoxy-2-methyl-1,2,3,4-tetrahydroisoquinoline ((*R*)-9b), (−)-*O*-benzyllaudanidine

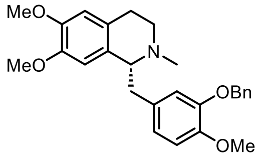

(*R*)-1-(3-(Benzyloxy)-4-methoxybenzyl)-6,7-dimethoxy-1,2,3,4-tetrahydroisoquinoline **(*R*)-8b** (647 mg, 1.54 mmol), formalin (37% aq., 2.30 mL, 30.1 mmol) and sodium borohydride (175 mg, 4.62 mmol) in MeOH (16 mL) were submitted to **General Procedure E**. Purification by flash column chromatography (EtOAc/MeOH, 98:2) afforded the title compound as a yellow oil (0.430 g, 0.990 mmol, 65%).

^{1}H NMR (400 MHz, $CDCl_3$) δ 7.44 – 7.39 (m, 2H), 7.38 – 7.31 (m, 2H), 7.30 – 7.23 (m, 1H), 6.77 (d, *J* = 8.2 Hz, 1H), 6.67 (d, *J* = 2.0 Hz, 1H) 6.61 (dd, *J* = 8.1, 2.0 Hz, 1H), 6.54 (s, 1H), 6.01 (s, 1H), 5.07 (s, 2H), 3.85 (s, 3H), 3.83 (s, 3H), 3.64 – 3.56 (m, 1H), 3.57 (s, 3H), 3.17 – 3.02 (m, 2H), 2.86 – 2.66 (m, 3H), 2.58 – 2.54 (m, 1H,), 2.49 (s, 3H).
^{13}C NMR (101 MHz, $CDCl_3$) δ 148.1, 147.8, 147.3, 146.4, 137.4, 132.5, 129.4, 128.6(2C), 127.8, 127.4(2C), 126.1, 122.7, 116.0, 111.7, 111.2, 111.0, 71.1, 64.9, 56.2, 55.8, 55.6, 47.1, 42.8, 40.8, 25.7.
IR (film) ν_{max}/cm^{-1} 2937, 2833, 1610, 1589, 1513.
HRMS (ESI$^+$) $C_{27}H_{31}NO_4$ found $[M+H]^+$ at 434.2325, expected at 434.2326.
$[\alpha]_D^{25}$ −74.6 (c. 1.33, $CHCl_3$).
Spectroscopic data is in agreement with that reported previously.[28]
Note: Spectroscopic data is reported for **rac-9b**.

(*S*)-5-((6,7-Dimethoxy-2-methyl-1,2,3,4-tetrahydroisoquinolin-1-yl)methyl)-2-methoxyphenol ((*S*)-10a), (+)-laudanidine

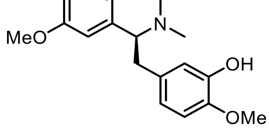

To an oven-dried flask containing Pd/C (10% wt., 21 mg, 20 µmol, 0.04 equiv.) under an inert atmosphere, was added anhydrous MeOH (0.2 mL) and the flask was evacuated and backfilled with hydrogen (×3). (*S*)-1-(3-(Benzyloxy)-4-methoxybenzyl)-6,7-dimethoxy-2-methyl-1,2,3,4-tetrahydro-isoquinoline **(*S*)-9b** (210 mg, 0.484 mmol, 1.0 equiv.) was then added as a solution in anhydrous MeOH (4.60 mL) and AcOH (60.0 µL, 0.107 mmol, 2.20 equiv.). The resulting mixture was stirred under a hydrogen atmosphere for 24 h. The reaction mixture was filtered through a pad of Celite®, the filter cake was washed with CH_2Cl_2/MeOH (9:1, 20 mL)

and the filtrate was concentrated *in vacuo*. The residue was then diluted with CH_2Cl_2 (5 mL) and washed with $NaHCO_3$ (aq., sat., to pH 7) and the aqueous phase was extracted with CH_2Cl_2 (3 × 5 mL). The combined organic extracts were then washed with brine (7 mL), dried ($MgSO_4$), filtered and concentrated *in vacuo*. The title compound was collected as a white solid (116 mg, 0.34 mmol, 71%).

^{1}H NMR (500 MHz, $CDCl_3$) δ 6.77 (d, *J* = 2.1 Hz, 1H), 6.71 (d, *J* = 8.1 Hz, 1H), 6.55 (s, 1H), 6.51 (dd, *J* = 8.1, 2.1 Hz, 1H), 6.04 (s, 1H), 3.84 (s, 3H), 3.83 (s, 3H), 3.71 (dd, *J* = 8.1, 5.1 Hz, 1H), 3.55 (s, 3H), 3.23 – 3.19 (m, 1H), 3.13 (dd, *J* = 13.7, 7.0 Hz, 1H), 2.91 – 2.75 (m, 2H), 2.70 (dd, *J* = 13.7, 7.0 Hz, 1H), 2.63 – 2.59 (m, 1H), 2.52 (s, 3H).
Note: O*H* resonance was not observed.
^{13}C NMR (126 MHz, $CDCl_3$) δ 147.4, 146.4, 145.7, 145.3, 133.2, 129.2, 125.6, 121.3, 116.0, 111.2, 111.1, 110.6, 64.9, 56.1, 55.8, 55.6, 46.6, 42.5, 40.9, 25.1.
HRMS (ESI$^+$) $C_{20}H_{25}NO_4$ found $[M+H]^+$ at 344.1850, expected at 344.1856.
$[\alpha]_D^{25}$ +94.4 (c. 1.18, $CHCl_3$). Ref [21] $[\alpha]_D^{25}$ +83.7 (c. 1.0, $CHCl_3$).
Spectroscopic data is in agreement with that reported previously.[21]

(*R*)-5-((6,7-Dimethoxy-2-methyl-1,2,3,4-tetrahydroisoquinolin-1-yl)methyl)-2-methoxyphenol ((*R*)-10a), (–)-laudanidine

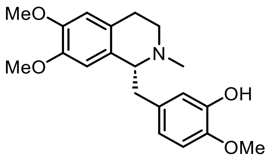

To an oven-dried flask containing Pd/C (10% wt., 36 mg, 34 μmol, 0.04 equiv.) under an inert atmosphere, was added anhydrous MeOH (1.0 mL) and the flask was evacuated and backfilled with hydrogen (× 3). (*S*)-1-(3-(benzyloxy)-4-methoxybenzyl)-6,7-dimethoxy-2-methyl-1,2,3,4-tetrahydro-isoquinoline **(*R*)-9b** (0.36 g, 0.83 mmol, 1.0 equiv.) was then added as a solution in anhydrous MeOH (5.5 mL) and AcOH (0.11 mL, 1.8 mmol, 2.2 equiv.). The resulting mixture was stirred under a hydrogen atmosphere for 24 h. The reaction mixture was filtered through a pad of Celite®, the filter cake was washed with CH_2Cl_2/MeOH (9:1, 20 mL) and the filtrate was concentrated *in vacuo*. The residue was then diluted with CH_2Cl_2 (5 mL) and washed with $NaHCO_3$ (aq., sat., to pH 7) and the aqueous phase was extracted with CH_2Cl_2 (3 × 5 mL). The combined organic extracts were then washed with brine (7 mL), dried (MgSO4), filtered and concentrated *in vacuo*. The title compound was collected as a white solid (0.20 g, 0.59 mmol, 71%).

^{1}H NMR (500 MHz, $CDCl_3$) δ 6.76 (d, *J* = 2.1 Hz, 1H), 6.71 (d, *J* = 8.2 Hz, 1H), 6.53 (s, 1H), 6.50 (dd, *J* = 8.2, 2.1 Hz, 1H), 6.03 (s, 1H), 3.84 (s, 3H), 3.83 (s, 3H), 3.72 – 3.68 (m, 1H), 3.54 (s, 3H), 3.25 – 3.10 (m, 2H), 2.90 – 2.75 (m, 2H), 2.70 (dd, *J* = 13.6, 8.1 Hz, 1H), 2.65 – 2.57 (m, 1H), 2.51 (s, 3H).
Note: O*H* resonance was not observed.
^{13}C NMR (126 MHz, $CDCl_3$) δ 147.4, 146.4, 145.7, 145.3, 133.1, 129.1, 125.5, 121.3, 116.0, 111.2, 111.1, 110.6, 64.9, 56.0, 55.8, 55.5, 46.5, 42.4, 40.9, 25.0.
HRMS (ESI$^+$) $C_{20}H_{25}NO_4$ found $[M+H]^+$ at 344.1854, expected at 344.1856.
$[\alpha]_D^{25}$ –93.5 (c. 1.18, $CHCl_3$). Ref [29] $[\alpha]_D^{25}$ –94.7 (c. 0.4, $CHCl_3$).
Spectroscopic data is in agreement with that reported previously for **(*S*)-10b**.[21]

(*S*)-1-(3-Bromo-4-((4-methoxybenzyl)oxy)benzyl)-6,7-dimethoxy-2-methyl-1,2,3,4-tetrahydroisoquinoline ((*S*)-9c)

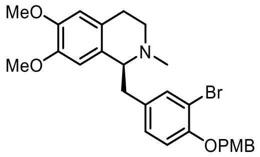

(*S*)-1-(3-Bromo-4-((4-methoxybenzyl)oxy)benzyl)-6,7-dimethoxy-1,2,3,4-tetrahydroisoquinoline **(*S*)-8c** (824 mg, 1.65 mmol), formalin (37% aq., 3.40 mL, 45.7 mmol) and sodium borohydride (187 mg, 4.95 mmol) in MeOH (33 mL) were submitted to **General Procedure E**. Purification by flash column chromatography (EtOAc/7N ammonia solution in MeOH, 99:1) afforded the title compounds as an amber foam (692 mg, 1.35 mmol, 82%).

^{1}H NMR (500 MHz, $CDCl_3$) δ 7.38 (d, *J* = 8.7 Hz, 2H), 7.34 (d, *J* = 2.1 Hz, 1H), 6.93 – 6.89 (m, 3H), 6.81 (d, *J* = 8.4 Hz, 1H), 6.55 (s, 1H), 6.08 (s, 1H), 5.05 (s, 2H), 3.84 (s, 3H), 3.81 (s, 3H), 3.64 (dd, *J* = 7.3, 5.3 Hz, 1H), 3.58 (s, 3H), 3.19 – 3.12 (m, 1H), 3.07 (dd, *J* = 13.8, 5.3 Hz, 1H), 2.84 – 2.70 (m, 3H), 2.56 (dt, *J* = 15.6, 4.6 Hz, 1H), 2.52 (s, 3H).
^{13}C NMR (126 MHz, $CDCl_3$) δ 159.5, 153.5, 147.7, 146.6, 134.5, 134.3, 129.8, 129.2, 128.8(3C), 126.2, 114.1(2C), 114.0, 112.3, 111.4, 111.0, 70.9, 64.9, 55.9, 55.7, 55.4, 47.0, 42.8, 40.3, 25.6.
IR (film) ν_{max}/cm^{-1} 3002, 2936, 2834, 1612, 1515, 1494, 1464, 1228, 761.
HRMS (ESI^{+}) $C_{27}H_{30}Br^{79}NO_4$ found $[M+H]^+$ at 512.1420, expected at 512.1431.
$[\alpha]_D^{25}$ +44.1 (c. 1.39, $CHCl_3$).

(*R*)-1-(3-Bromo-4-((4-methoxybenzyl)oxy)benzyl)-6,7-dimethoxy-2-methyl-1,2,3,4-tetrahydroisoquinoline ((*R*)-9c)

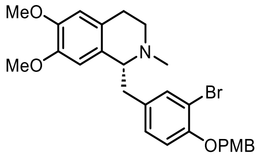

(*R*)-1-(3-Bromo-4-((4-methoxybenzyl)oxy)benzyl)-6,7-dimethoxy-1,2,3,4-tetrahydroisoquinoline **(*R*)-8c** (544 mg, 1.09 mmol), formalin (37% aq., 2.25 mL, 30.2 mmol) and sodium borohydride (124 mg, 3.27 mmol) in MeOH (22 mL) were submitted to **General Procedure E**. Purification by flash column chromatography (EtOAc/7N ammonia solution in MeOH, 99:1) afforded the title compounds as an amber foam (444 mg, 0.866 mmol, 79%).

^{1}H NMR (500 MHz, $CDCl_3$ δ 7.38 (d, *J* = 8.7 Hz, 2H), 7.34 (d, *J* = 2.1 Hz, 1H), 6.99 – 6.89 (m, 3H), 6.81 (d, *J* = 8.4 Hz, 1H), 6.55 (s, 1H), 6.08 (s, 1H), 5.05 (s, 2H), 3.84 (s, 3H), 3.81 (s, 3H), 3.64 (dd, *J* = 7.2, 5.4 Hz, 1H), 3.58 (s, 3H), 3.20 – 3.11 (m, 1H), 3.06 (dd, *J* = 13.8, 5.4 Hz, 1H), 2.86 – 2.70 (m, 3H), 2.56 (dt, *J* = 15.7, 4.7 Hz, 1H), 2.51 (s, 3H).
^{13}C NMR (126 MHz, $CDCl_3$) δ 159.5, 153.5, 147.5, 146.6, 134.6, 134.3, 129.8, 129.2, 128.9(3C), 126.3, 114.1(2C), 114.0, 112.3, 111.4, 111.0, 71.0, 64.9, 55.9, 55.8, 55.4, 47.1, 42.8, 40.3, 25.6.
IR (film) ν_{max}/cm^{-1} 3002, 2936, 2834, 1612, 1515, 1494, 1464, 1228, 761.
HRMS (ESI^{+}) $C_{27}H_{30}Br^{79}NO_4$ found $[M+H]^+$ at 512.1421, expected at 512.1431.
$[\alpha]_D^{25}$ –43.6 (c. 0.80, $CHCl_3$).

S)-1-(4-(Benzyloxy)benzyl)-6,7-dimethoxy-2-methyl-1,2,3,4-tetrahydroisoquinoline ((S)-9d), (+)-O-benzylarmepavine

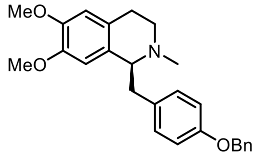

(*S*)-1-(4-(Benzyloxy)benzyl)-6,7-dimethoxy-1,2,3,4-tetrahydroisoquinoline **(*S*)-8d** (443 mg, 1.14 mmol), formalin (37% aq., 2.3 mL, 30.9 mmol) and sodium borohydride (130 mg, 3.42 mmol) in MeOH (23 mL) were submitted to **General Procedure E**. Purification by flash column chromatography (EtOAc/MeOH, 94:6) afforded the title compounds as an orange oil (358 mg, 0.887 mmol, 78%).

^{1}H NMR (500 MHz, $CDCl_3$) δ 7.45 – 7.40 (m, 2H), 7.41 – 7.35 (m, 2H), 7.35 – 7.29 (m, 1H), 7.01 (d, *J* = 8.7 Hz, 2H), 6.88 (d, *J* = 8.7 Hz, 2H), 6.56 (s, 1H), 6.01 (s, 1H), 5.04 (s, 2H), 3.84 (s, 3H), 3.67 (dd, *J* = 8.0, 5.0 Hz, 1H), 3.53 (s, 3H), 3.24 – 3.11 (m, 2H), 2.90 – 2.72 (m, 3H), 2.59 (dt, *J* = 15.9, 4.6 Hz, 1H), 2.54 (s, 3H).
^{13}C NMR (126 MHz, $CDCl_3$) δ 157.2, 147.3, 146.3, 137.3, 132.5, 130.9(2C), 129.4, 128.7(2C), 128.0, 127.5(2C), 126.0, 114.7(2C), 111.2, 111.1, 70.1, 65.0, 55.8, 55.6, 47.0, 42.8, 40.5, 25.6.
$[\alpha]_D^{25}$ +69.4 (c. 1.74, $CHCl_3$).
Spectroscopic data is in agreement with that reported previously.[28]
Note: Spectroscopic data is reported for **rac-9d**.

(R)-1-(4-(Benzyloxy)benzyl)-6,7-dimethoxy-2-methyl-1,2,3,4-tetrahydroisoquinoline ((R)-9d), (–)-O-benzylarmepavine

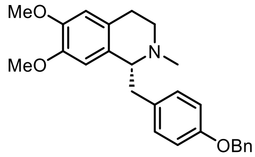

(*R*)-1-(4-(Benzyloxy)benzyl)-6,7-dimethoxy-1,2,3,4-tetrahydroisoquinoline **(*R*)-8d** (410 mg, 1.05 mmol), formalin (37% aq., 2.16 mL, 29.0 mmol) and sodium borohydride (120 mg, 3.15 mmol) in MeOH (20 mL) were submitted to **General Procedure E**. Purification by flash column chromatography (EtOAc/MeOH, 94:6) afforded the title compounds as an orange oil (337 mg, 0.835 mmol, 80%).

^{1}H NMR (500 MHz, $CDCl_3$) δ 7.45 – 7.41 (m, 2H), 7.40 – 7.34 (m, 2H), 7.34 – 7.29 (m, 1H), 7.03 (d, *J* = 8.7 Hz, 2H), 6.89 (d, *J* = 8.7 Hz, 2H), 6.57 (s, 1H), 6.03 (s, 1H), 5.04 (s, 2H), 3.84 (s, 3H), 3.69 (dd, *J* = 7.9, 5.0 Hz, 1H), 3.54 (s, 3H), 3.24 – 3.13 (m, 2H), 2.90 – 2.72 (m, 3H), 2.60 (dt, *J* = 15.9, 4.6 Hz, 1H), 2.55 (s, 3H).
^{13}C NMR (126 MHz, $CDCl_3$) δ 157.1, 147.2, 146.2, 137.2, 132.3, 130.7(2C), 129.3, 128.5(2C), 127.9, 127.4(2C), 125.9, 114.5(2C), 111.1, 111.0, 69.9, 64.9, 55.7, 55.4, 46.8, 42.7, 40.4, 25.5.
$[\alpha]_D^{25}$ –68.5 (c. 1.06, $CHCl_3$).
Spectroscopic data is in agreement with that reported previously.[28]
Note: Spectroscopic data is reported for **rac-9d**.

(*S*)-4-((6,7-Dimethoxy-2-methyl-1,2,3,4-tetrahydroisoquinolin-1-yl)methyl)phenol ((*S*)-10b), (+)-armepavine

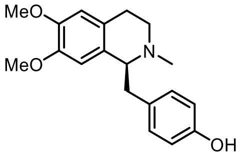

Method 1: (*S*)-1-(3-Bromo-4-((4-methoxybenzyl)oxy)benzyl)-6,7-dimethoxy-2-methyl-1,2,3,4-tetrahydro-isoquinoline **(*S*)-9c** (290 mg, 0.566 mmol), Pd/C (10% wt., 87.0 mg, 81.8 μmol, 0.14 equiv.) and triethylamine (790 μL, 5.66 mmol) in anhydrous MeOH (5.7 mL) were submitted to **General Procedure F** for 30 h. Upon completion, the reaction mixture was filtered through a pad of Celite®, the filter cake was washed with CH_2Cl_2/MeOH (8:2, 100 mL) and the filtrate was concentrated *in vacuo*. The residue was then diluted with $CHCl_3$, washed sequentially with HCl (aq., 1 M, 3 mL) and $NaHCO_3$ (aq., sat., to pH 7). The aqueous phase was extracted with $CHCl_3$:IPA (9:1, 3 × 15 mL). The combined organic extracts were then dried ($MgSO_4$), filtered and concentrated *in vacuo*. The title compound was collected as an off-white foam (160 mg, 0.511 mmol, 90%). No further purification was necessary.

Method 2: To an oven-dried flask containing Pd/C (10% wt., 33 mg, 31 μmol, 0.04 equiv.) under an inert atmosphere, was added anhydrous MeOH (1.0 mL) and the flask was evacuated and backfilled with hydrogen (×3). (*S*)-1-(4-(Benzyloxy)benzyl)-6,7-dimethoxy-2-methyl-1,2,3,4-tetrahydroiso-quinoline **(*S*)-9d** (0.33 g, 0.82 mmol, 1.0 equiv.) was then added as a solution in MeOH (7.0 mL) and AcOH (0.10 mL, 1.8 mmol, 2.2 equiv.). Using a gas bag, hydrogen was continuously bubbled through the reaction mixture by having the needle submerged in the reaction mixture and a second exit needle for 18 h. The gas bag was refilled as needed. Upon completion, the reaction mixture was filtered through a pad of Celite®, the filter cake was washed with CH_2Cl_2/MeOH (8:2, 60 mL) and the filtrate was concentrated *in vacuo*. The residue was then diluted with $CHCl_3$ and washed with $NaHCO_3$ (aq., sat., to pH 7). The aqueous phase was then extracted with $CHCl_3$:IPA (9:1, 3 × 10 mL). The combined organic extracts were then dried ($MgSO_4$), filtered and concentrated *in vacuo*. The title compound was collected as an off-white foam (0.24 g, 0.77 mmol, 94%). No further purification was necessary.

^{1}H NMR (500 MHz, $CDCl_3$) δ 6.91 (d, *J* = 8.4 Hz, 2H), 6.63 (d, *J* = 8.4 Hz, 2H), 6.56 (s, 1H), 6.01 (s, 1H), 3.83 (s, 3H), 3.73 (dd, *J* = 8.1, 5.3 Hz, 1H), 3.56 (s, 3H), 3.32 – 3.21 (m, 1H), 3.14 (dd, *J* = 13.7, 5.3 Hz, 1H), 2.93 – 2.79 (m, 2H), 2.75 (dd, *J* = 13.7, 8.1 Hz, 1H), 2.67 – 2.58 (m, 1H), 2.53 (s, 3H).
Note: O*H* resonance was not observed.
^{13}C NMR (126 MHz, $CDCl_3$) δ 154.8, 147.5, 146.4, 131.1, 130.9(2C), 128.8, 125.4, 115.5(2C), 111.32, 111.30, 65.1, 55.9, 55.6, 46.2, 42.3, 40.6, 24.7.
$[\alpha]_D^{25}$ +95.3 (c. 0.93, $CHCl_3$). Ref.[21] $[\alpha]_D^{25}$ +94.2 (c. 1.0, $CHCl_3$).
Spectroscopic data is in agreement with that reported previously.[21]

(*R*)-4-((6,7-Dimethoxy-2-methyl-1,2,3,4-tetrahydroisoquinolin-1-yl)methyl)phenol ((*R*)-10b), (−)-armepavine

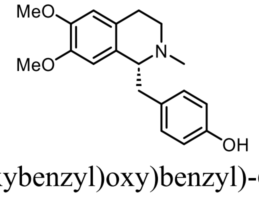

Method 1: (*R*)-1-(3-Bromo-4-((4-methoxybenzyl)oxy)benzyl)-6,7-dimethoxy-2-methyl-1,2,3,4-tetrahydro-isoquinoline **(*R*)-9c** (220 mg, 0.429 mmol), Pd/C (10% wt., 66.0 mg, 62.0 μmol, 0.14 equiv.) and triethylamine (600 μL, 4.29 mmol) in anhydrous MeOH (4.3 mL) were submitted to **General Procedure F** for 30 h. Upon completion, the reaction mixture was filtered through a pad of Celite®, the filter cake was washed with

CH_2Cl_2/MeOH (8:2, 100 mL) and the filtrate was concentrated *in vacuo*. The residue was then diluted with $CHCl_3$, washed with HCl (aq., 1 M, 3 mL) and $NaHCO_3$ (aq., sat., to pH 7). The aqueous phase was extracted with $CHCl_3$:IPA (9:1, 3 × 15 mL). The combined organic extracts were then dried ($MgSO_4$), filtered and concentrated *in vacuo*. The title compound was collected as an off-white foam (116 mg, 0.370 mmol, 86%). No further purification was necessary.
$[\alpha]_D^{25}$ –92.8 (c. 0.90, $CHCl_3$).

Method 2: To an oven-dried flask containing Pd/C (10% wt., 34 mg, 32 µmol, 0.04 equiv.) under an inert atmosphere, was added anhydrous MeOH (1.0 mL) and the flask was evacuated and backfilled with hydrogen (× 3). (*R*)-1-(4-(Benzyloxy)benzyl)-6,7-dimethoxy-2-methyl-1,2,3,4-tetrahydroiso-quinoline **(*R*)-9d** (0.34 g, 0.84 mmol, 1.0 equiv.) was then added as a solution in MeOH (7.0 mL) and AcOH (0.11 mL, 1.8 mmol, 2.2 equiv.). Using a gas bag, hydrogen was continuously bubbled through the reaction mixture by having the needle submerged in the reaction mixture and a second exit needle for 18 h. The gas bag was refilled as needed. Upon completion, the reaction mixture was filtered through a pad of Celite®, the filter cake was washed with CH_2Cl_2/MeOH (8:2, 60 mL) and the filtrate was concentrated *in vacuo*. The residue was then diluted with $CHCl_3$ and washed with $NaHCO_3$ (aq., sat., to pH 7). The aqueous phase was then extracted with $CHCl_3$:IPA (9:1, 3 × 15 mL). The combined organic extracts were then dried ($MgSO_4$), filtered and concentrated *in vacuo*. The title compound was collected as an off-white foam (0.24 g, 0.75 mmol, 90%). No further purification was necessary.

^{1}H NMR (500 MHz, $CDCl_3$) δ 6.89 (d, *J* = 8.5 Hz, 2H), 6.62 (d, *J* = 8.5 Hz, 2H), 6.56 (s, 1H), 5.96 (s, 1H), 3.82 (s, 3H), 3.74 (dd, *J* = 8.5, 5.0 Hz, 1H), 3.52 (s, 3H), 3.29 – 3.25 (m, 1H), 3.16 (dd, *J* = 13.6, 5.0 Hz, 1H), 2.95 – 2.80 (m, 2H), 2.73 (dd, *J* = 13.6, 8.5 Hz, 1H), 2.65 – 2.61 (m, 1H), 2.53 (s, 3H).
Note: O*H* resonance was not observed.
^{13}C NMR (126 MHz, $CDCl_3$) δ 155.2, 147.5, 146.0, 130.9(2C), 130.5, 128.5, 125.1, 115.6(2C), 111.34, 111.26, 65.0, 55.8, 55.5, 45.9, 42.0, 40.5, 24.5.
$[\alpha]_D^{25}$ –94.9 (c. 0.84, $CHCl_3$). Ref.[21] (+)-armepavine $[\alpha]_D^{25}$ +94.2 (c. 1.0, $CHCl_3$).
Spectroscopic data is in agreement with that reported previously.[30]

(*S*)-6,7-Dimethoxy-1-(4-((4-methoxybenzyl)oxy)benzyl)-2-methyl-1,2,3,4-tetrahydroisoquinoline, ((*S*)-10c)

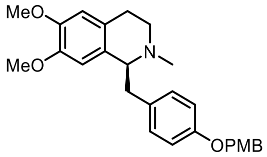

To an oven-dried vial containing Pd/C (10% wt., 6.5 mg, 6.1 µmol, 0.10 equiv.) under an inert atmosphere, was added anhydrous MeOH (0.1 mL) and the vial was evacuated and backfilled with hydrogen (× 3). (*S*)-1-(3-Bromo-4-((4-methoxybenzyl)oxy)benzyl)-6,7-dimethoxy-2-methyl-1,2,3,4-tetrahydroisoquinoline **(*S*)-9c** (31 mg, 61 µmol, 1.0 equiv.) was then added as a solution in MeOH (0.5 mL) followed by the addition of AcOH (7.0 µL, 13 µmol, 2.2 equiv.). The mixture was stirred under a hydrogen atmosphere (three-layered balloon) for 18 h. Upon completion, the reaction mixture was filtered through a pad of Celite®, the filter cake was washed with CH_2Cl_2/MeOH (9:1) and the filtrate was concentrated *in vacuo*. The residue was then diluted with CH_2Cl_2, neutralised with $NaHCO_3$ (aq., sat.) and the aqueous phase was extracted with $CHCl_3$/IPA (9:1, 2 × 7 mL). The combined organic extracts were dried ($MgSO_4$), filtered and concentrated *in vacuo*. The title compound was collected as an off-white foam (26 mg, 60 µmol, 98%). No further purification was necessary.

^{1}H NMR (500 MHz, $CDCl_3$) δ 7.34 (d, *J* = 8.7 Hz, 2H), 7.00 (d, *J* = 8.6 Hz, 2H), 6.91 (d, *J* = 8.7 Hz, 2H), 6.87 (d, *J* = 8.6 Hz, 2H), 6.55 (s, 1H), 6.00 (s, 1H), 4.96 (s, 2H), 3.83 (s, 3H), 3.81 (s, 3H), 3.68 (dd, *J* = 7.9,

5.0 Hz, 1H), 3.52 (s, 3H), 3.22 – 3.17 (m, 1H), 3.15 (dd, J = 13.7, 5.0 Hz, 1H), 2.89 – 2.70 (m, 3H), 2.60 (dt, J = 15.8, 4.6 Hz, 1H), 2.53 (s, 3H).
^{13}C NMR (126 MHz, $CDCl_3$) δ 159.5, 157.3, 147.3, 146.4, 132.3, 130.9(2C), 129.3(2C), 129.2(2C), 125.9, 114.7(2C), 114.1(2C), 111.3, 111.2, 69.9, 65.1, 55.9, 55.6, 55.4, 46.9, 42.7, 40.5, 25.5.
IR (film) v_{max}/cm^{-1} 3001, 2836, 1612, 1514, 1465, 1252, 1175, 1139, 1102.
HRMS (ESI^+) $C_{27}H_{31}NO_4$ found $[M+H]^+$ at 434.2315, expected at 434.2326.
$[\alpha]_D^{25}$ +58.1 (c. 1.31, $CHCl_3$).

(*R*)-6,7-Dimethoxy-1-(4-((4-methoxybenzyl)oxy)benzyl)-2-methyl-1,2,3,4-tetrahydroisoquinoline, ((*R*)-10c)

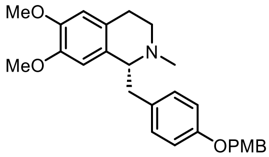

To an oven-dried vial containing Pd/C (10% wt., 9 mg, 0.84 mmol, 0.10 equiv.) under an inert atmosphere, was added anhydrous MeOH (0.05 mL) and the vial was evacuated and backfilled with hydrogen (× 3). (*R*)-1-(3-Bromo-4-((4-methoxybenzyl)oxy)benzyl)-6,7-dimethoxy-2-methyl-1,2,3,4-tetrahydroisoquinoline **(*R*)-9c** (42 mg, 8.2 mmol, 1.0 equiv.) was then added as a solution in MeOH (0.5 mL) followed by the addition of AcOH (9.5 µL, 18 mmol, 2.2 equiv.). The mixture was stirred under a hydrogen atmosphere (three-layered balloon) for 18 h. Upon completion, the reaction mixture was filtered through a pad of Celite®, the filter cake was washed with CH_2Cl_2/MeOH (9:1) and the filtrate was concentrated *in vacuo*. The residue was then diluted with CH_2Cl_2, neutralised with $NaHCO_3$ (aq., sat.) and the aqueous phase was extracted with $CHCl_3$/IPA (9:1, 2 × 5 mL). The combined organic extracts were then dried ($MgSO_4$), filtered and concentrated *in vacuo*. The title compound was collected as an off-white foam (35 mg, 8.1 mmol, 99%). No further purification was necessary.

^{1}H NMR (500 MHz, $CDCl_3$) δ 7.34 (d, J = 8.7 Hz, 2H), 7.00 (d, J = 8.5 Hz, 2H), 6.91 (d, J = 8.7 Hz, 2H), 6.87 (d, J = 8.5 Hz, 2H), 6.55 (s, 1H), 5.98 (s, 1H), 4.96 (s, 2H), 3.83 (s, 3H), 3.81 (s, 3H), 3.69 (dd, J = 8.0, 5.0 Hz, 1H), 3.52 (s, 3H), 3.24 – 3.19 (m, 1H), 3.16 (dd, J = 13.4, 5.0 Hz, 1H), 2.89 – 2.70 (m, 3H), 2.60 (dt, J = 15.6, 4.5 Hz, 1H), 2.54 (s, 3H).
^{13}C NMR (126 MHz, $CDCl_3$) δ 159.5, 157.3, 147.4, 146.4, 132.3, 130.9(2C), 129.3, 129.24(2C), 129.19, 125.8, 114.8(2C), 114.1(2C), 111.3, 111.2, 69.9, 65.0, 55.9, 55.6, 55.4, 46.8, 42.7, 40.5, 25.5.
IR (film) v_{max}/cm^{-1} 3997, 2837, 1613, 1515, 1466, 1253, 1176, 1139, 1103.
HRMS (ESI^+) $C_{27}H_{31}NO_4$ found $[M+H]^+$ at 434.2316, expected at 434.2326.
$[\alpha]_D^{25}$ –57.4 (c. 0.35, $CHCl_3$).

(*S*)-1-(4-(5-(((*S*)-6,7-Dimethoxy-2-methyl-1,2,3,4-tetrahydroisoquinolin-1-yl)methyl)-2-methoxyphenoxy)benzyl)-6,7-dimethoxy-2-methyl-1,2,3,4-tetrahydroisoquinoline ((*S,S*)-11), (+)-*O*-methylthalibrine

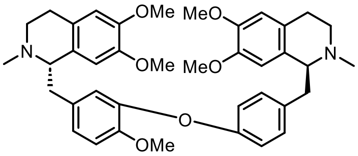

(*S*)-1-(4-Bromobenzyl)-6,7-dimethoxy-2-methyl-1,2,3,4-tetrahydroisoquinoline **(*S*)-9a** (60 mg, 0.16 mmol), (*S*)-5-((6,7-dimethoxy-2-methyl-1,2,3,4-tetrahydroisoquinolin-1-yl)methyl)-2-methoxyphenol **(*S*)-10a** (61 mg, 0.18 mmol), potassium carbonate (55 mg, 0.40 mmol) and cupric oxide (25 mg, 0.32 mmol) in anhydrous pyridine (0.25 mL) were submitted to **General Procedure G**. The title compound was isolated as a pale-yellow foam (38 mg, 59 µmol, 37%).

^{1}H NMR (500 MHz, CDCl3) δ 6.99 (d, *J* = 8.5 Hz, 2H), 6.87 (d, *J* = 8.3 Hz, 1H), 6.82 (dd, *J* = 8.3, 2.1 Hz, 1H), 6.77 (d, *J* = 8.5 Hz, 2H), 6.73 (d, *J* = 2.1 Hz, 1H), 6.54 (s, 1H), 6.51 (s, 1H), 6.09 (s, 1H), 6.03 (s, 1H), 3.82 (s, 3H), 3.80 (s, 3H), 3.79 (s, 3H), 3.68 (dd, *J* = 7.9, 5.0 Hz, 1H), 3.64 (dd, *J* = 7.3, 5.4 Hz, 1H), 3.61 (s, 3H), 3.57 (s, 3H), 3.21 – 3.02 (m, 4H), 2.87 – 2.67 (m, 6H), 2.59 – 2.55 (m, 2H), 2.52 (s, 3H), 2.48 (s, 3H).
^{13}C NMR (126 MHz, CDCl3) δ 156.4, 149.8, 147.38, 147.36, 146.5, 146.4, 144.7, 134.0, 133.1, 130.9(2C), 129.4, 129.2, 126.3, 126.0, 126.1, 122.6, 116.8(2C), 112.6, 111.31, 111.28, 111.2, 111.0, 65.0, 64.8, 56.2, 55.9, 55.8, 55.71, 55.65, 47.1, 47.0, 42.83, 42.79, 40.7, 40.4, 25.7, 25.6.
IR (film) ν_{max}/cm^{-1} 2998, 2935, 2907, 2834, 2795, 1609, 1507, 1463, 1444, 1259, 1225, 1168, 1138, 757.
HRMS (ESI^{+}) $C_{39}H_{46}N_2O_6$ found [M+H]$^{+}$ at 639.3427, expected at 639.3429.
[α]$_D^{25}$ +72.8 (c. 1.21, $CHCl_3$). Ref [21] [α]$_D^{25}$ +79.2 (c. 0.45, $CHCl_3$).
Spectroscopic data is in agreement with that reported previously.[21]

(*R*)-1-(4-(5-(((*R*)-6,7-Dimethoxy-2-methyl-1,2,3,4-tetrahydroisoquinolin-1-yl)methyl)-2-methoxyphenoxy)benzyl)-6,7-dimethoxy-2-methyl-1,2,3,4-tetrahydroisoquinoline ((*R,R*)-11), (−)-*O*-methyldauricine

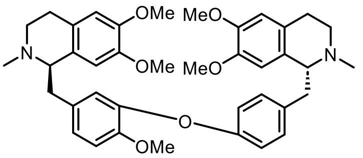

(*R*)-1-(4-Bromobenzyl)-6,7-dimethoxy-2-methyl-1,2,3,4-tetrahydroisoquinoline **(*R*)-9a** (60 mg, 0.16 mmol), (*R*)-5-((6,7-dimethoxy-2-methyl-1,2,3,4-tetrahydroisoquinolin-1-yl)methyl)-2-methoxyphenol **(*R*)-10a** (60 mg, 0.18 mmol), potassium carbonate (55 mg, 0.40 mmol) and cupric oxide (25 mg, 0.32 mmol) in anhydrous pyridine (0.25 mL) were subjected to **General Procedure G**. The title compound was isolated as a pale-yellow foam (17 mg, 27 μmol, 17%)

^{1}H NMR (500 MHz, $CDCl_3$) δ 7.00 (d, *J* = 8.6 Hz, 2H), 6.87 (d, *J* = 8.3 Hz, 1H), 6.82 (dd, *J* = 8.3, 2.1 Hz, 1H), 6.78 (d, *J* = 8.6 Hz, 2H), 6.73 (d, *J* = 2.1 Hz, 1H), 6.54 (s, 1H), 6.51 (s, 1H), 6.08 (s, 1H), 6.03 (s, 1H), 3.83 (s, 3H), 3.81 (s, 3H), 3.79 (s, 3H), 3.68 (dd, *J* = 7.7, 5.1 Hz, 1H), 3.64 (dd, *J* = 7.3, 5.4 Hz, 1H), 3.61 (s, 3H), 3.57 (s, 3H), 3.23 – 3.01 (m, 4H), 2.87 – 2.68 (m, 6H), 2.60 – 2.66 (m, 2H), 2.52 (s, 3H), 2.48 (s, 3H).
^{13}C NMR (126 MHz, $CDCl_3$) δ 156.4, 149.8, 147.37, 147.34, 146.5, 146.4, 144.7, 134.0, 133.1, 130.9(2C), 129.4, 129.2, 126.3, 126.0, 126.1, 122.6, 116.8(2C), 112.6, 111.29, 111.26, 111.2, 111.0, 65.0, 64.8, 56.2, 55.9, 55.8, 55.72, 55.65, 47.1, 47.0, 42.84, 42.81, 40.7, 40.4, 25.7, 25.6.
IR (film) ν_{max}/cm^{-1} 3017, 2937, 2907, 2834, 2795, 1609, 1507, 1464, 1450, 1257, 1225, 1169, 1126, 756.
HRMS (ESI^{+}) $C_{39}H_{46}N_2O_6$ found [M+H]$^{+}$ at 639.3424, expected at 639.3429.
[α]$_D^{25}$ −81.7 (c. 1.15, $CHCl_3$). Ref [31] [α]$_D^{9}$ −64.2 (c. 1.32, $CHCl_3$).
Spectroscopic data is in agreement with that reported previously.[31]

(*R*)-1-(4-(5-(((*S*)-6,7-Dimethoxy-2-methyl-1,2,3,4-tetrahydroisoquinolin-1-yl)methyl)-2-methoxyphenoxy)benzyl)-6,7-dimethoxy-2-methyl-1,2,3,4-tetrahydroisoquinoline ((*S,R*)-11), (−)-*O,O'*-dimethylgrisabine

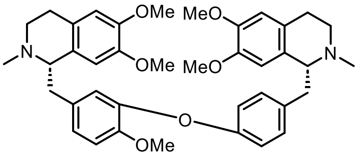

(*R*)-1-(4-bromobenzyl)-6,7-dimethoxy-2-methyl-1,2,3,4-tetrahydroisoquinoline **(*R*)-9a** (66 mg, 0.18 mmol), (*S*)-5-((6,7-dimethoxy-2-methyl-1,2,3,4-tetrahydroisoquinolin-1-yl)methyl)-2-methoxyphenol **(*S*)-10a** (66 mg, 0.19 mmol), potassium carbonate (61 mg, 0.44 mmol) and cupric oxide (28 mg, 0.35 mmol) in anhydrous

pyridine (0.28 mL) were submitted to **General Procedure G**. The title compound was isolated as a pale-yellow foam (25 mg, 40 μmol, 23%).

^{1}H NMR (500 MHz, $CDCl_3$) δ 7.00 (d, J = 8.6 Hz, 2H), 6.87 (d, J = 8.3 Hz, 1H), 6.82 (dd, J = 8.3, 2.1 Hz, 1H), 6.78 (d, J = 8.6 Hz, 2H), 6.74 (d, J = 2.1 Hz, 1H), 6.54 (s, 1H), 6.52 (s, 1H), 6.08 (s, 1H), 6.02 (s, 1H), 3.83 (s, 3H), 3.82 (s, 3H), 3.79 (s, 3H), 3.69 (dd, J = 7.8, 5.1 Hz, 1H), 3.64 (dd, J = 7.4, 5.3 Hz, 1H), 3.61 (s, 3H), 3.57 (s, 3H), 3.23 – 3.01 (m, 4H), 2.88 – 2.68 (m, 6H), 2.62 – 2.54 (m, 2H), 2.53 (s, 3H), 2.48 (s, 3H).
^{13}C NMR (126 MHz, $CDCl_3$) δ 156.4, 149.8, 147.38, 147.34, 146.5, 146.4, 144.7, 134.0, 133.1, 130.9(2C), 129.3, 129.2, 126.2, 126.01, 126.02, 122.6, 116.8(2C), 112.6, 111.30, 111.26, 111.14, 110.98, 65.0, 64.8, 56.2, 55.87, 55.85, 55.71, 55.66, 47.02, 46.98, 42.81, 42.80, 40.6, 40.4, 25.6(2C).
IR (film) ν_{max}/cm^{-1} 3000, 2935, 2909, 2835, 2795, 1610, 1507, 1464, 1450, 1258, 1226, 1169, 1138, 760.
HRMS (ESI$^+$) $C_{39}H_{46}N_2O_6$ found $[M+H]^+$ at 639.3432, expected at 639.3429.
$[\alpha]_D^{25}$ –25.0 (c. 1.12, $CHCl_3$). Ref [32] $[\alpha]_D$ –10 (c. 1.0, $CHCl_3$).
Spectroscopic data is in agreement with that reported previously.[32]

(*S*)-1-(4-(5-(((*R*)-6,7-Dimethoxy-2-methyl-1,2,3,4-tetrahydroisoquinolin-1-yl)methyl)-2-methoxyphenoxy)benzyl)-6,7-dimethoxy-2-methyl-1,2,3,4-tetrahydroisoquinoline ((*R*,*S*)-11)

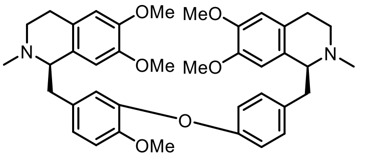

(*S*)-1-(4-bromobenzyl)-6,7-dimethoxy-2-methyl-1,2,3,4-tetrahydroisoquinoline **(*S*)-9a** (60 mg, 0.16 mmol), (*R*)-5-((6,7-dimethoxy-2-methyl-1,2,3,4-tetrahydroisoquinolin-1-yl)methyl)-2-methoxyphenol **(*R*)-10a** (60 mg, 0.18 mmol), potassium carbonate (55 mg, 0.40 mmol) and cupric oxide (25 mg, 0.32 mmol) in anhydrous pyridine (0.25 mL) were submitted to **General Procedure G**. The title compound was isolated as a pale-yellow foam (22 mg, 0.035 mmol, 22%).

^{1}H NMR (500 MHz, $CDCl_3$) δ 7.00 (d, J = 8.5 Hz, 2H), 6.87 (d, J = 8.3 Hz, 1H), 6.82 (dd, J = 8.3, 2.1 Hz, 1H), 6.78 (d, J = 8.5 Hz, 2H), 6.73 (d, J = 2.1 Hz, 1H), 6.55 (s, 1H), 6.52 (s, 1H), 6.06 (s, 1H), 6.00 (s, 1H), 3.83 (s, 3H), 3.82 (s, 3H), 3.79 (s, 3H), 3.72 (dd, J = 8.0, 4.9 Hz, 1H), 3.66 (dd, J = 7.4, 5.3 Hz, 1H), 3.60 (s, 3H), 3.56 (s, 3H), 3.24 – 3.05 (m, 4H), 2.89 – 2.70 (m, 6H), 2.63 – 2.56 (m, 2H), 2.55 (s, 3H), 2.49 (s, 3H).
^{13}C NMR (126 MHz, $CDCl_3$) δ 156.5, 149.9, 147.40, 147.37, 146.5, 146.4, 144.7, 134.0, 133.1, 130.9(2C), 129.3, 129.2, 126.2, 126.0, 126.1, 122.6, 116.8(2C), 112.6, 111.31, 111.28, 111.2, 111.0, 65.0, 64.8, 56.2, 55.89, 55.87, 55.72, 55.68, 47.03, 46.98, 42.81, 42.80, 40.7, 40.5, 25.6(2C).
IR (film) ν_{max}/cm^{-1} 3020, 2935, 2907, 2835, 2794, 1610, 1509, 1464, 1443, 1259, 1226, 1169, 1126, 762.
HRMS (ESI$^+$) $C_{39}H_{46}N_2O_6$ found $[M+H]^+$ at 639.3425, expected at 639.3429.
$[\alpha]_D^{25}$ +23.7 (c. 0.88, $CHCl_3$). Ref [33] $[\alpha]_D$ +14 (c. unknown, $CHCl_3$).
Some spectroscopic data has been reported previously.[33]

(*S*)-1-(4-(5-(((*R*)-6,7-Dimethoxy-2-methyl-1,2,3,4-tetrahydroisoquinolin-1-yl)methyl)-2-((4-methoxybenzyl)oxy) phenoxy)benzyl)-6,7-dimethoxy-2-methyl-1,2,3,4-tetrahydroisoquinoline, ((*S,R*)-12)

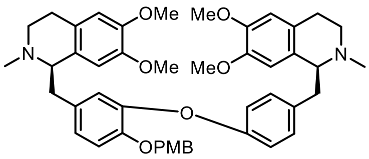

(+)-Armepavine **(*S*)-10b** (80.0 mg, 0.255 mmol), (*R*)-1-(3-bromo-4-((4-methoxybenzyl)oxy)benzyl)-6,7-dimethoxy-2-methyl-1,2,3,4-tetrahydroisoquinoline **(*R*)-9c** (119 mg, 0.232 mmol), potassium carbonate (80.2 mg, 0.580 mmol) and cupric oxide (36.8 mg, 0.463 mmol) in anhydrous pyridine (0.40 mL) were submitted to **General Procedure G**. The title compound was isolated as an off-white foam (50.6 mg, 67.9 µmol, 29%).

^{1}H NMR (400 MHz, $CDCl_3$) δ 7.15 (d, J = 8.7 Hz, 2H), 7.01 (d, J = 8.6 Hz, 2H), 6.90 (d, J = 8.8 Hz, 1H), 6.83 – 6.75 (m, 6H), 6.55 (s, 1H), 6.52 (s, 1H), 6.05 (s, 1H), 6.04 (s, 1H), 4.98 (s, 2H), 3.82 (s, 3H), 3.81 (s, 3H), 3.76 (s, 3H), 3.69 (dd, J = 7.7, 5.1 Hz, 1H), 3.65 (dd, J = 7.6, 5.2 Hz, 1H), 3.55 (s, 3H), 3.52 (s, 3H), 3.23 – 3.04 (m, 4H), 2.88 – 2.68 (m, 6H), 2.64 – 2.60 (m, 1H), 2.59 – 2.55 (m, 1H), 2.53 (s, 3H), 2.48 (s, 3H).
^{13}C NMR (101 MHz, $CDCl_3$) δ 159.4, 156.8, 148.9, 147.41(2C), 146.5, 146.4, 145.4, 133.71, 133.67, 130.8(2C), 129.2, 129.1, 129.0, 128.9(2C), 126.1, 125.9, 125.8, 123.2, 116.7(2C), 115.6, 113.9(2C), 111.28, 111.26, 111.1, 111.0, 71.0, 65.0, 64.8, 55.86(2C), 55.6(2C), 55.4, 46.9, 46.8, 42.7(2C), 40.7, 40.5, 25.5, 25.4.
IR (film) ν_{max}/cm^{-1} 3019, 2934, 2835, 2796, 1612, 1577, 1513, 1464, 1253, 1219, 1173, 1138, 1122, 1102, 1068, 753.
HRMS (ESI^{+}) $C_{46}H_{52}N_2O_7$ found $[M+H]^+$ at 745.3830, expected at 745.3847.
$[\alpha]_D^{25}$ +5.47 (c. 1.51, $CHCl_3$).

(*R*)-1-(4-(5-(((*S*)-6,7-Dimethoxy-2-methyl-1,2,3,4-tetrahydroisoquinolin-1-yl)methyl)-2-((4-methoxybenzyl)oxy)phenoxy)benzyl)-6,7-dimethoxy-2-methyl-1,2,3,4-tetrahydroisoquinoline, ((*R,S*)-12):

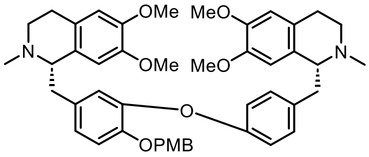

(–)-Armepavine **(*R*)-10b** (100 mg, 0.319 mmol), (*S*)-1-(3-bromo-4-((4-methoxybenzyl)oxy)benzyl)-6,7-dimethoxy-2-methyl-1,2,3,4-tetrahydroisoquinoline **(*S*)-9c** (149 mg, 0.290 mmol), potassium carbonate (100 mg, 0.725 mmol) and cupric oxide (46.1 mg, 0.580 mmol) in anhydrous pyridine (0.50 mL) were submitted to **General Procedure G**. The title compound was isolated as an off-white foam (54.9 mg, 73.6 µmol, 25%).

^{1}H NMR (500 MHz, $CDCl_3$) δ 7.15 (d, *J* = 8.6 Hz, 2H), 7.01 (d, *J* = 8.5 Hz, 2H), 6.90 (d, *J* = 8.9 Hz, 1H), 6.83 – 6.75 (m, 6H), 6.55 (s, 1H), 6.52 (s, 1H), 6.05 (s, 1H), 6.04 (1s, 1H), 4.98 (s, 2H), 3.82 (s, 3H), 3.81 (s, 3H), 3.77 (s, 3H), 3.69 (dd, *J* = 7.7, 5.1 Hz, 1H), 3.65 (dd, *J* = 7.6, 5.2 Hz, 1H), 3.55 (s, 3H), 3.53 (s, 3H), 3.24 – 3.03 (m, 4H), 2.88 – 2.68 (m, 6H), 2.60 (t, *J* = 4.7 Hz, 1H), 2.57 (q, *J* = 4.3 Hz, 1H), 2.53 (s, 3H), 2.49 (s, 3H).
^{13}C NMR (126 MHz, $CDCl_3$) δ 159.3, 156.8, 148.9, 147.4(2C), 146.5, 146.4, 145.4, 133.83, 133.77, 130.8(2C), 129.3, 129.20, 129.17, 128.9(2C), 126.1(2C), 125.9, 123.2, 116.7(2C), 115.6, 113.9(2C), 111.28, 111.26, 111.1, 111.0, 71.0, 65.0, 64.8, 55.85, 55.84, 55.6(2C), 55.3, 47.0, 46.9, 42.8(2C), 40.7, 40.5, 25.6, 25.5.
IR (film) ν_{max}/cm^{-1} 3016, 2935, 2835, 2795, 1611, 1585, 1513, 1464, 1253, 1219, 1173, 1138, 1123, 1102, 1067, 757.
HRMS (ESI^{+}) $C_{46}H_{52}N_2O_7$ found $[M+H]^+$ at 745.3833, expected at 745.3847.
$[\alpha]_D^{25}$ –5.81 (c. 2.39, $CHCl_3$).

(*S*)-1-(4-(5-(((*S*)-6,7-Dimethoxy-2-methyl-1,2,3,4-tetrahydroisoquinolin-1-yl)methyl)-2-((4-methoxybenzyl)oxy)phenoxy)benzyl)-6,7-dimethoxy-2-methyl-1,2,3,4-tetrahydroisoquinoline, ((*S,S*)-12)

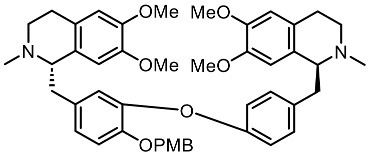

(+)-Armepavine **(*S*)-10b** (135 mg, 0.431 mmol), (*S*)-1-(3-bromo-4-((4-methoxybenzyl)oxy)benzyl)-6,7-dimethoxy-2-methyl-1,2,3,4-tetrahydroisoquinoline **(*S*)-9c** (200 mg, 0.390 mmol), potassium carbonate (135 mg, 0.976 mmol) and cupric oxide (62.0 mg, 0.779 mmol) in anhydrous pyridine (0.70 mL) were submitted to **General Procedure G**. The title compound was isolated as an off-white foam (108 mg, 0.146 mmol, 37%).

^{1}H NMR (500 MHz, $CDCl_3$) δ 7.15 (d, *J* = 8.7 Hz, 2H), 7.00 (d, *J* = 8.6 Hz, 2H), 6.89 (d, *J* = 8.8 Hz, 1H), 6.83 – 6.75 (m, 6H), 6.55 (s, 1H), 6.52 (s, 1H), 6.05 (s, 2H), 4.98 (s, 2H), 3.82 (s, 3H), 3.80 (s, 3H), 3.76 (s, 3H), 3.69 (dd, *J* = 7.6, 5.2 Hz, 1H), 3.65 (dd, *J* = 7.5, 5.1 Hz, 1H), 3.55 (s, 3H), 3.53 (s, 3H), 3.24 – 3.03 (m, 4H), 2.88 – 2.68 (m, 6H), 2.60 (t, *J* = 4.7 Hz, 1H), 2.57 (t, *J* = 4.5 Hz, 1H), 2.53 (s, 3H), 2.49 (s, 3H).
^{13}C NMR (126 MHz, $CDCl_3$) δ 159.3, 156.7, 148.9, 147.35, 147.34, 146.44, 146.42, 145.41, 133.8, 133.7, 130.8(2C), 129.3, 129.2, 129.1, 128.9(2C), 126.10(2C), 125.9, 123.1, 116.7(2C), 115.6, 113.9(2C), 111.25(2C), 111.1, 110.9, 71.0, 65.0, 64.8, 55.84, 55.80, 55.64, 55.63, 55.33, 47.0, 46.9, 42.78, 42.75, 40.7, 40.5, 25.6, 25.5.
IR (film) ν_{max}/cm^{-1} 3021, 2935, 2835, 2794, 1612, 1586, 1514, 1464, 1253, 1220, 1173, 1138, 1122, 1103, 1033, 759.
HRMS (ESI$^+$) $C_{46}H_{52}N_2O_7$ found $[M+H]^+$ at 745.3833, expected at 745.3847.
$[\alpha]_D^{25}$ +76.9 (c. 1.81, $CHCl_3$).

(*R*)-1-(4-(5-(((*R*)-6,7-Dimethoxy-2-methyl-1,2,3,4-tetrahydroisoquinolin-1-yl)methyl)-2-((4-methoxybenzyl)oxy)phenoxy)benzyl)-6,7-dimethoxy-2-methyl-1,2,3,4-tetrahydroisoquinoline ((*R,R*)-12)

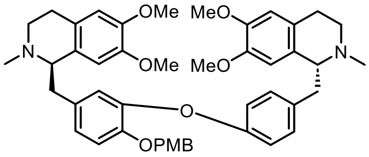

(–)-Armepavine **(*R*)-10b** (80.0 mg, 0.255 mmol), (*R*)-1-(3-bromo-4-((4-methoxybenzyl)oxy)benzyl)-6,7-dimethoxy-2-methyl-1,2,3,4-tetrahydroisoquinoline **(*R*)-9c** (119 mg, 0.232 mmol), potassium carbonate (80.2 mg, 0.580 mmol) and cupric oxide (36.8 mg, 0.463 mmol) in anhydrous pyridine (0.40 mL) were submitted to **General Procedure G**. The title compound was isolated as an off-white foam (42.4 mg, 56.9 µmol, 25%).

^{1}H NMR (500 MHz, $CDCl_3$) δ 7.15 (d, *J* = 8.7 Hz, 2H), 7.00 (d, *J* = 8.5 Hz, 2H), 6.89 (d, *J* = 8.8 Hz, 1H), 6.83 – 6.75 (m, 6H), 6.55 (s, 1H), 6.52 (s, 1H), 6.05 (s, 2H), 4.98 (s, 2H), 3.82 (s, 3H), 3.81 (s, 3H), 3.77 (s, 3H), 3.69 (dd, *J* = 7.7, 5.1 Hz, 1H), 3.65 (dd, *J* = 7.5, 5.2 Hz, 1H), 3.55 (s, 3H), 3.53 (s, 3H), 3.24 – 3.03 (m, 4H), 2.88 – 2.69 (m, 6H), 2.60 (t, *J* = 4.7 Hz, 1H), 2.57 (t, *J* = 4.6 Hz, 1H), 2.53 (s, 3H), 2.49 (s, 3H).
^{13}C NMR (126 MHz, $CDCl_3$) δ 159.4, 156.7, 148.9, 147.37, 147.38, 146.46, 146.44, 145.42, 133.8, 133.7, 130.8(2C), 129.3, 129.2, 129.1, 128.9(2C), 126.1(2C), 125.9, 123.1, 116.7(2C), 115.6, 113.9(2C), 111.26(2C), 111.1, 110.9, 71.1, 65.1, 64.8, 55.85, 55.82, 55.65, 55.64, 55.4, 47.0, 46.9, 42.79, 42.75, 40.8, 40.5, 25.6, 25.5.
IR (film) ν_{max}/cm^{-} 3014, 2934, 2835, 2794, 1611, 1585, 1513, 1464, 1253, 1223, 1173, 1138, 1123, 1103, 1016, 759.
HRMS (ESI$^+$) $C_{46}H_{52}N_2O_7$ found $[M+H]^+$ at 745.3829, expected at 745.3847.
$[\alpha]_D^{25}$ –75.9 (c. 1.40, $CHCl_3$).

4-(((*R*)-6,7-Dimethoxy-2-methyl-1,2,3,4-tetrahydroisoquinolin-1-yl)methyl)-2-(4-(((*S*)-6,7-dimethoxy-2-methyl-1,2,3,4-tetrahydroisoquinolin-1-yl)methyl)phenoxy)phenol ((*S,R*)-13)

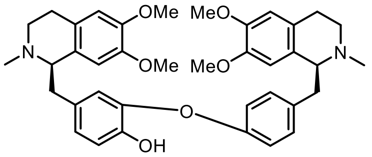

(*S*)-1-(4-(5-(((*R*)-6,7-Dimethoxy-2-methyl-1,2,3,4-tetrahydroisoquinolin-1-yl)methyl)-2-((4-methoxy benzyl)oxy)phenoxy)benzyl)-6,7-dimethoxy-2-methyl-1,2,3,4-tetrahydroisoquinoline **(*S,R*)-12** (18 mg, 25 μmol), Pd/C (10% wt., 5.4 mg, 5.1 μmol, 0.20 equiv.) and triethylamine (30 μL, 25 mmol) in anhydrous MeOH (0.25 mL) were submitted to **General Procedure F** for 20 h. The title compound was isolated as an off-white foam (9.1 mg, 15 μmol, 60%).

^{1}H NMR (600 MHz, $CDCl_3$) δ 7.06 (d, *J* = 8.5 Hz, 2H), 6.95 (d, *J* = 8.2 Hz, 1H), 6.85 – 6.81 (m, 3H), 6.59 (s, 2H), 6.55 (s, 1H), 6.05 (s, 1H), 6.01 (s, 1H), 3.86 (s, 3H), 3.84 (s, 3H), 3.84 – 3.80 (m, 1H), 3.77 – 3.72 (m, 1H), 3.61 (s, 3H), 3.59 (s, 3H), 3.34 – 3.16 (m, 4H), 2.94 – 2.81 (m, 5H), 2.72 (dd, *J* = 13.6, 8.2 Hz, 1H), 2.69 – 2.61 (m, 2H), 2.60 (s, 3H), 2.56 (s, 3H).
Note: O*H* resonance was not observed.
^{13}C NMR (151 MHz, $CDCl_3$) δ 155.5, 147.83, 147.78, 146.77, 146.67, 146.14, 143.10, 134.4, 131.7, 131.2(2C), 127.7(2C), 126.2, 125.3(2C), 120.8, 117.5(2C), 116.1, 111.40, 111.35, 111.2(2C), 64.89, 64.85, 55.9(2C), 55.8, 55.7, 46.5(2C), 42.2(2C), 40.6, 40.5, 24.8(2C).
IR (film) ν_{max}/cm^{-1} 3022, 2933, 2850, 2836, 1611, 1593, 1512, 1465, 1451, 1289, 1256, 1226, 1137, 1116, 1015.
HRMS (ESI^+) $C_{38}H_{44}N_2O_6$ found $[M+H]^+$ at 625.3264, expected at 625.3272.
$[\alpha]_D^{25}$ +15.8 (c. 1.03, $CHCl_3$).

4-(((*S*)-6,7-Dimethoxy-2-methyl-1,2,3,4-tetrahydroisoquinolin-1-yl)methyl)-2-(4-(((*R*)-6,7-dimethoxy-2-methyl-1,2,3,4-tetrahydroisoquinolin-1-yl)methyl)phenoxy)phenol ((*R,S*)-13)

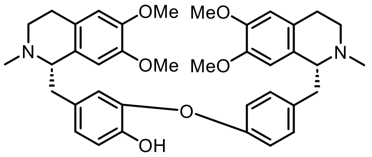

(*R*)-1-(4-(5-(((*S*)-6,7-Dimethoxy-2-methyl-1,2,3,4-tetrahydroisoquinolin-1-yl)methyl)-2-((4-methoxybenzyl) oxy)phenoxy)benzyl)-6,7-dimethoxy-2-methyl-1,2,3,4-tetrahydroisoquinoline **(*R,S*)-12** (32 mg, 43 μmol), Pd/C (10% wt., 9.3 mg, 8.7 μmol, 0.20 equiv.) and triethylamine (60 μL, 43 mmol) in anhydrous MeOH (0.43 mL) were submitted to **General procedure F** for 20 h. The title compound was isolated as an off-white foam (15 mg, 23 μmol, 54%).

^{1}H NMR (600 MHz, $CDCl_3$) δ 7.06 (d, *J* = 8.4 Hz, 2H), 6.96 (d, *J* = 8.2 Hz, 1H), 6.85 – 6.83 (m, 3H), 6.59 (s, 2H), 6.55 (s, 1H), 6.04 (s, 1H), 6.01 (s, 1H), 3.86 (s, 3H), 3.85 – 3.82 (m, 4H), 3.81 – 3.74 (m, 1H), 3.60(s, 3H), 3.59 (s, 3H), 3.34 – 3.20 (m, 4H), 2.95 – 2.81 (m, 5H), 2.73 (dd, *J* = 13.6, 8.4 Hz, 1H), 2.70 – 2.63 (m, 2H), 2.61 (s, 3H), 2.58 (s, 3H).
Note: O*H* resonance was not observed.
^{13}C NMR (151 MHz, $CDCl_3$) δ 155.5, 147.90, 147.85, 146.8, 146.7, 146.3, 143.1, 134.2, 131.4, 131.2(2C), 127.7(2C), 126.2, 125.2(2C), 120.9, 117.5(2C), 116.2, 111.39, 111.34, 111.2(2C), 64.91, 64.87, 55.93(2C), 55.79, 55.71, 46.5, 46.4, 42.1(2C), 40.6, 40.5, 24.7(2C).
IR (film) ν_{max}/cm^{-1} 3021, 2935, 2850, 2836, 1611, 1592, 1512, 1465, 1450, 1289, 1256, 1225, 1137, 1116, 1015.
HRMS (ESI^+) $C_{38}H_{44}N_2O_6$ found $[M+H]^+$ at 625.3266, expected at 625.3272.
$[\alpha]_D^{25}$ –14.4 (c. 1.32, $CHCl_3$).

4-(((*S*)-6,7-Dimethoxy-2-methyl-1,2,3,4-tetrahydroisoquinolin-1-yl)methyl)-2-(4-(((*S*)-6,7-dimethoxy-2-methyl-1,2,3,4-tetrahydroisoquinolin-1-yl)methyl)phenoxy)phenol ((*S*,*S*)-13), (+)-dauricine

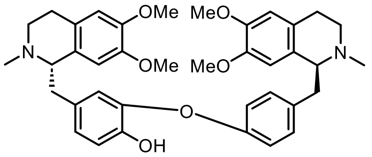

(*S*)-1-(4-(5-(((*S*)-6,7-Dimethoxy-2-methyl-1,2,3,4-tetrahydroisoquinolin-1-yl)methyl)-2-((4-methoxybenzyl) oxy)phenoxy)benzyl)-6,7-dimethoxy-2-methyl-1,2,3,4-tetrahydroisoquinoline, **(*S*,*S*)-12** (17 mg, 23 μmol), Pd/C (10% wt., 5.3 mg, 5.0 μmol, 0.20 equiv.) and triethylamine (30 μL, 23 mmol) in anhydrous MeOH (0.25 mL) were submitted to **General Procedure F** for 20 h. The title compound was isolated as an off-white foam (9.1 mg, 15 μmol, 65%).

^{1}H NMR (500 MHz, $CDCl_3$) δ 7.03 (d, *J* = 8.1 Hz, 2H), 6.92 (d, *J* = 8.2 Hz, 1H), 6.85 – 6.77 (m, 3H,), 6.57 (d, *J* = 2.0 Hz, 1H), 6.55 (s, 1H), 6.51 (s, 1H), 6.09 (s, 1H), 6.03 (s, 1H), 3.83 (s, 3H), 3.80 (s, 3H), 3.75 – 3.69 (m, 1H), 3.64 (dd, *J* = 8.0, 4.8 Hz, 1H), 3.60 (s, 6H), 3.24 – 3.09 (m, 3H), 3.06 (dd, *J* = 13.7, 5.1 Hz, 1H), 2.88 – 2.65 (m, 6H), 2.62 – 2.53 (m, 2H), 2.52 (s, 3H), 2.49 (s, 3H).
Note: O*H* resonance was not observed.
^{13}C NMR (126 MHz, $CDCl_3$) δ 155.3, 147.54, 147.46, 146.6, 146.5, 145.9, 143.0, 135.0, 132.3, 131.2(2C), 129.0, 128.9, 126.1(3C), 120.6, 117.5(2C), 115.9, 111.4, 111.3, 111.10, 111.08, 64.87, 64.86, 55.9, 55.8, 55.74, 55.71, 46.93, 46.85, 42.69, 42.66, 40.7, 40.5, 25.5, 25.3.
IR (film) v_{max}/cm^{-1} 3021, 2936, 2834, 1611, 1593, 1509, 1465, 1450, 1288, 1256, 1226, 1137, 1116, 1016, 758.
HRMS (ESI$^+$) $C_{38}H_{44}N_2O_6$ found $[M+H]^+$ at 625.3262, expected at 625.3272.
$[\alpha]_D^{25}$ +68.2 (c. 0.83, $CHCl_3$).
Spectroscopic data is in agreement with that reported previously.[34, 35]
Note: data is reported for (–)-dauricine **(*R*,*R*)-13**.

4-(((*R*)-6,7-Dimethoxy-2-methyl-1,2,3,4-tetrahydroisoquinolin-1-yl)methyl)-2-(4-(((*R*)-6,7-dimethoxy-2-methyl-1,2,3,4-tetrahydroisoquinolin-1-yl)methyl)phenoxy)phenol ((*R*,*R*)-13), (–)-dauricine

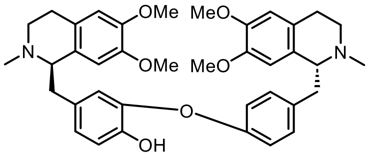

(*R*)-1-(4-(5-(((*R*)-6,7-Dimethoxy-2-methyl-1,2,3,4-tetrahydroisoquinolin-1-yl)methyl)-2-((4-methoxybenzyl) oxy)phenoxy)benzyl)-6,7-dimethoxy-2-methyl-1,2,3,4-tetrahydroisoquinoline **(*R*,*R*)-12** (40 mg, 54 μmol), Pd/C (10% wt., 12 mg, 11.3 μmol, 0.20 equiv.) and triethylamine (70 μL, 0.54 mmol) in anhydrous MeOH (0.54 mL) were submitted to **General Procedure F** for 20 h. The title compound was isolated as an off-white foam (19 mg, 30 μmol, 56%).

^{1}H NMR (500 MHz, $CDCl_3$) δ 7.03 (d, *J* = 8.4 Hz, 2H), 6.91 (d, *J* = 8.2 Hz, 1H), 6.85 – 6.77 (m, 3H), 6.57 (d, *J* = 2.0 Hz, 1H), 6.55 (s, 1H), 6.51 (s, 1H), 6.08 (s, 1H), 6.03 (s, 1H), 3.83 (s, 3H), 3.80 (s, 3H), 3.74 – 3.68 (m, 1H), 3.64 (dd, *J* = 7.6, 5.3 Hz, 1H), 3.59 (s, 6H), 3.23 – 3.10 (m, 3H), 3.05 (dd, *J* = 13.7, 5.1 Hz, 1H), 2.88 – 2.65 (m, 6H), 2.61 – 2.53 (m, 2H), 2.52 (s, 3H), 2.49 (s, 3H).
Note: O*H* resonance was not observed.
^{13}C NMR (126 MHz, $CDCl_3$) δ 155.3, 147.53, 147.45, 146.6, 146.5, 145.9, 143.0, 135.0, 132.3, 131.1(2C), 129.0, 128.9, 126.0(3C), 120.6, 117.4(2C), 115.9, 111.4, 111.3, 111.10, 111.08, 64.87, 64.85, 55.9, 55.8, 55.73, 55.70, 46.9, 46.8, 42.69, 42.65, 40.7, 40.5, 25.5, 25.3.
IR (film) v_{max}/cm^{-1} 3018, 2938, 2835, 2799, 1611, 1593, 1509, 1465, 1451, 1288, 1255, 1226, 1137, 1116, 1016, 757.

HRMS (ESI^+) $C_{38}H_{44}N_2O_6$ found $[M+H]^+$ at 625.3262, expected at 625.3272.
$[\alpha]_D^{25}$ −69.5 (c. 1.2, $CHCl_3$). Ref [35] $[\alpha]_D^{19}$ −115.1 (c. 0.73, MeOH).
Spectroscopic data is in agreement with that reported previously.[34, 35]

tert-Butyl 1-(4-bromobenzyl)-6,7-dimethoxy-3,4-dihydroisoquinoline-2(*1H*)-carboxylate, (rac-S1)

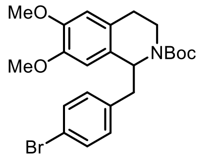

1-(4-Bromobenzyl)-6,7-dimethoxy-1,2,3,4-tetrahydroisoquinoline **rac-8a** (30 mg, 80 µmol), di-*tert*-butyl dicarbonate (21 mg, 96 µmol) and triethylamine (14 µL, 96 µmol) in anhydrous CH_2Cl_2 (0.4 mL) were submitted to **General Procedure H**. Purification by flash column chromatography (pentane/EtOAc, 4:1) afforded the title compound as a colourless oil (35 mg, 77 µmol, 96%).

^{1}H NMR (400 MHz, $CDCl_3$, analysis revealed the presence of two rotamers present in a 0.63:37 ratio, Major(M) and minor(m)) δ 7.48 – 7.29 (m, 2H, both rotamers), 7.01 – 6.93 (m, 2H, both rotamers), 6.63 – 6.56 (m, 1H, both rotamers), 6.34 (s, 0.63H, (M)), 6.18 (s, 0.37H, (m)), 5.21 (t, *J* = 7.0 Hz, 0.37H, (m)), 5.06 (t, *J* = 7.0 Hz, 0.63H, (M)), 4.20 – 4.10 (m, 0.63H, (M)), 3.87 – 3.82 (m, 3H, both rotamers) 3.83 – 3.77 (m, 0.37H, (m)), 3.75 (s, 2H, (M)), 3.65 (s, 1H, (m)), 3.36 – 3.17 (m, 1H, both rotamers), 3.08 – 2.69 (m, 3H, both rotamers), 2.65 – 2.51 (m, 1H, both rotamers), 1.42 (s, 3.3H, (m)), 1.30 (s, 5.7H, (M)).
^{13}C NMR (126 MHz, $CDCl_3$, major rotamer) δ 154.5, 148.0, 147.2, 137.7, 131.6(2C), 131.5(2C), 128.3, 126.8, 120.4, 111.6, 110.3, 80.0, 56.4, 56.0(2C), 42.4, 37.6, 28.3(3C), 28.2.
IR (film) ν_{max}/cm^{-1} 2973, 2933, 2835, 1686, 1612, 1517, 1486.
HRMS (ESI^+) $C_{23}H_{28}Br^{79}NO_4$ found $[M+Na]^+$ at 484.1098, expected at 484.1094.

tert-Butyl (*S*)-1-(4-bromobenzyl)-6,7-dimethoxy-3,4-dihydroisoquinoline-2(*1H*)-carboxylate ((*S*)-S1)

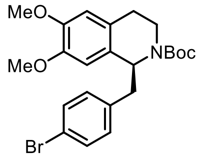

(*S*)-1-(4-Bromobenzyl)-6,7-dimethoxy-1,2,3,4-tetrahydroisoquinoline **(*S*)-8a** (52 mg, 0.14 mmol), di-*tert*-butyl dicarbonate (38 mg, 0.17 mmol) and triethylamine (24 µL, 0.17 mmol) in anhydrous CH_2Cl_2 (0.7 mL) were submitted to *General Procedure H*. Purification by flash column chromatography (pentane/EtOAc, 4:1) afforded the title compound as a colourless oil (57 mg, 0.12 mmol, 86%, 94% *ee*).

^{1}H NMR (400 MHz, $CDCl_3$, analysis revealed the presence of two rotamers present in a 0.64:0.36 ratio, Major(M) and minor(m)) δ 7.45 – 7.30 (m, 2H, both rotamers), 7.01 – 6.93 (m, both rotamers), 6.64 – 6.56 (m, 1H, both rotamers), 6.34 (s, 0.64H, (M)), 6.18 (s, 0.36H, (m)), 5.21 (t, *J* = 7.0 Hz, 0.36H, (m)), 5.05 (t, *J* = 7.0 Hz, 0.64H, (M)), 4.19 – 4.10 (m, 0.64H, (M)), 3.89 – 3.77 (m, 3H, both rotamers), 3.83 – 3.77 (m, 0.36H, (m)), 3.75 (s, 2H, (M)), 3.65 (s, 1H, (m)), 3.37 – 3.15 (m, 1H, both rotamers), 3.10 – 2.68 (m, 3H, both rotamers), 2.65 – 2.50 (m, 1H, both rotamers), 1.42 (s, 3.3H, (m)), 1.30 (m, 5.7H, (M)).
^{13}C NMR (101 MHz, $CDCl_3$, major rotamer) δ 154.5, 148.0, 147.2, 137.7, 131.6(2C), 131.5(2C), 128.3, 126.8, 120.4, 111.6, 110.6, 79.9, 56.4, 56.0(2C), 42.4, 37.3, 28.3(3C), 28.2.
IR (film) ν_{max}/cm^{-1} 2973, 2933, 1686, 1612, 1517, 1488.
HRMS (ESI^+) $C_{23}H_{28}Br^{79}NO_4$ found $[M+Na]^+$ at 484.1113, expected at 484.1118.
$[\alpha]_D^{25}$ +71.7 (c. 1.23, $CHCl_3$).

SFC Conditions Gradient from 1% to 30% MeOH in 5 min. λ = 215.0 nm; 97:3 *er* (major enantiomer t_R = 3.16 min; minor enantiomer t_R = 3.34 min), ***94% ee***.

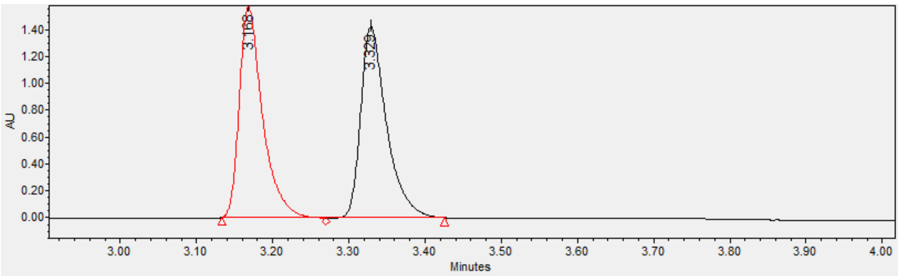

	Name	Retention Time (min)	Purity1 Angle	Purity1 Threshold	PDA/FLR Match1 Spect. Name	PDA/FLR Match1 Angle	PDA/FLR Match1 Threshold	PDA/FLR Match1 Lib. Name	Area (µV*sec)	% Area	Height (µV)	Int Type	Amount
1		3.168							3310035	49.79	1570562	bv	
2		3.329							3338141	50.21	1423018	vb	

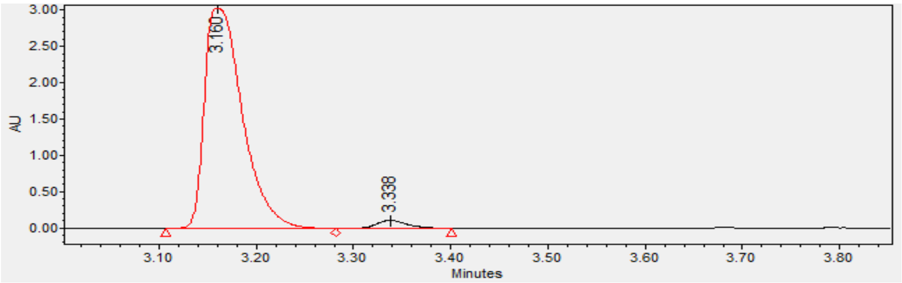

	Name	Retention Time (min)	Purity1 Angle	Purity1 Threshold	PDA/FLR Match1 Spect. Name	PDA/FLR Match1 Angle	PDA/FLR Match1 Threshold	PDA/FLR Match1 Lib. Name	Area (µV*sec)	% Area	Height (µV)	Int Type	Amount
1		3.160							8225941	97.19	3030269	bv	
2		3.338							237629	2.81	110221	vb	

***tert*-Butyl (*R*)-1-(4-bromobenzyl)-6,7-dimethoxy-3,4-dihydroisoquinoline-2(*1H*)-carboxylate ((*R*)-S1)**

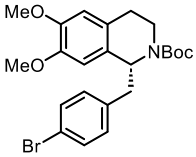

(*R*)-1-(4-Bromobenzyl)-6,7-dimethoxy-1,2,3,4-tetrahydroisoquinoline **(*R*)-8a** (14 mg, 39 µmol), di-*tert*-butyl dicarbonate (10 mg, 46 µmol) and triethylamine (6.0 µL, 46 µmol) in anhydrous CH_2Cl_2 (0.2 mL) were submitted to *General Procedure H*. Purification by flash column chromatography (pentane/EtOAc, 4:1) afforded the title compound as a colourless oil (13 mg, 27 µmol, 69%, 99% *ee*).

^{1}H NMR (500 MHz, $CDCl_3$, analysis revealed the presence of two rotamers present in a 0.64:0.36 ratio, Major(M) and minor(m)) δ 7.43 – 7.33 (m, 2H, both rotamers), 7.01 – 6.94 (m, 2H, both rotamers), 6.63 – 6.65 (m, 1H, both rotamers), 6.34 (s, 0.64H, (M)), 6.18 (s, 0.36H, (m)), 5.21 (t, *J* = 7.0 Hz, 0.36H, (m)), 5.06 (t, *J* = 7.0 Hz, 0.64H, (M)), 4.19 – 4.12 (m, 0.64H, (M)), 3.88 – 3.83 (m, 3H, both rotamers), 3.83 – 3.77 (m, 0.36H, (m)), 3.75 (s, 2H, (M)), 3.65 (s, 1H, (m)), 3.36 – 3.16 (m, 1H, both rotamers), 3.08 – 2.69 (m, 3H, both rotamers), 2.65 – 2.52 (m, 1H, both rotamers), 1.42 (s, 3.3H, (m)), 1.30 (s, 5.7H, (M)).
^{13}C NMR (126 MHz, $CDCl_3$, major rotamer) δ 154.5, 148.0, 147.2, 137.7, 131.6(2C), 131.5(2C), 128.3, 126.9, 120.5, 111.6, 110.3, 80.0, 56.3, 56.0(2C), 42.5, 37.4, 28.4(3C), 28.2.
IR (film) ν_{max}/cm^{-1} 2973, 2933, 1687, 1612, 1518, 1488.
HRMS (ESI$^+$) $C_{23}H_{28}Br^{79}NO_4$ found $[M+Na]^+$ at 484.1095, expected at 484.1118.
$[\alpha]_D^{25}$ –73.8 (c. 0.89, $CHCl_3$).
SFC Conditions Gradient from 1% to 30% MeOH in 5 min. λ = 215.0 nm; >99:1 *er* (major enantiomer t_R = 3.36 min; minor enantiomer t_R = 3.20 min), ***99% ee***.

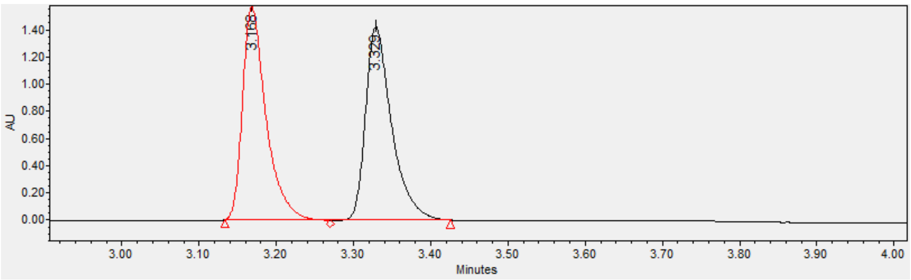

	Name	Retention Time (min)	Purity1 Angle	Purity1 Threshold	PDA/FLR Match1 Spect. Name	PDA/FLR Match1 Angle	PDA/FLR Match1 Threshold	PDA/FLR Match1 Lib. Name	Area (µV*sec)	% Area	Height (µV)	Int Type	Amount
1		3.168							3310035	49.79	1570562	bv	
2		3.329							3338141	50.21	1423018	vb	

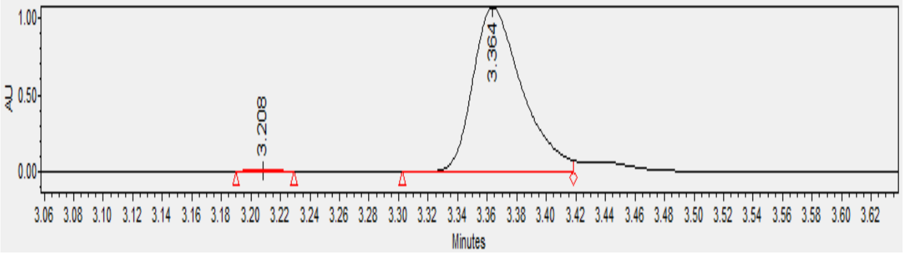

	Name	Retention Time (min)	Purity1 Angle	Purity1 Threshold	PDA/FLR Match1 Spect. Name	PDA/FLR Match1 Angle	PDA/FLR Match1 Threshold	PDA/FLR Match1 Lib. Name	Area (µV*sec)	% Area	Height (µV)	Int Type	Amount
1		3.208							10288	0.41	7929	bb	
2		3.364							2492900	99.59	1078836	bv	

***tert*-Butyl 1-(3-(benzyloxy)-4-methoxybenzyl)-6,7-dimethoxy-3,4-dihydroisoquinoline-2(*1H*)-carboxylate (rac-S2)**

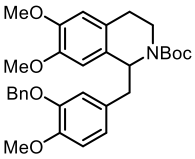

1-(3-(Benzyloxy)-4-methoxybenzyl)-6,7-dimethoxy-1,2,3,4-tetrahydroisoquinoline **rac-8b** (60 mg, 0.14 mmol), di-*tert*-butyl dicarbonate (37 mg, 0.17 mmol) and triethylamine (24 µL, 0.17 mmol) in anhydrous CH_2Cl_2 (0.7 mL) were submitted to **General Procedure H**. Purification by flash column chromatography (pentane/EtOAc, 1:1) afforded the title compound as a colourless oil (40 mg, 76 µmol, 55%).

^{1}H NMR (400 MHz, $CDCl_3$, analysis revealed the presence of two rotamers present in a 0.64:0.36 ratio, Major(M) and minor(m)) δ 7.44 – 7.39 (m, 2H, both rotamers), 7.39 – 7.32 (m, 2H, both rotamers), 7.31 – 7.26 (m, 1H, both rotamers), 6.84 – 6.72 (m, 1H, both rotamers), 6.68 – 6.54 (m, 3H, both rotamers), 6.27 (s, 0.64H, (M)), 6.19 (s, 0.36H, (m)), 5.22 (t, J = 6.7 Hz, 0.36H, (m)), 5.24 – 4.97 (m, 2H, both rotamers + 0.64H, (M)), 4.13 – 4.05 (m, 0.64H, (M)), 3.88 – 3.85 (m, 6H, both rotamers), 3.79 – 3.72 (m, 0.36H, (m)), 3.70 (s, 2H, (M)), 3.62 (s, 1H, (m)), 3.27 – 3.12 (m, 1H, both rotamers), 3.05 – 2.66 (m, 3H, both rotamers), 2.61 – 2.42 (m, 1H, both rotamers), 1.46 (s, 3.3H, (m)), 1.37 (s, 5.7H, (M)).
^{13}C NMR (101 MHz, $CDCl_3$, major rotamer) δ 154.6, 148.6, 148.2, 147.8, 147.0, 137.3, 131.2, 128.6(3C), 127.9, 127.4(2C), 126.8, 122.7, 115.9, 112.0, 111.4, 110.5, 79.7, 71.2, 56.6, 56.3, 56.0(2C), 42.5, 37.5, 28.4(3C), 28.3.
IR (film) ν_{max}/cm^{-1} 2972, 2934, 2836, 1684, 1611, 1590, 1515.
HRMS (ESI$^+$) $C_{31}H_{37}NO_6$ found $[M+Na]^+$ at 542.2508, expected at 542.2513.

tert-Butyl (_S_)-1-(3-(benzyloxy)-4-methoxybenzyl)-6,7-dimethoxy-3,4-dihydroisoquinoline-2(_1H_)-carboxylate ((_S_)-S2)

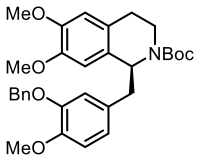

(_S_)-1-(3-(Benzyloxy)-4-methoxybenzyl)-6,7-dimethoxy-1,2,3,4-tetrahydroisoquinoline **(_S_)-8b** (0.12 g, 0.29 mmol), di-_tert_-butyl dicarbonate (76 mg, 0.35 mmol) and triethylamine (49 µL, 0.35 mmol) in anhydrous CH_2Cl_2 (1.5 mL) were submitted to **General Procedure H**. Purification by flash column chromatography (pentane/EtOAc, 1:1) afforded the title compound as a colourless oil (0.11 g, 0.20 mmol, 70%, 94% _ee_).

^{1}H NMR (600 MHz, $CDCl_3$, analysis revealed the presence of two rotamers present in a 0.64:0.36 ratio, Major(M) and minor(m)) δ 7.45 – 7.39 (m, 2H, both rotamers), 7.38 – 7.33 (m, 2H, both rotamers), 7.31 – 7.26 (m, 1H, both rotamers), 6.80 (d, J = 8.1 Hz, 0.64H, (M)), 6.76 (d, J = 8.1 Hz, 0.36H, (m)), 6.67 – 6.55 (m, 3H, both rotamers), 6.26 (s, 0.64H, (M)), 6.23 (s, 0.36H, (m)), 5.21 (t, J = 6.8 Hz, 0.36H, (m)), 5.10 – 4.98 (m, 2H, both rotamers + 0.64H, (M)), 4.12 – 4.04 (m, 0.64H, (M)), 3.88 – 3.81 (m, 6H, both rotamers), 3.77 – 3.74 (m, 0.36H, (m)), 3.70 (s, 2H, (M)), 3.62 (s, 1H, (m)), 3.25 – 3.14 (m, 1H, both rotamers), 3.05 – 2.67 (m, 3H, both rotamers), 2.59 – 2.45 (m, 1H, both rotamers), 1.46 (s, 3.3H, (m)), 1.36 (s, 5.7H, (M)).
^{13}C NMR (151 MHz, $CDCl_3$, major rotamer) δ 154.6, 148.6, 148.2, 147.8, 147.1, 137.3, 131.3, 128.6(3C), 127.9, 127.4(2C), 126.8, 122.7, 115.9, 112.1, 111.5, 110.5, 79.7, 71.3, 56.6, 56.3, 56.1(2C), 42.5, 37.6, 28.5(3C), 28.3.
IR (film) ν_{max}/cm^{-1} 2971, 2933, 2835, 1686, 1610, 1590, 1515.
HRMS (ESI$^+$) $C_{31}H_{37}NO_6$ found $[M+Na]^+$ at 542.2514, expected at 542.2513.
$[\alpha]_D^{25}$ +45.7 (c. 1.17, $CHCl_3$).
SFC Conditions Gradient from 1% to 30% MeOH in 5 min. λ = 215.0 nm; 97:3 _er_ (major enantiomer t_R = 3.97 min; minor enantiomer t_R = 4.31 min), **94% _ee_**.

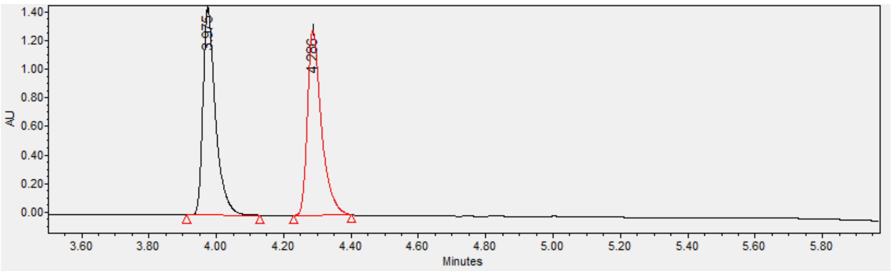

	Name	Retention Time (min)	Purity1 Angle	Purity1 Threshold	PDA/FLR Match1 Spect. Name	PDA/FLR Match1 Angle	PDA/FLR Match1 Threshold	PDA/FLR Match1 Lib. Name	Area (µV*sec)	% Area	Height (µV)	Int Type	Amount
1		3.975							3734697	50.00	1452542	bb	
2		4.286							3734271	50.00	1291503	bb	

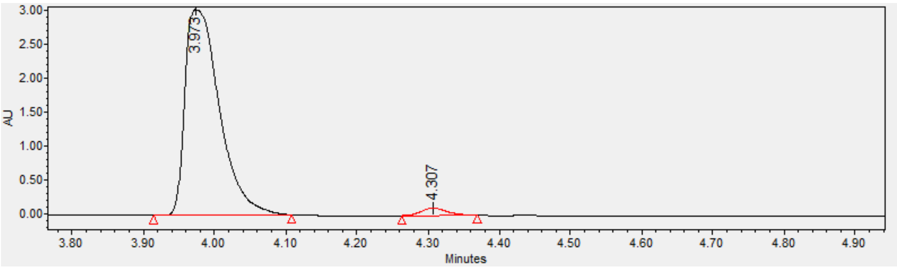

	Name	Retention Time (min)	Purity1 Angle	Purity1 Threshold	PDA/FLR Match1 Spect. Name	PDA/FLR Match1 Angle	PDA/FLR Match1 Threshold	PDA/FLR Match1 Lib. Name	Area (µV*sec)	% Area	Height (µV)	Int Type	Am
1		3.973							10222212	97.39	3027015	bb	
2		4.307							274377	2.61	109575	bb	

***tert*-Butyl (*S*)-1-(3-(benzyloxy)-4-methoxybenzyl)-6,7-dimethoxy-3,4-dihydroisoquinoline-2(*1H*)-carboxylate ((*R*)-S6)**

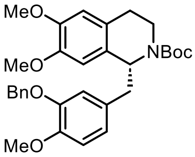

(*R*)-1-(3-(Benzyloxy)-4-methoxybenzyl)-6,7-dimethoxy-1,2,3,4-tetrahydroisoquinoline **(*R*)-8b** (12 mg, 28 µmol), di-*tert*-butyl dicarbonate (7.3 mg, 34 µmol) and triethylamine (5.0 µL, 34 µmol) in anhydrous CH_2Cl_2 (0.14 mL) were submitted to **General Procedure H**. Purification by flash column chromatography (pentane/EtOAc, 1:1) afforded the title compound as a colourless oil (7.4 mg, 14 µmol, 51%, 96% *ee*).

^{1}H NMR (600 MHz, $CDCl_3$, analysis revealed the presence of two rotamers present in a 0.64:0.36 ratio, Major(M) and minor(m)) δ 7.43 – 7.49 (m, 2H, both rotamers), 7.38 – 7.31 (m, 2H, both rotamers), 7.31 – 7.27 (m, 1H, both rotamers), 6.80 (d, *J* = 8.2 Hz, 0.64H, (M)), 6.76 (d, J = 8.2 Hz, 0.36H, (m)), 6.68 – 6.49 (m, 3H, both rotamers), 6.26 (s, 0.64H, (M)), 6.19 (s, 0.36H, (m)), 5.21 (t, *J* = 6.7 Hz, 0.36H, (m)), 5.10 – 4.98 (m, 2H, both rotamers + 0.64H, (M)), 4.13 – 4.06 (m, 0.64H, (M)), 3.86 – 3.81 (m, 6H, both rotamers), 3.79 – 3.71 (m, 0.36H, (m)), 3.70 (s, 2H, (M)), 3.62 (s, 1H, (m)), 3.26 – 3.13 (m, 1H, both rotamers), 3.05 – 2.67 (m, 3H, both rotamers), 2.61 – 2.44 (m, 1H, both rotamers), 1.46 (s, 3.3H, (m)), 1.37 (s, 5.7H, (M)).
^{13}C NMR (151 MHz, $CDCl_3$, major rotamer) δ 154.7, 148.7, 148.2, 147.9, 147.1, 137.3, 131.3, 128.7(3C), 128.0, 127.4(2C), 126.9, 122.8, 116.0, 112.1, 111.5, 110.5, 79.8, 71.3, 56.6, 56.4, 56.0(2C), 42.6, 37.6, 28.4(3C), 28.3.
IR (film) ν_{max}/cm^{-1} 2979, 2903, 2834, 1684, 1647, 1590, 1516.
HRMS (ESI$^+$) $C_{31}H_{37}NO_6$ found $[M+Na]^+$ at 542.2510, expected at 542.2513.
$[\alpha]_D^{25}$ –47.4 (c. 0.76, $CHCl_3$).
SFC Conditions Gradient from 1% to 30% MeOH in 5 min. λ = 215.0 nm; 98:2 *er* (major enantiomer t_R = 4.33 min; minor enantiomer t_R = 4.02 min), **96% *ee***.

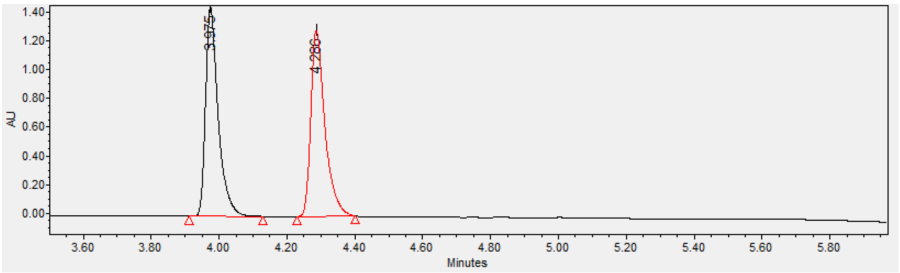

	Name	Retention Time (min)	Purity1 Angle	Purity1 Threshold	PDA/FLR Match1 Spect. Name	PDA/FLR Match1 Angle	PDA/FLR Match1 Threshold	PDA/FLR Match1 Lib. Name	Area (µV*sec)	% Area	Height (µV)	Int Type	Amount
1		3.975							3734697	50.00	1452542	bb	
2		4.286							3734271	50.00	1291503	bb	

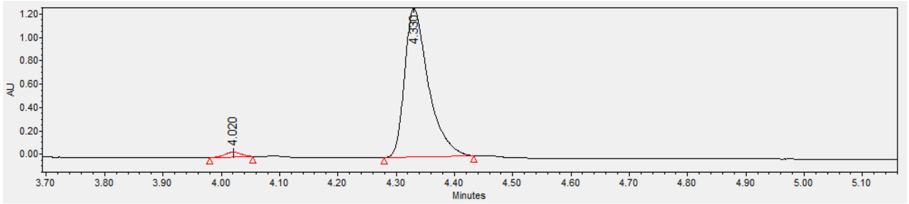

	Name	Retention Time (min)	Purity1 Angle	Purity1 Threshold	PDA/FLR Match1 Spect. Name	PDA/FLR Match1 Angle	PDA/FLR Match1 Threshold	PDA/FLR Match1 Lib. Name	Area (µV*sec)	% Area	Height (µV)	Int Type	Amount	Units	Peak Type	Peak Codes
1		4.020							76744	2.08	39614	bb			Unknown	
2		4.330							3606961	97.92	1271252	bb			Unknown	

***tert*-Butyl 1-(3-bromo-4-((4-methoxybenzyl)oxy)benzyl)-6,7-dimethoxy-3,4-dihydroisoquinoline-2(*1H*)-carboxylate (rac-S3)**

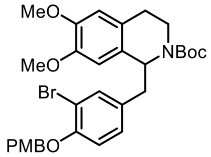

1-(3-Bromo-4-((4-methoxybenzyl)oxy)benzyl)-6,7-dimethoxy-1,2,3,4-tetrahydroisoquinoline **rac-8c** (70 mg, 0.14 mmol), di-*tert*-butyl dicarbonate (37 mg, 0.17 µmol) and triethylamine (24 µL, 0.17 µmol) in anhydrous CH_2Cl_2 (0.7 mL) were submitted to **General Procedure H**. Purification by flash column chromatography (pentane/Et_2O, 1:1) afforded the title compound as a colourless oil (60 mg, 99 µmol, 71%).

^{1}H NMR (500 MHz, $CDCl_3$, analysis revealed the presence of two rotamers present in a 0.66:034 ratio, Major(M) and minor(m)) δ 7.42 – 7.27 (m, 3H, both rotamers), 7.00 – 6.87 (m, 3H, both rotamers), 6.86 – 6.79 (m, 1H, both rotamers), 6.64 – 4.57 (m, 1H, both rotamers), 6.38 (s, 0.66H, (M)) 6.22 (s, 0.34H, (m)), 5.18 (t, *J* = 7.0 Hz, (m)), 5.08 – 5.02 (m, 2H, both rotamers + 0.66H, (M)), 4.21 – 4.16 (m, 0.66H, (M)), 3.87 – 3.83 (m, 3H, both rotamers + 0.34H, (m)), 3.81 (s, 3H, both rotamers), 3.78 – 3.60 (m, 3H, both rotamers), 3.35 – 3.18 (m, 1H, both rotamers), 3.04 – 2.70 (m, 3H, both rotamers), 2.65 – 2.51 (m, 1H, both rotamers), 1.43 (s, 3.3H, (m)), 1.30 (s, 5.7H, (M)).
^{13}C NMR (126 MHz, $CDCl_3$, major rotamer) δ 159.5, 154.5, 153.9, 147.9, 147.2, 134.4, 132.8, 129.7, 128.8, 128.7(2C), 128.5, 126.7, 114.1(2C), 114.0, 112.5, 111.6, 110.2, 79.9, 70.9, 56.4, 55.9(2C), 55.4, 41.9, 37.1, 28.3(3C), 28.2.
IR (film) ν_{max}/cm^{-1} 3005, 2934, 2836, 1684, 1613, 1516, 1494, 1246, 1172, 910, 731.
HRMS (ESI$^+$) $C_{31}H_{36}Br^{79}NO_6$ found $[M+H]^+$ at 598.1782, expected at 598.1799.

***tert*-Butyl (*S*)-1-(3-bromo-4-((4-methoxybenzyl)oxy)benzyl)-6,7-dimethoxy-3,4-dihydroisoquinoline-2(*1H*)-carboxylate ((*S*)-S3)**

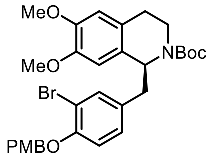

(*S*)-1-(3-Bromo-4-((4-methoxybenzyl)oxy)benzyl)-6,7-dimethoxy-1,2,3,4-tetrahydroisoquinoline **(*S*)-8c** (17 mg, 35 µmol), di-*tert*-butyl dicarbonate (9.0 mg, 41 µmol) and triethylamine (5.8 µL, 41 µmol) in anhydrous CH_2Cl_2 (0.2 mL) were submitted to **General Procedure H**. Purification by flash column chromatography (pentane/Et_2O, 1:1) afforded the title compound as a colourless oil (8.1 mg, 14 µmol, 39%, 96% *ee*).

^{1}H NMR (500 MHz, $CDCl_3$, analysis revealed the presence of two rotamers present in a 0.66:034 ratio, Major(M) and minor(m)) δ 7.42 – 7.27 (m, 3H, both rotamers), 7.00 – 6.88 (m, 3H, both rotamers), 6.86 – 6.80 (m, 1H, both rotamers), 6.62 – 6.54 (m, 1H, both rotamers), 6.38 (s, 0.66H, (M)), 6.22 (s, 0.34H, (m)), 5.18 (t, *J* = 7.0 Hz, 0.34H, (m)), 5.08 – 5.01 (m, 2H, both rotamers + 0.66H, (M)), 4.21 – 4.14 (m, 0.66H, (M)), 3.87 – 3.83 (m, 3H, both rotamers), 3.83 – 3.78 (m, 3H, both rotamers + 0.34H, (m)), 3.75 – 3.61 (m, 3H, both rotamers), 3.34 – 3.19 (m, 1H, both rotamers), 3.04 – 2.70 (m, 3H, both rotamers), 2.65 – 2.51 (m, 1H, both rotamers), 1.43 (s, 3.3H, (m)), 1.30 (m, 5.7H, (M)).
^{13}C NMR (126 MHz, $CDCl_3$, major rotamer) δ 159.5, 154.5, 153.9, 148.0, 147.3, 134.4, 132.9, 129.7, 128.9, 128.8(2C), 128.6, 126.7, 114.13(2C), 114.08, 112.6, 111.6, 110.3, 79.9, 71.0, 56.5, 56.1(2C), 55.4, 42.0, 37.2, 28.4(3C), 28.2.
IR (film) ν_{max}/cm^{-1} 3001, 2932, 2837, 1686, 1613, 1516, 1494, 1246, 1172.
HRMS (ESI$^+$) $C_{31}H_{36}Br^{79}NO_6$ found $[M+H]^+$ at 598.1798, expected at 598.1799.
$[\alpha]_D^{25}$ +69.4 (c. 1.16, $CHCl_3$).

SFC Conditions Gradient from 1% to 30% MeOH in 5 min then hold 30% MeOH for 2 min. λ = 215.0 nm; 98:2 *er* (major enantiomer t_R = 5.07 min; minor enantiomer t_R = 5.29 min), ***96% ee***.

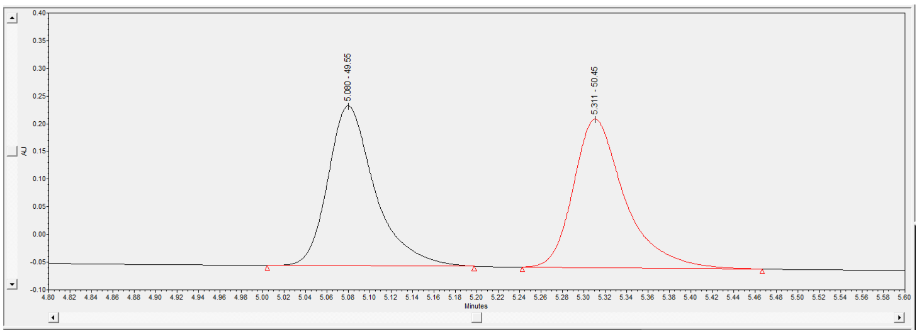

	Name	Retention Time (min)	Purity1 Angle	Purity1 Threshold	PDA/FLR Match1 Spect. Name	PDA/FLR Match1 Angle	PDA/FLR Match1 Threshold	PDA/FLR Match1 Lib. Name	Area (µV*sec)	% Area	Height (µV)	Int Type	Amount	Units	Peak Type	Peak Codes
1		5.080							880946	49.55	288922	bb			Unknown	
2		5.311							896995	50.45	268473	bb			Unknown	

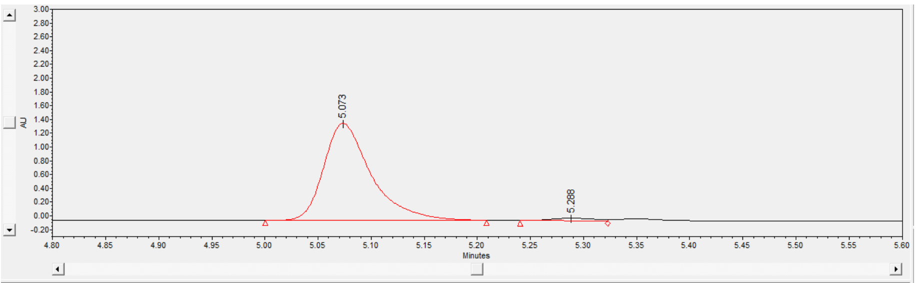

	Name	Retention Time (min)	Purity1 Angle	Purity1 Threshold	PDA/FLR Match1 Spect. Name	PDA/FLR Match1 Angle	PDA/FLR Match1 Threshold	PDA/FLR Match1 Lib. Name	Area (µV*sec)	% Area	Height (µV)	Int Type	Amount	Units	Peak Type	Peak Codes
1		5.073							4378056	97.78	1408017	bb			Unknown	
2		5.288							99523	2.22	35977	bv			Unknown	

***tert*-Butyl (*R*)-1-(3-bromo-4-((4-methoxybenzyl)oxy)benzyl)-6,7-dimethoxy-3,4-dihydroisoquinoline-2(*1H*)-carboxylate ((*R*)-S3)**

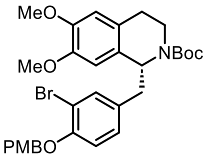

(*R*)-1-(3-Bromo-4-((4-methoxybenzyl)oxy)benzyl)-6,7-dimethoxy-1,2,3,4-tetrahydroisoquinoline **(*R*)-8c** (13 mg, 26 µmol), di-*tert*-butyl dicarbonate (6.7 mg, 31 µmol) and triethylamine (4.3 µL, 31 µmol) in anhydrous CH_2Cl_2 (0.13 mL) were submitted to **General Procedure H**. Purification by flash column chromatography (pentane/Et_2O, 1:1) afforded the title compound as a colourless oil (13 mg, 22 µmol, 85%, 92% *ee*).

^{1}H NMR (500 MHz, $CDCl_3$, analysis revealed the presence of two rotamers present in a 0.64:036 ratio, Major(M) and minor(m)) δ 7.44 – 7.27 (m, 3H, both rotamers), 6.99 – 6.88 (m, 3H, both rotamers), 6.86 – 6.78 (m, 1H, both rotamers) 6.63 – 6.57 (m, 1H, both rotamers), 6.37 (s, 0.64H, (M)), 6.22 (s, 0.34H, (m)), 5.18 (t, *J* = 7.0 Hz, 0.36H, (m)), 5.08 – 4.99 (m, 2H, both rotamers + 0.64H, (M)), 4.21 – 4.14 (m, 0.64H, (M)), 3.88 – 3.83 (m, 3H, both rotamers), 3.83 – 3.77 (m, 3H, both rotamers + 0.36H, (m)), 3.75 (s, 2H, (M)), 3.64 (s, 1H, (m)), 3.37 – 3.16 (m, 1H, both rotamers), 3.04 – 2.70 (m, 3H, both rotamers), 2.64 – 2.51 (m, 1H, both rotamers), 1.43 (s, 3.3H, (m)), 1.30 (s, 5.7H, (M)).
^{13}C NMR (126 MHz, $CDCl_3$, major rotamer) δ 159.6, 154.5, 153.9, 148.0, 147.3, 134.4, 132.9, 129.7, 128.9, 128.8(2C), 128.6, 126.7, 114.14(2C), 114.10, 112.6, 111.7, 110.3, 79.9, 71.0, 56.5, 56.1(2C), 55.5, 42.0, 37.2, 28.4(3C), 28.3.
IR (film) ν_{max}/cm^{-1} 3005, 2930, 2837, 1687, 1613, 1516, 1494, 1246, 1172.
HRMS (ESI$^+$) $C_{31}H_{36}Br^{79}NO_6$ found $[M+H]^+$ at 598.1797, expected at 598.1799.

[α]$_D^{25}$ –66.4 (c. 0.96, $CHCl_3$).

SFC Conditions Gradient from 1% to 30% MeOH in 5 min then hold 30% MeOH for 2 min. λ = 215.0 nm; 96:4 *er* (major enantiomer t_R = 5.30 min; minor enantiomer t_R = 5.08 min), **92% *ee***.

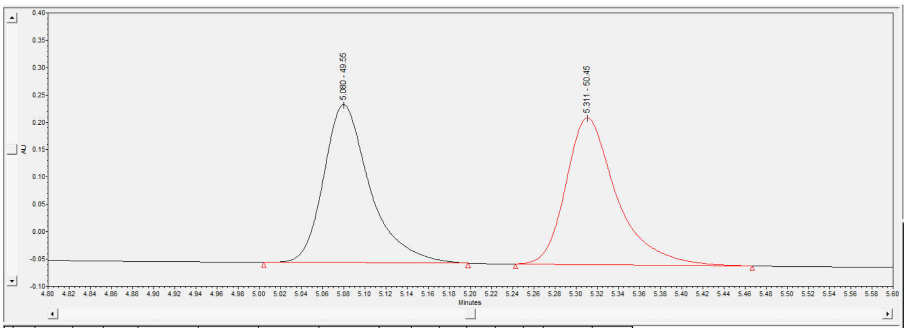

	Name	Retention Time (min)	Purity1 Angle	Purity1 Threshold	PDA/FLR Match1 Spect. Name	PDA/FLR Match1 Angle	PDA/FLR Match1 Threshold	PDA/FLR Match1 Lib. Name	Area (µV*sec)	% Area	Height (µV)	Int Type	Amount	Units	Peak Type	Peak Codes
1		5.080							880946	49.55	288922	bb			Unknown	
2		5.311							896995	50.45	268473	bb			Unknown	

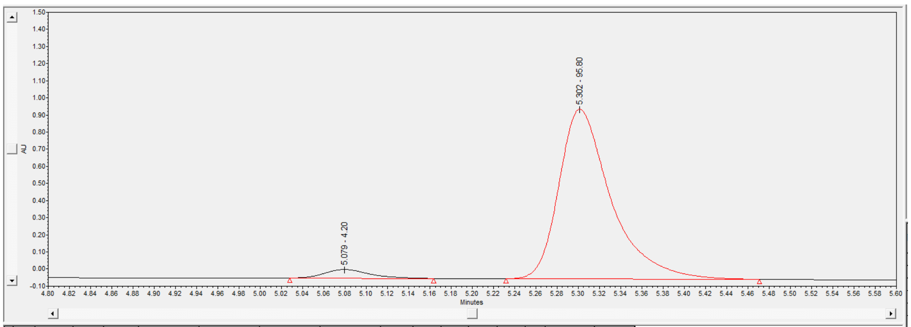

	Name	Retention Time (min)	Purity1 Angle	Purity1 Threshold	PDA/FLR Match1 Spect. Name	PDA/FLR Match1 Angle	PDA/FLR Match1 Threshold	PDA/FLR Match1 Lib. Name	Area (µV*sec)	% Area	Height (µV)	Int Type	Amount	Units	Peak Type	Peak Codes
1		5.079							148956	4.20	50977	bb			Unknown	
2		5.302							3396395	95.80	992624	bb			Unknown	

Methyl-1-(4-(benzyloxy)benzyl)-6,7-dimethoxy-3,4-dihydroisoquinoline-2(*1H*)-carboxylate (rac-S4)

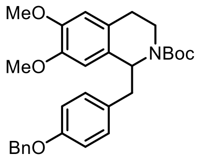

1-(4-(Benzyloxy)benzyl)-6,7-dimethoxy-1,2,3,4-tetrahydroisoquinoline **rac-8d** (24 mg, 61 µmol), di-*tert*-butyl dicarbonate (16 mg, 73 µmol) and triethylamine (10 µL, 73 µmol) in anhydrous CH_2Cl_2 (0.30 mL) were submitted to **General Procedure H**. Purification by flash column chromatography (pentane/Et_2O, 1:1) afforded the title compound as a colourless oil (25 mg, 51 µmol, 84%).

^{1}H NMR (500 MHz, $CDCl_3$, analysis revealed the presence of two rotamers present in a 0.64:0.36 ratio, Major(M) and minor(m)) δ 7.43 – 7.39 (m, 2H, both rotamers), 7.39 – 7.35 (m, 2H, both rotamers), 7.34 – 7.29 (m, 1H, both rotamers), 7.05 – 6.96 (m, 2H, both rotamers), 6.92 – 6.83 (m, 2H, both rotamers), 6.62 – 6.59 (m, 1H, both rotamers), 6.32 (s, 0.64H, (M)), 6.17 (s, 0.34H, (m)), 5.20 (t, *J* = 6.9 Hz, 0.34H, (m)), 5.08 – 4.99 (m, 2H, both rotamers + 0.64H, (M)), 4.19 – 4.12 (m, 0.64H, (M)), 3.88 – 3.77 (m, 3H, both rotamers + 0.34H, (m)), 3.70 (s, 2H, (M)), 3.60 (s, 1H, (m)), 3.36 – 3.20 (m, 1H, both rotamers), 3.10 – 2.98 (m, 1H, both rotamers), 2.94 – 2.70 (m, 2H, both rotamers), 2.66 – 2.52 (m, 1H, both rotamers), 1.43 (s, 3.3H, (m)), 1.30 (s, 5.7H, (M)).

^{13}C NMR (126 MHz, $CDCl_3$, major rotamer) δ 157.6, 154.6, 147.8, 147.1, 137.3, 131.1, 131.0, 130.8(2C), 128.7(2C), 128.1, 127.5(2C), 126.7, 114.9(2C), 111.5, 110.4, 79.7, 70.2, 56.6, 56.00, 55.95, 42.2, 37.4, 28.4(3C), 28.3.

Spectroscopic data is in agreement with that reported previously.[25]

Methyl (*S*)-1-(4-(benzyloxy)benzyl)-6,7-dimethoxy-3,4-dihydroisoquinoline-2(*1H*)-carboxylate ((*S*)-S4)

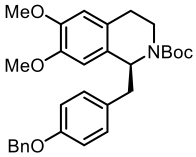

(*S*)-1-(4-(Benzyloxy)benzyl)-6,7-dimethoxy-1,2,3,4-tetrahydroisoquinoline **(*S*)-8d** (16 mg, 41 µmol), di-*tert*-butyl dicarbonate (11 mg, 49 µmol) and triethylamine (6.8 µL, 49 µmol) in anhydrous CH_2Cl_2 (0.2 mL) were submitted to **General Procedure H**. Purification by flash column chromatography (pentane/Et_2O, 1:1) afforded the title compound as a colourless oil (18 mg, 37 µmol, 90%, 96% *ee*).

^{1}H NMR (500 MHz, $CDCl_3$, analysis revealed the presence of two rotamers present in a 0.64:0.36 ratio, Major(M) and minor(m)) δ 7.44 – 7.28 (m, 5H, both rotamers), 7.05 – 6.96 (m, 2H, both rotamers), 6.92 – 6.83 (m, 2H, both rotamers), 6.62 – 6.59 (m, 1H, both rotamers), 6.32 (s, 0.64H, (M)), 6.17 (s, 0.34H, (m)), 5.20 (t, *J* = 6.9 Hz, 0.34H, (m)), 5.08 – 5.01 (m, 2H, both rotamers + 0.64H, (M)), 4.20 – 4.13 (m, 0.64H, (M)), 3.89 – 3.86 (m, 3H, both rotamers + 0.34H, (m)), 3.71 (s, 2H, (M)), 3.60 (s, 1H, (m)), 3.36 – 3.20 (m, 1H, both rotamers), 3.10 – 2.98 (m, 1H, both rotamers), 2.94 – 2.70 (m, 2H, both rotamers), 2.66 – 2.52 (m, 1H, both rotamers), 1.44 (s, 3.3H, (m)), 1.31 (s, 5.7H, (M)).
^{13}C NMR (126 MHz, $CDCl_3$, major rotamer) δ 157.7, 154.7, 147.8, 147.1, 137.3, 131.2, 131.0, 130.8(2C), 128.7(2C), 128.1, 127.5(2C), 126.8, 114.9(2C), 111.5, 110.5, 79.8, 70.2, 56.7, 56.02, 55.97, 42.2, 37.4, 28.4(3C), 28.3.
$[\alpha]_D^{25}$ +59.5 (c. 1.16, $CHCl_3$).
SFC Conditions Gradient from 1% to 30% MeOH in 5 min. λ = 215.0 nm; 98:2 *er* (major enantiomer t_R = 4.19 min; minor enantiomer t_R = 4.47 min), 96% *ee.*
Spectroscopic data is in agreement with that reported previously.[25]
Note: Spectroscopic data is reported for **rac-S4**.

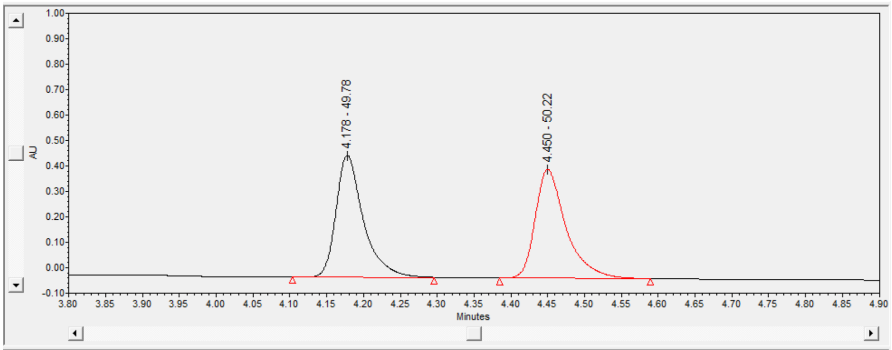

	Name	Retention Time (min)	Purity1 Angle	Purity1 Threshold	PDA/FLR Match1 Spect. Name	PDA/FLR Match1 Angle	PDA/FLR Match1 Threshold	PDA/FLR Match1 Lib. Name	Area (µV*sec)	% Area	Height (µV)	Int Type	Amount	Units	Peak Type	Peak
1		4.178							1234387	49.78	479097	bb			Unknown	
2		4.450							1245064	50.22	428800	bb			Unknown	

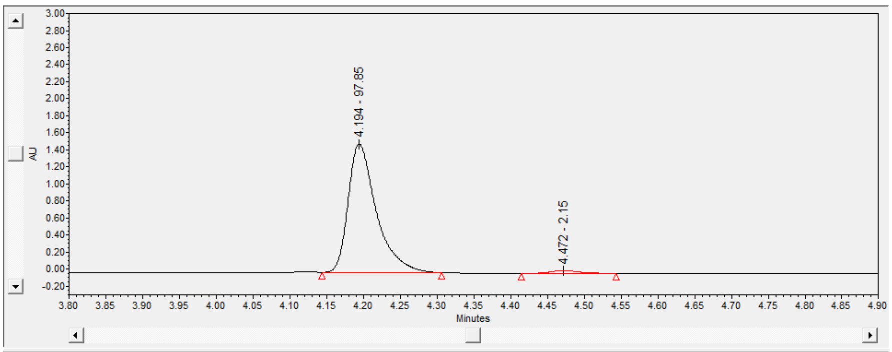

	Name	Retention Time (min)	Purity1 Angle	Purity1 Threshold	PDA/FLR Match1 Spect. Name	PDA/FLR Match1 Angle	PDA/FLR Match1 Threshold	PDA/FLR Match1 Lib. Name	Area (µV*sec)	% Area	Height (µV)	Int Type	Amount	Units	Peak Type	P
1		4.194							4011250	97.85	1510175	bb			Unknown	
2		4.472							88338	2.15	33229	bb			Unknown	

Methyl (*R*)-1-(4-(benzyloxy)benzyl)-6,7-dimethoxy-3,4-dihydroisoquinoline-2(*1H*)-carboxylate ((*R*)-S4)

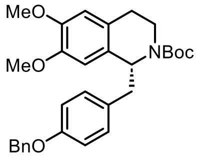

(*R*)-1-(4-(Benzyloxy)benzyl)-6,7-dimethoxy-1,2,3,4-tetrahydroisoquinoline **(*R*)-8d** (9.8 mg, 25 µmol), di-*tert*-butyl dicarbonate (6.5 mg, 30 µmol) and triethylamine (4.2 µL, 30 µmol) in anhydrous CH_2Cl_2 (0.2 mL) were submitted to **General Procedure H**. Purification by flash column chromatography (pentane/Et_2O, 1:1) afforded the title compound as a colourless oil (9.3 mg, 19 µmol, 76%, 94% *ee*).

^{1}H NMR (500 MHz, $CDCl_3$, analysis revealed the presence of two rotamers present in a 0.64:0.36 ratio, Major(M) and minor(m)) δ 7.44 – 7.29 (m, 5H, both rotamers), 7.05 – 6.96 (m, 2H, both rotamers), 6.92 – 6.83 (m, 2H, both rotamers), 6.62 – 6.54 (m, 1H, both rotamers), 6.32 (s, 0.64H, (M)), 6.17 (s, 0.34H, (m)), 5.20 (t, *J* = 6.9 Hz, 0.34H, (m)), 5.07 – 5.00 (m, *2H,* both rotamers + 0.64H, (M)), 4.20 – 4.11 (m, 0.64H, (M)), 3.89 – 3.77 (m, 3H, both rotamers + 0.34H, (m)), 3.70 (s, 2H, (M)), 3.59 (s, 1H, (m)), 3.36 – 3.20 (m, 1H, both rotamers), 3.10 – 2.98 (m, 1H, both rotamers), 2.94 – 2.70 (m, 2H, both rotamers), 2.66 – 2.52 (m, 1H, both rotamers), 1.43 (s, 3.3H, (m)), 1.30 (s, 5.7H, (M)).
^{13}C NMR (126 MHz, $CDCl_3$, major rotamer) δ 157.7, 154.7, 147.8, 147.1, 137.3, 131.2, 131.0, 130.8(2C), 128.7(2C), 128.1, 127.5(2C), 126.8, 114.9(2C), 111.5, 110.5, 79.8, 70.2, 56.7, 56.02, 56.97, 42.2, 37.4, 28.4(3C), 28.3.
$[\alpha]_D^{25}$ –58.9 (c. 0.36, $CHCl_3$).
SFC Conditions Gradient from 1% to 30% MeOH in 5 min. λ = 215.0 nm; 97:3 *er* (major enantiomer t_R = 4.44 min; minor enantiomer t_R = 4.20 min), ***94% ee***.
Spectroscopic data is in agreement with that reported previously.[25]
Note: Spectroscopic data is reported for **rac-S4**.

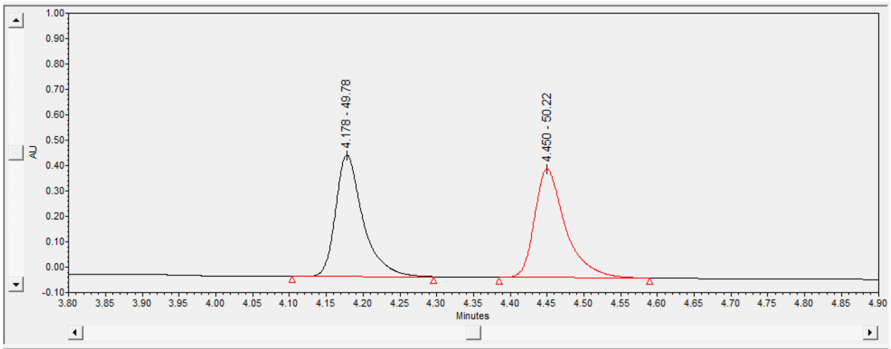

	Name	Retention Time (min)	Purity1 Angle	Purity1 Threshold	PDA/FLR Match1 Spect. Name	PDA/FLR Match1 Angle	PDA/FLR Match1 Threshold	PDA/FLR Match1 Lib. Name	Area (µV*sec)	% Area	Height (µV)	Int Type	Amount	Units	Peak Type	Peak
1		4.178							1234387	49.78	479097	bb			Unknown	
2		4.450							1245064	50.22	428800	bb			Unknown	

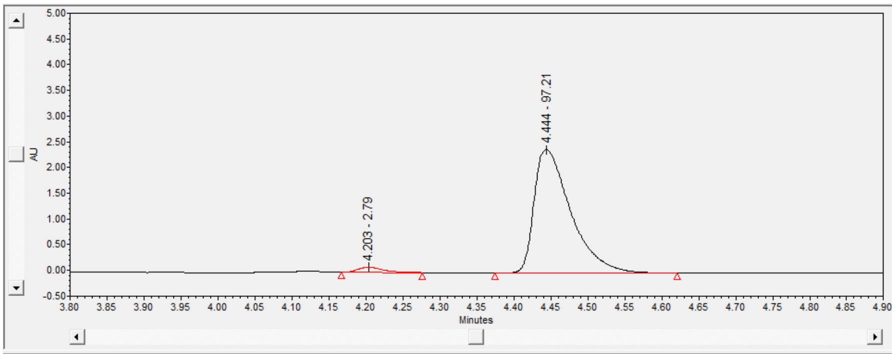

	Name	Retention Time (min)	Purity1 Angle	Purity1 Threshold	PDA/FLR Match1 Spect. Name	PDA/FLR Match1 Angle	PDA/FLR Match1 Threshold	PDA/FLR Match1 Lib. Name	Area (µV*sec)	% Area	Height (µV)	Int Type	Amount	Units	Peak Type
1		4.203							237441	2.79	100363	bb			Unknown
2		4.444							8281538	97.21	2396901	bb			Unknown

6. NMR Spectra

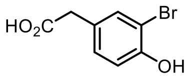

^{1}H NMR (400 MHz, MeOD) of **5c**

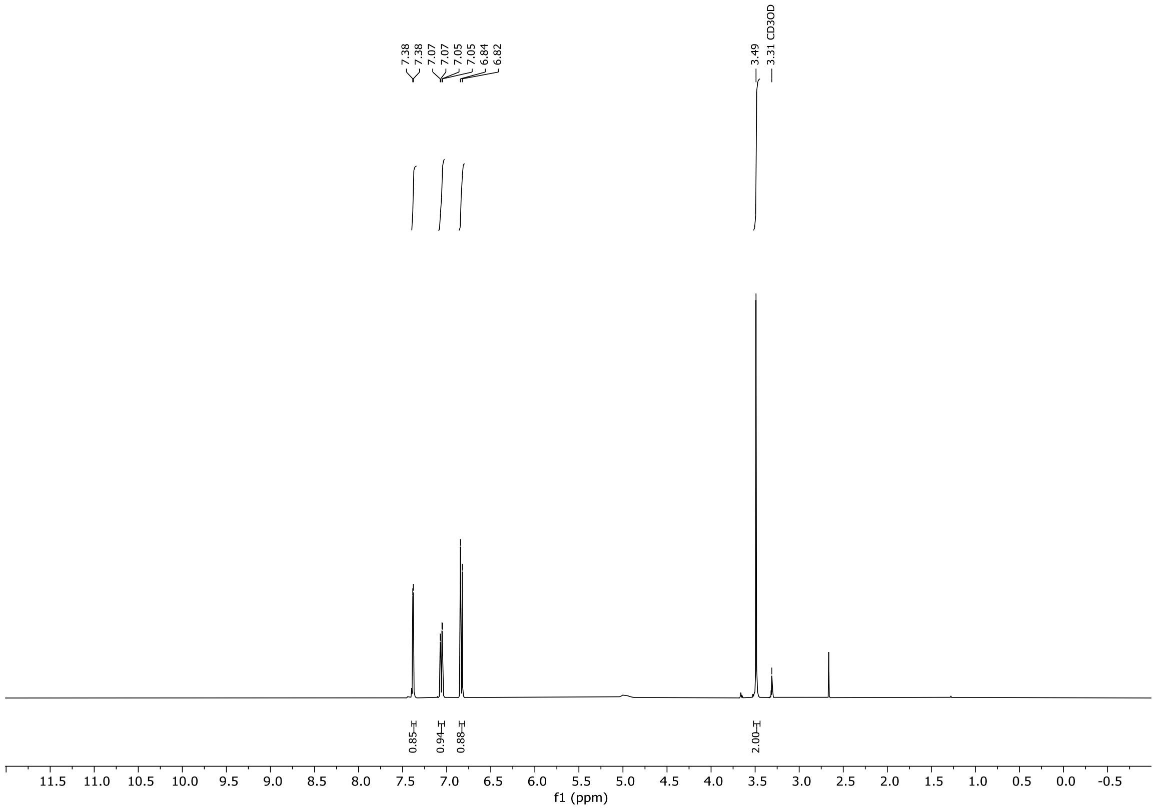

^{13}C NMR (101 MHz, MeOD) of **5c**

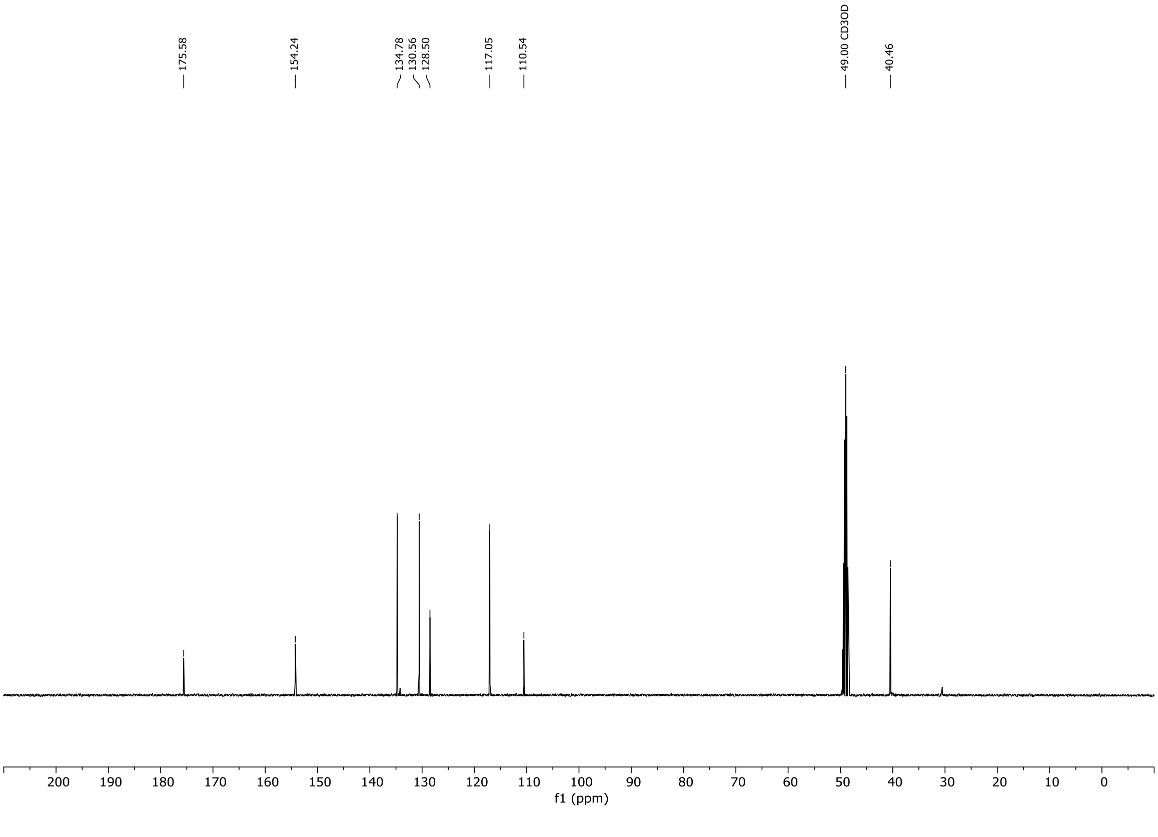

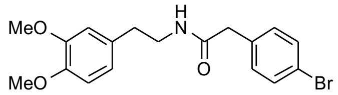

^{1}H NMR (400 MHz, CDCl3) of **6a**

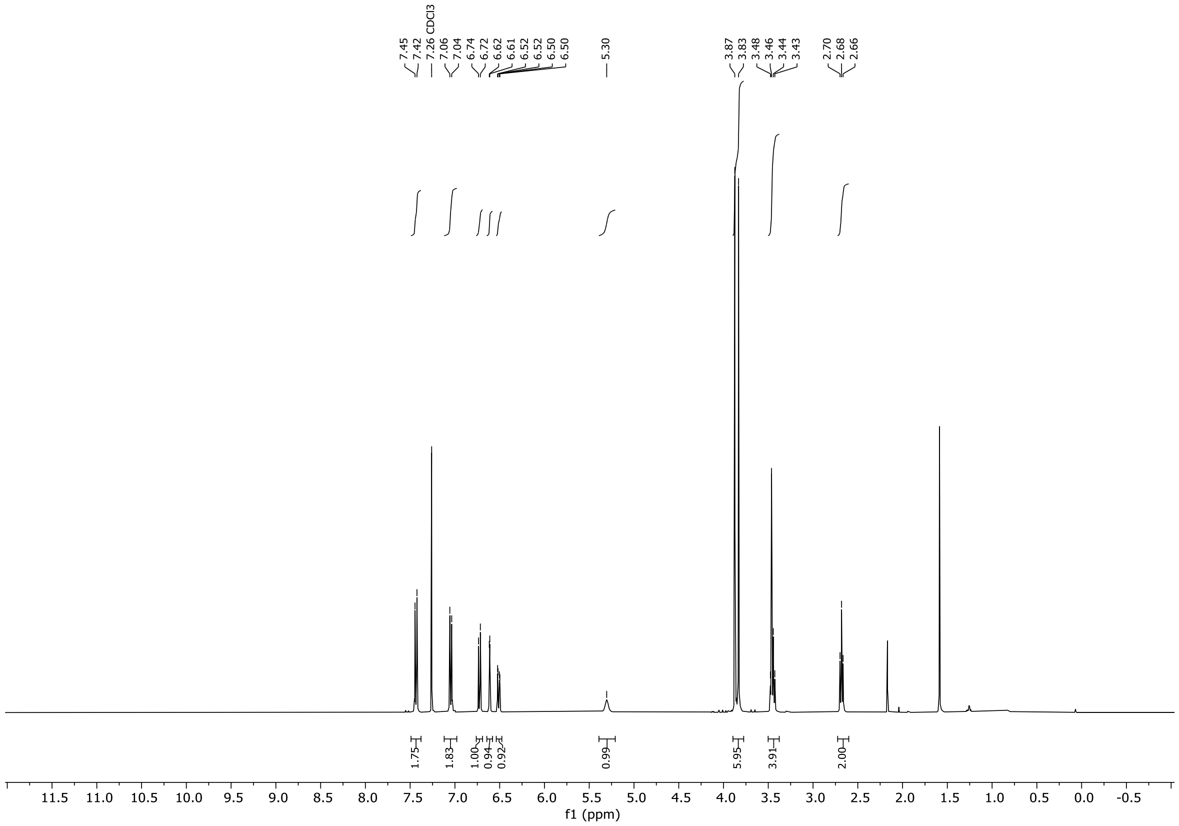

^{13}C NMR (101 MHz, CDCl3) of **6a**

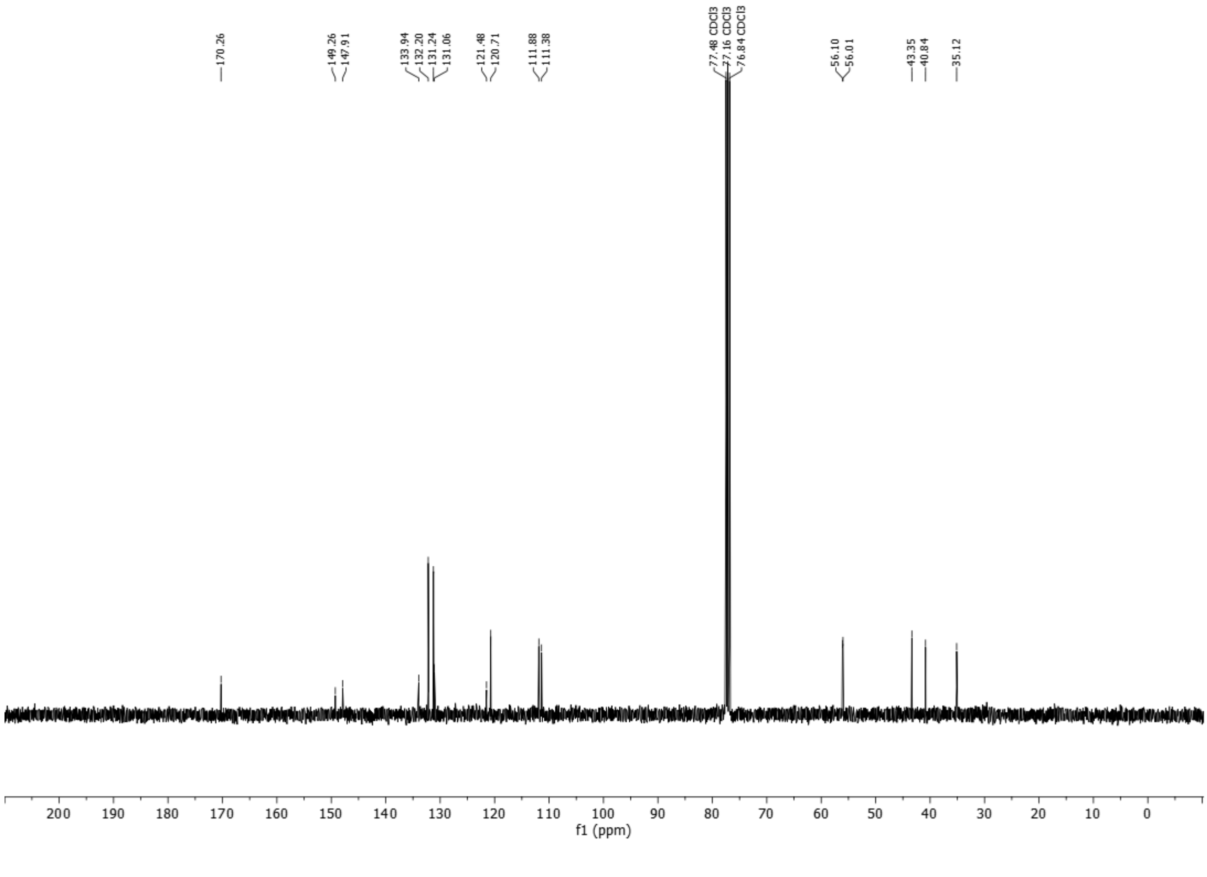

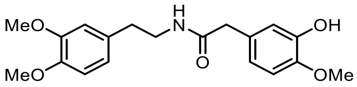

^{1}H NMR (400 MHz, $CDCl_3$) of **6b**

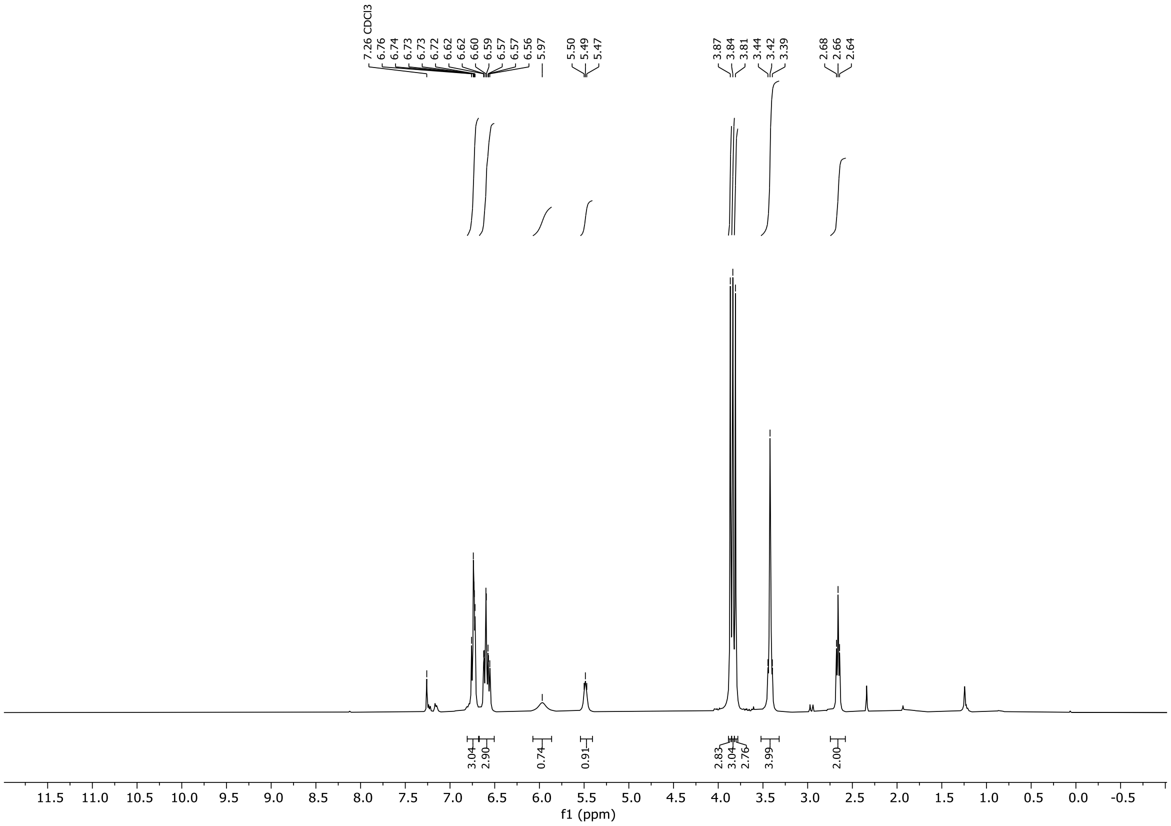

^{13}C NMR (101 MHz, $CDCl_3$) of **6b**

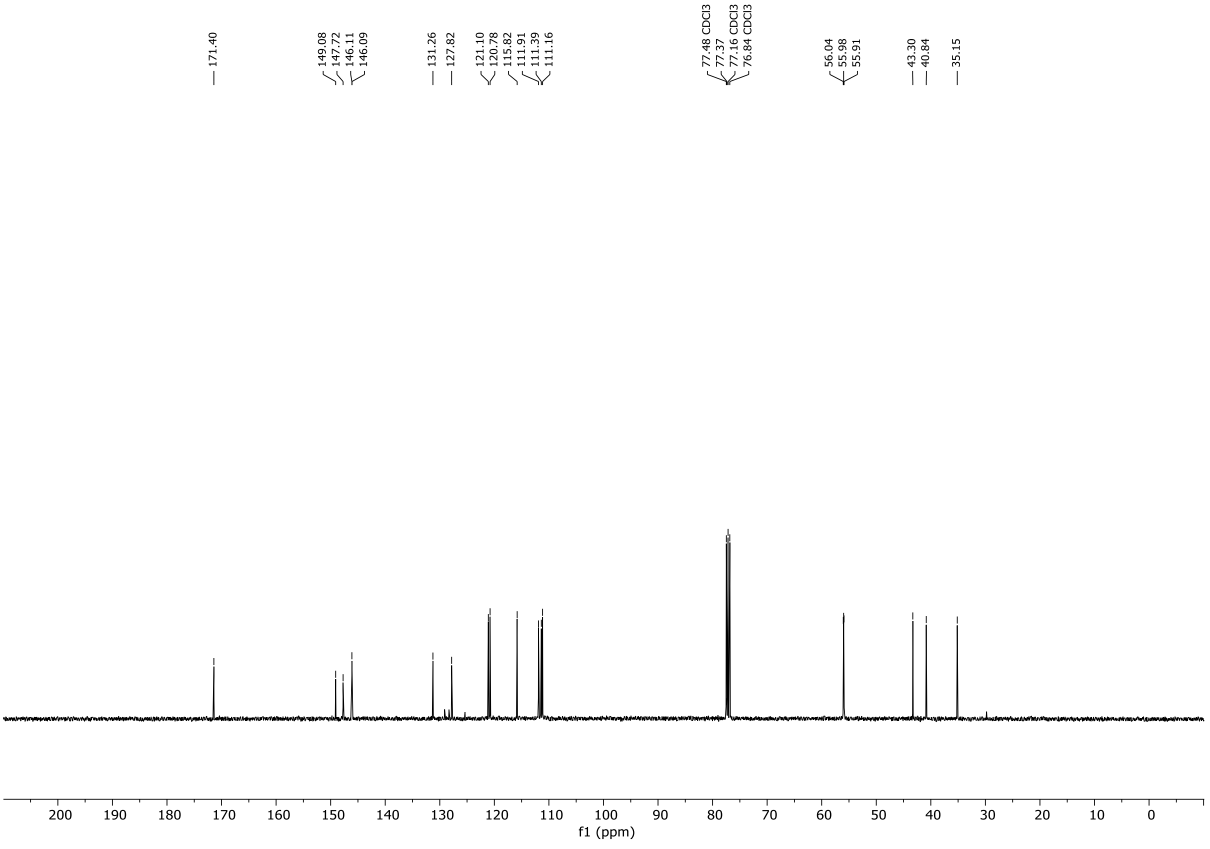

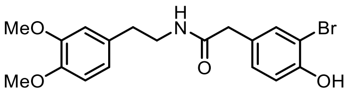

^{1}H NMR (500 MHz, $CDCl_3$) of **6c**

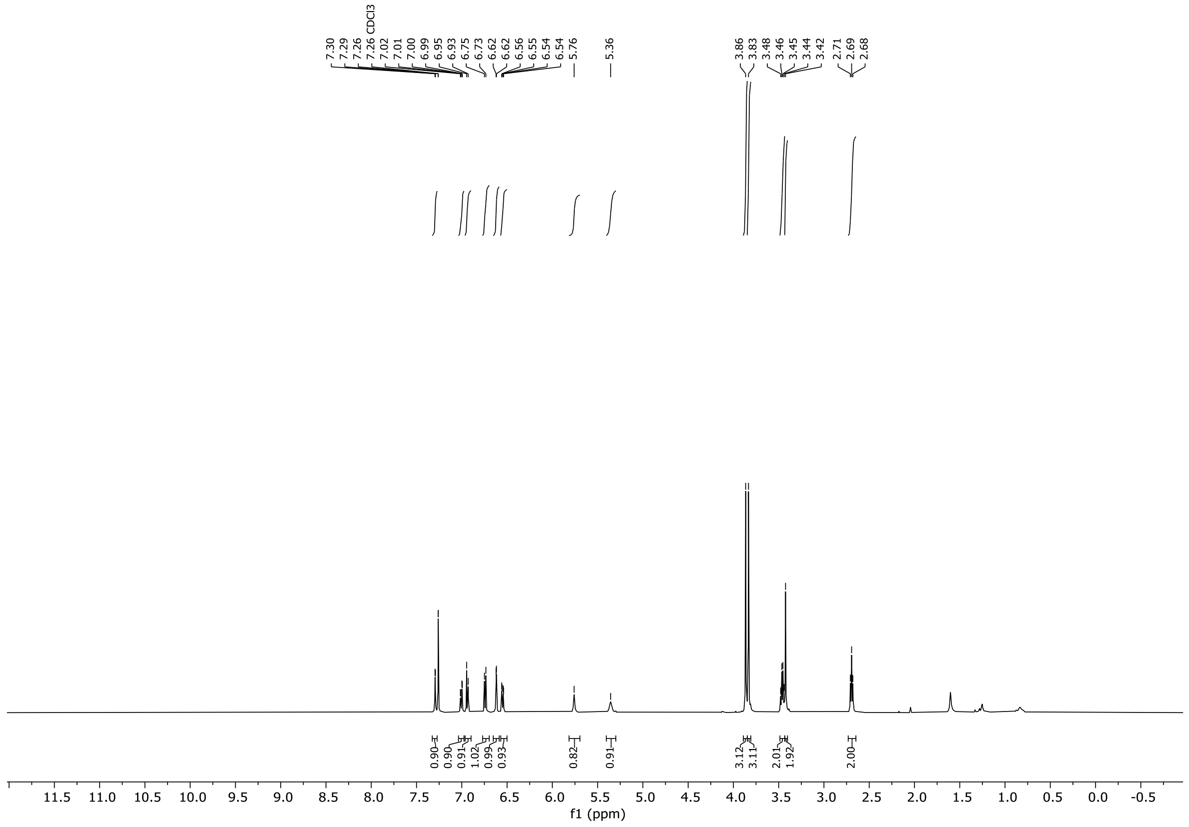

^{13}C NMR (126 MHz, $CDCl_3$) of **6c**

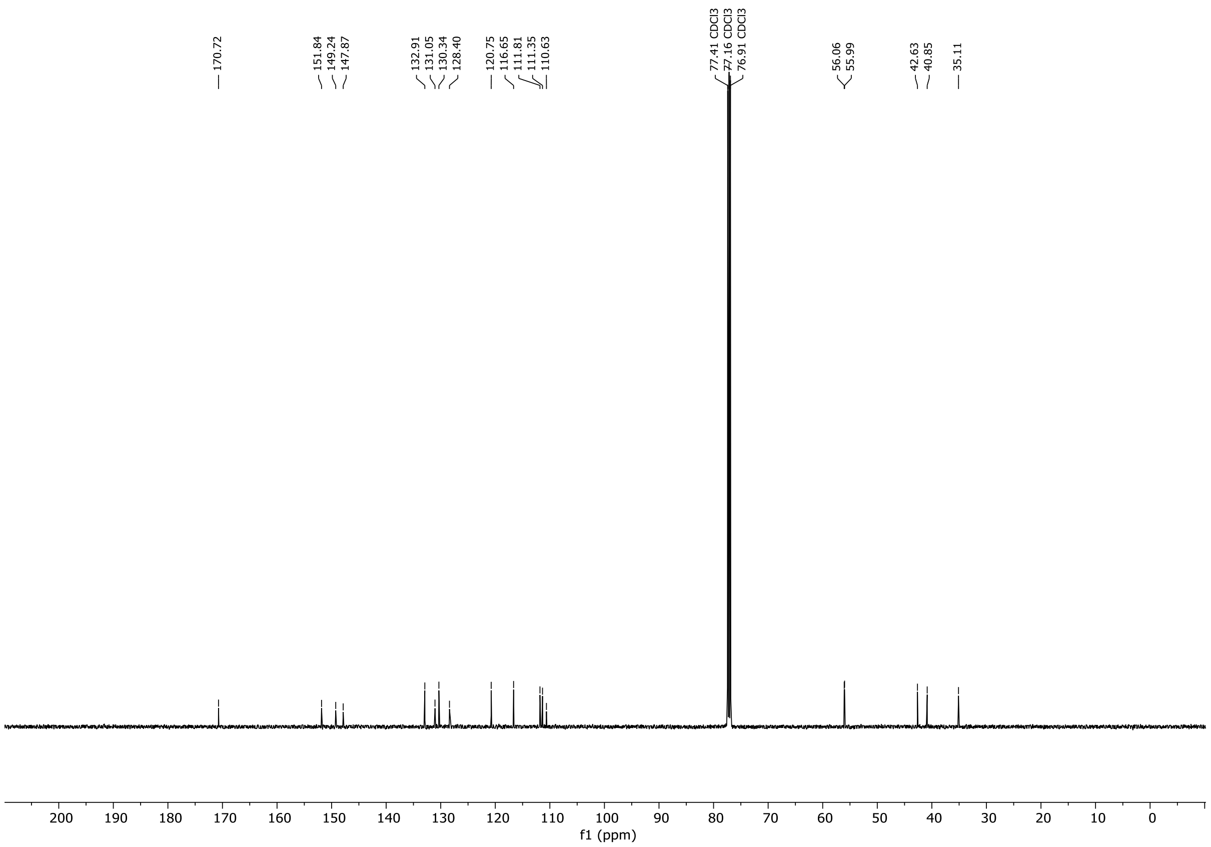

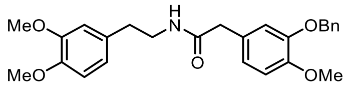

^{1}H NMR (500 MHz, $CDCl_3$) of **7a**

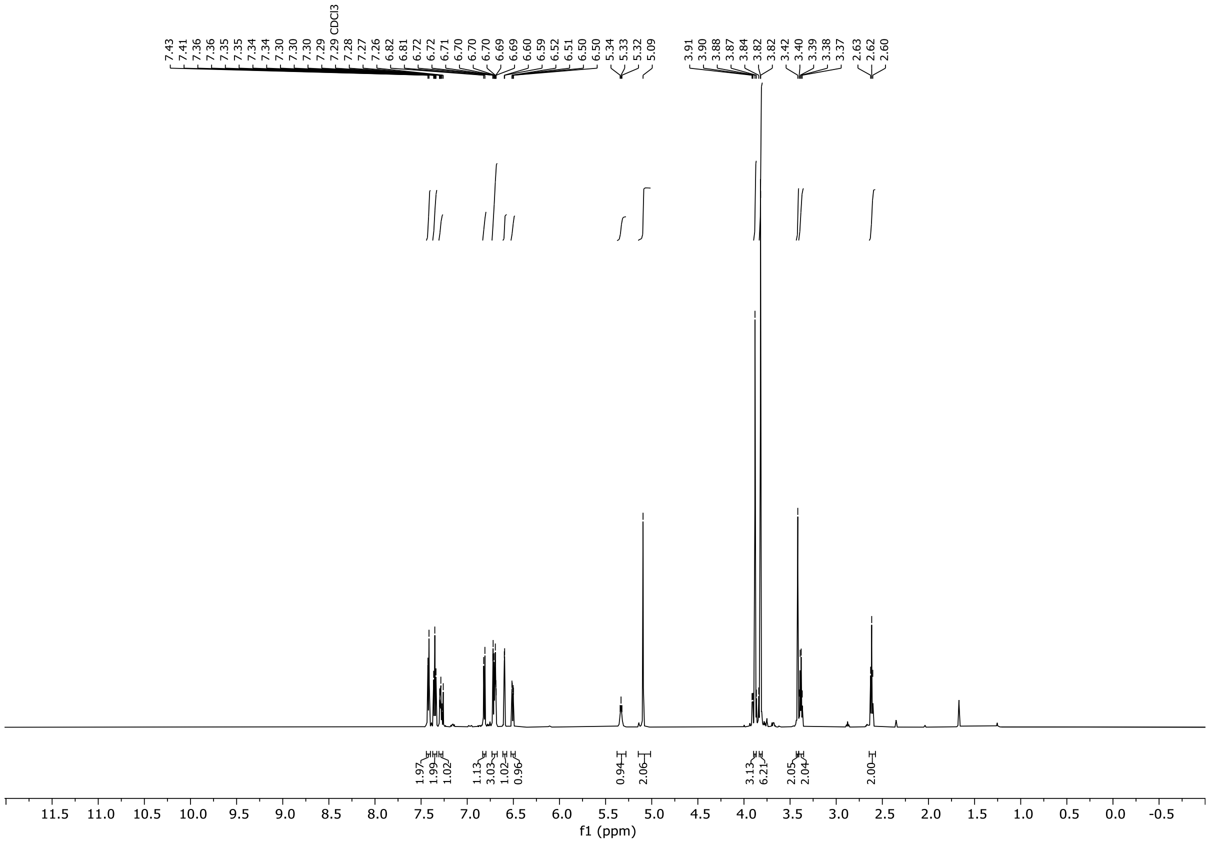

^{13}C NMR (151 MHz, $CDCl_3$) of **7a**

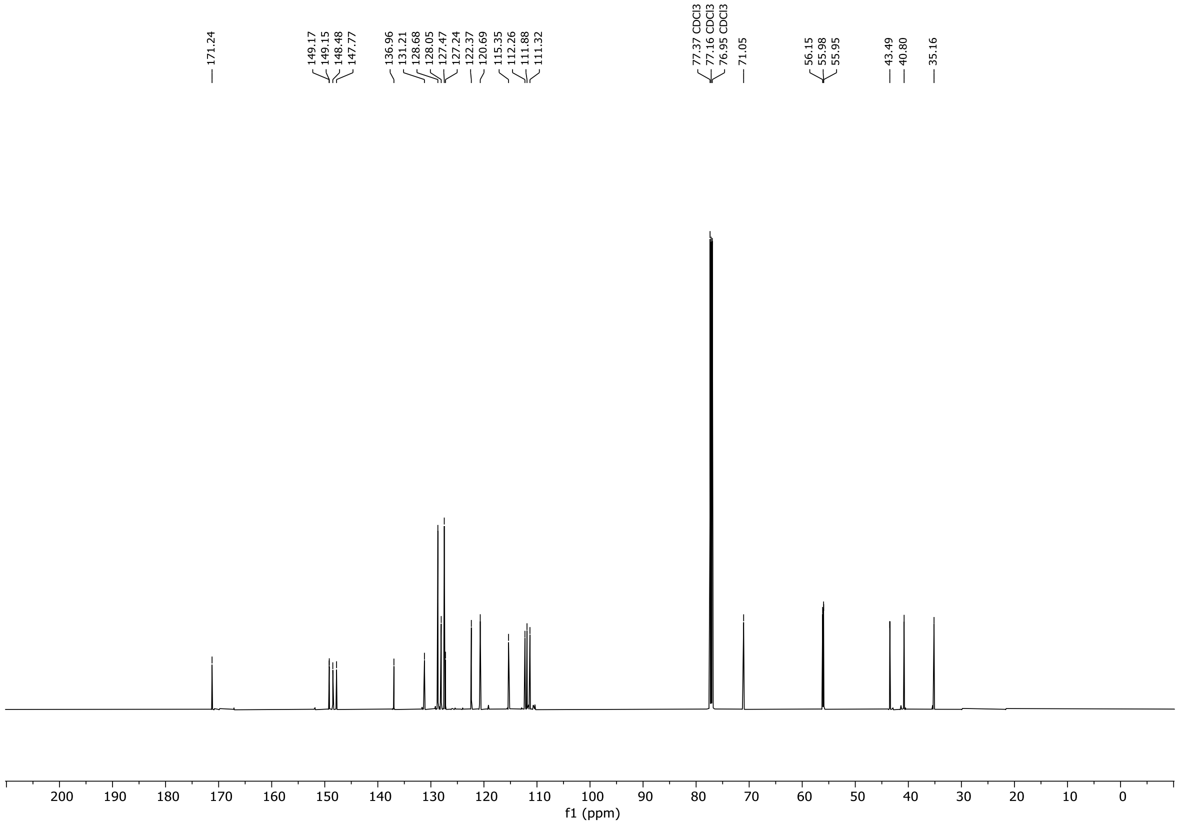

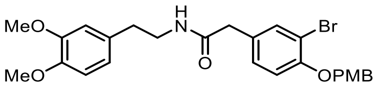

^{1}H NMR (500 MHz, $CDCl_3$) of **7b**

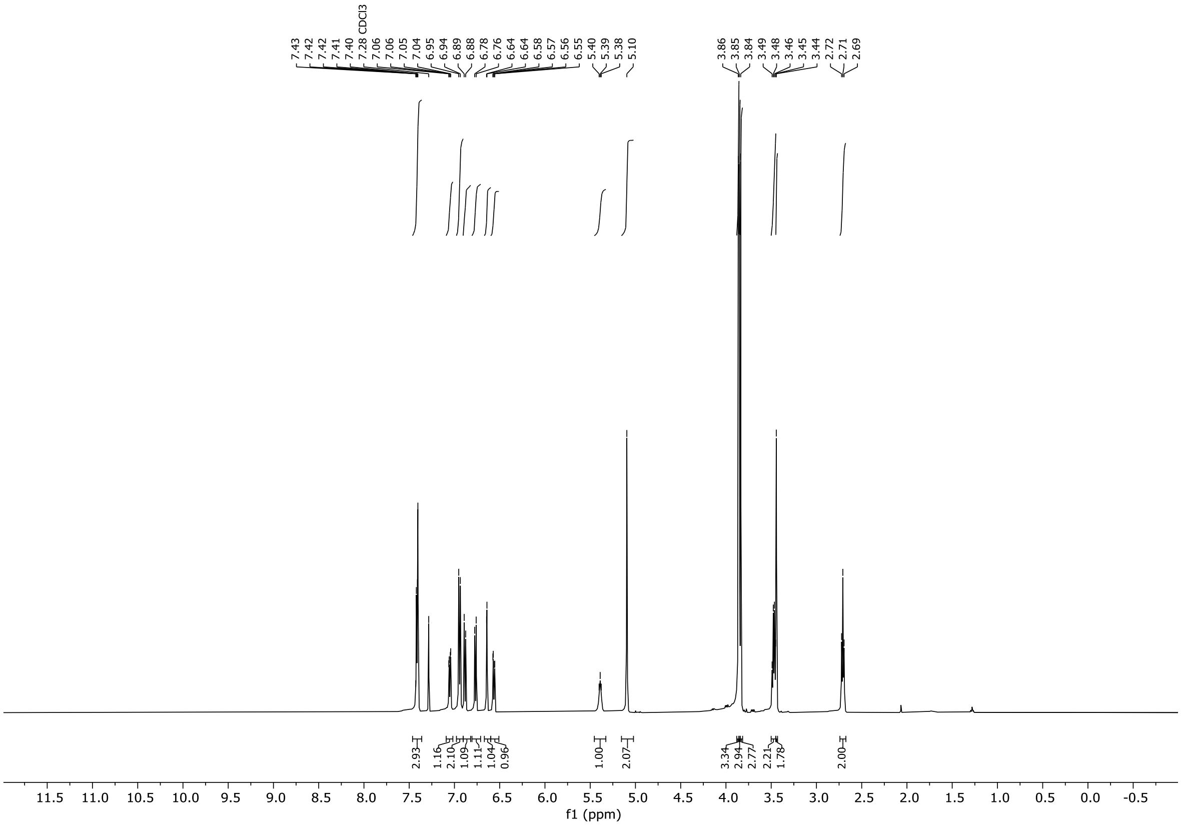

^{13}C NMR (126 MHz, $CDCl_3$) of **7b**

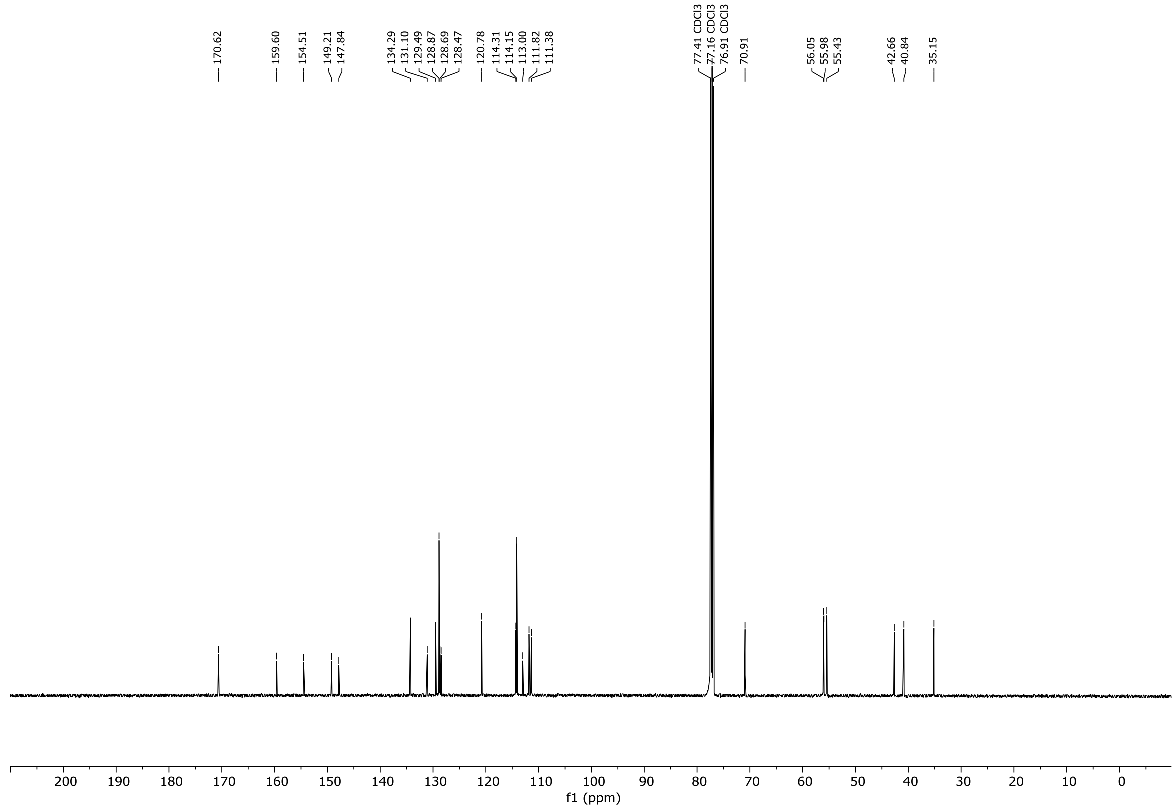

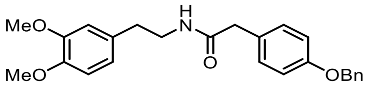

^{1}**H NMR** (500 MHz, $CDCl_3$) of **7c**

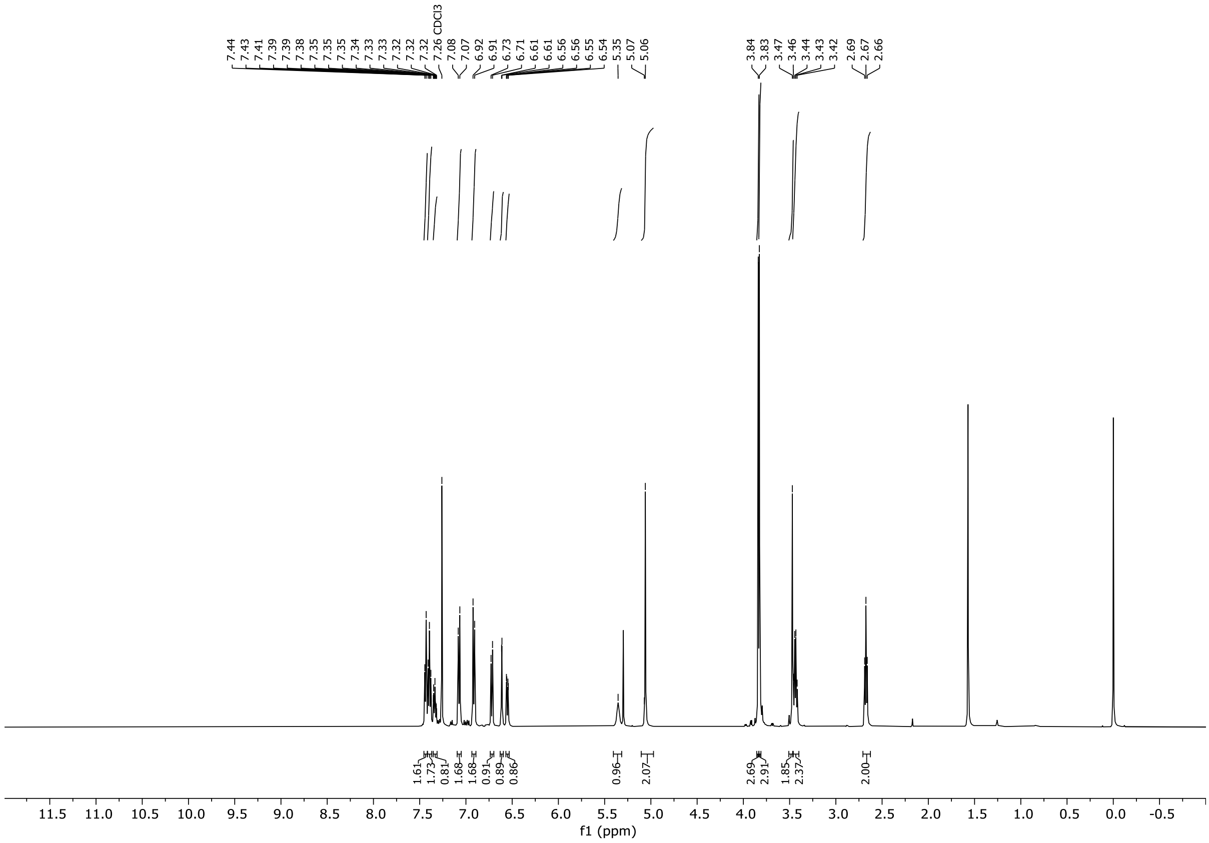

^{13}**C NMR** (126 MHz, $CDCl_3$) of **7c**

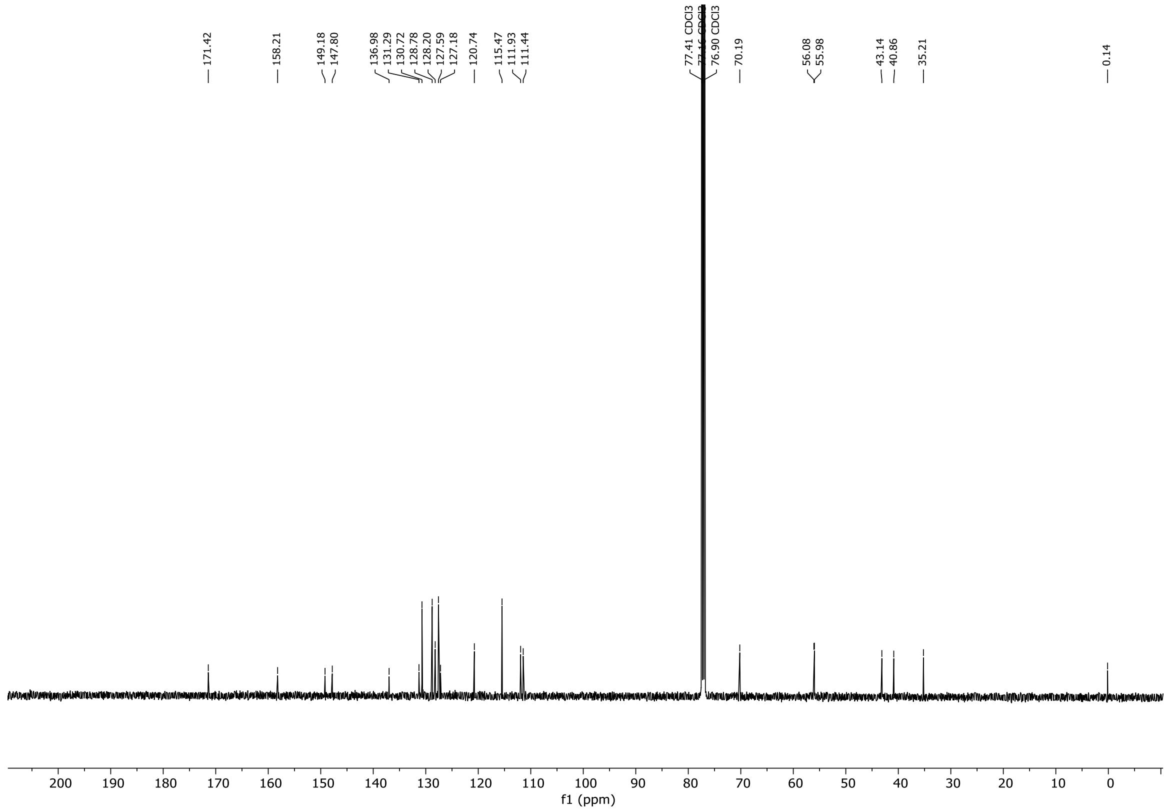

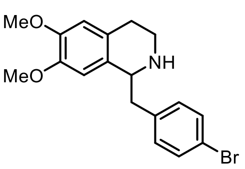

^{1}H NMR (500 MHz, $CDCl_3$) of **rac-8a**

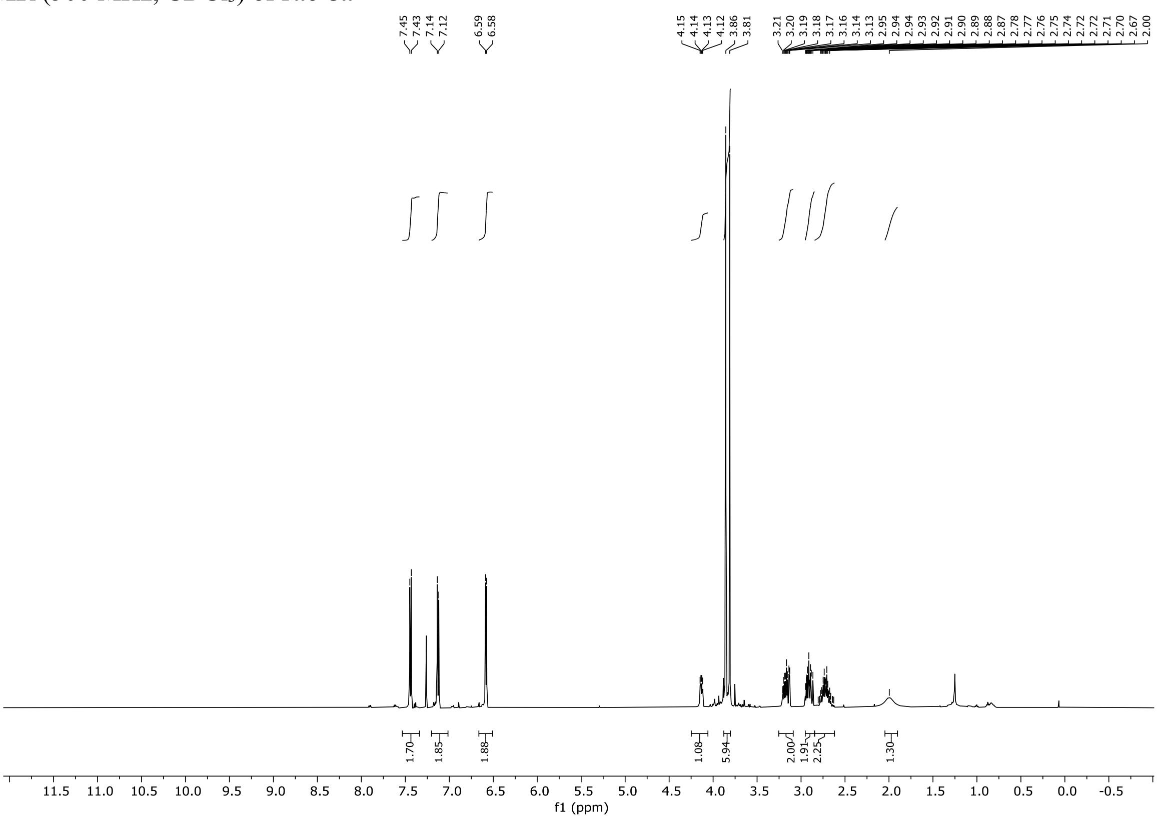

^{13}C NMR (126 MHz, $CDCl_3$) of **rac-8a**

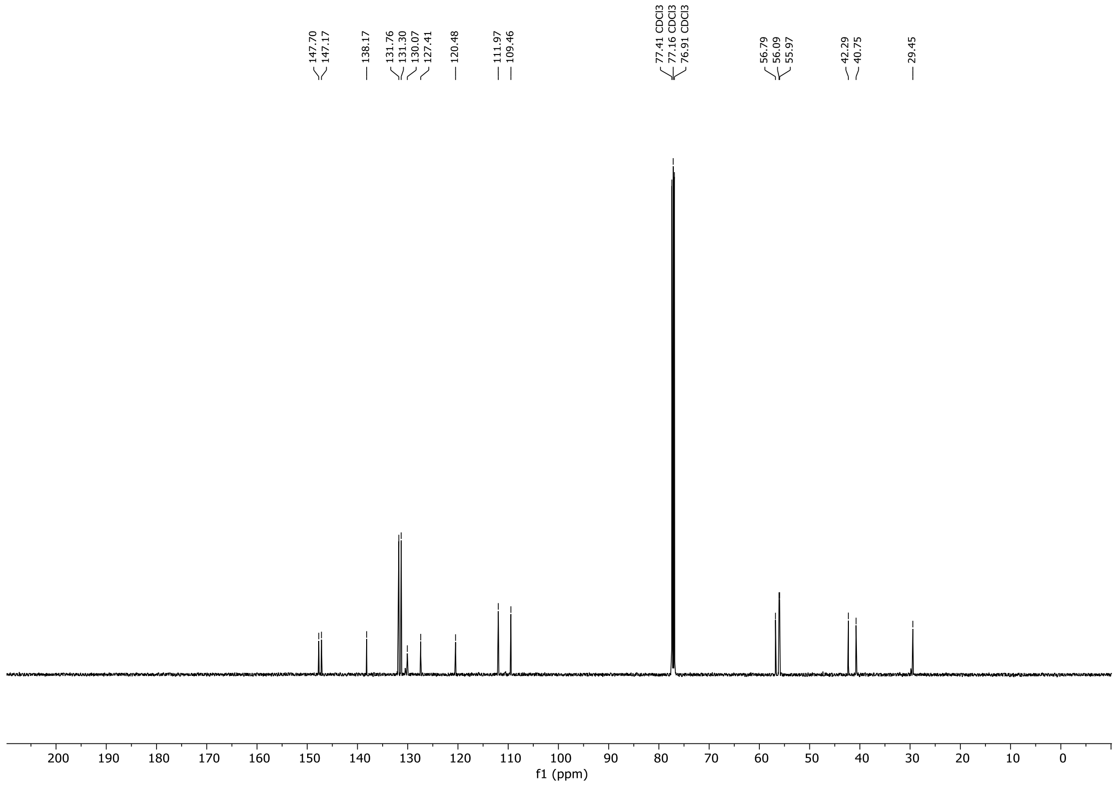

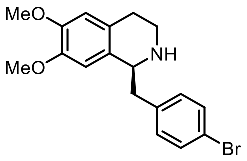

^{1}H NMR (400 MHz, $CDCl_3$) of ***(S)*-8a**

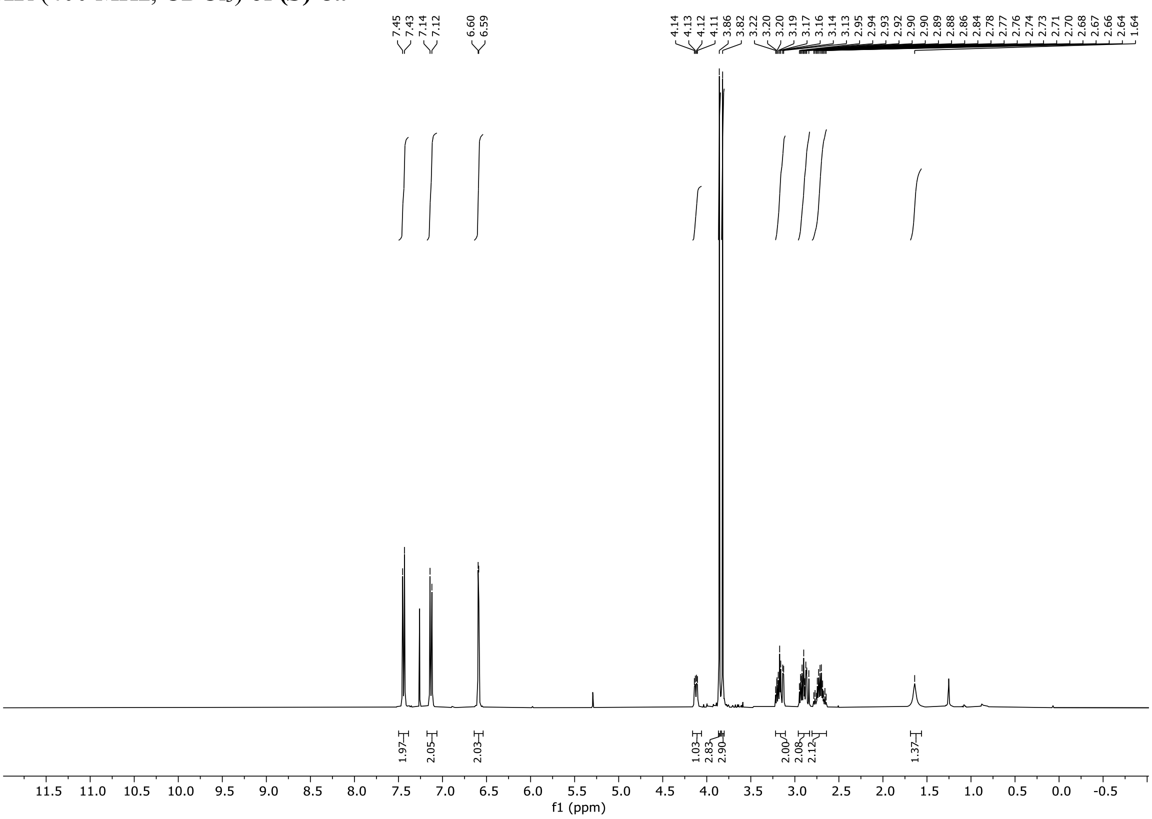

^{13}C NMR (101 MHz, $CDCl_3$) of ***(S)*-8a**

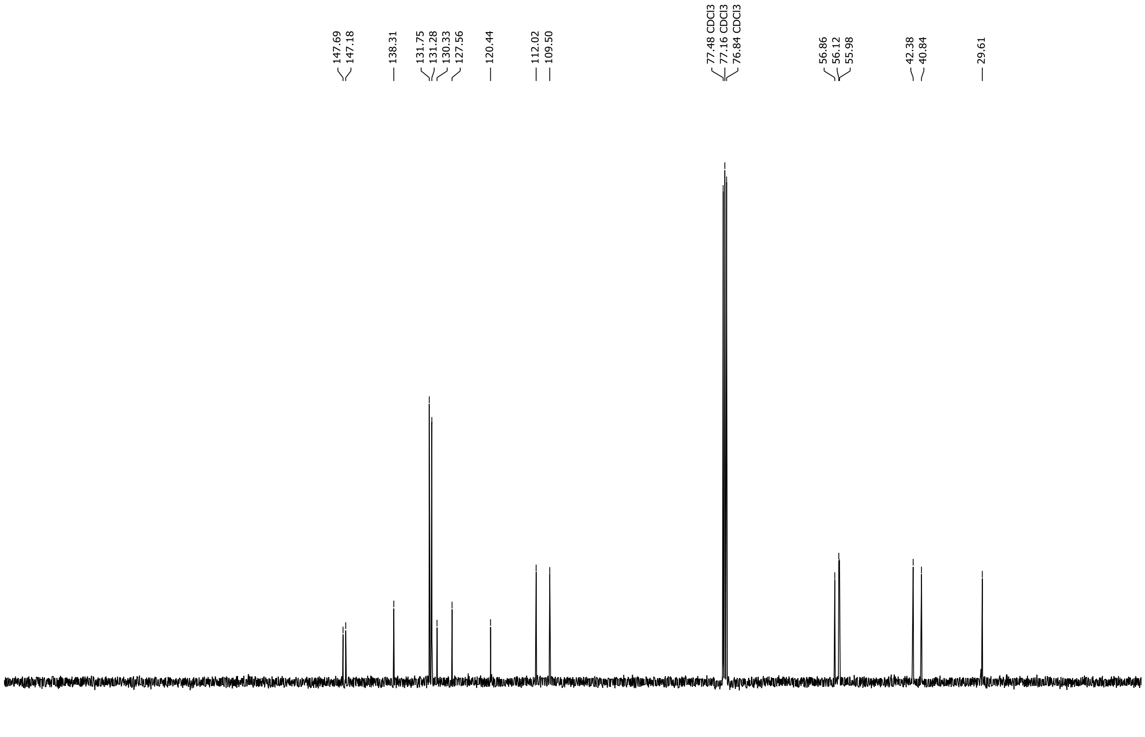

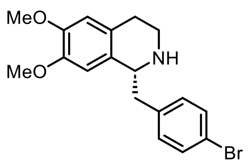

¹H NMR (400 MHz, $CDCl_3$) of ***(R)*-8a**

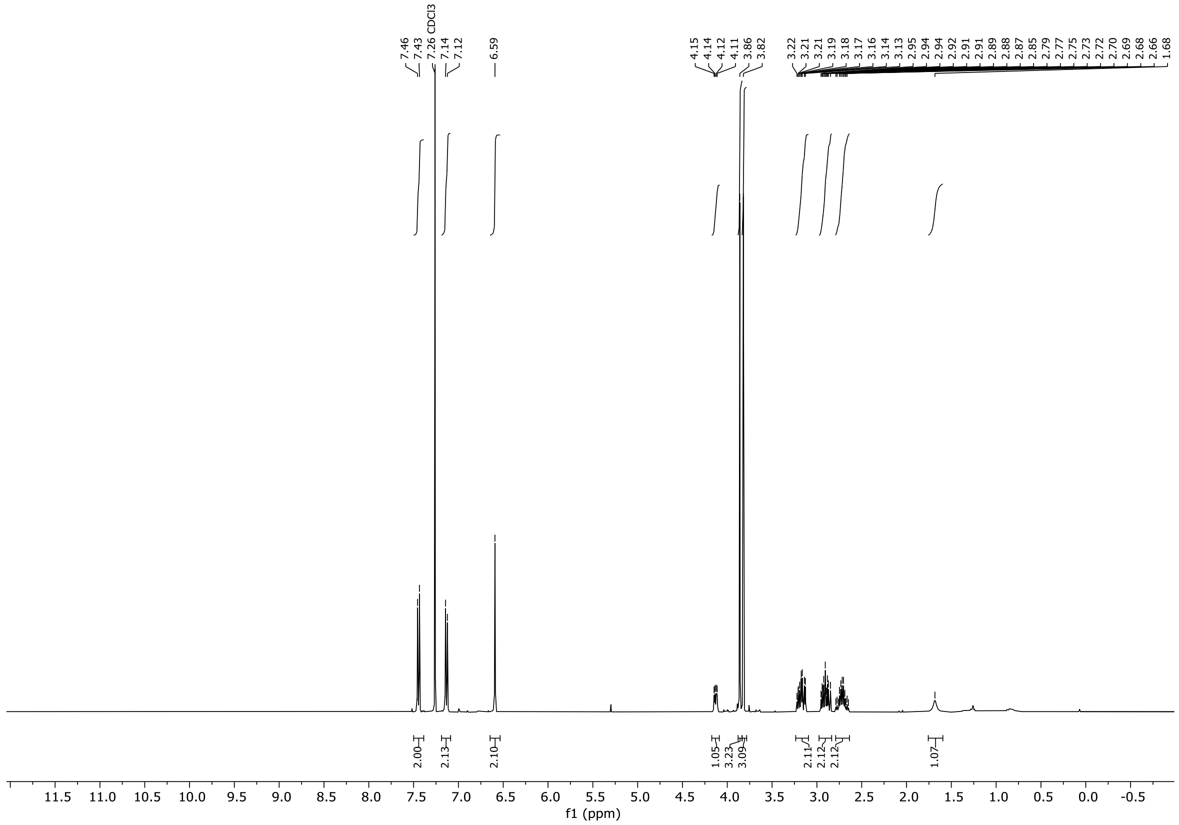

¹³C NMR (126 MHz, $CDCl_3$) of ***(R)*-8a**

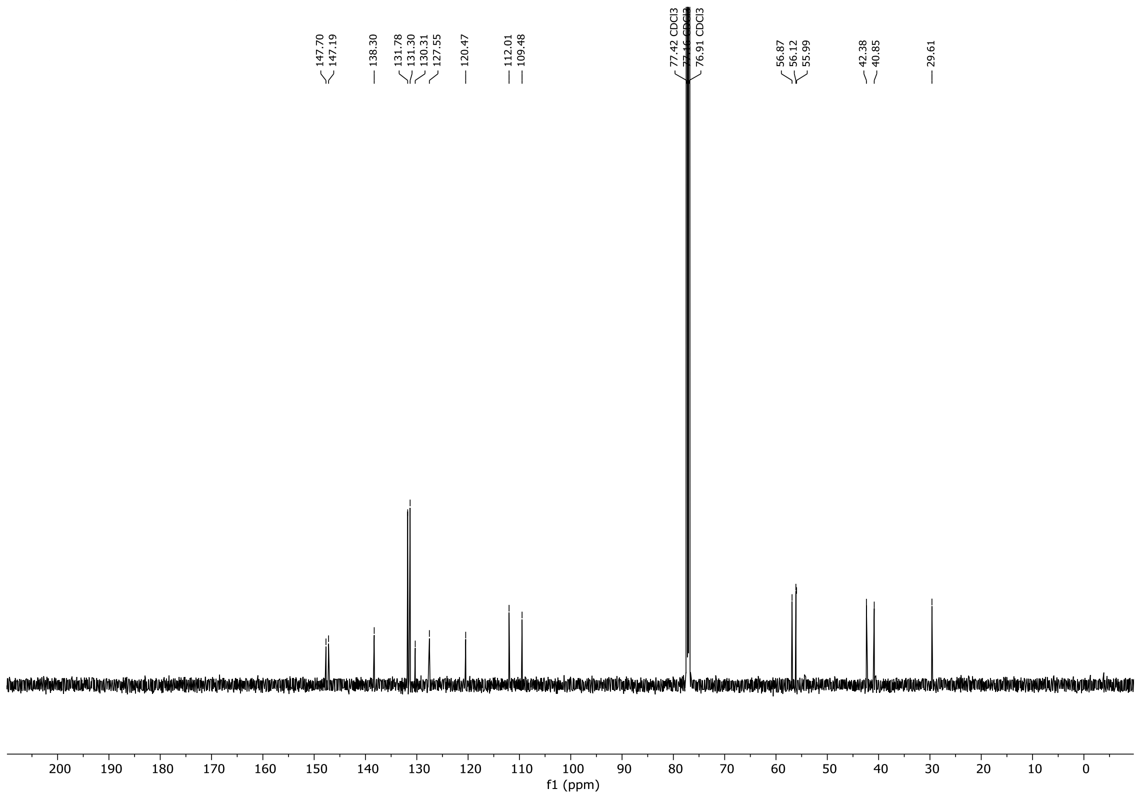

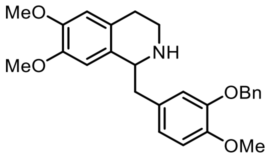

^{1}H NMR (400 MHz, $CDCl_3$) of **rac-8b**

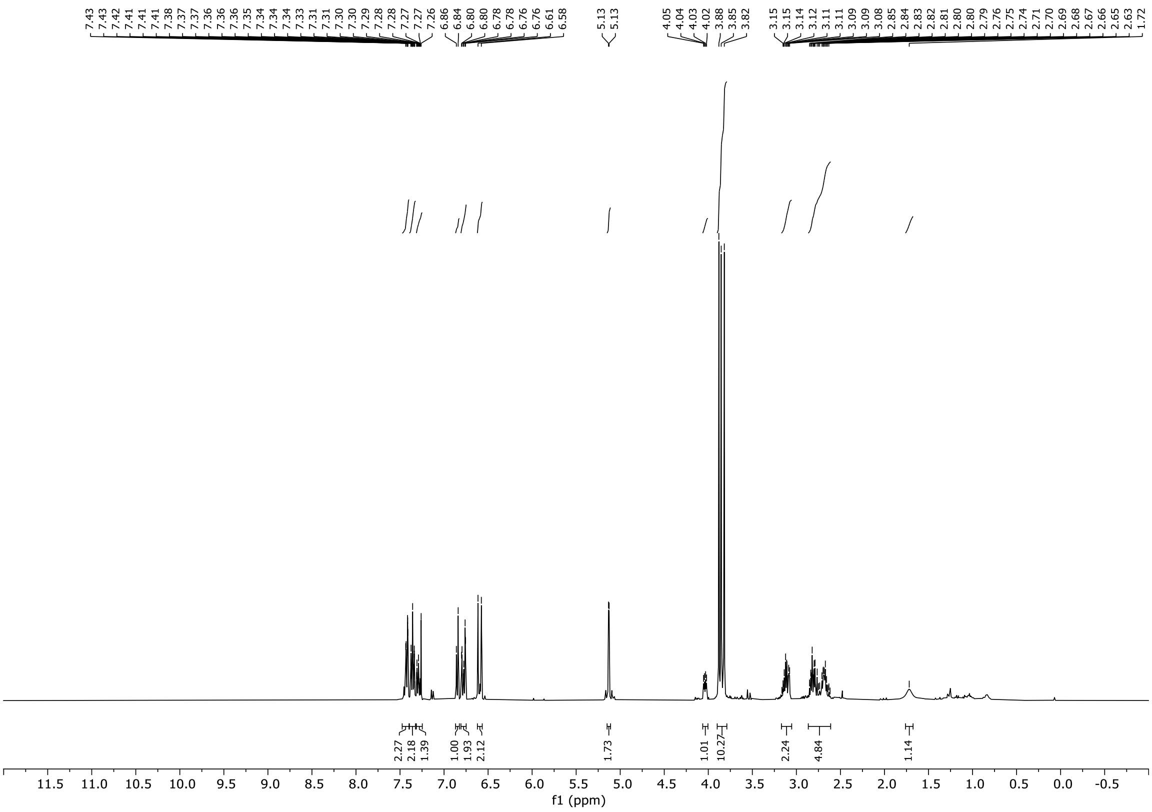

^{13}C NMR (101 MHz, $CDCl_3$) of **rac-8b**

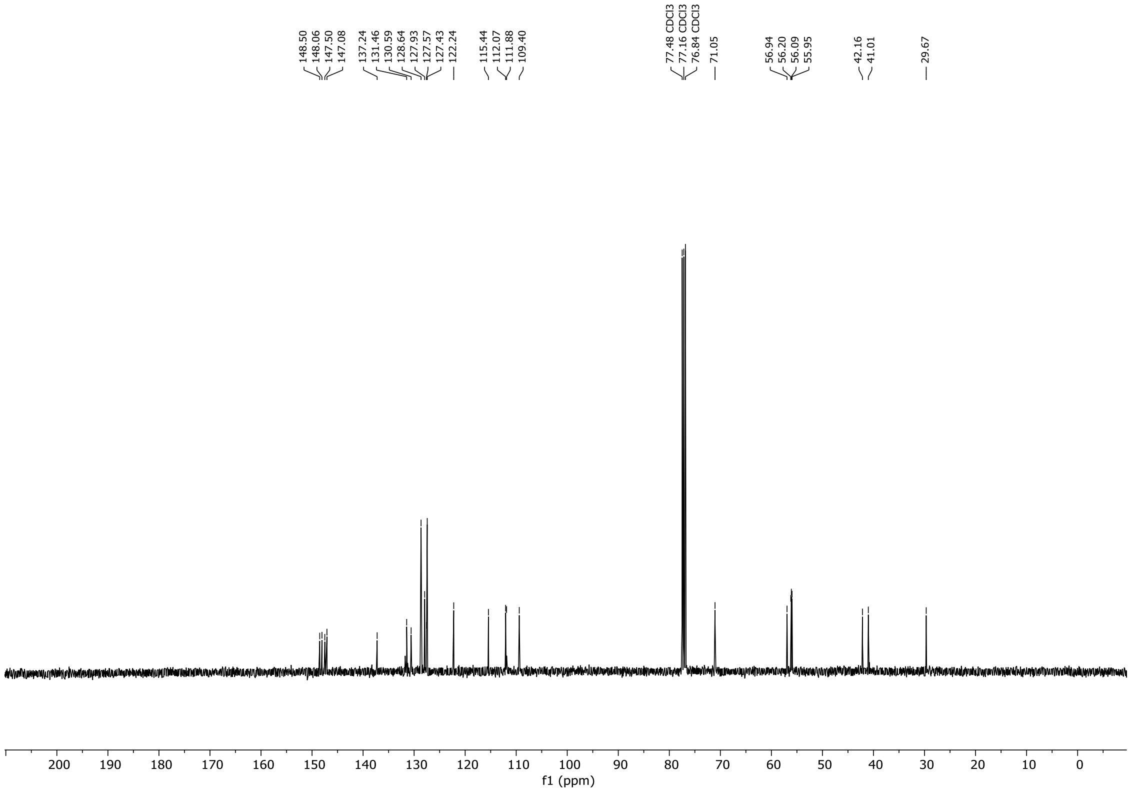

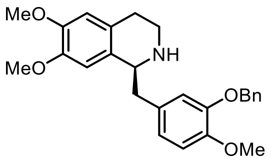

^{1}H NMR (400 MHz, $CDCl_3$) of ***(S)*-8b**

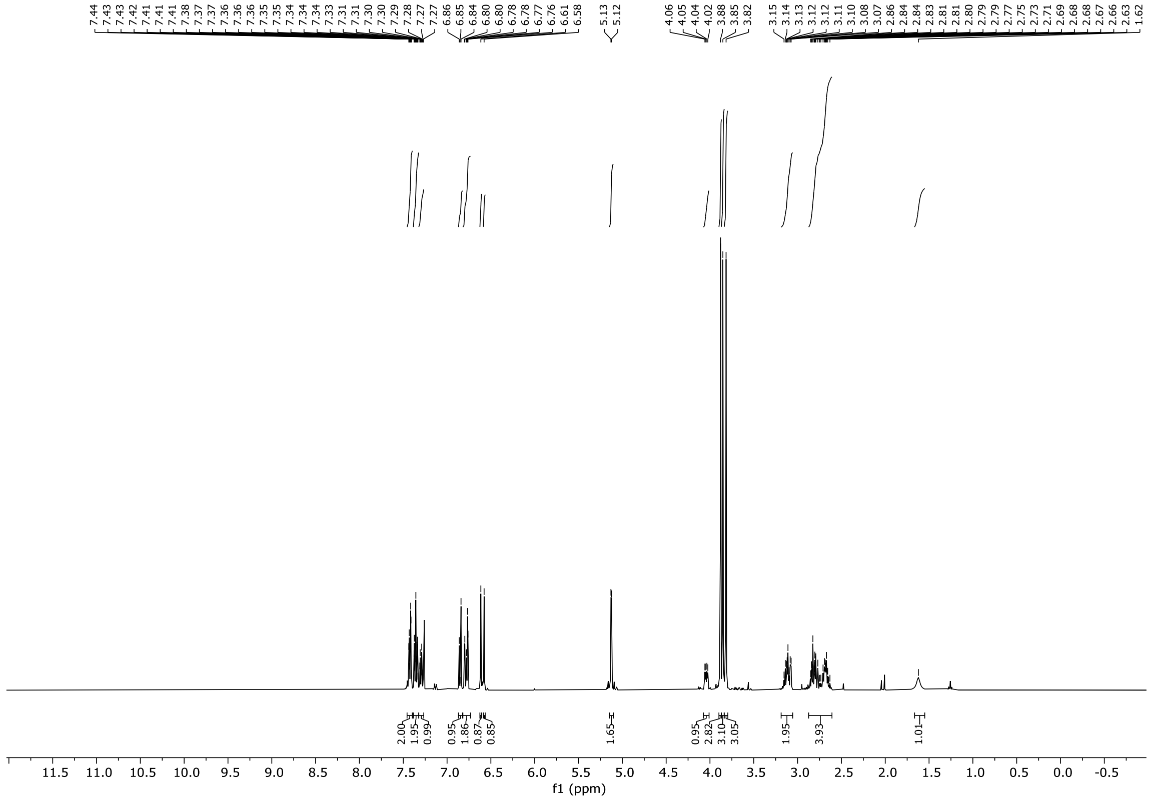

^{13}C NMR (101 MHz, $CDCl_3$)

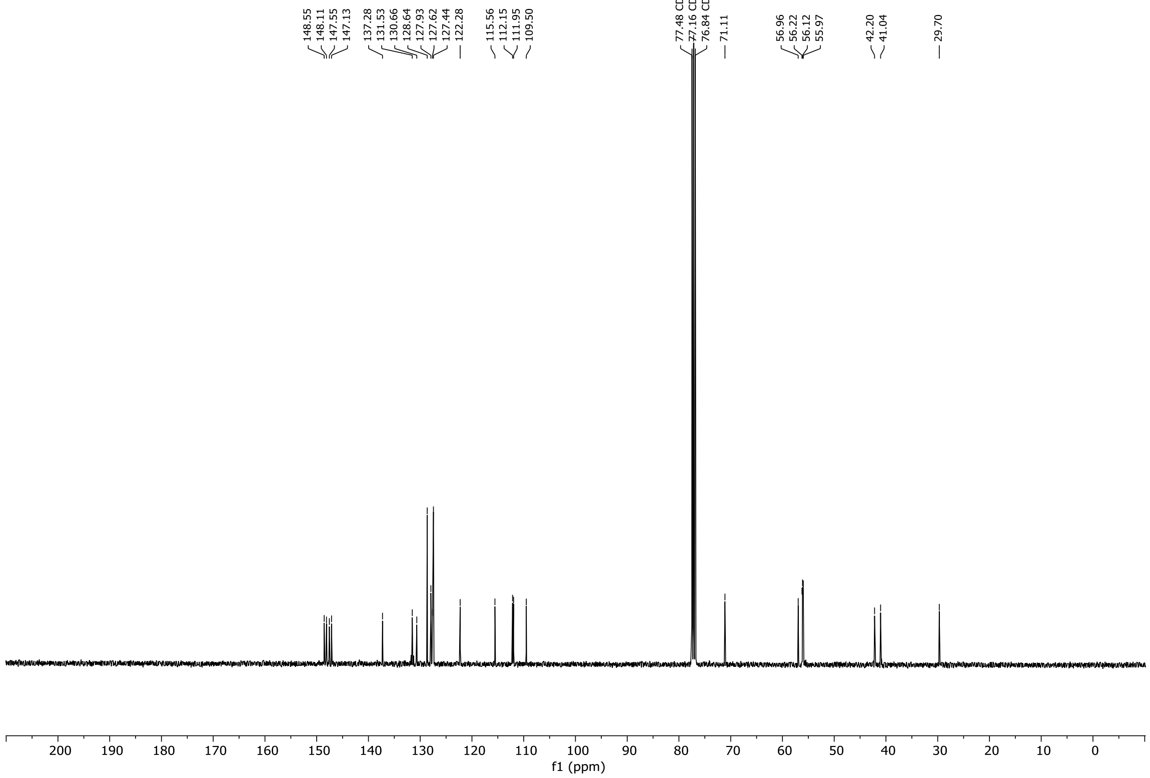

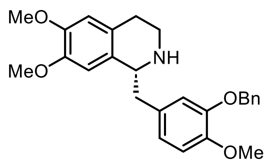

^{1}H NMR (500 MHz, $CDCl_3$) of ***(R)*-8b**

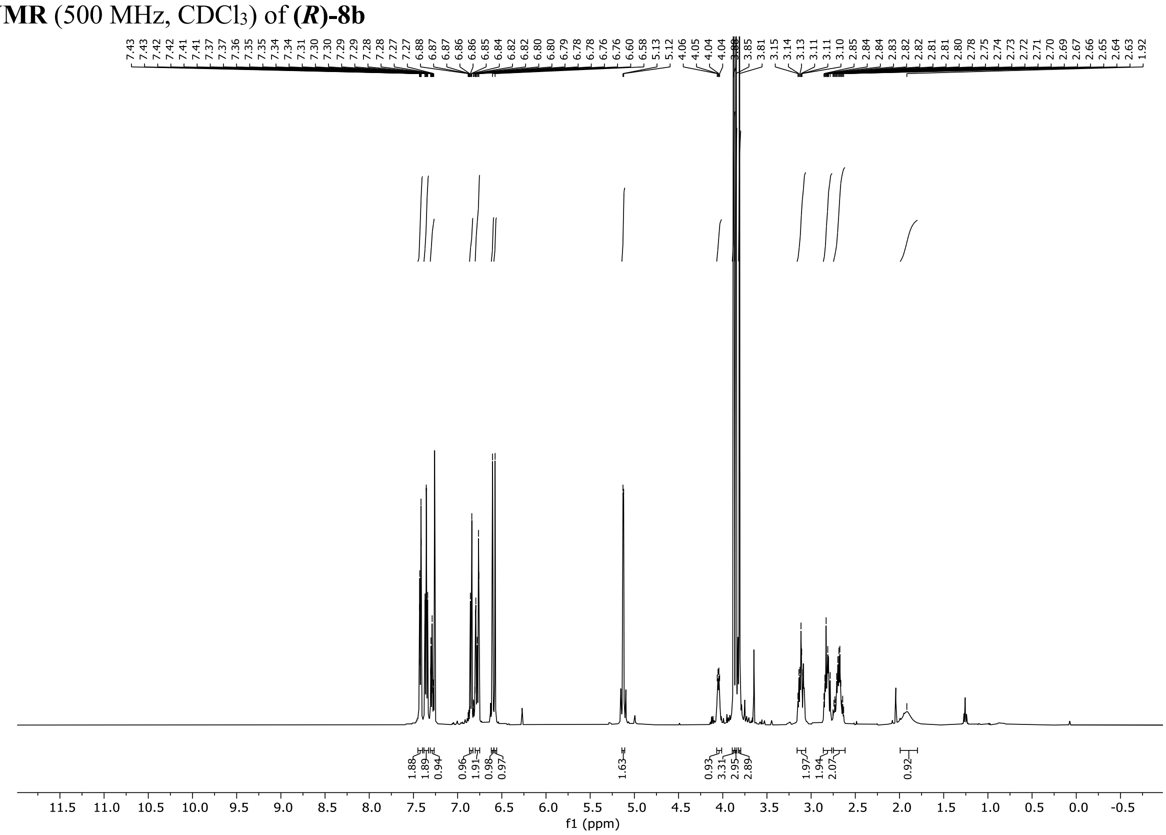

^{13}C NMR (126 MHz, $CDCl_3$) of ***(R)*-8b**

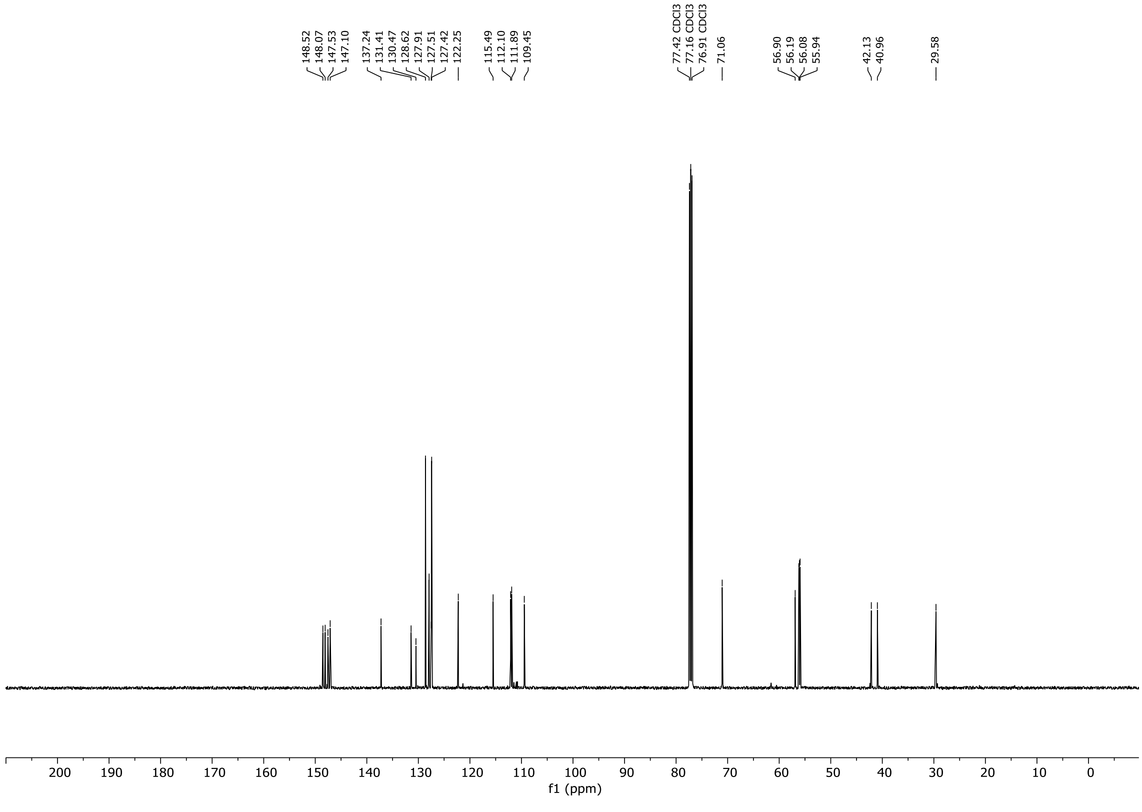

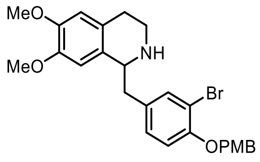

^{1}H NMR (500 MHz, $CDCl_3$) of **rac-8c**

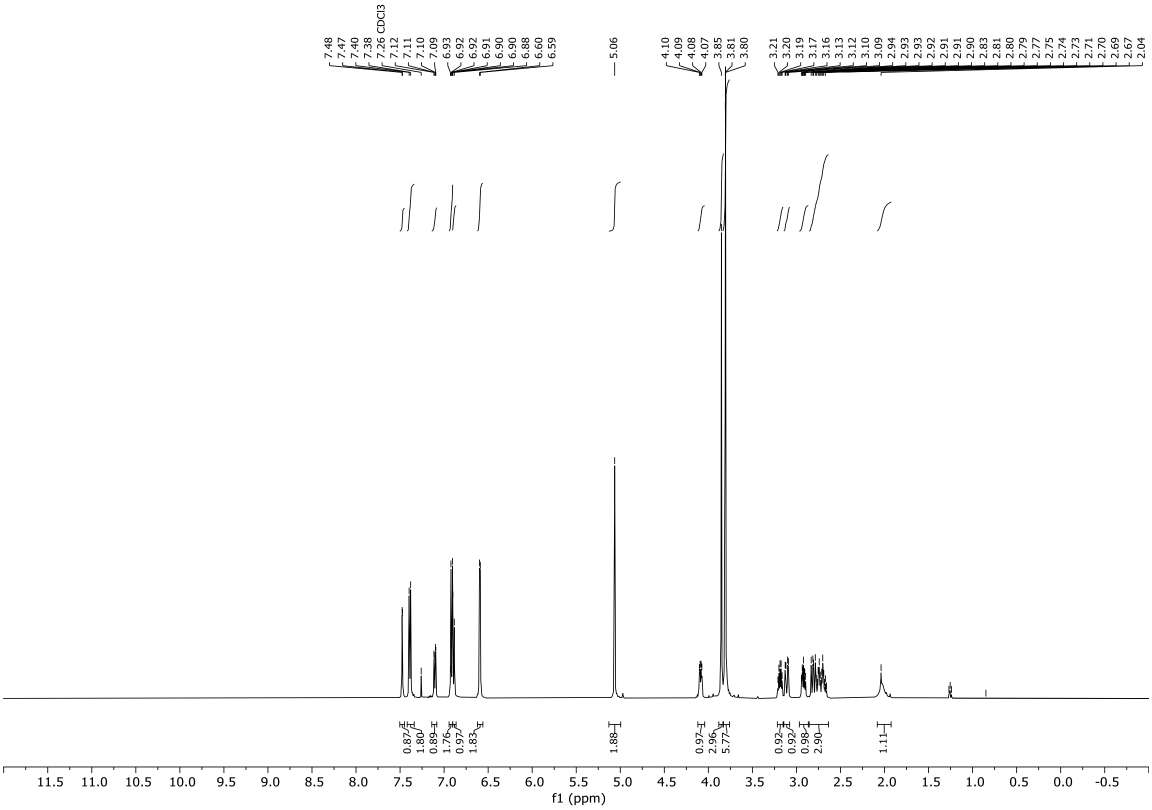

^{13}C NMR (126 MHz, $CDCl_3$) of **rac-8c**

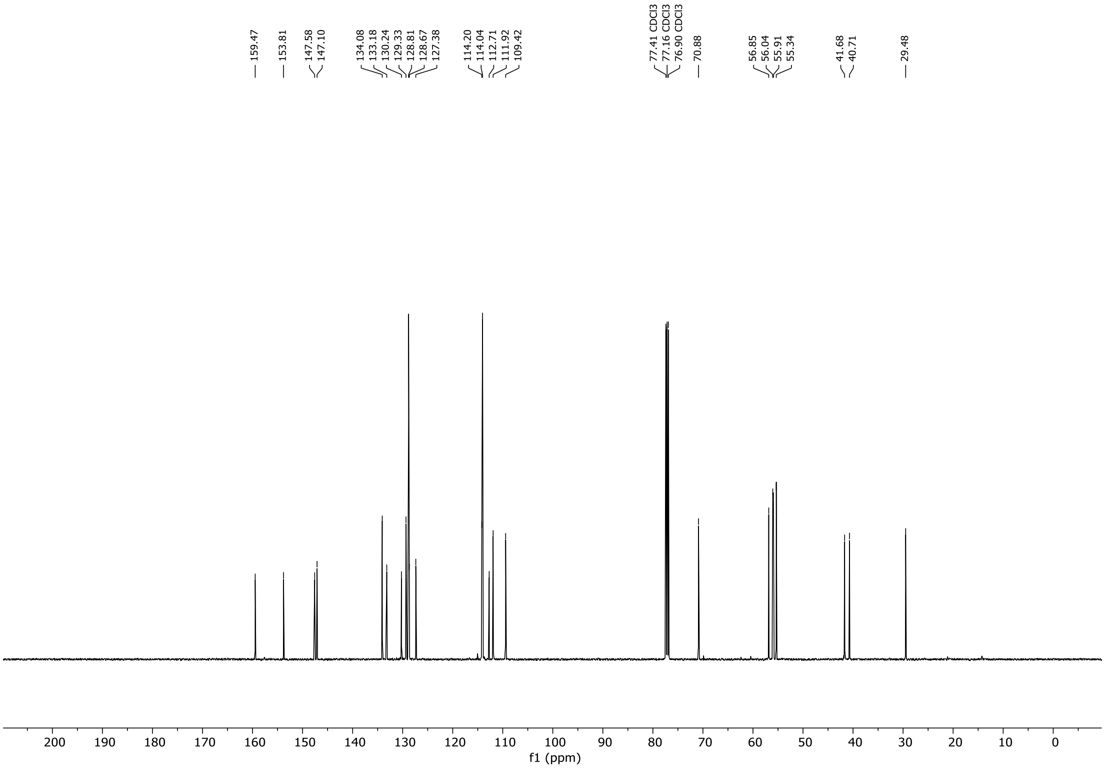

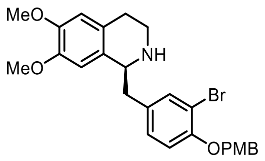

^{1}H NMR (500 MHz, $CDCl_3$) of ***(S)*-8c**

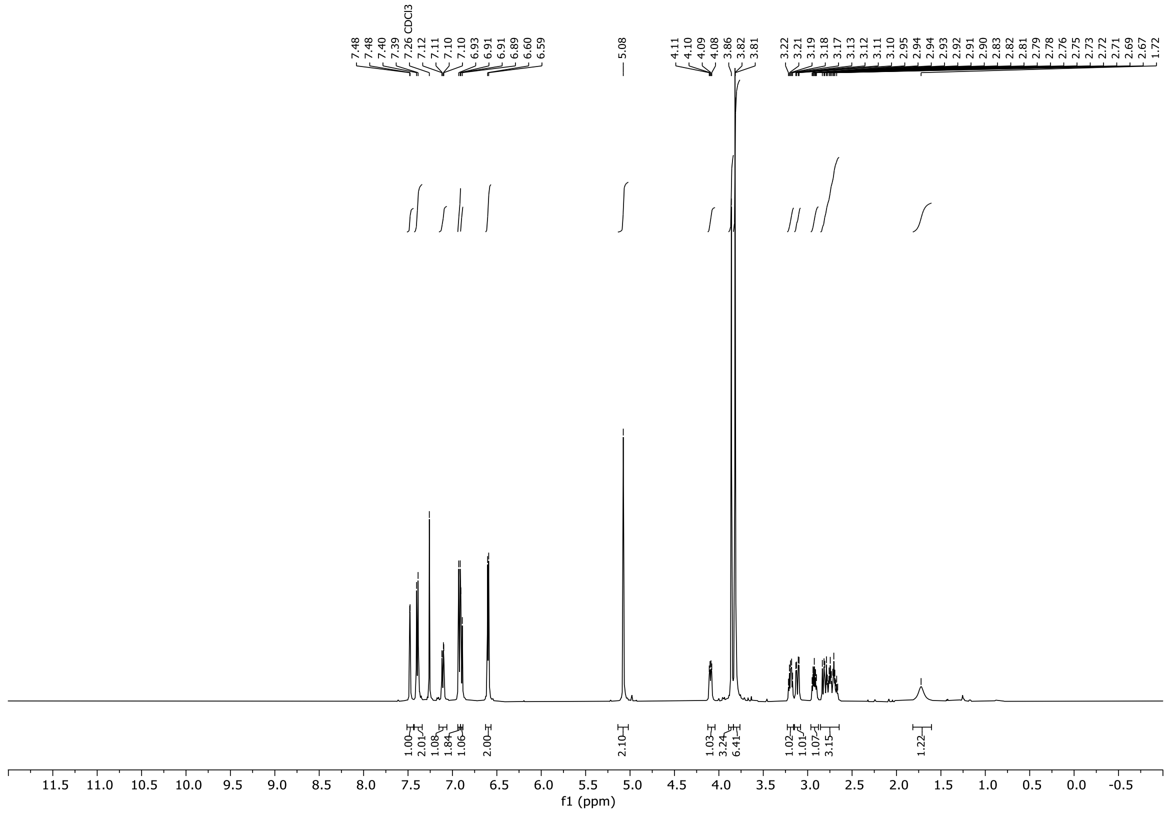

^{13}C NMR (126 MHz, $CDCl_3$) of ***(S)*-8c**

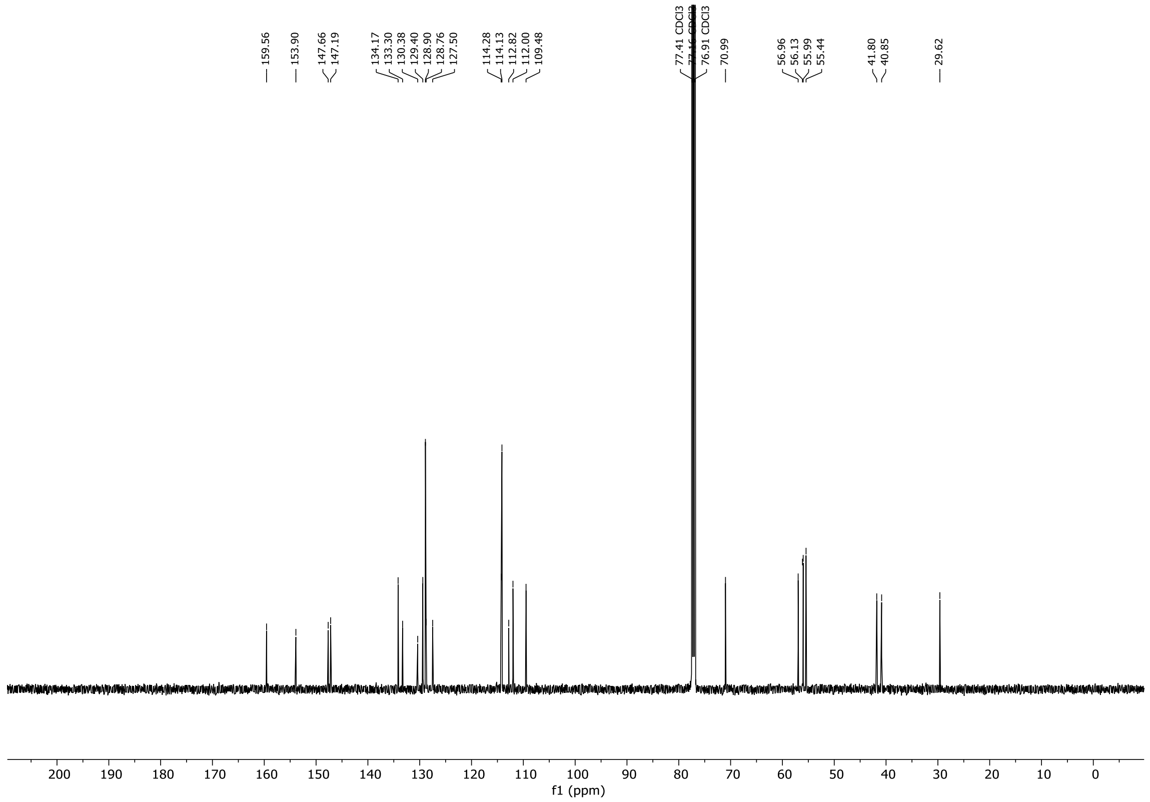

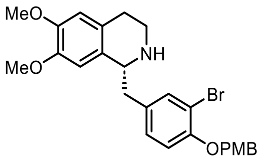

^{1}H NMR (500 MHz, CDCl$_3$) of ***(R)*-8c**

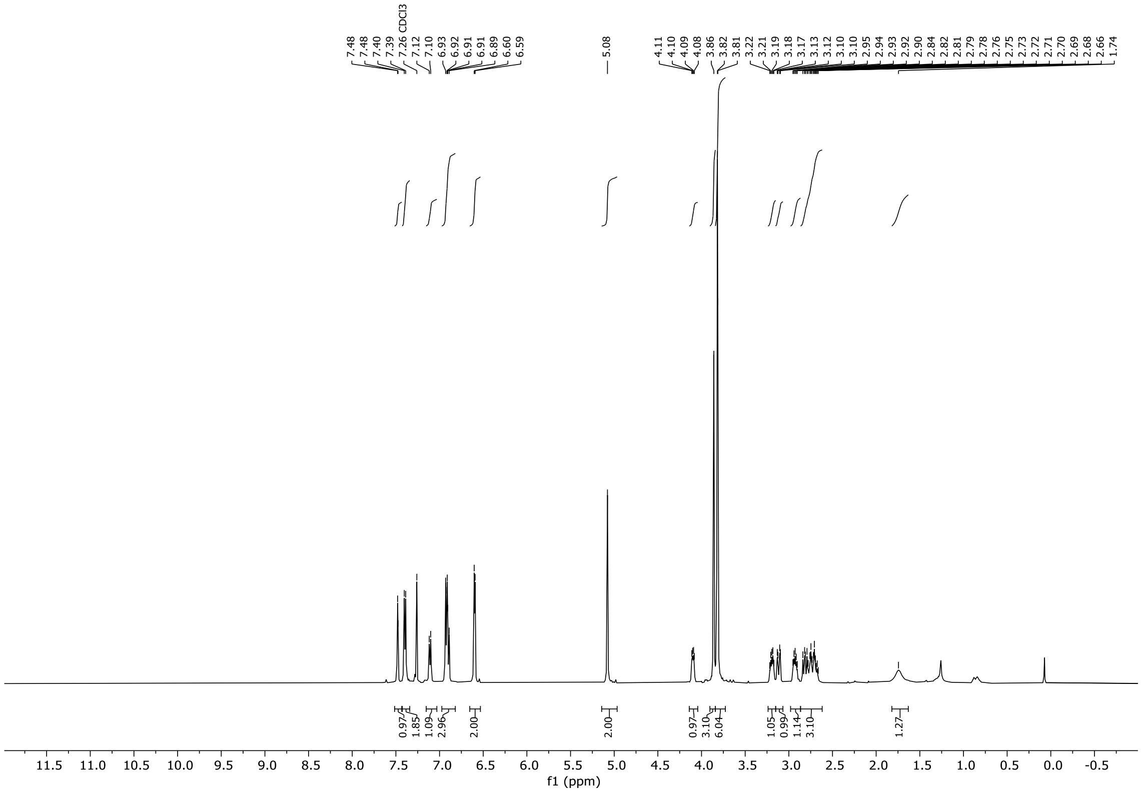

^{13}C NMR (126 MHz, CDCl$_3$) of ***(R)*-8c**

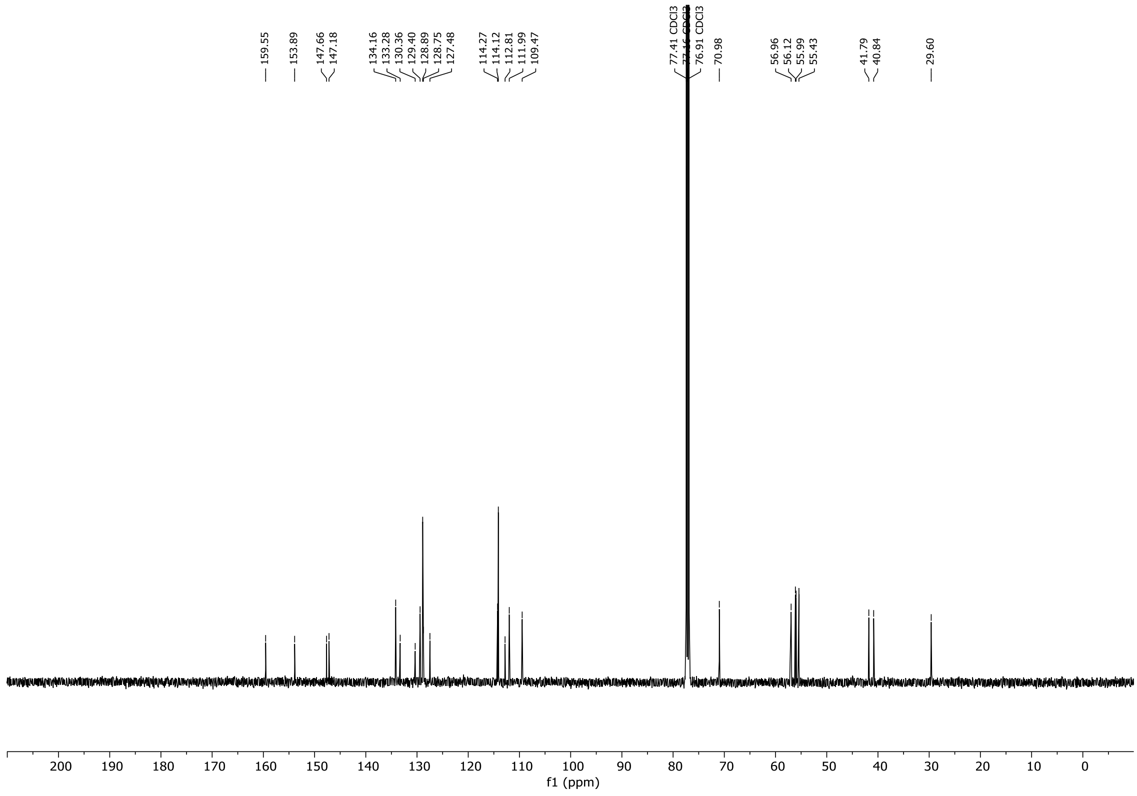

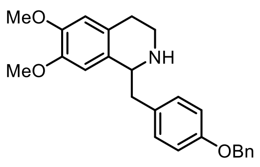

^{1}**H NMR** (500 MHz, CDCl$_3$) of **rac-8d**

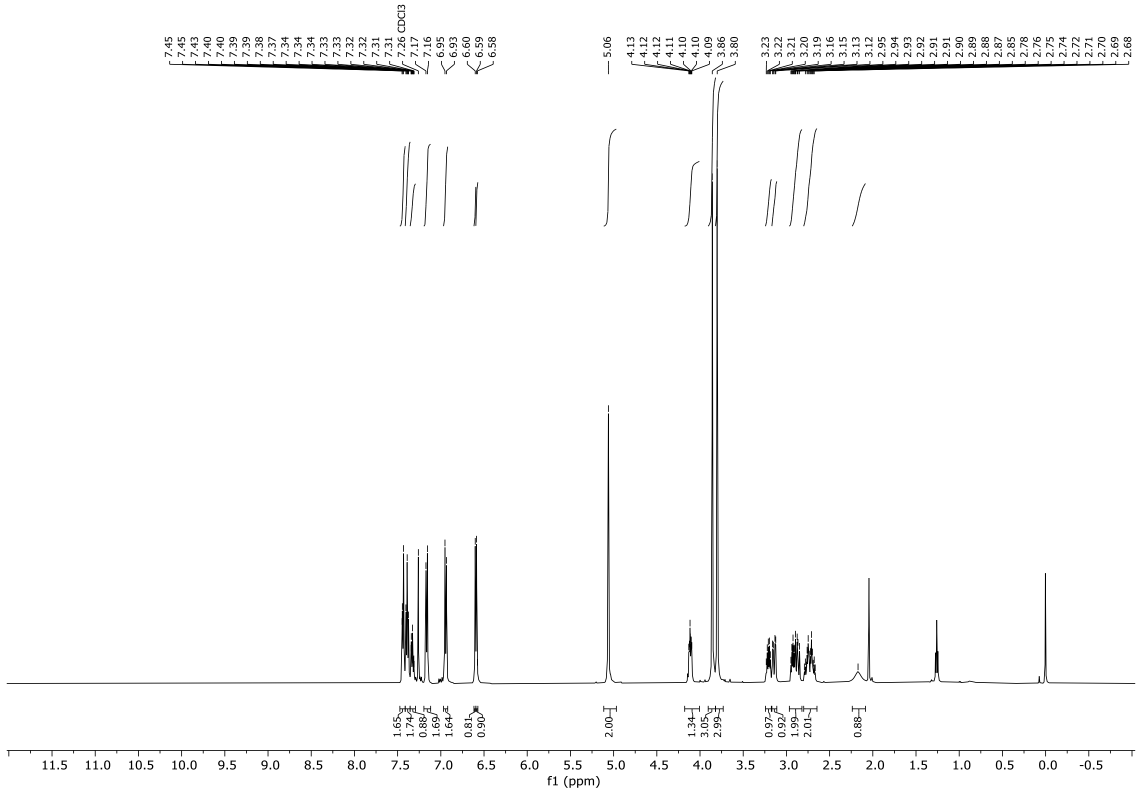

^{13}**C NMR** (126 MHz, CDCl$_3$) of **rac-8d**

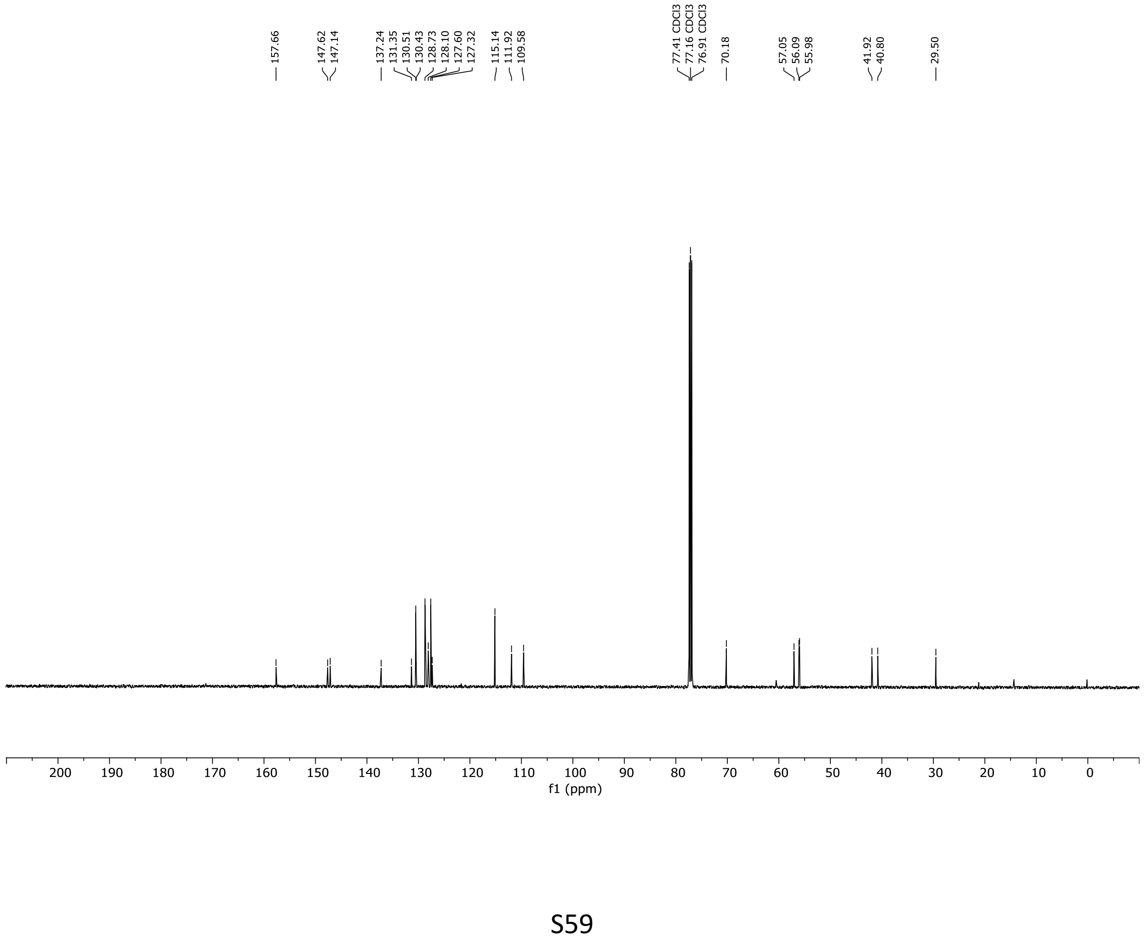

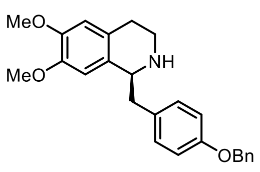

^{1}H NMR (500 MHz, $CDCl_3$) of **(*S*)-8d**

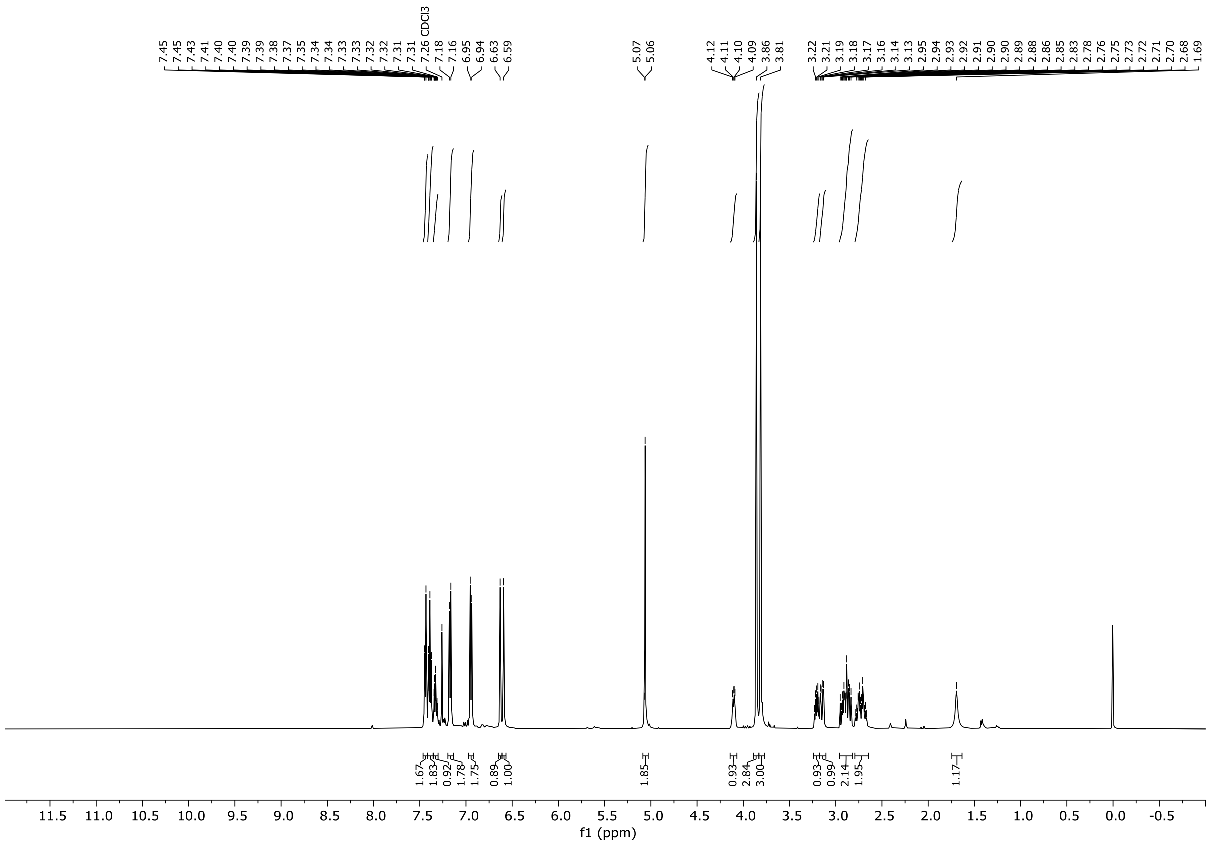

^{13}C NMR (126 MHz, $CDCl_3$) of **(*S*)-8d**

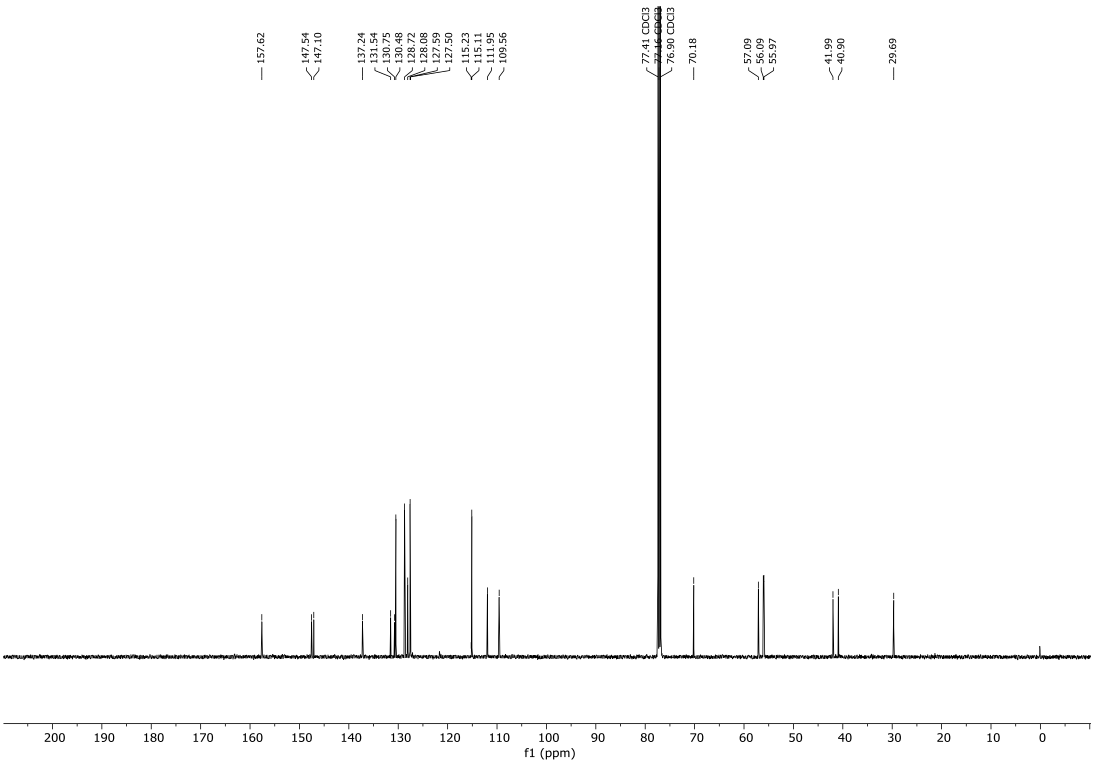

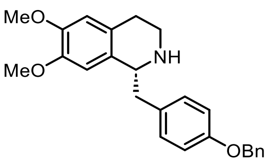

^{1}H NMR (500 MHz, $CDCl_3$) of **(*R*)-8d**

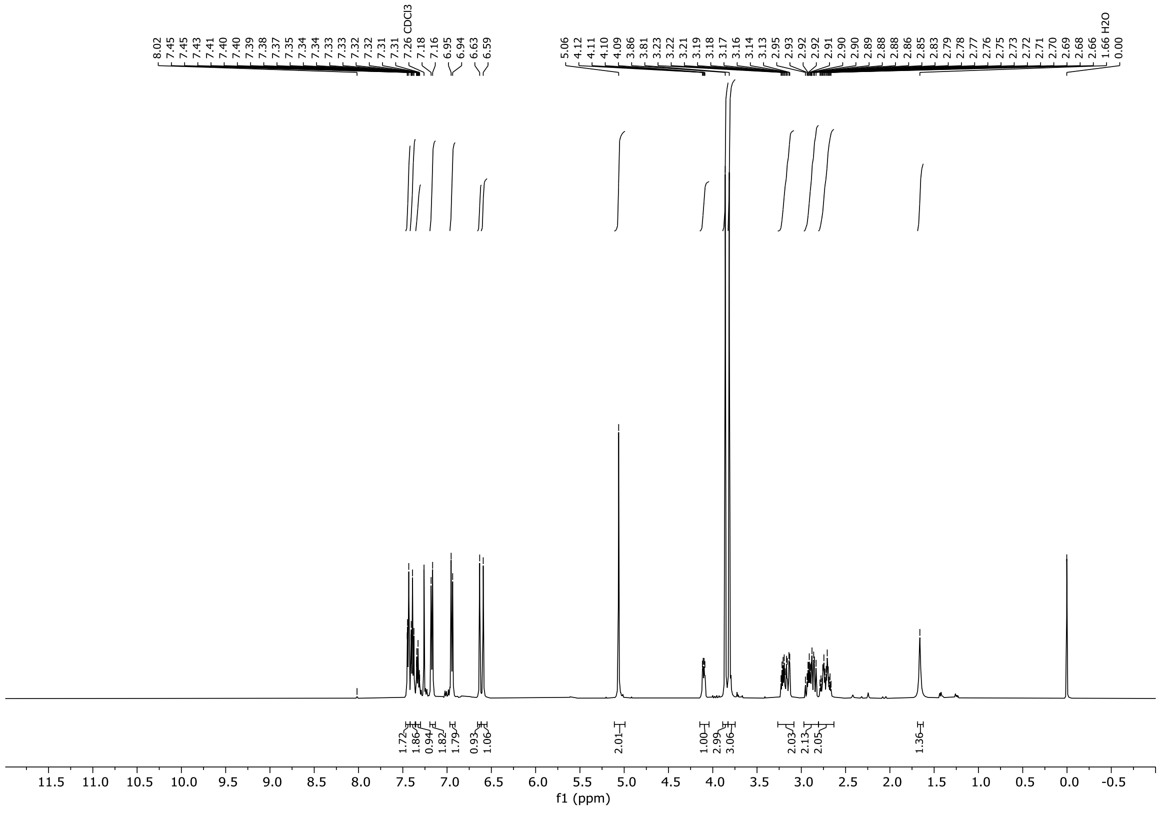

^{13}C NMR (126 MHz, $CDCl_3$) of **(*R*)-8d**

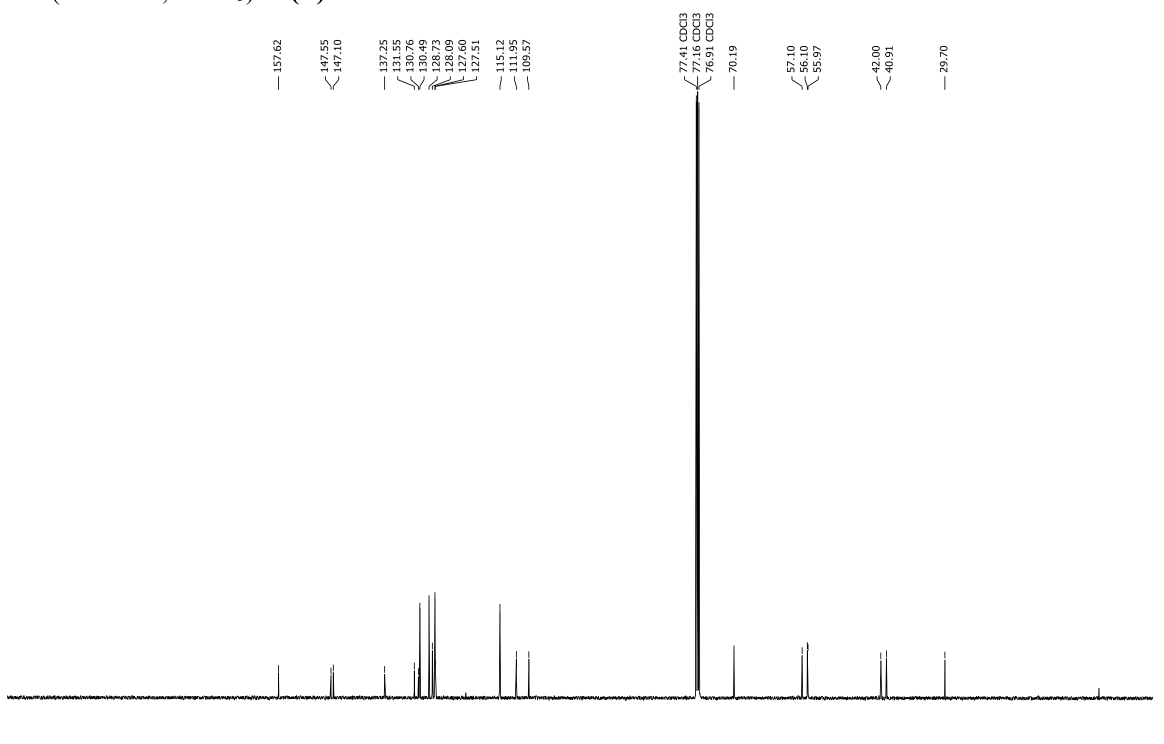

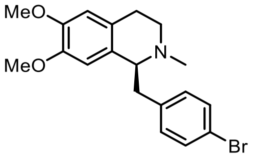

^{1}**H NMR** (400 MHz, $CDCl_3$) of **(*S*)-9a**

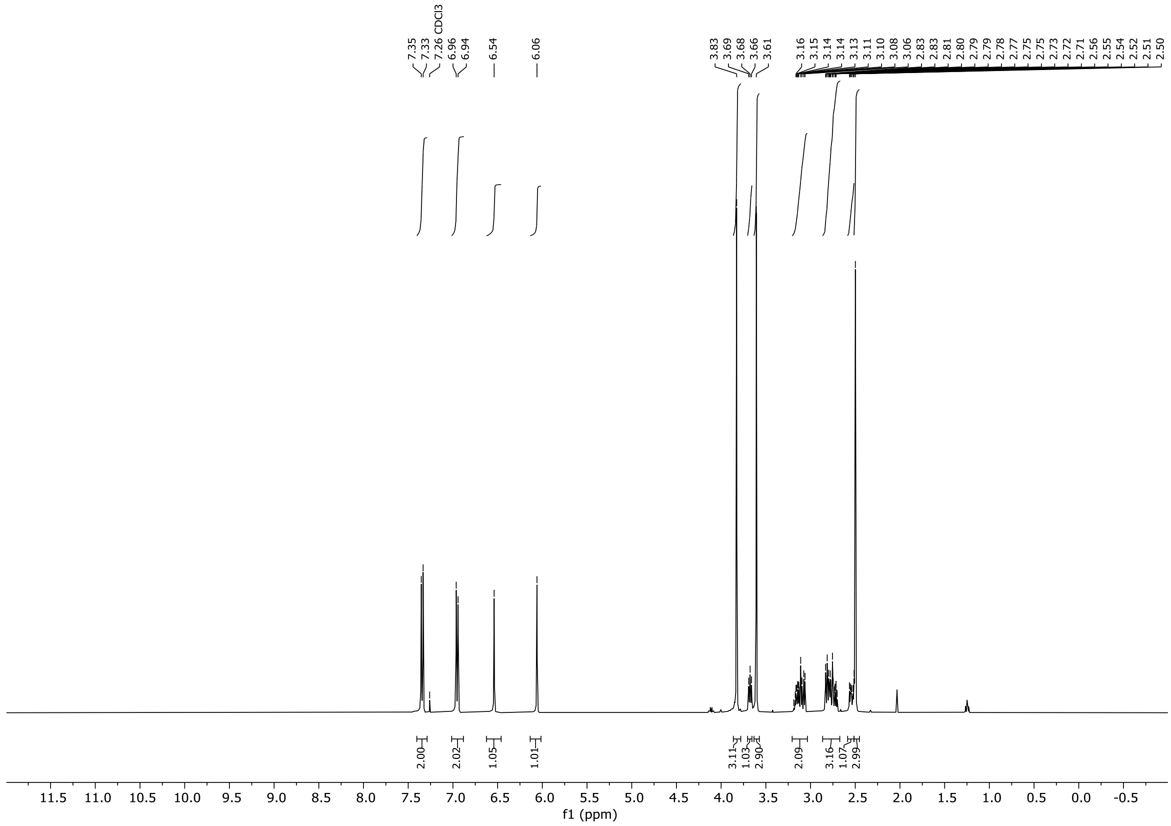

^{13}**C NMR** (101 MHz, $CDCl_3$) of **(*S*)-9a**

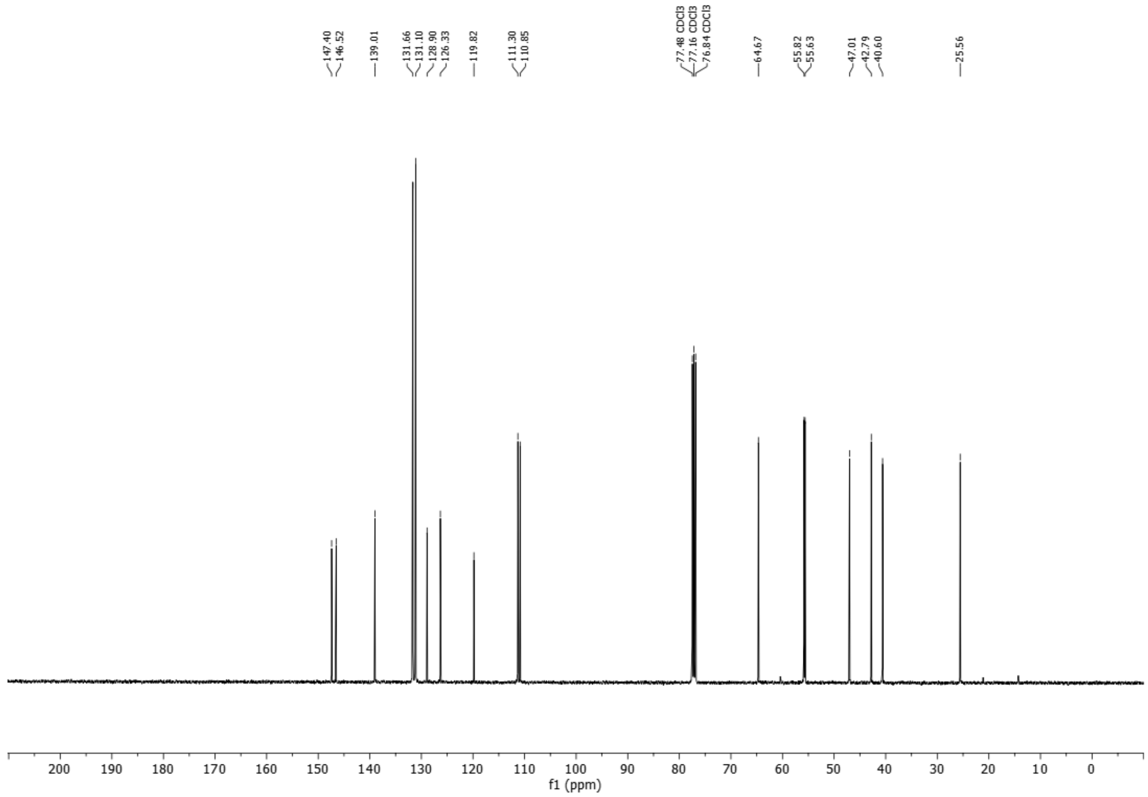

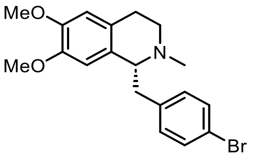

¹H NMR (500 MHz, $CDCl_3$) of **(*R*)-9a**

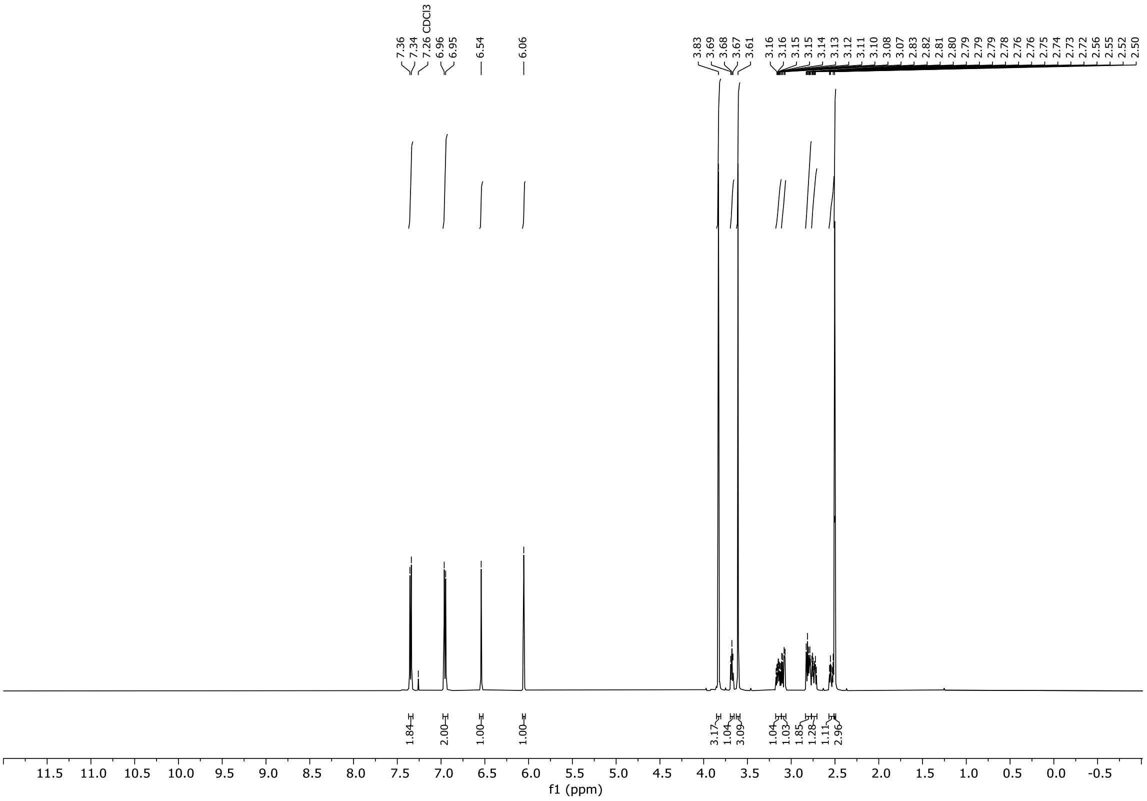

¹³C NMR (126 MHz, $CDCl_3$) of **(*R*)-9a**

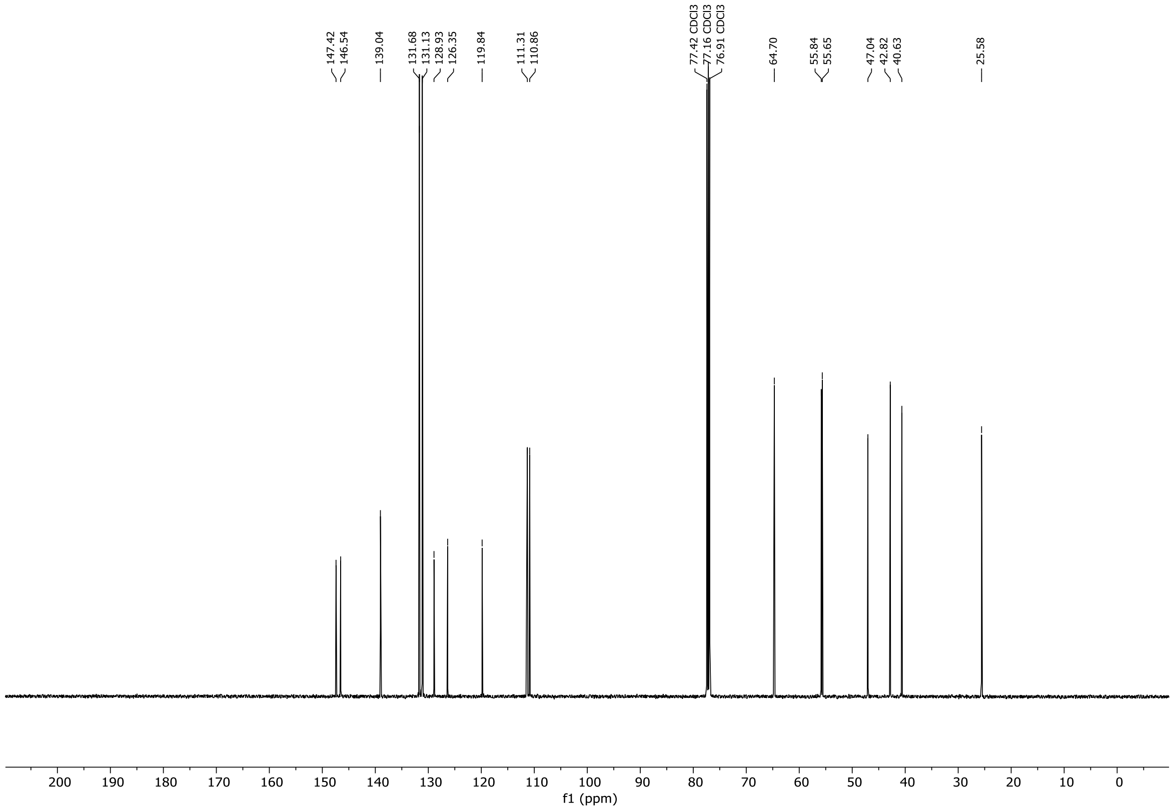

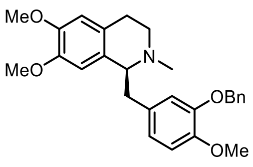

^{1}H NMR (500 MHz, $CDCl_3$) of **(*S*)-9b**

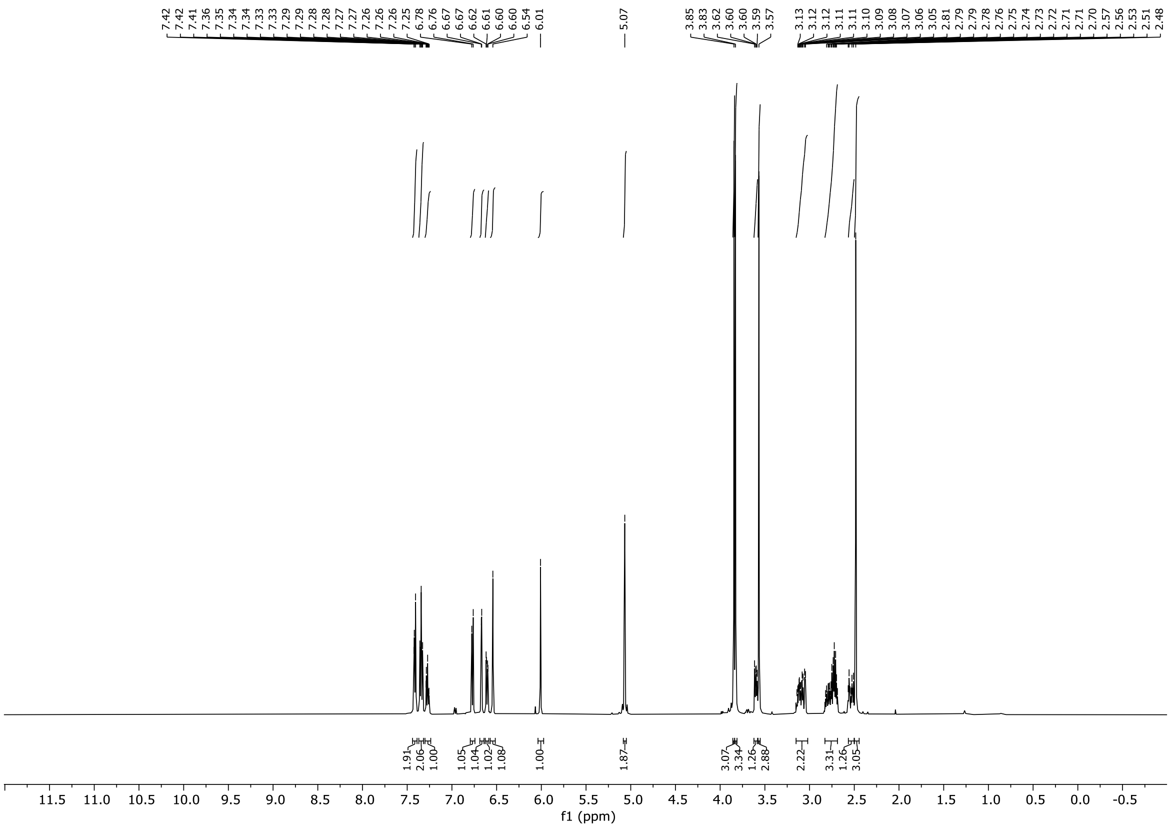

^{13}C NMR (126 MHz, $CDCl_3$) of **(*S*)-9b**

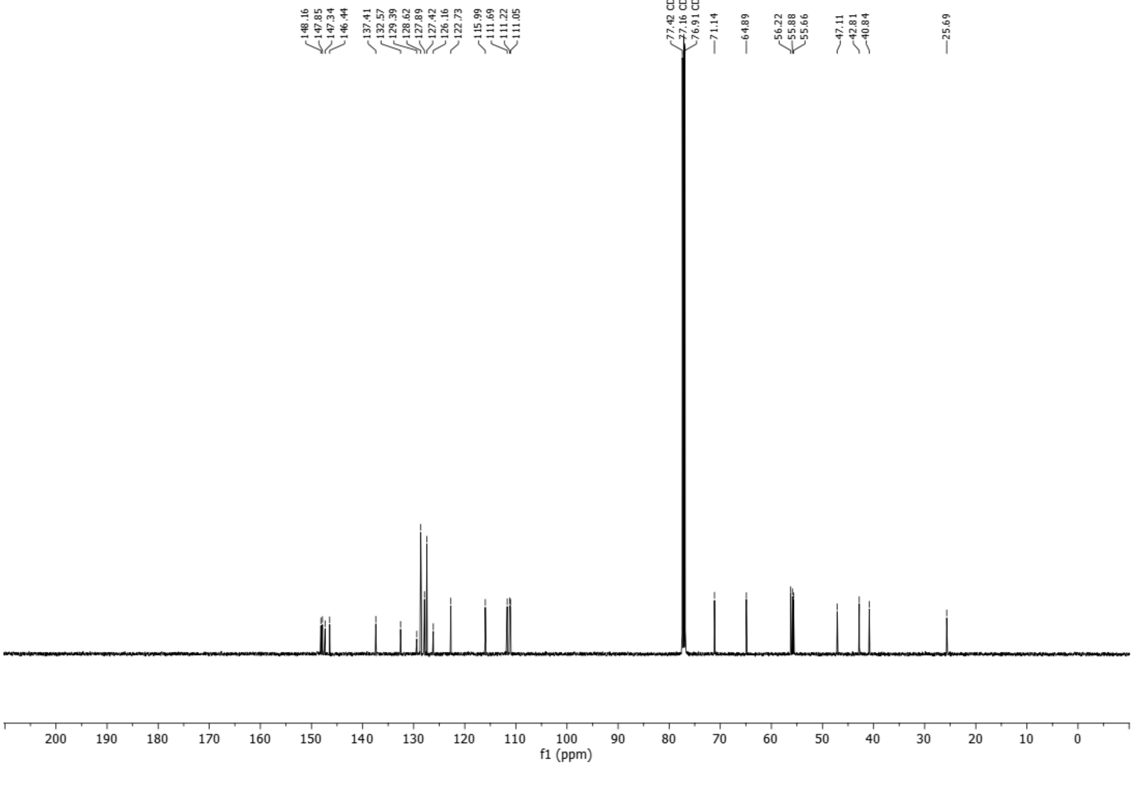

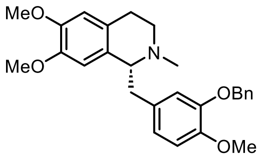

^{1}H NMR (400 MHz, $CDCl_3$) of ***(R)*-9b**

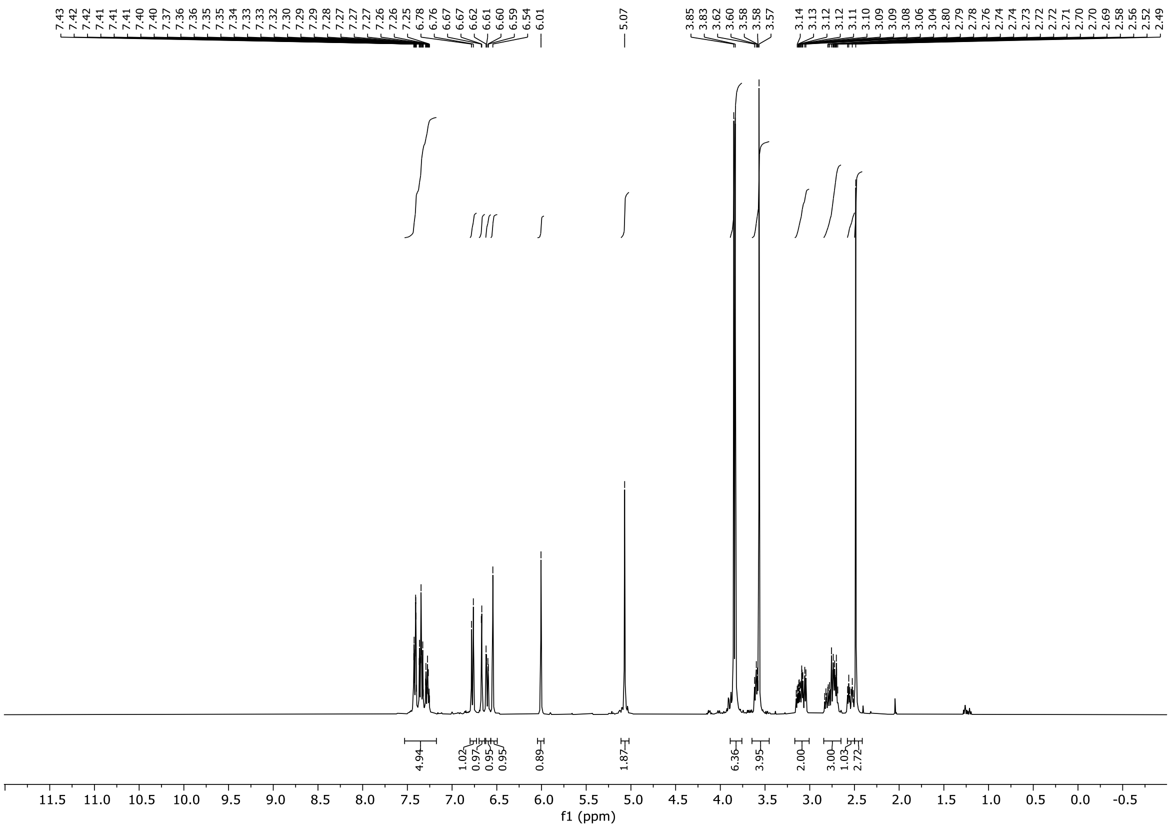

^{13}C NMR (101 MHz, $CDCl_3$) of ***(R)*-9b**

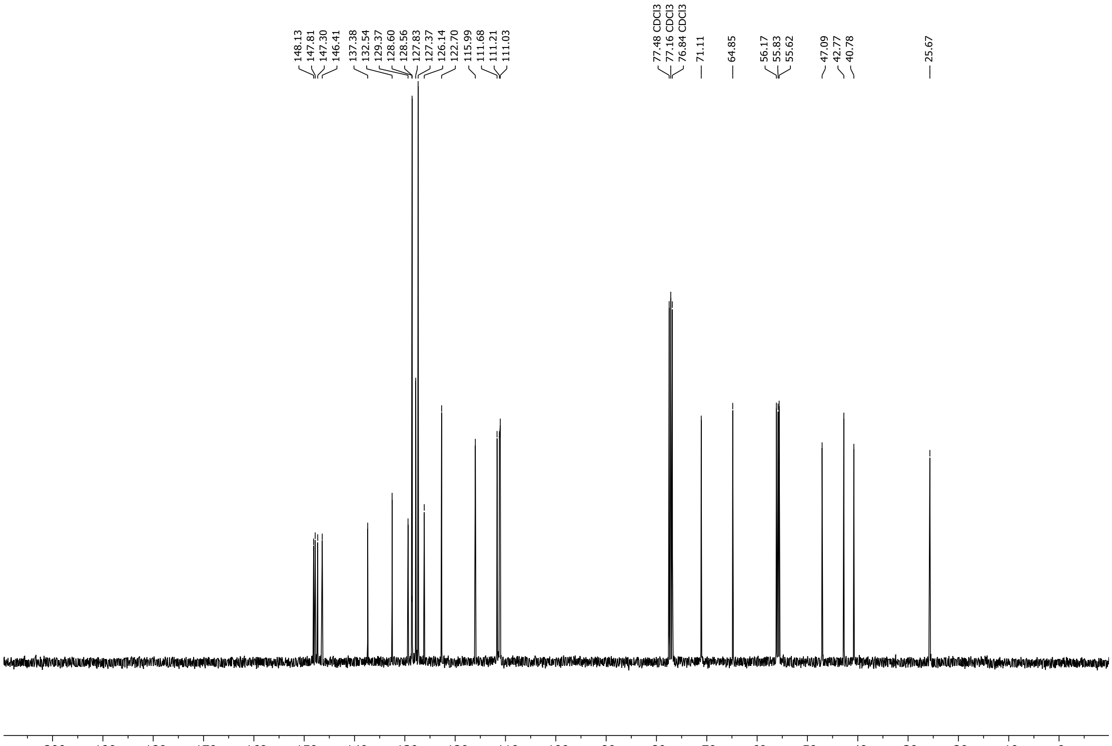

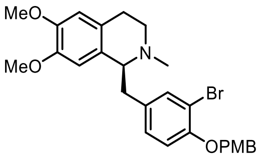

^{1}H NMR (500 MHz, $CDCl_3$) of **(*S*)-9c**

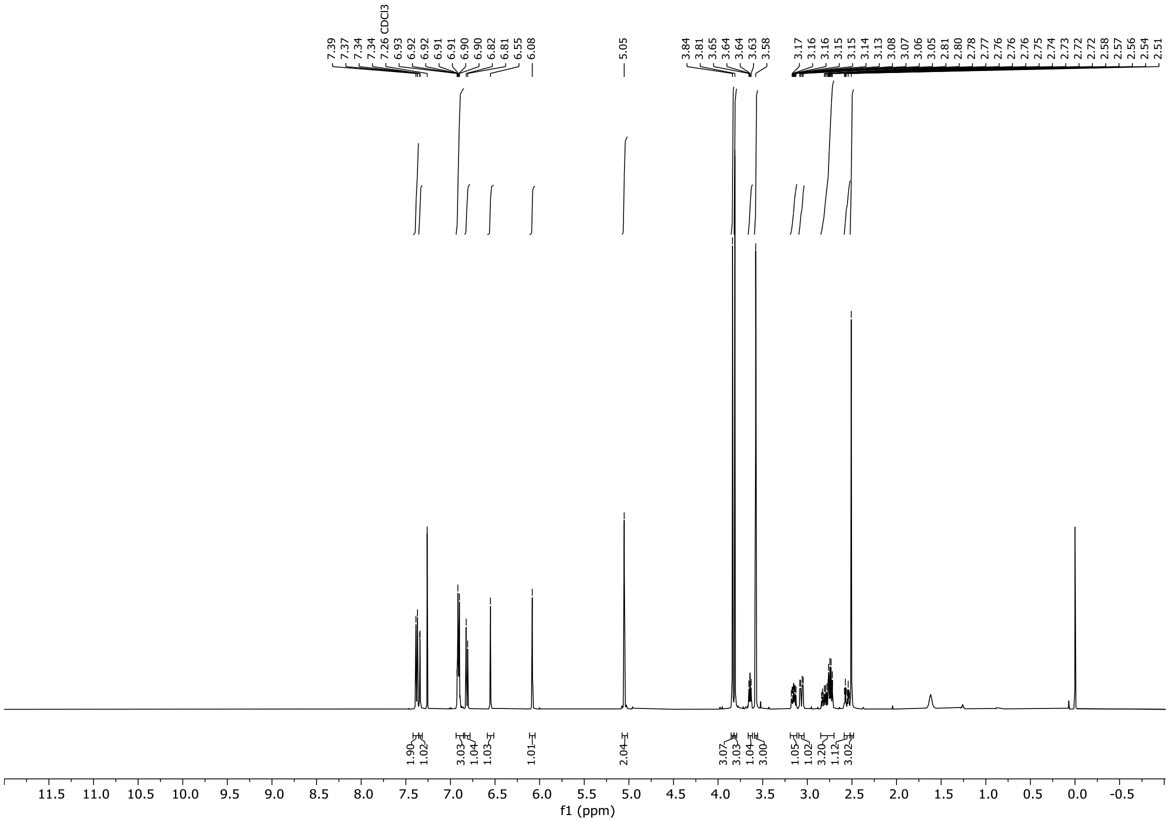

^{13}C NMR (126 MHz, $CDCl_3$) of **(*S*)-9c**

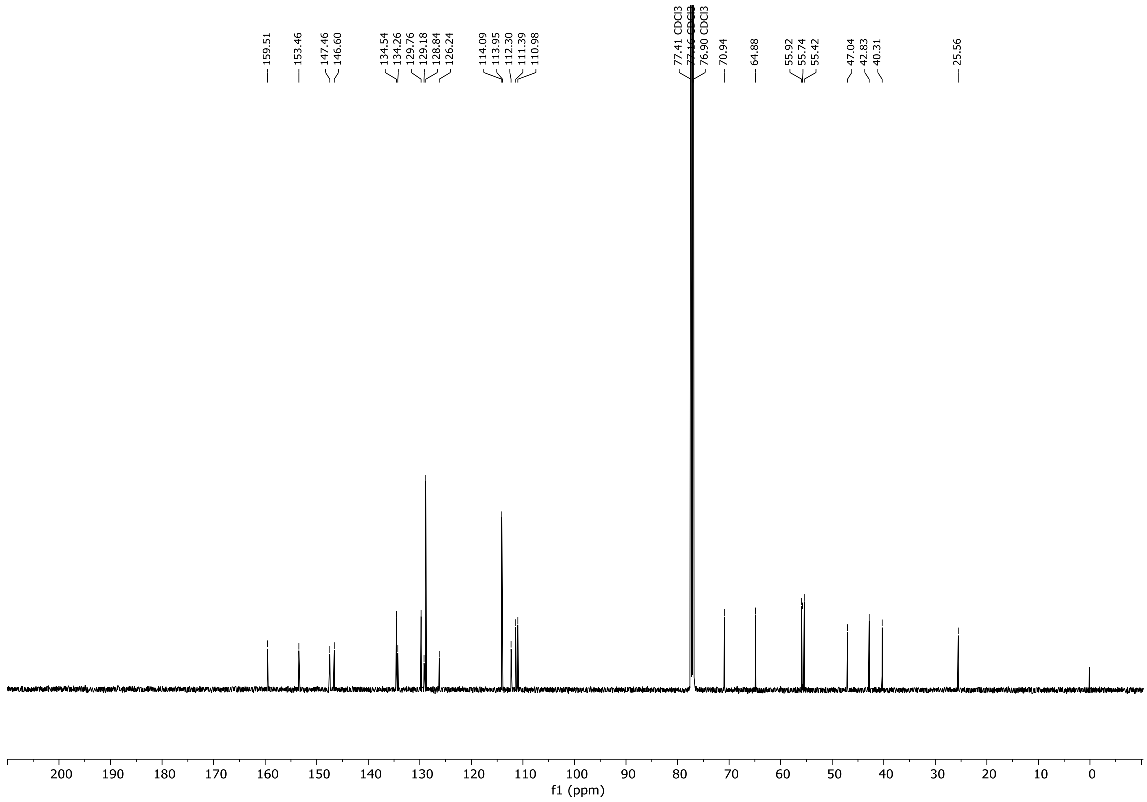

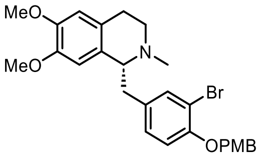

^{1}H NMR (500 MHz, CDCl$_3$) of **(*R*)-9c**

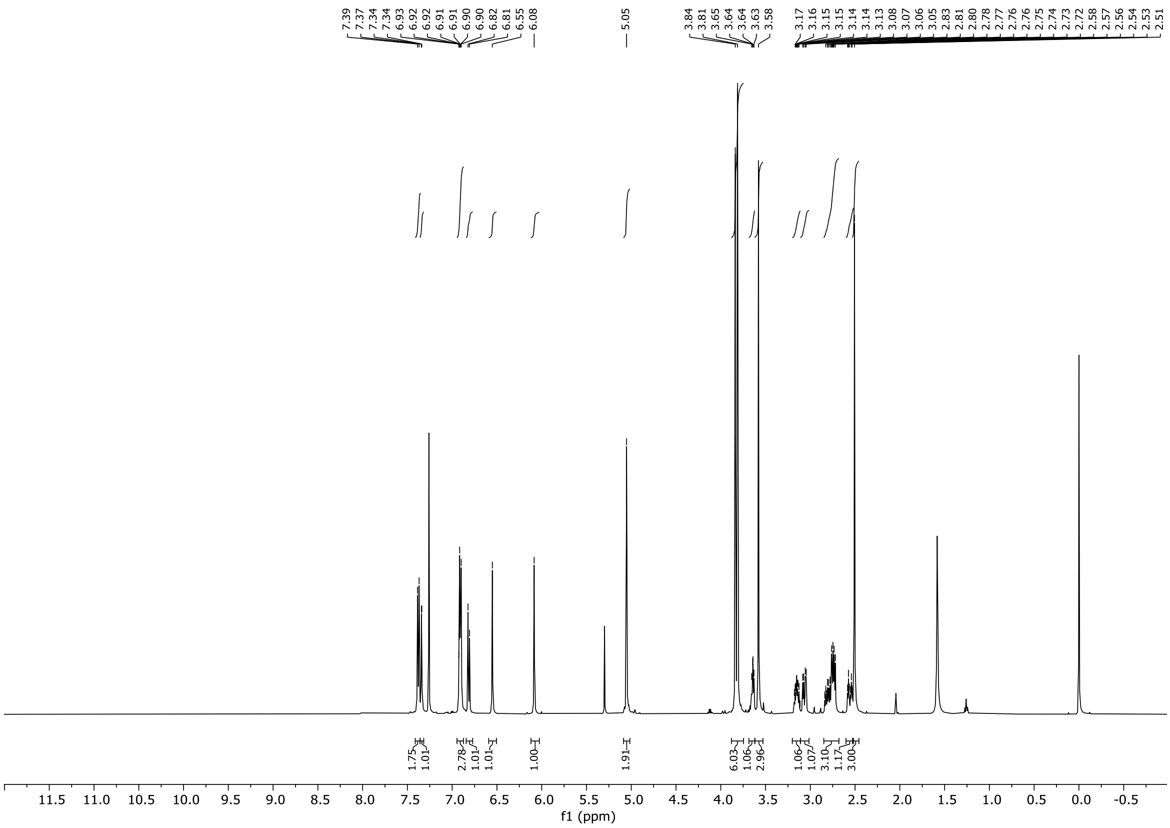

^{13}C NMR (126 MHz, CDCl$_3$) of **(*R*)-9c**

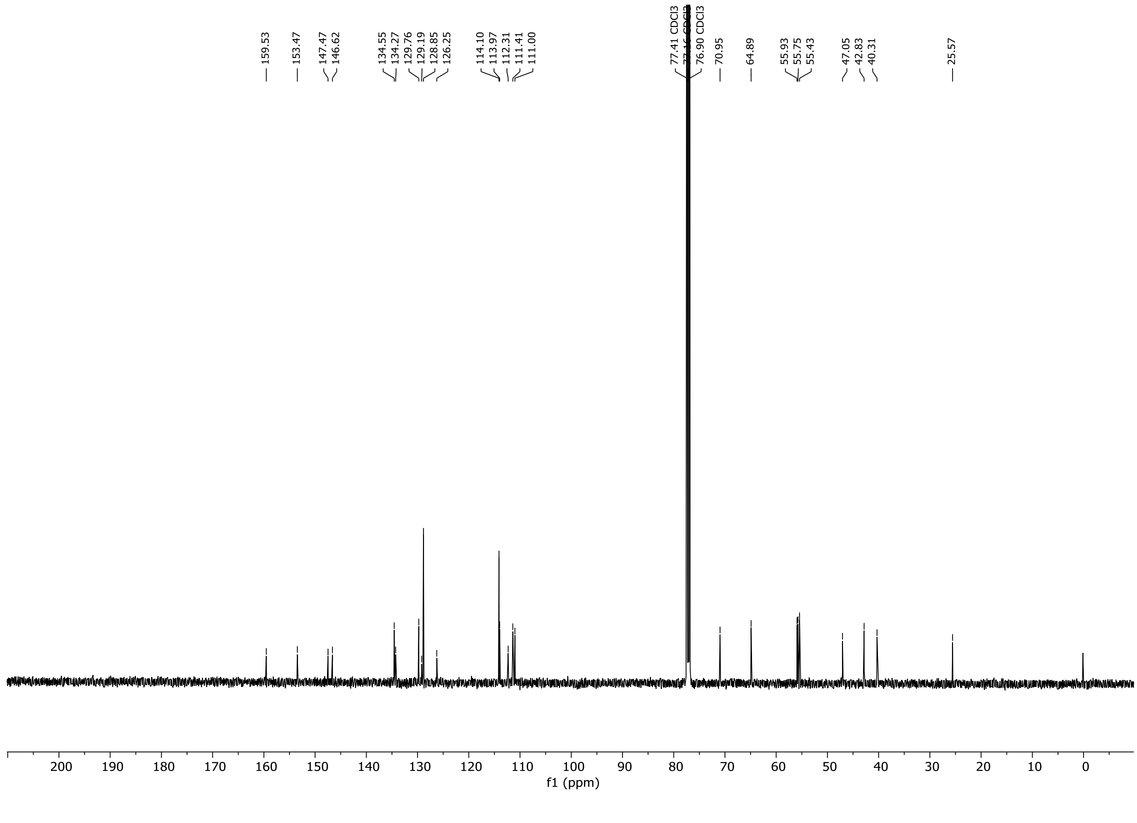

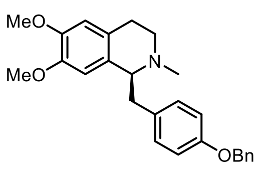

[1]**H NMR** (500 MHz, $CDCl_3$) of ***(S)*-9d**

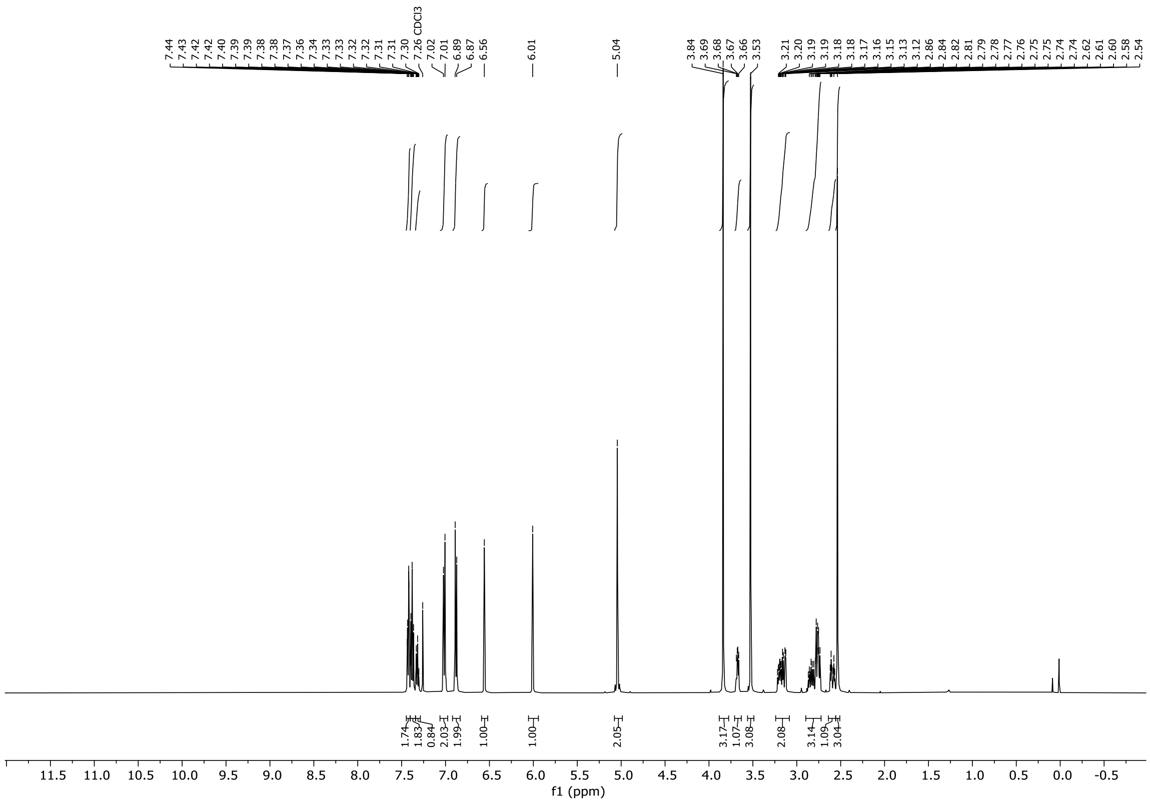

[13]**C NMR** (126 MHz, $CDCl_3$) of ***(S)*-9d**

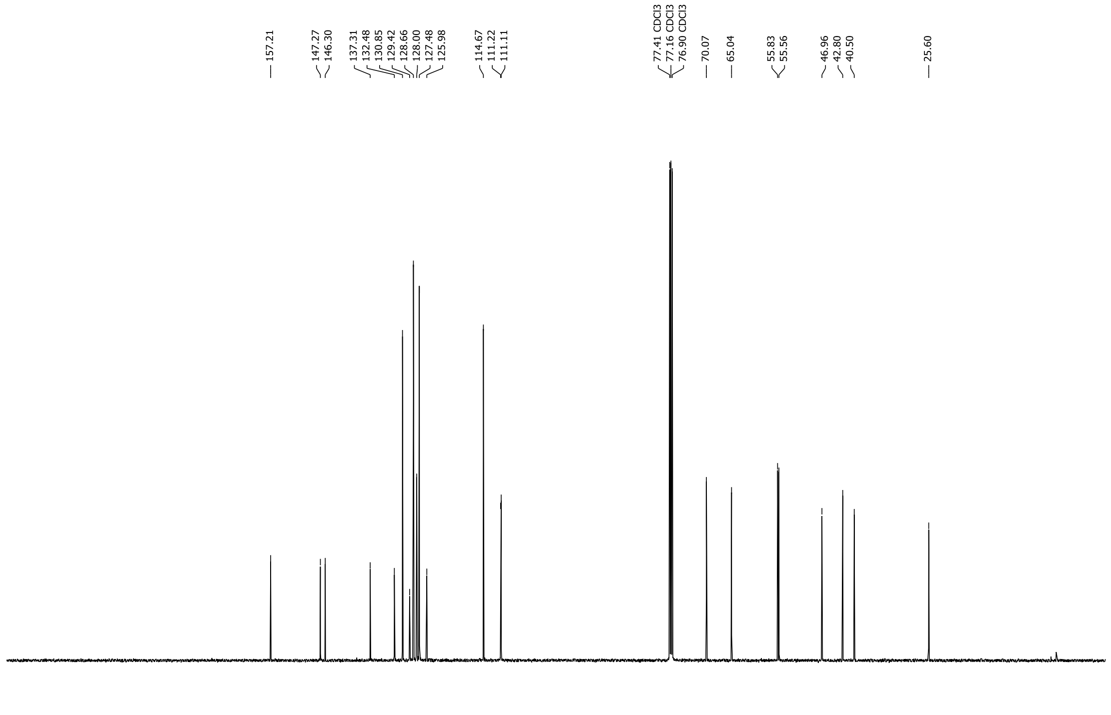

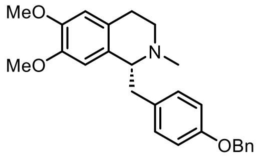

^{1}**H NMR** (500 MHz, $CDCl_3$) of **(*R*)-9d**

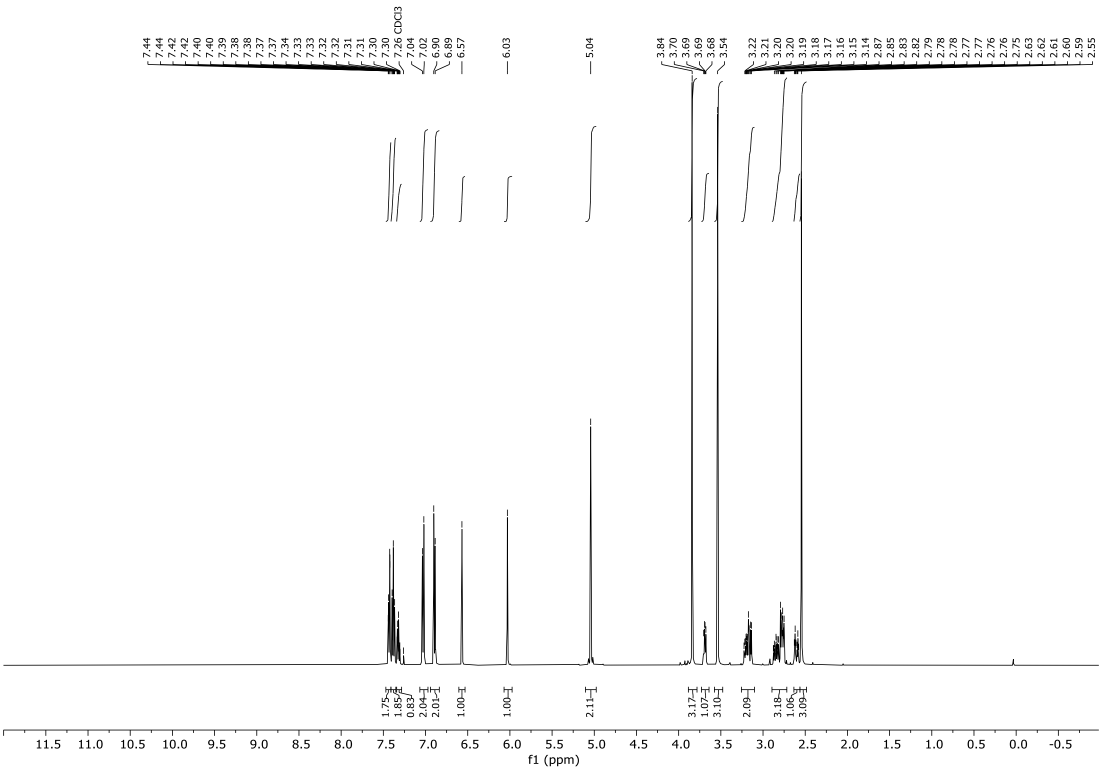

^{13}**C NMR** (126 MHz, $CDCl_3$) of **(*R*)-9d**

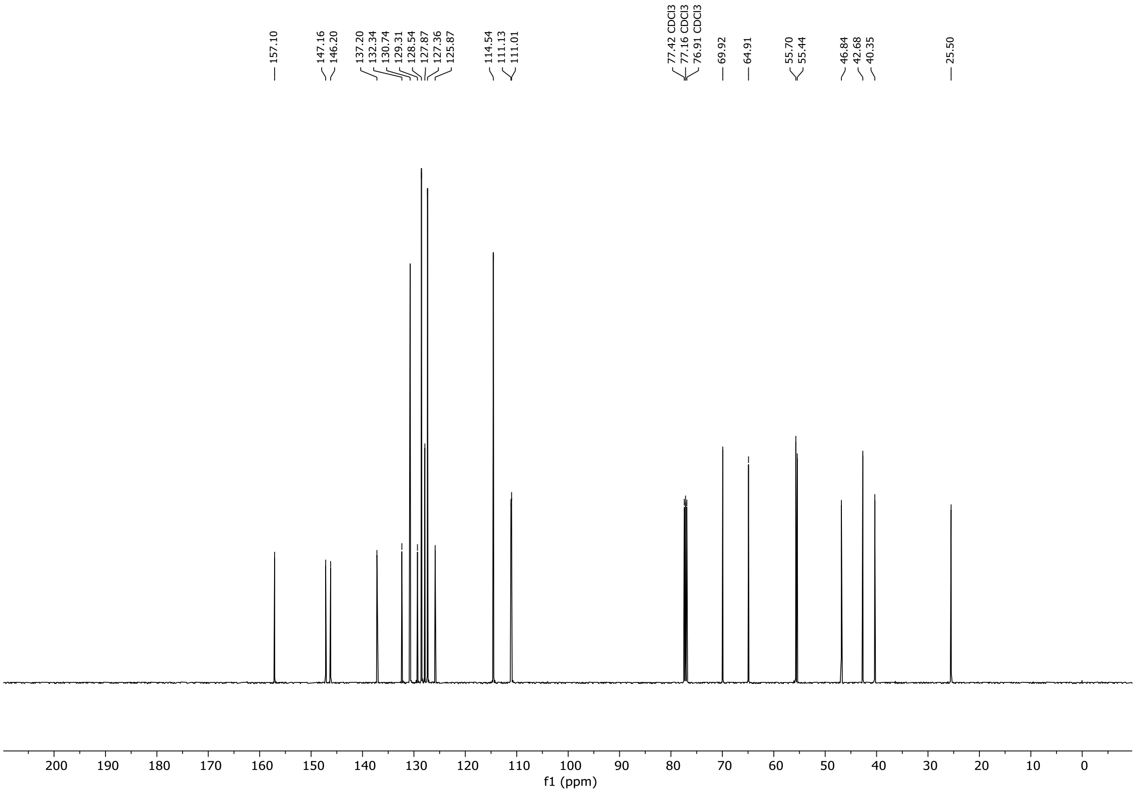

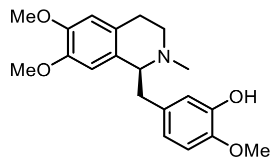

^{1}H NMR (500 MHz, $CDCl_3$) of ***(S)*-10a**

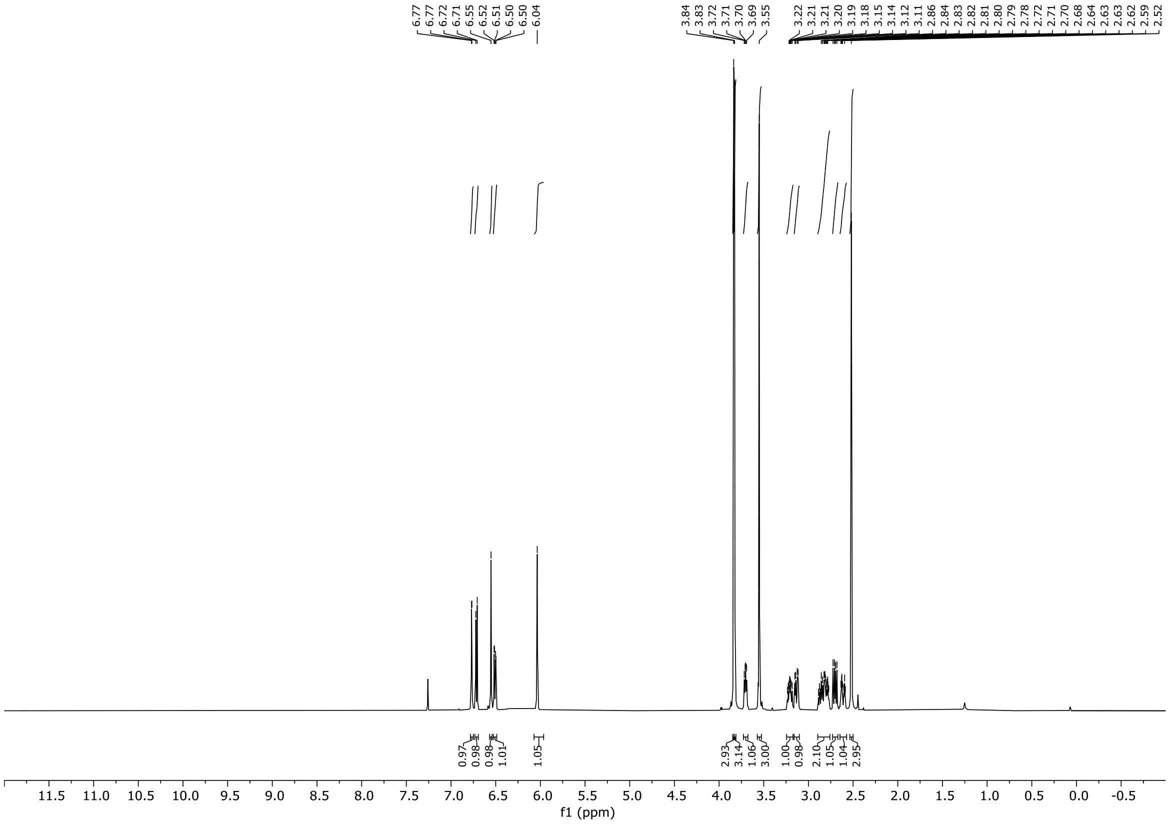

^{13}C NMR (126 MHz, $CDCl_3$) of ***(S)*-10a**

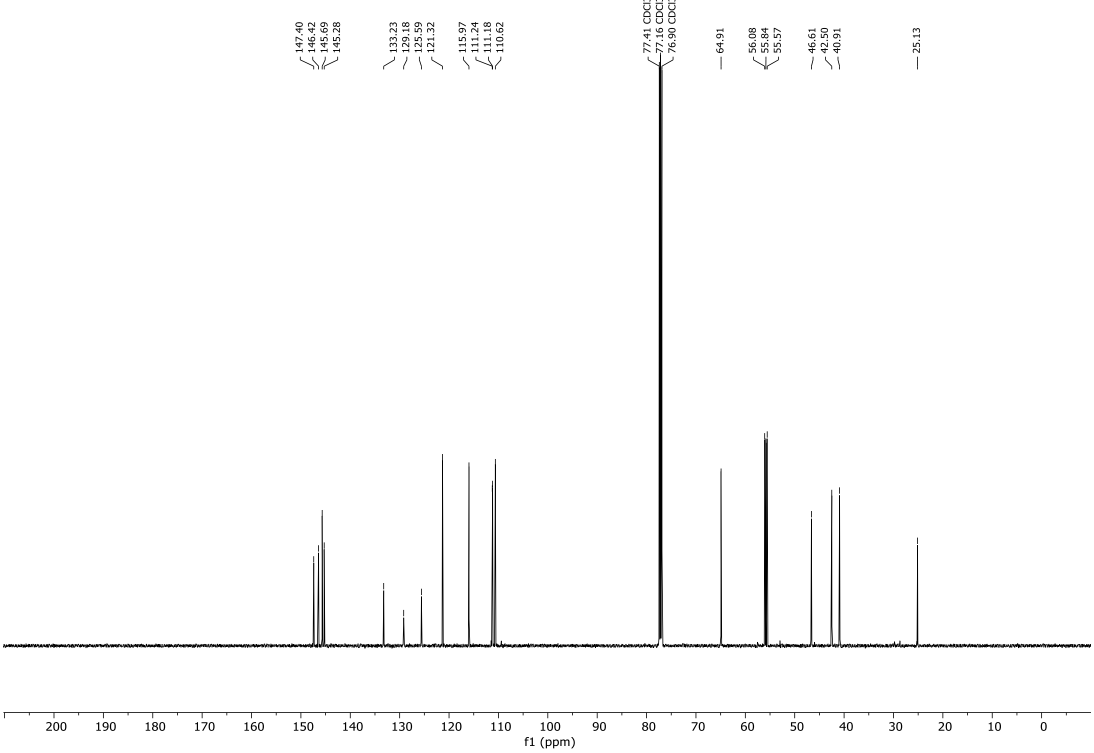

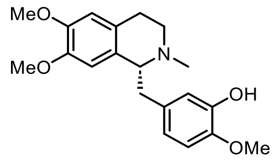

^{1}H NMR (500 MHz, CDCl$_3$) of ***(R)*-10a**

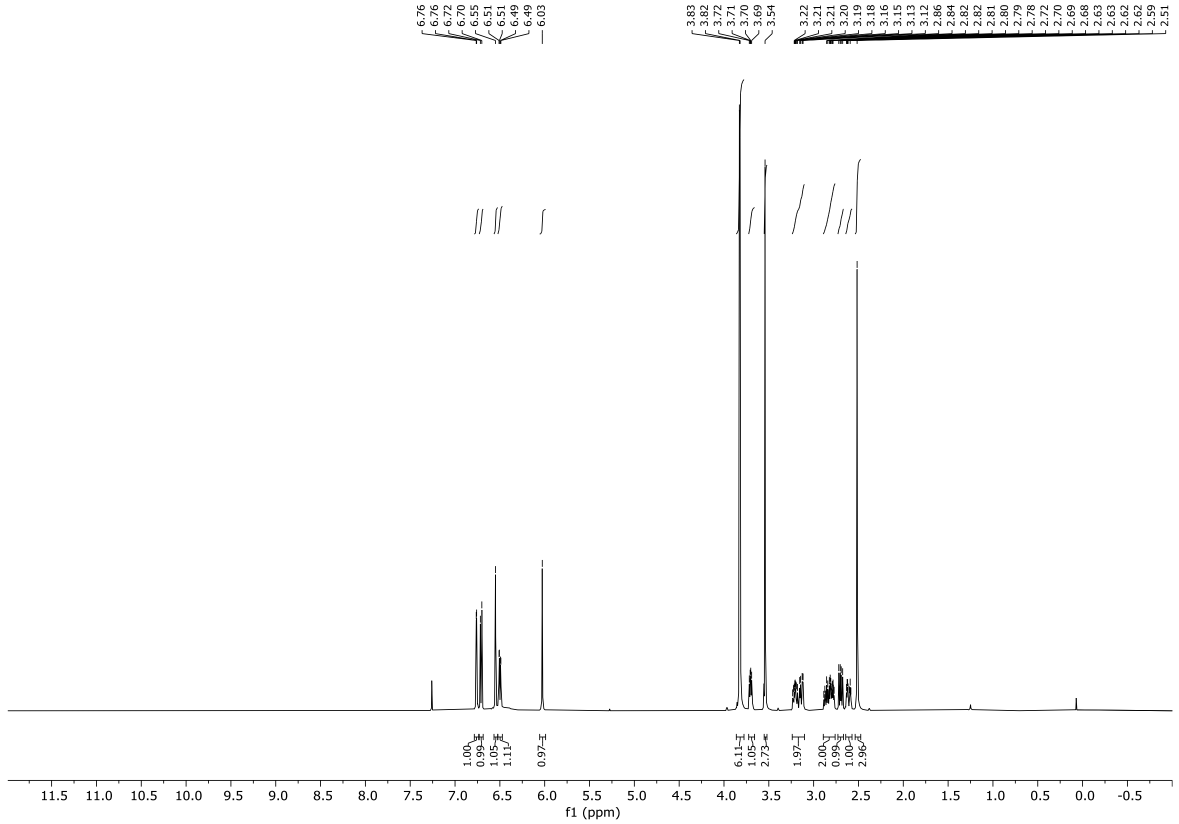

^{13}C NMR (126 MHz, CDCl$_3$) of ***(R)*-10a**

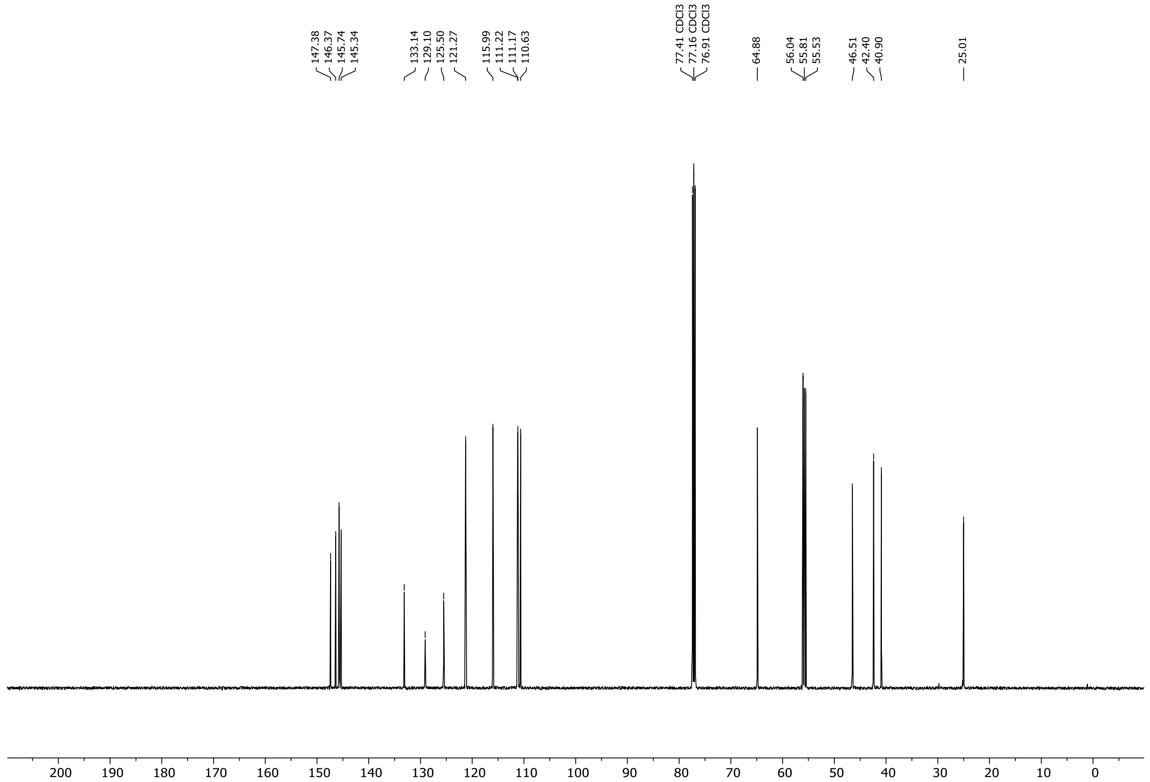

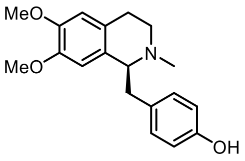

^{1}H NMR (500 MHz, $CDCl_3$) of ***(S)*-10b**

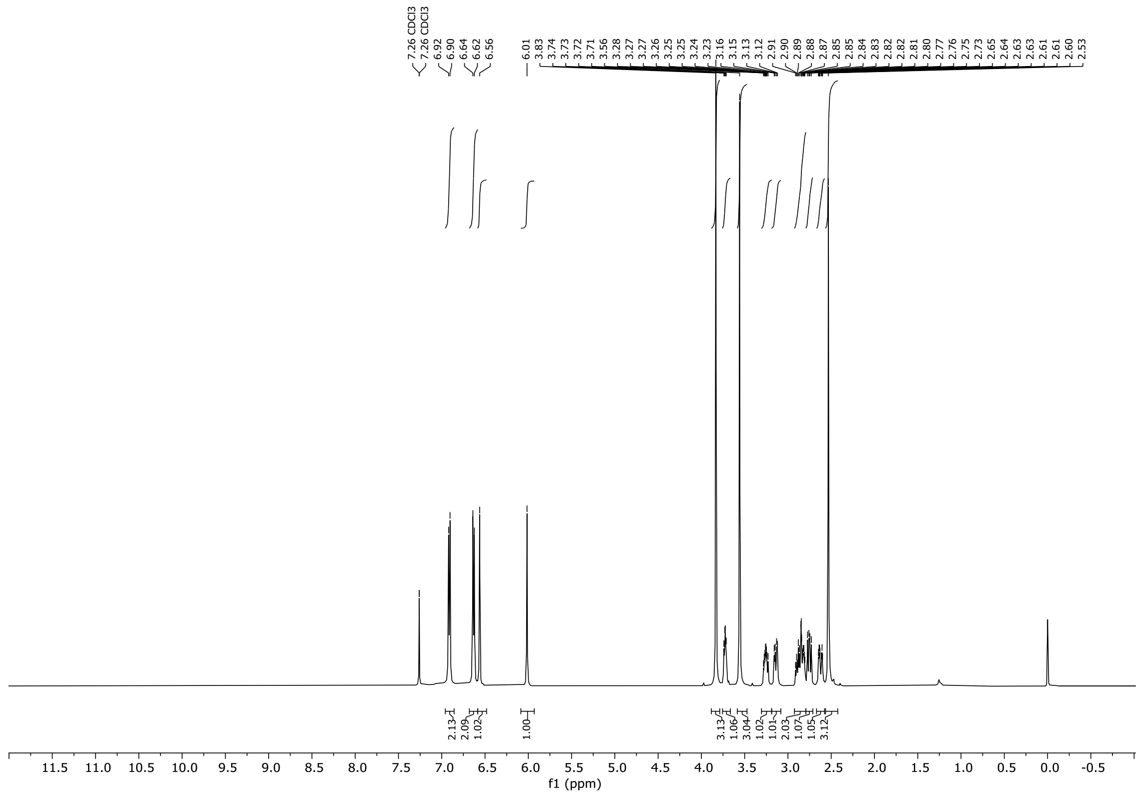

^{13}C NMR (126 MHz, $CDCl_3$) of ***(S)*-10b**

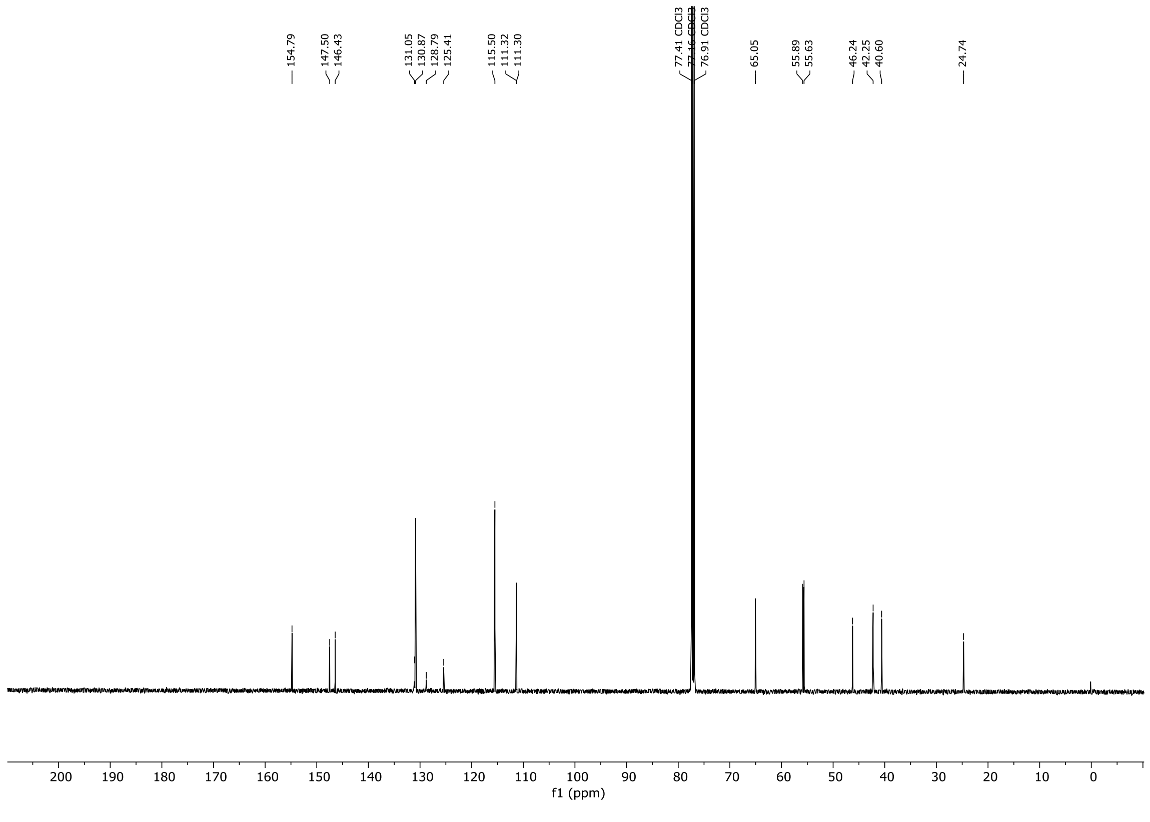

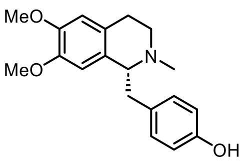

^{1}H NMR (500 MHz, $CDCl_3$) of **(*R*)-10b**

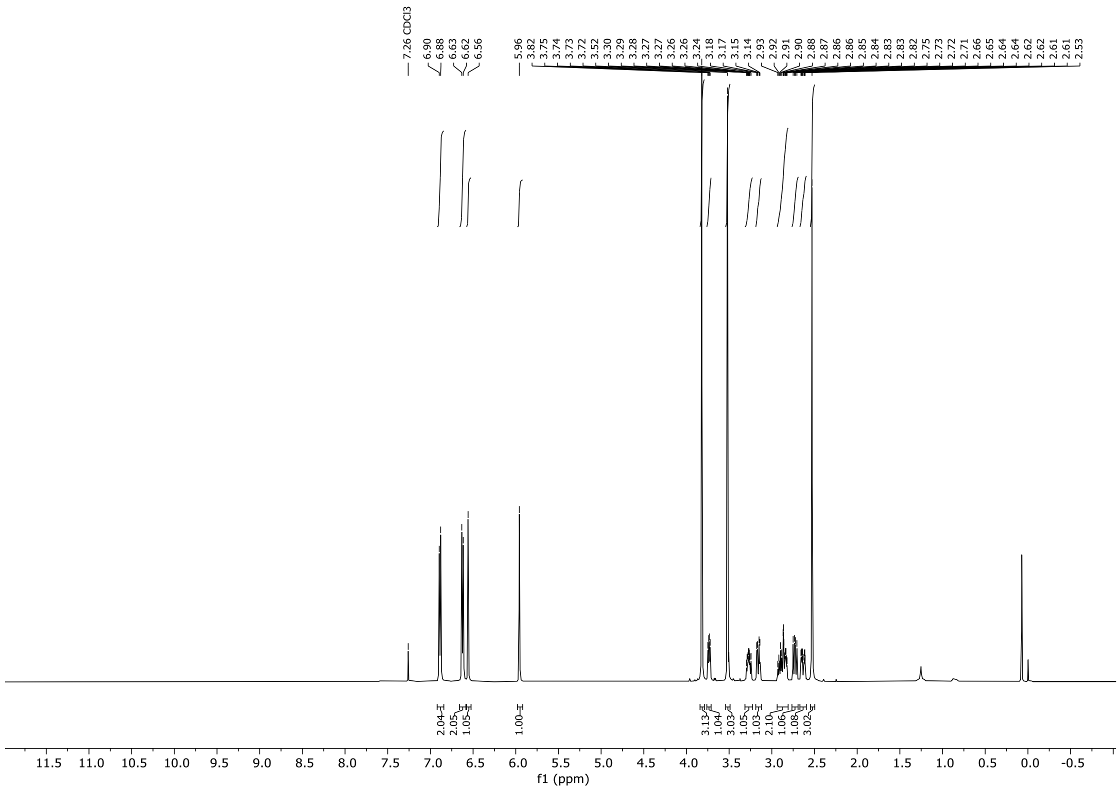

^{13}C NMR (126 MHz, $CDCl_3$) of **(*R*)-10b**

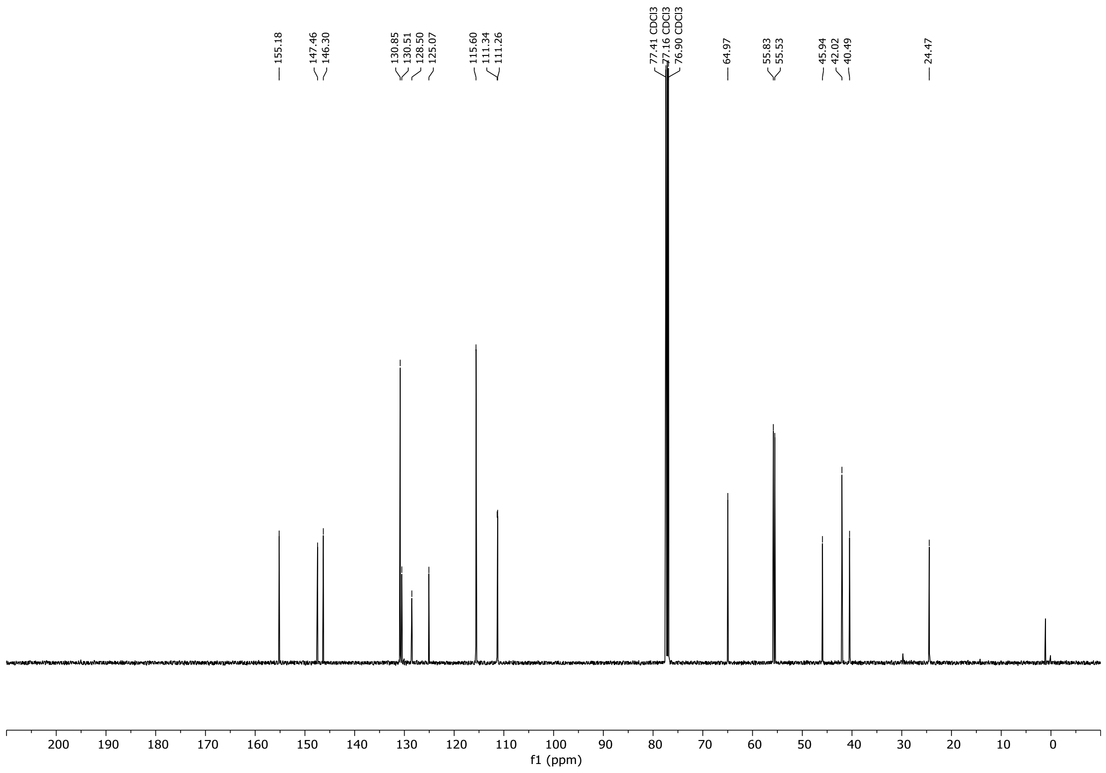

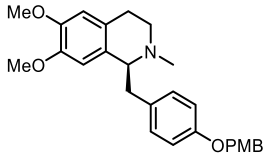

^{1}H NMR (500 MHz, $CDCl_3$) of ***(S)*-10c**

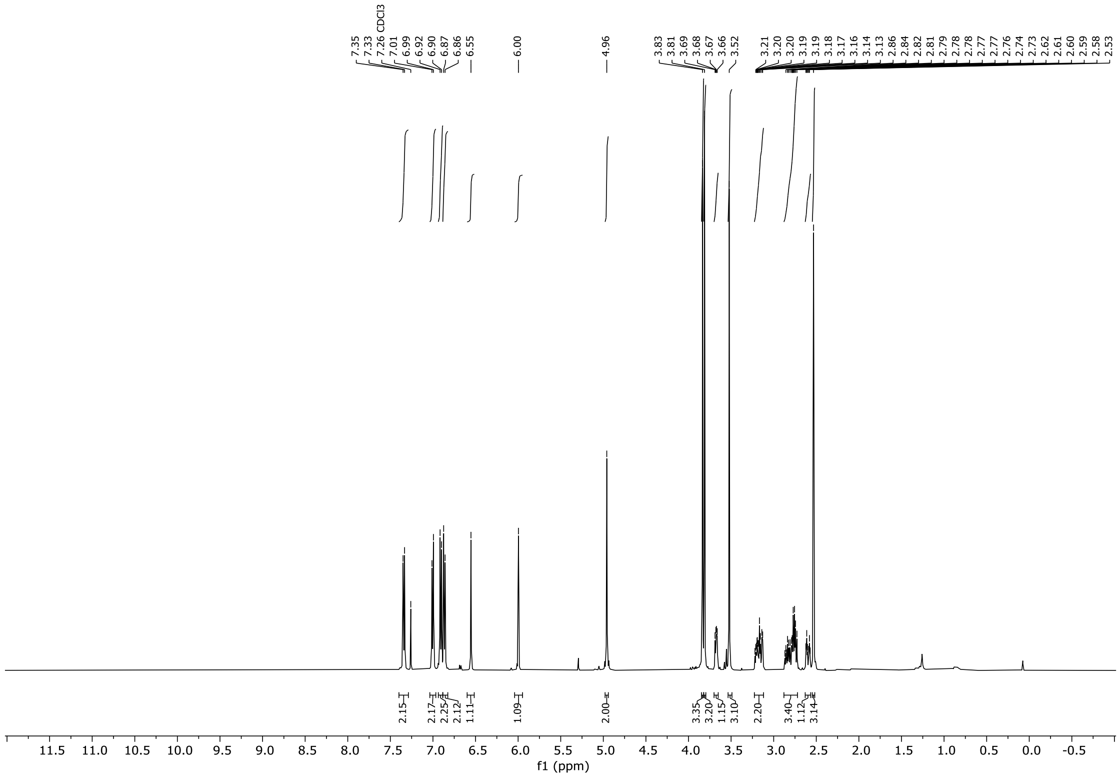

^{13}C NMR (126 MHz, $CDCl_3$) of ***(S)*-10c**

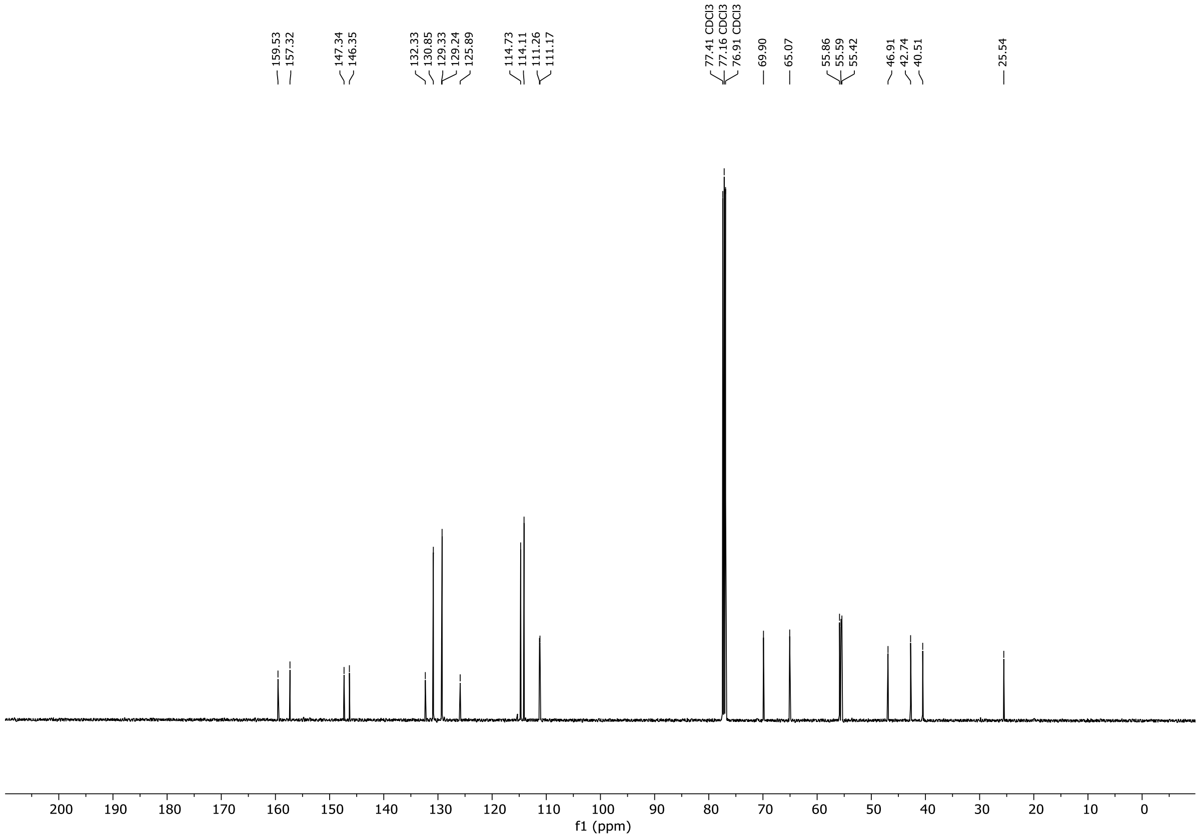

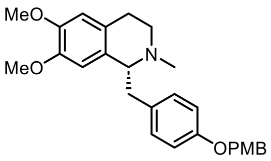

^{1}H NMR (500 MHz, $CDCl_3$) of ***(R)*-10c**

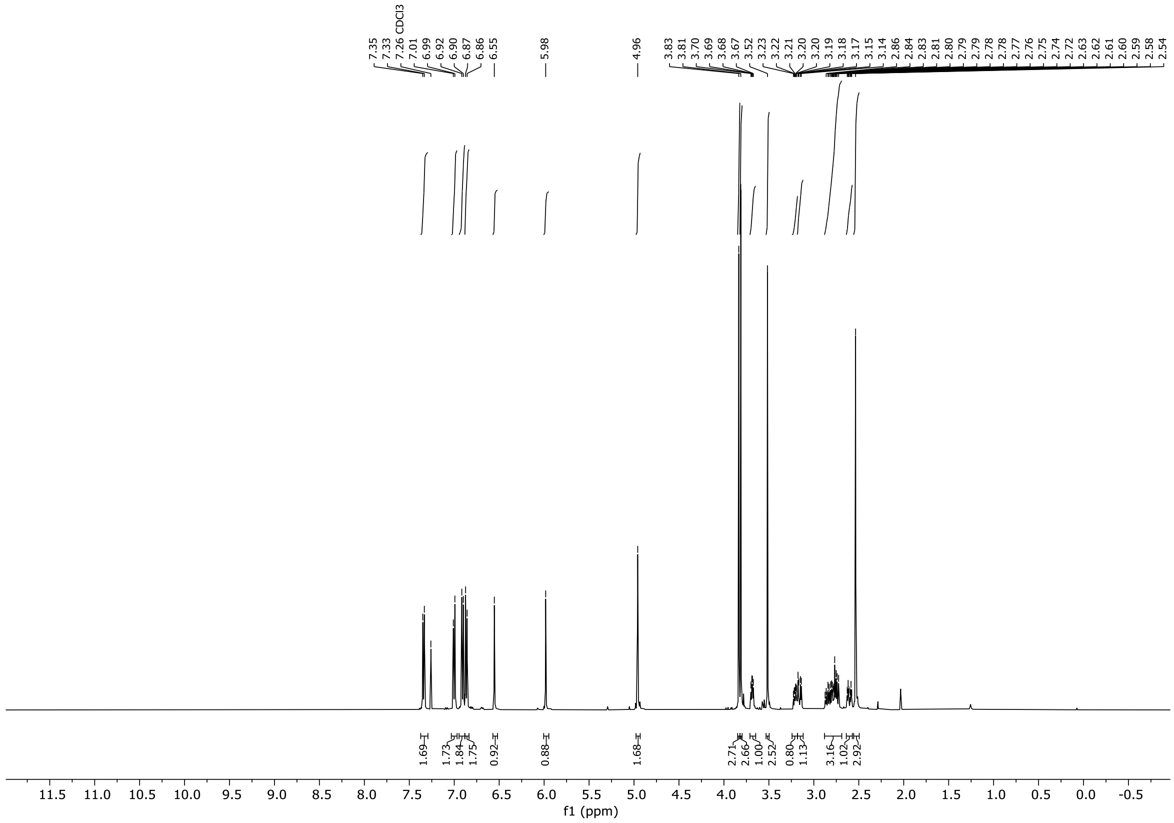

^{13}C NMR (126 MHz, $CDCl_3$) of ***(R)*-10c**

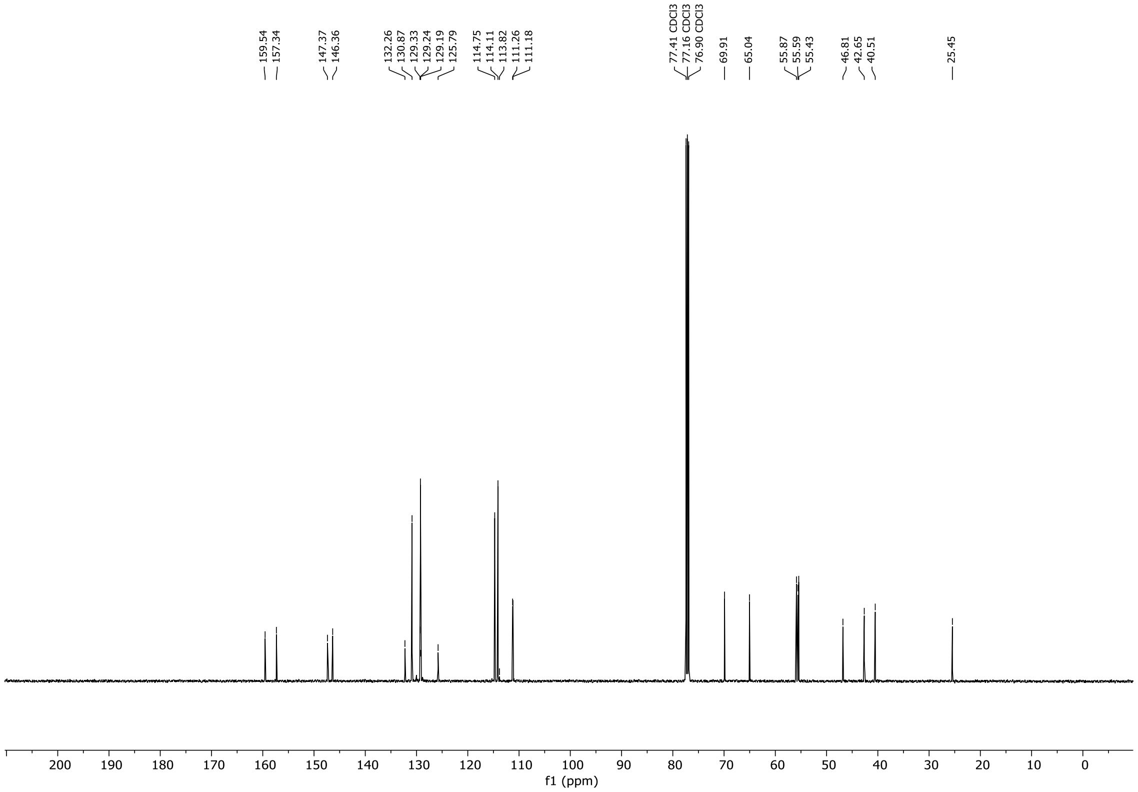

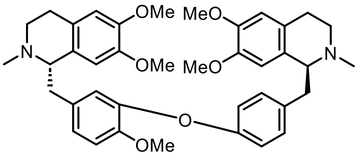

^{1}H NMR (500 MHz, $CDCl_3$) of **(*S*,*S*)-11**

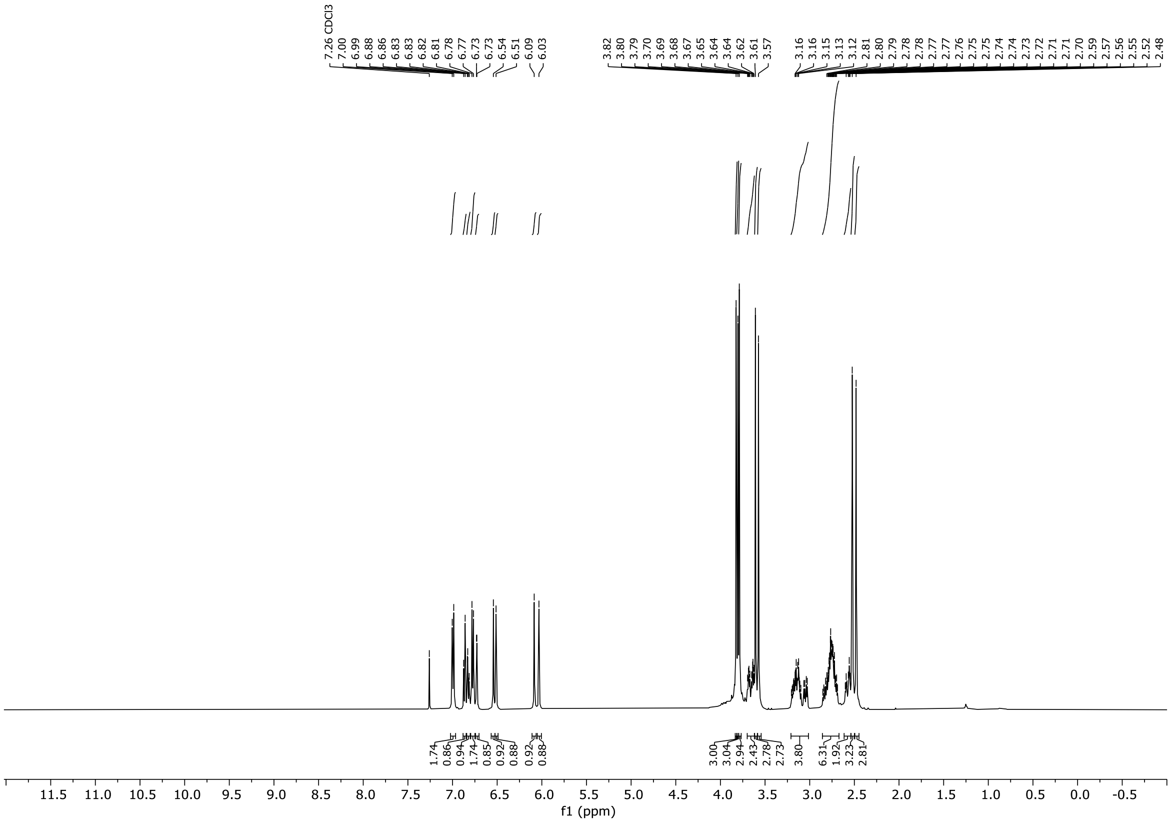

^{13}C NMR (126 MHz, $CDCl_3$) of **(*S*,*S*)-11**

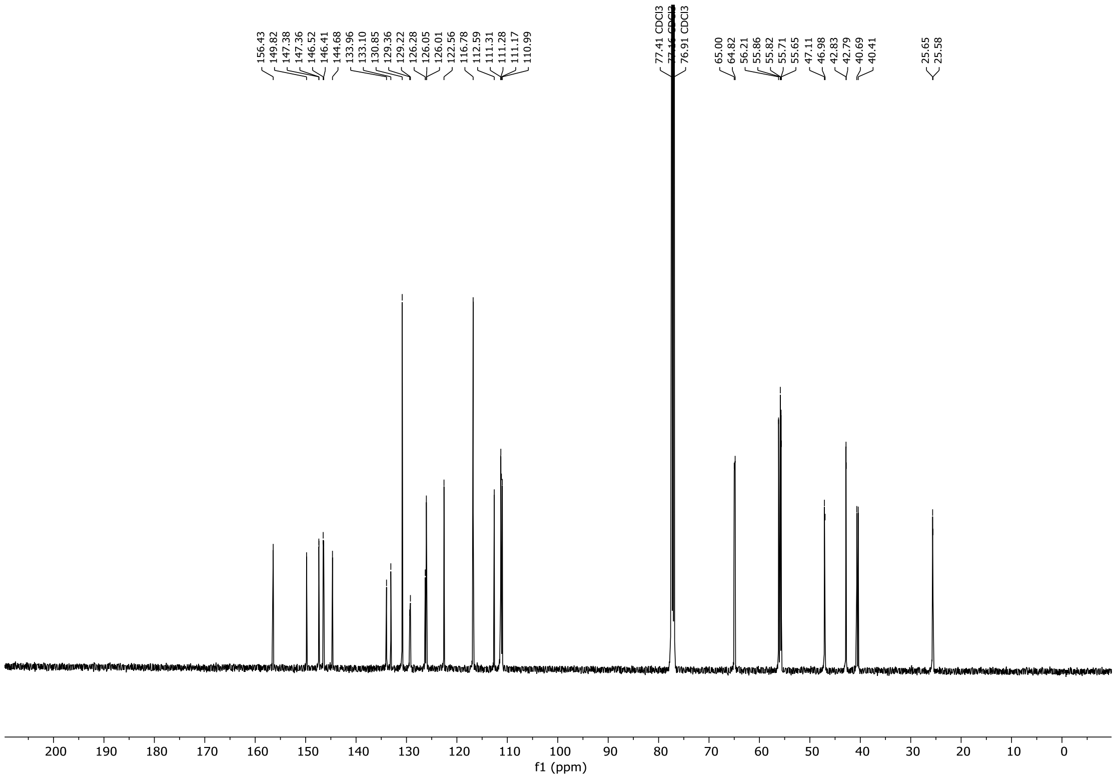

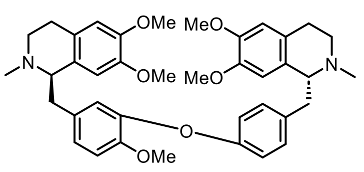

^{1}H NMR (500 MHz, $CDCl_3$) of ***(R,R)*-11**

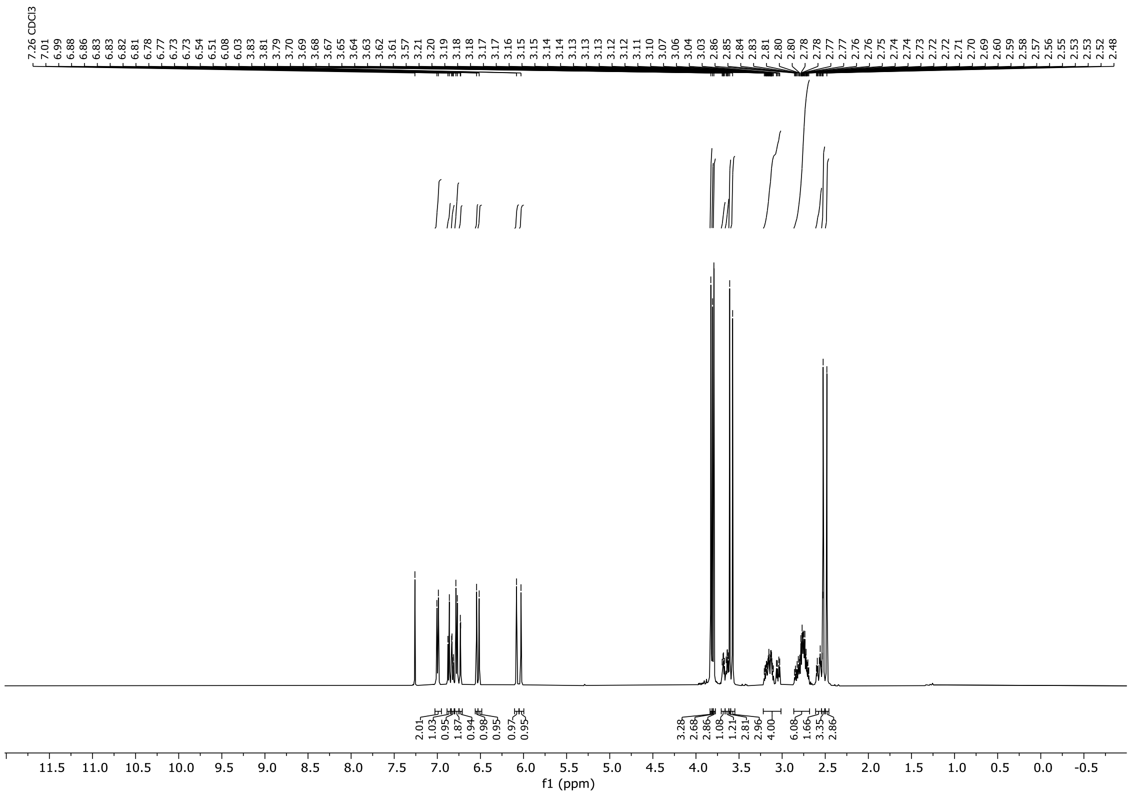

^{13}C NMR (126 MHz, $CDCl_3$) of ***(R,R)*-11**

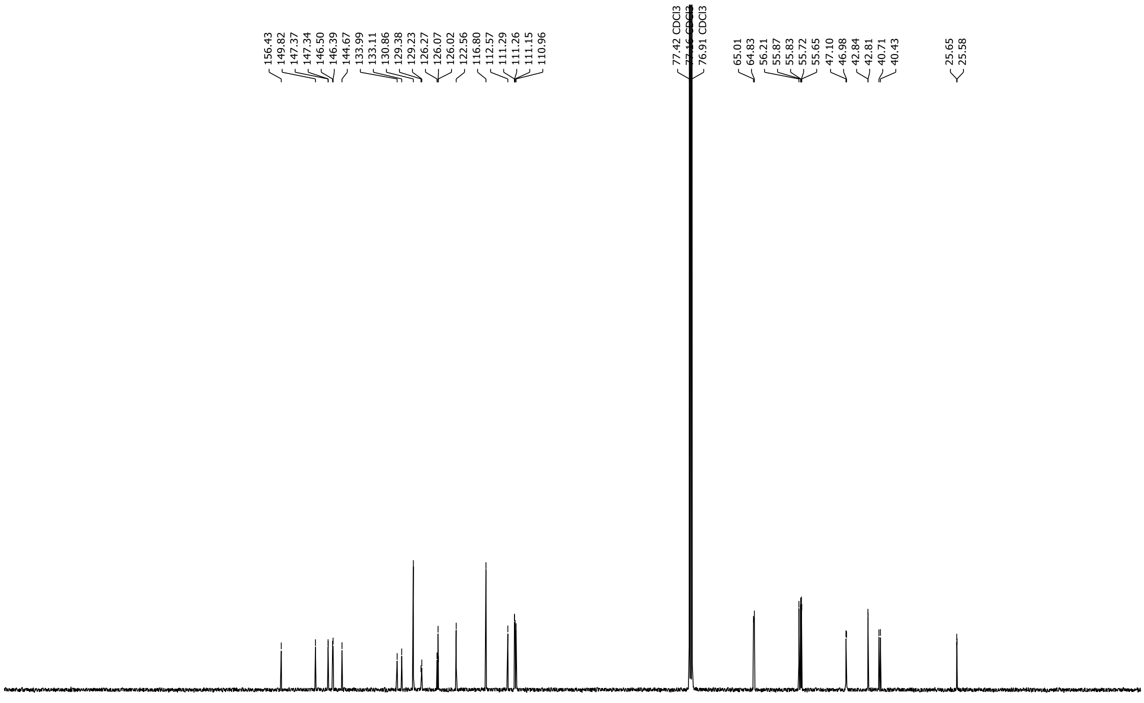

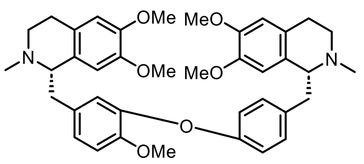

^{1}H NMR (500 MHz, $CDCl_3$) of ***(S,R)*-11**

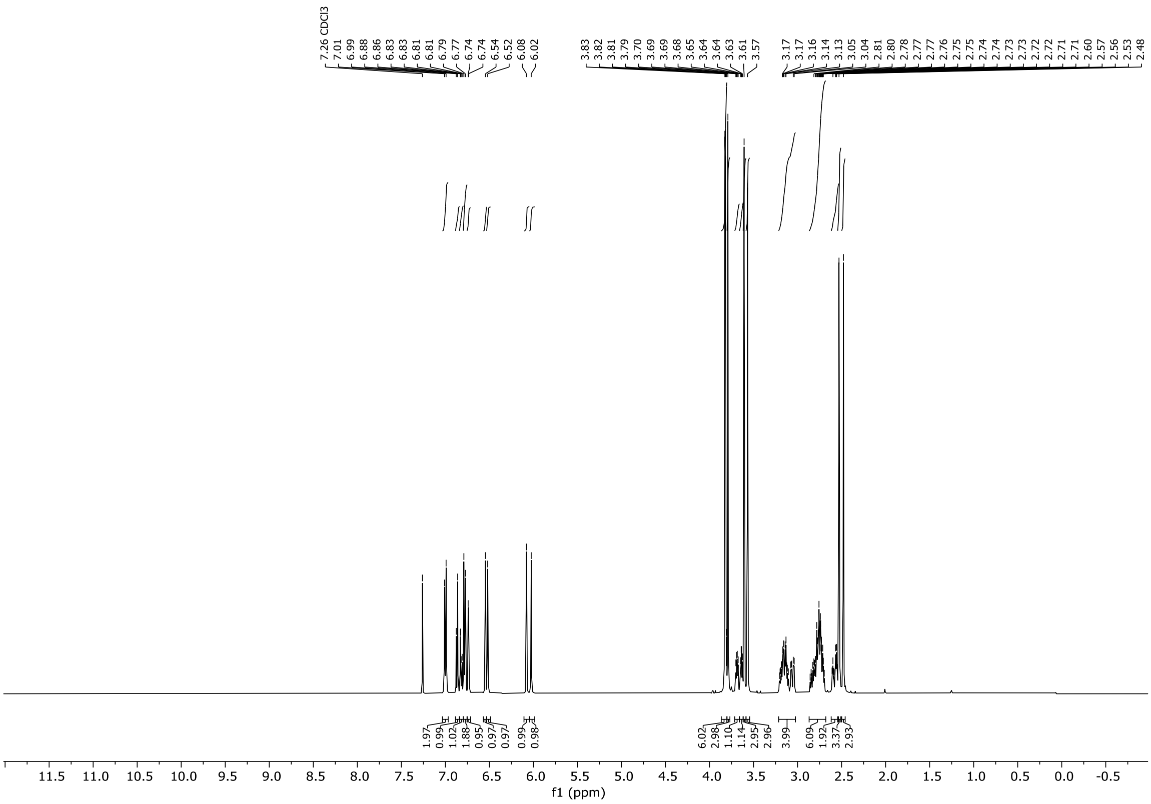

^{13}C NMR (126 MHz, $CDCl_3$) of ***(S,R)*-11**

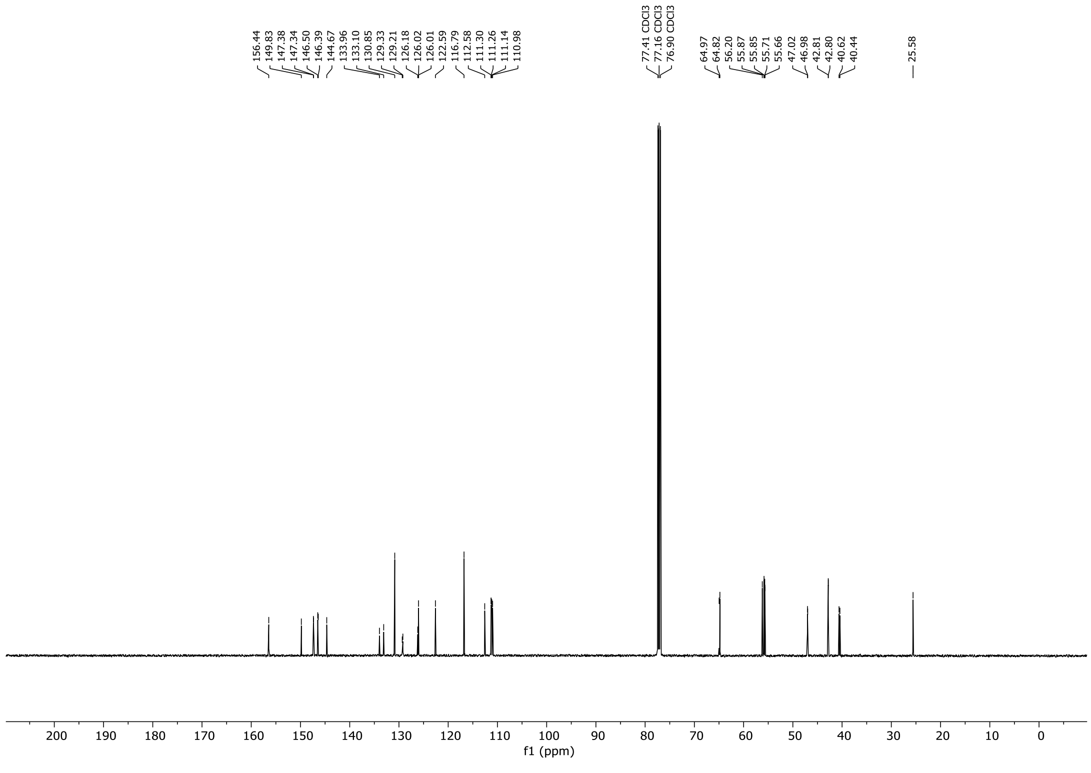

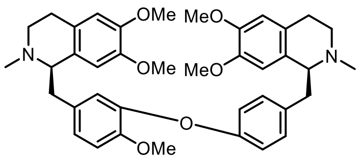

^{1}H NMR (500 MHz, $CDCl_3$) of ***(R,S)*-11**

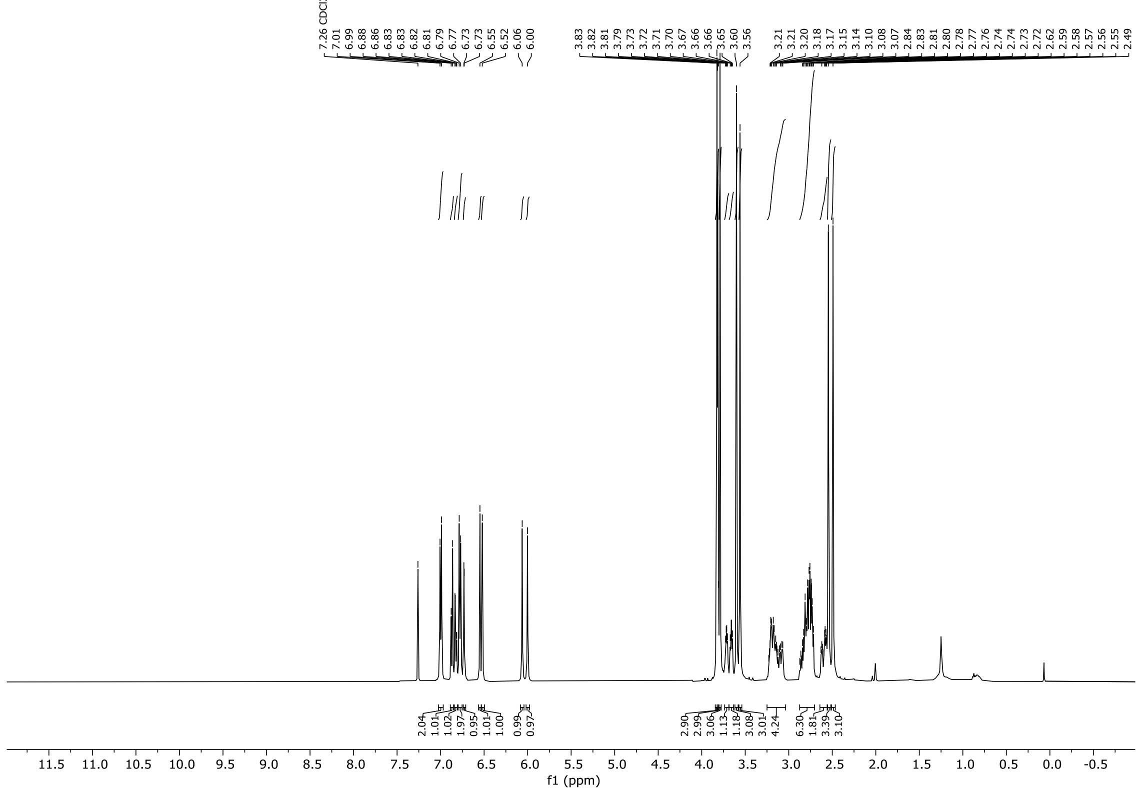

^{13}C NMR (126 MHz, $CDCl_3$) of ***(R,S)*-11**

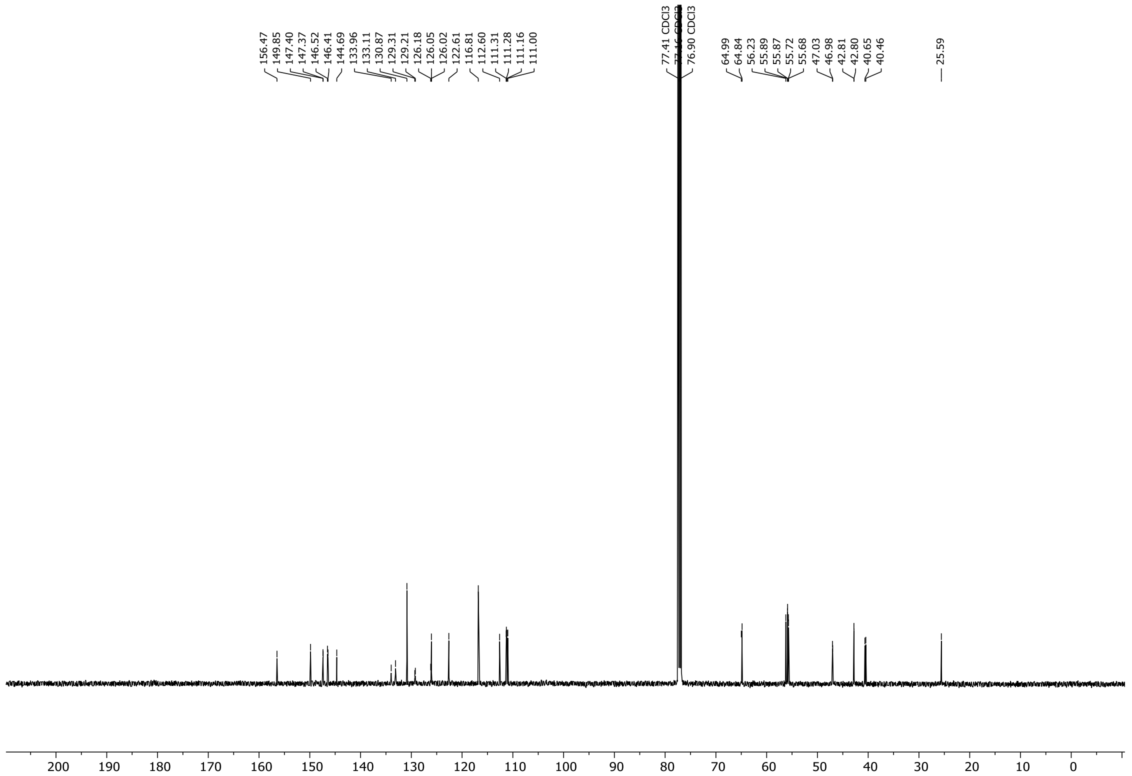

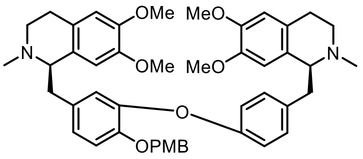

^{1}**H NMR** (400 MHz, $CDCl_3$) of ***(S,R)*-12**

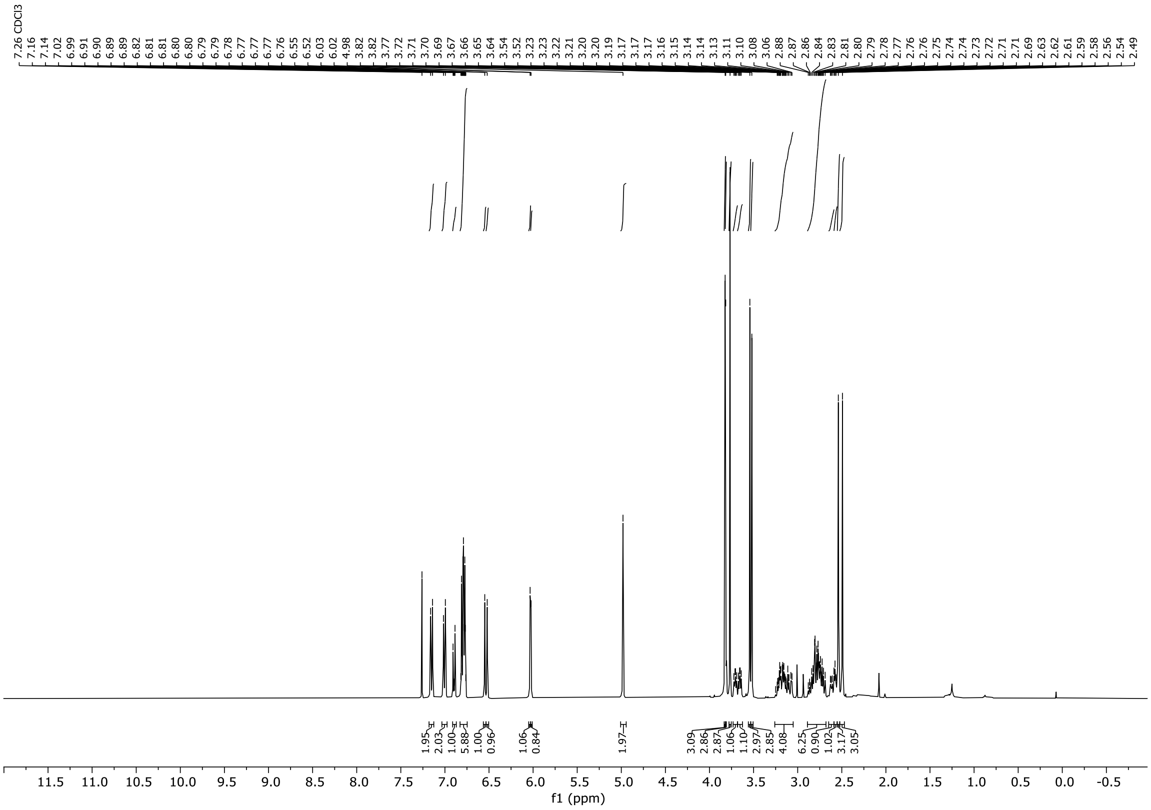

^{13}**C NMR** (101 MHz, $CDCl_3$) of ***(S,R)*-12**

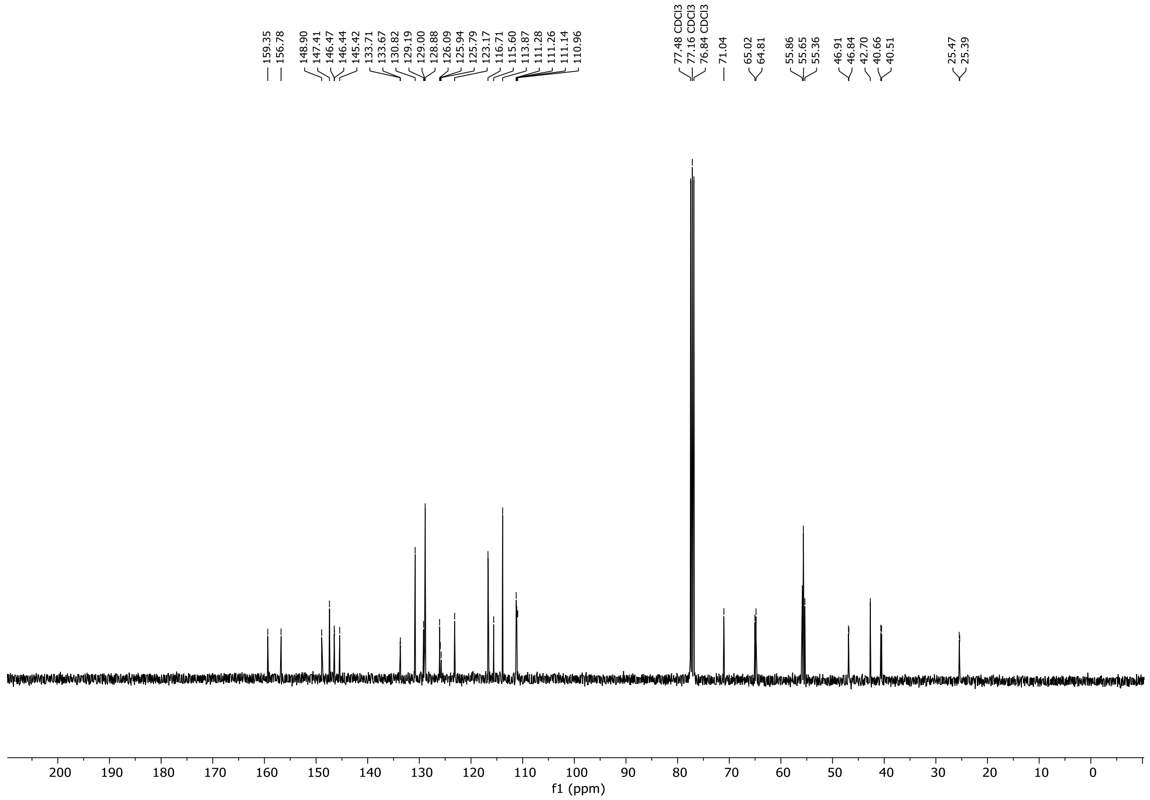

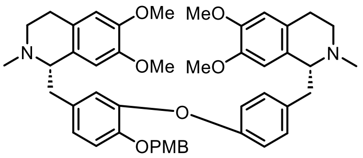

^{1}H NMR (500 MHz, $CDCl_3$) of ***(R,S)*-12**

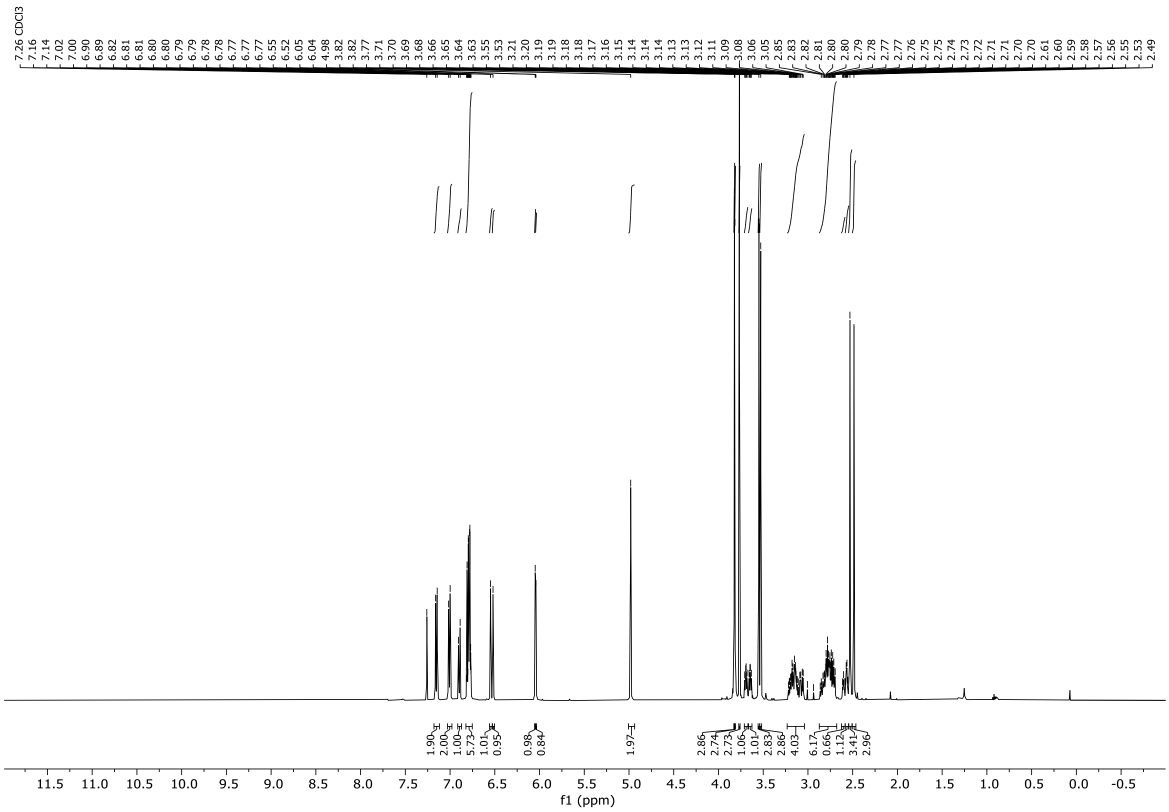

^{13}C NMR (126 MHz, $CDCl_3$) of ***(R,S)*-12**

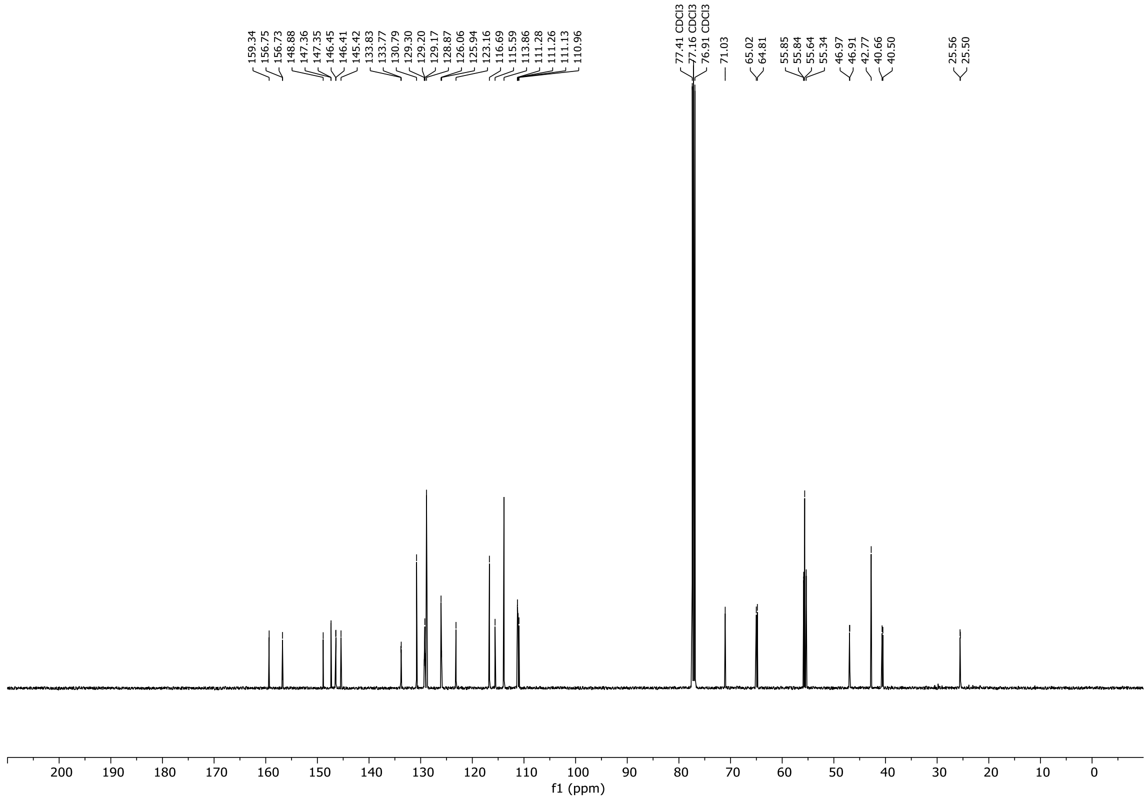

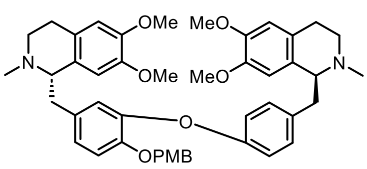

^{1}H NMR (500 MHz, CDCl$_3$) of ***(S,S)*-12**

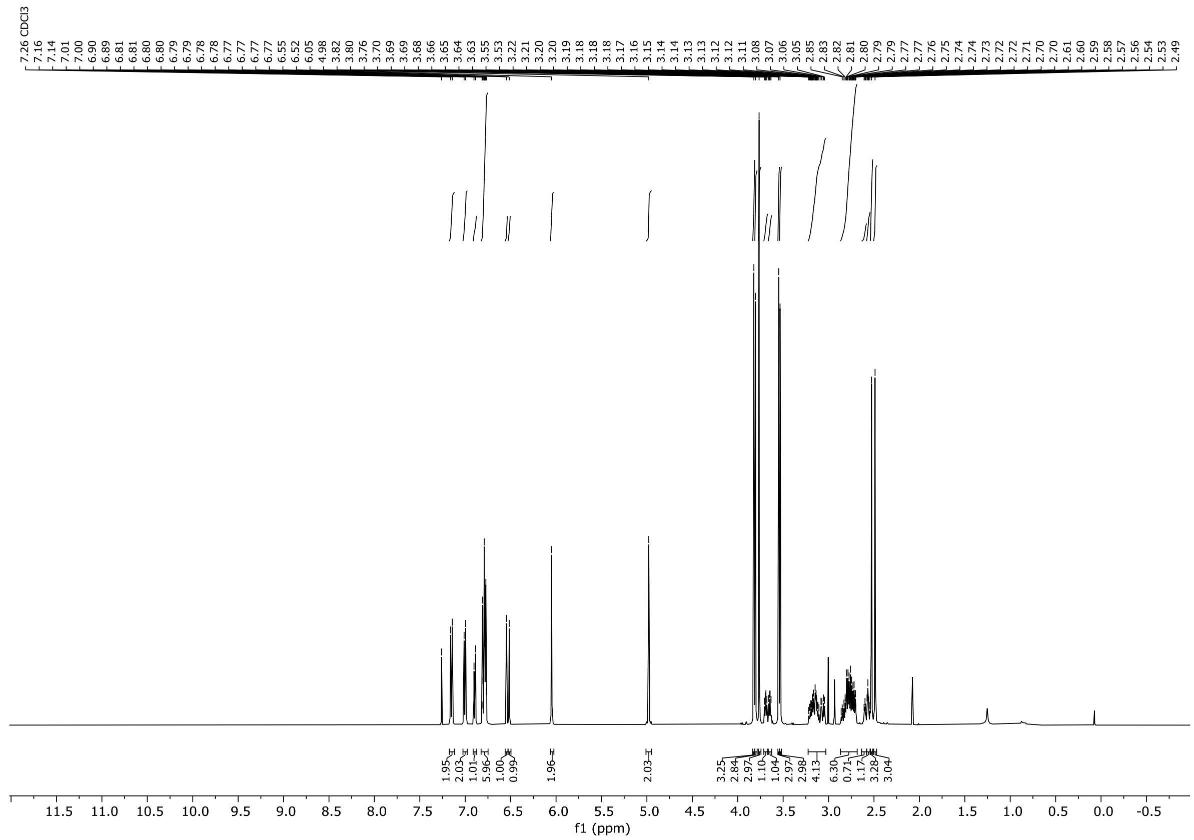

^{13}C NMR (126 MHz, CDCl$_3$) of ***(S,S)*-12**

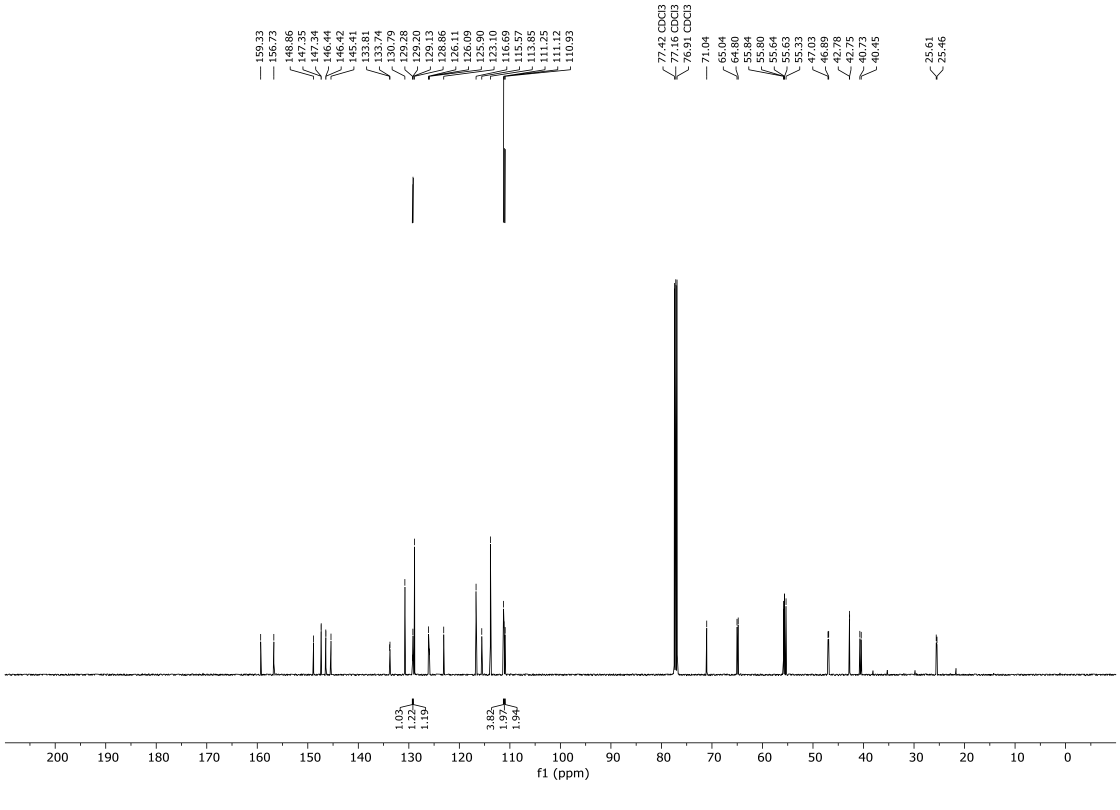

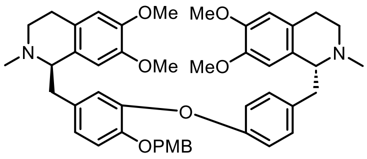

^{1}H NMR (500 MHz, CDCl$_3$) of ***(R,R)*-12**

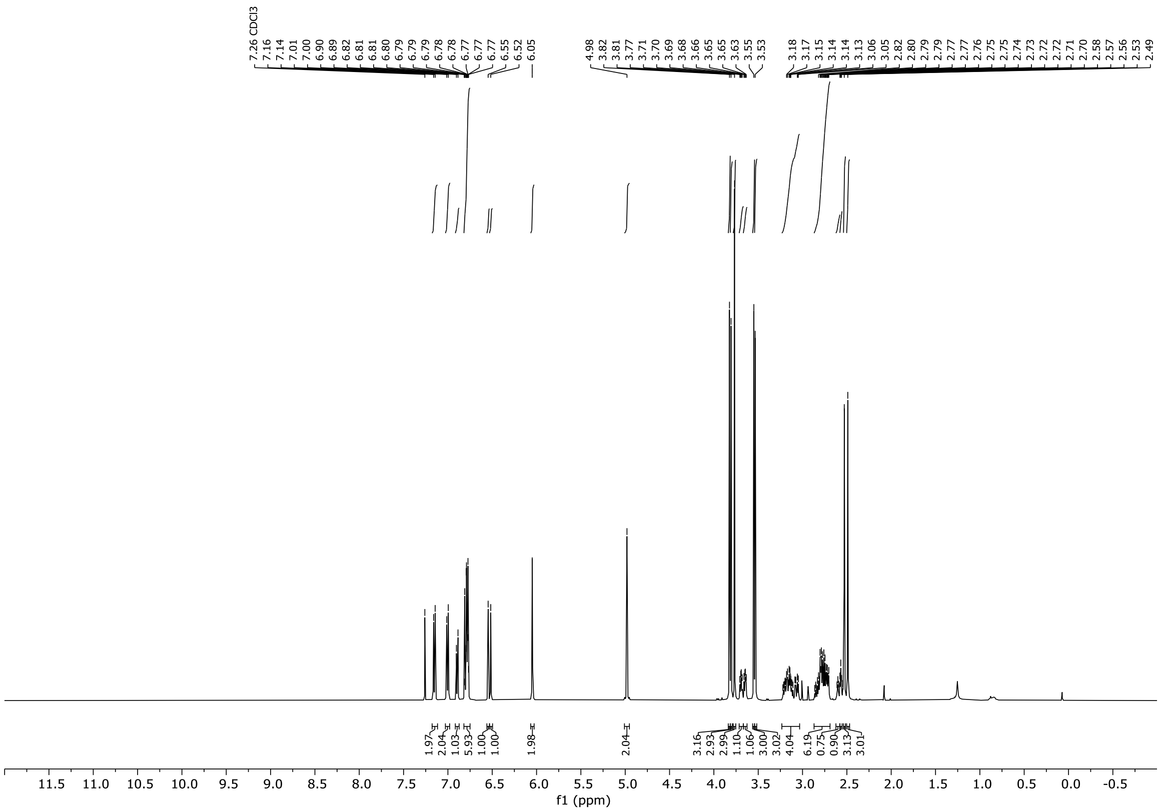

^{13}C NMR (126 MHz, CDCl$_3$) of ***(R,R)*-12**

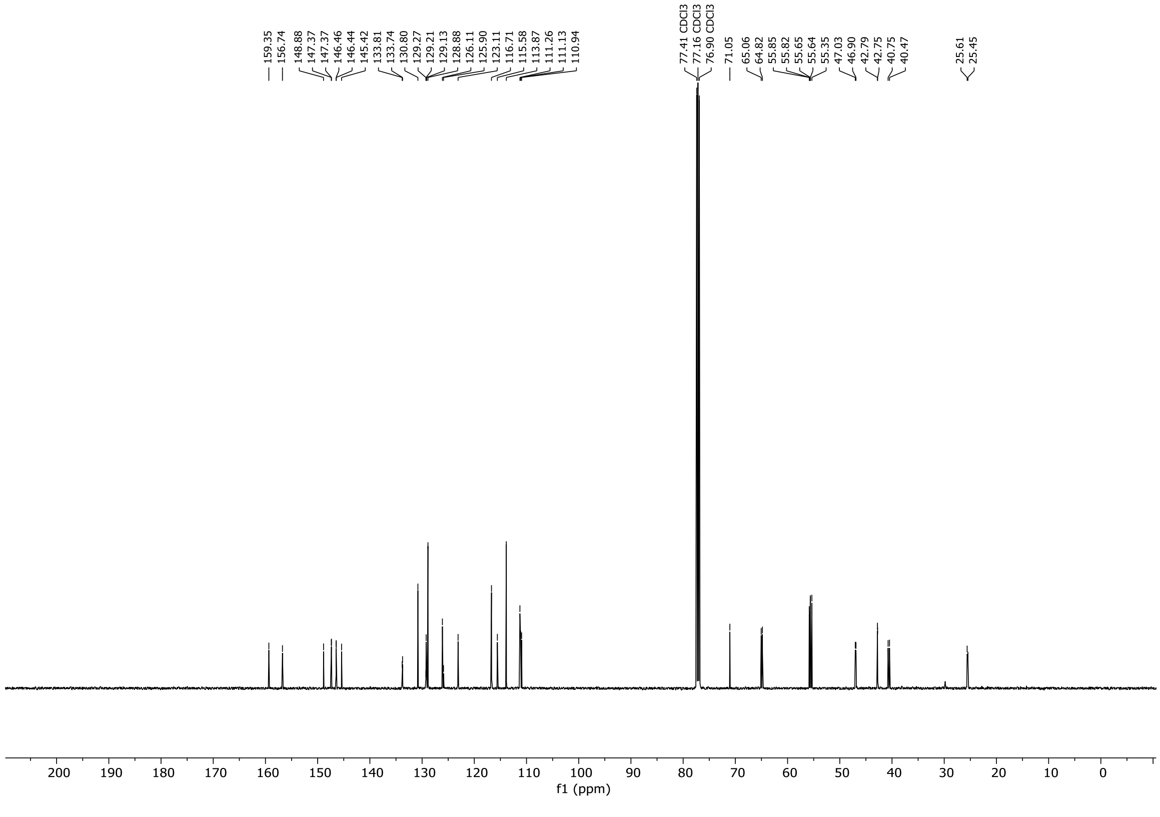

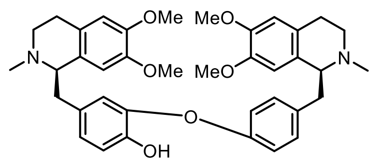

^{1}H NMR (600 MHz, $CDCl_3$) of ***(S,R)*-13**

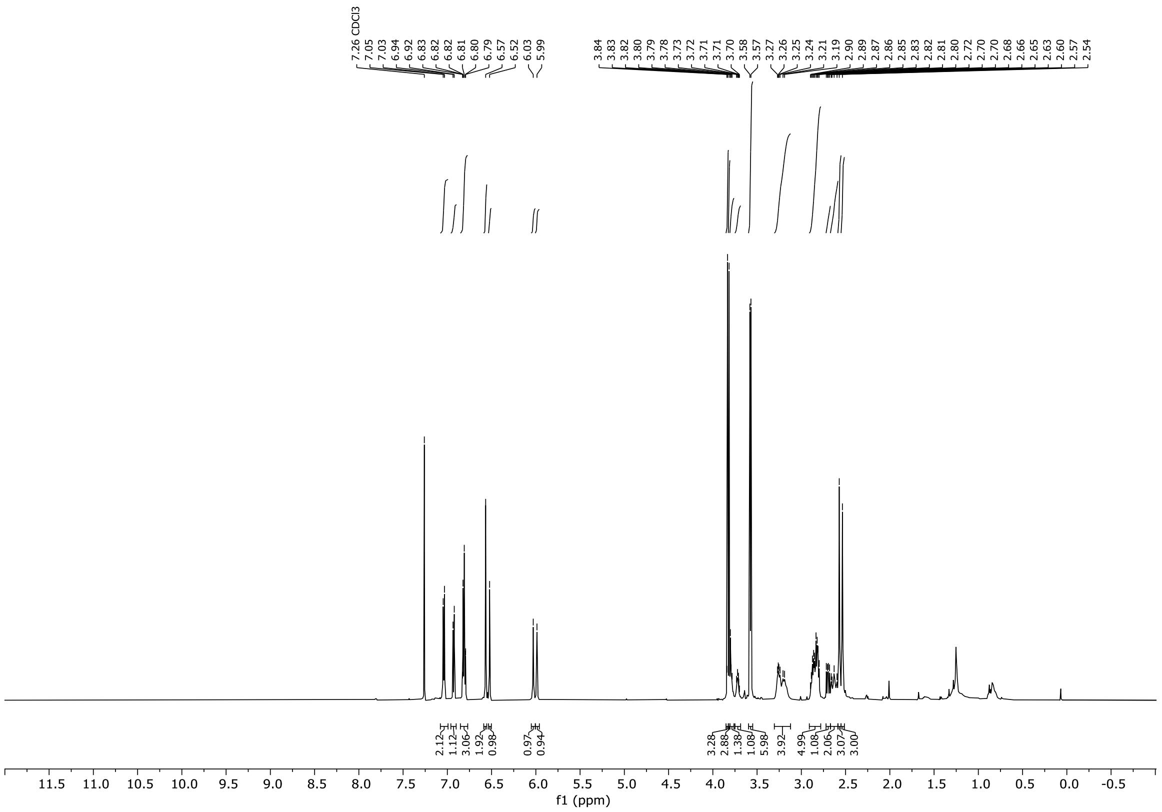

^{13}C NMR (151 MHz, $CDCl_3$) of ***(S,R)*-13**

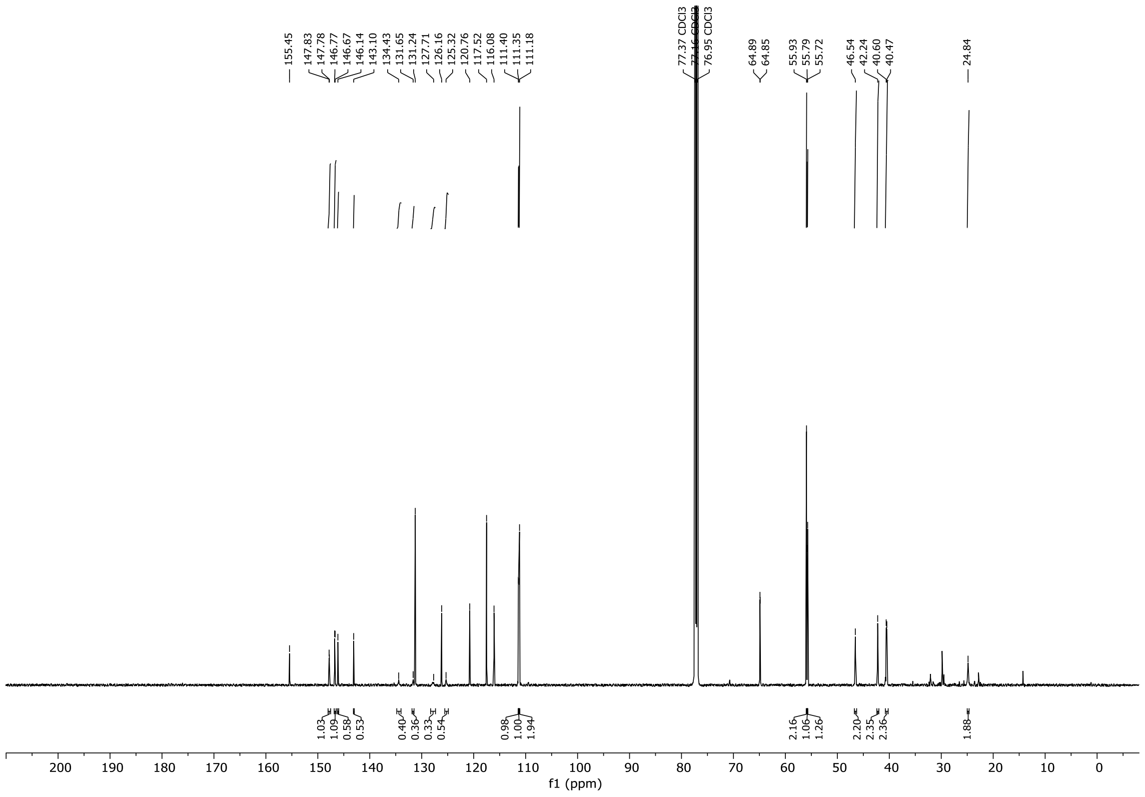

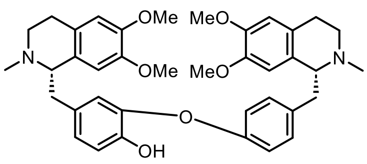

^{1}H NMR (600 MHz, $CDCl_3$) of ***(R,S)*-13**

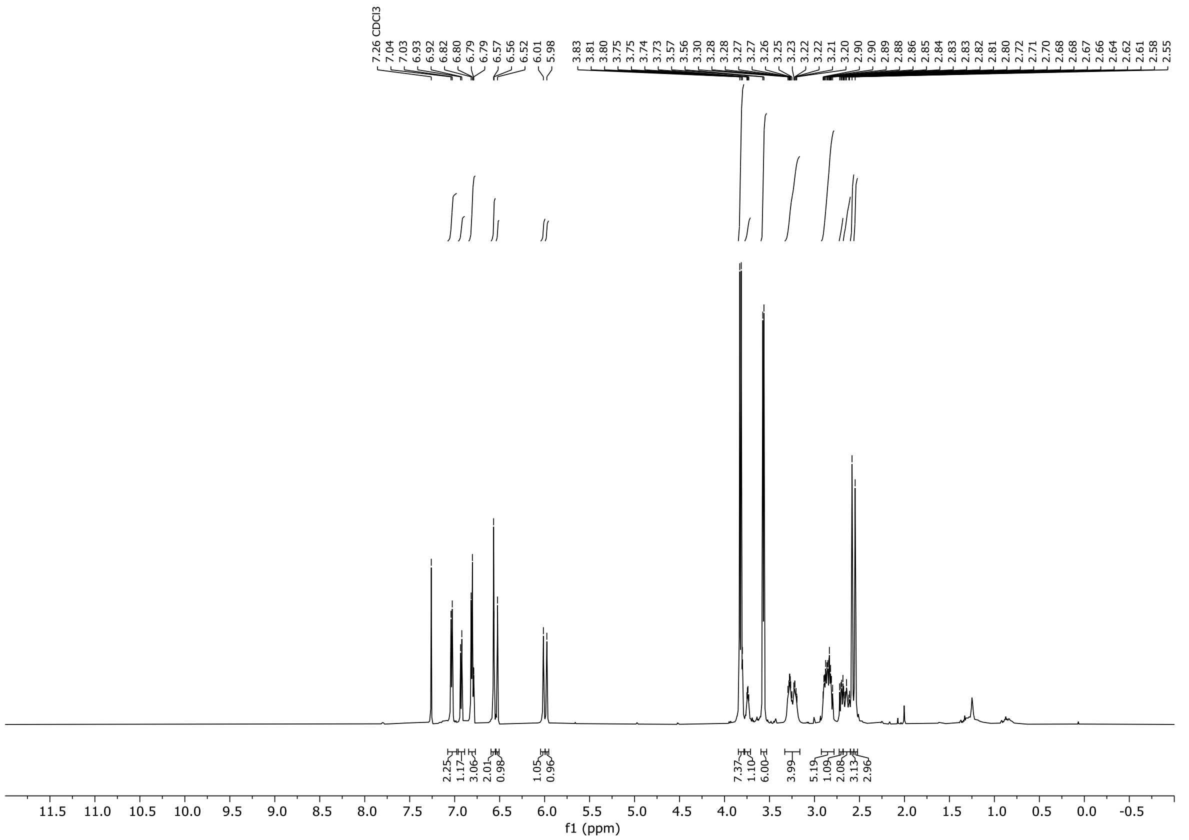

^{13}C NMR (151 MHz, $CDCl_3$) of ***(R,S)*-13**

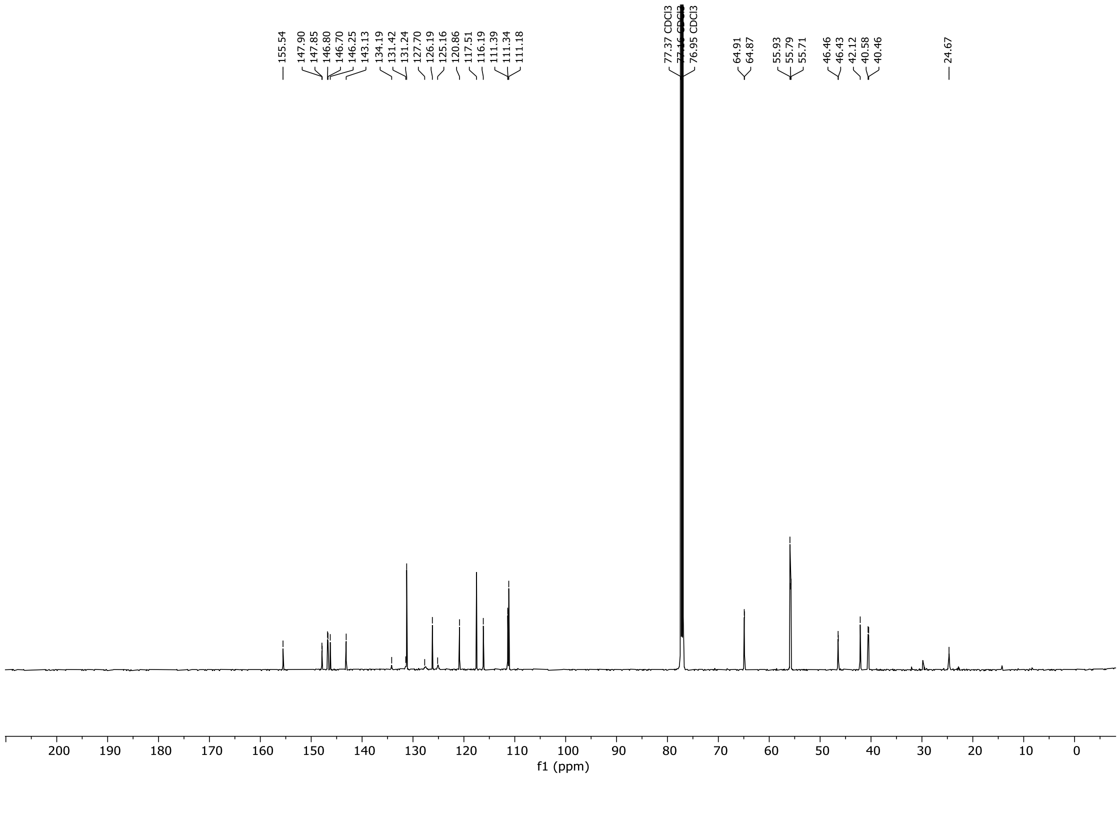

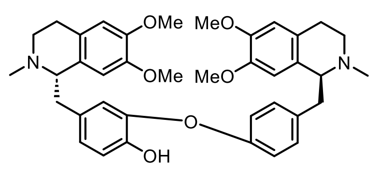

^{1}H NMR (500 MHz, CDCl$_3$) of ***(S,S)*-13**

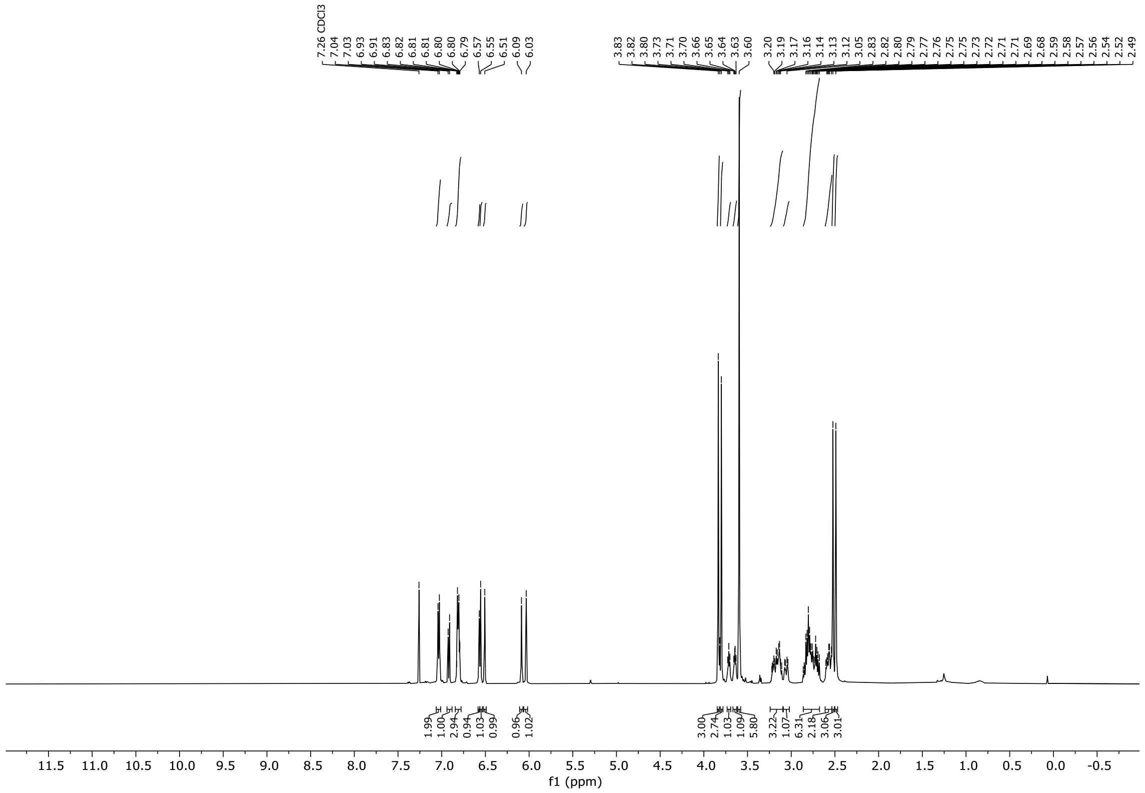

^{13}C NMR (126 MHz, CDCl$_3$) of ***(S,S)*-13**

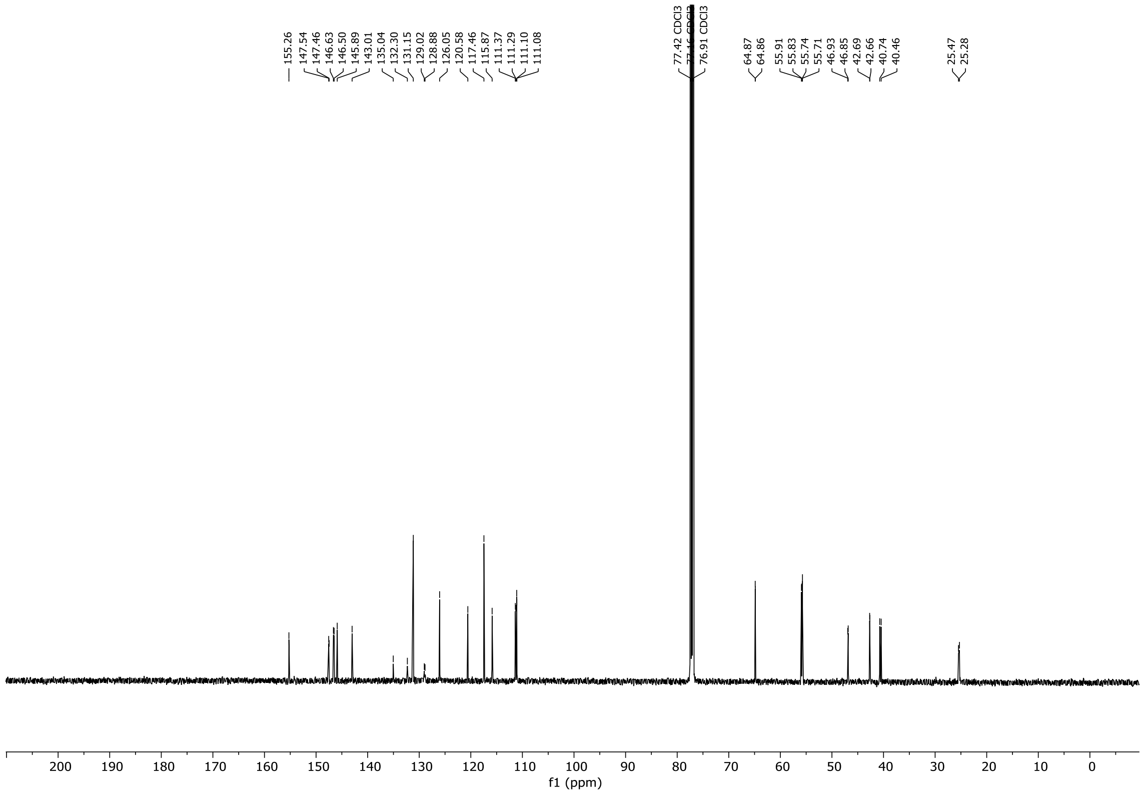

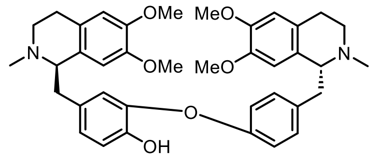

^{1}H NMR (500 MHz, $CDCl_3$) of **(*R*,*R*)-13**

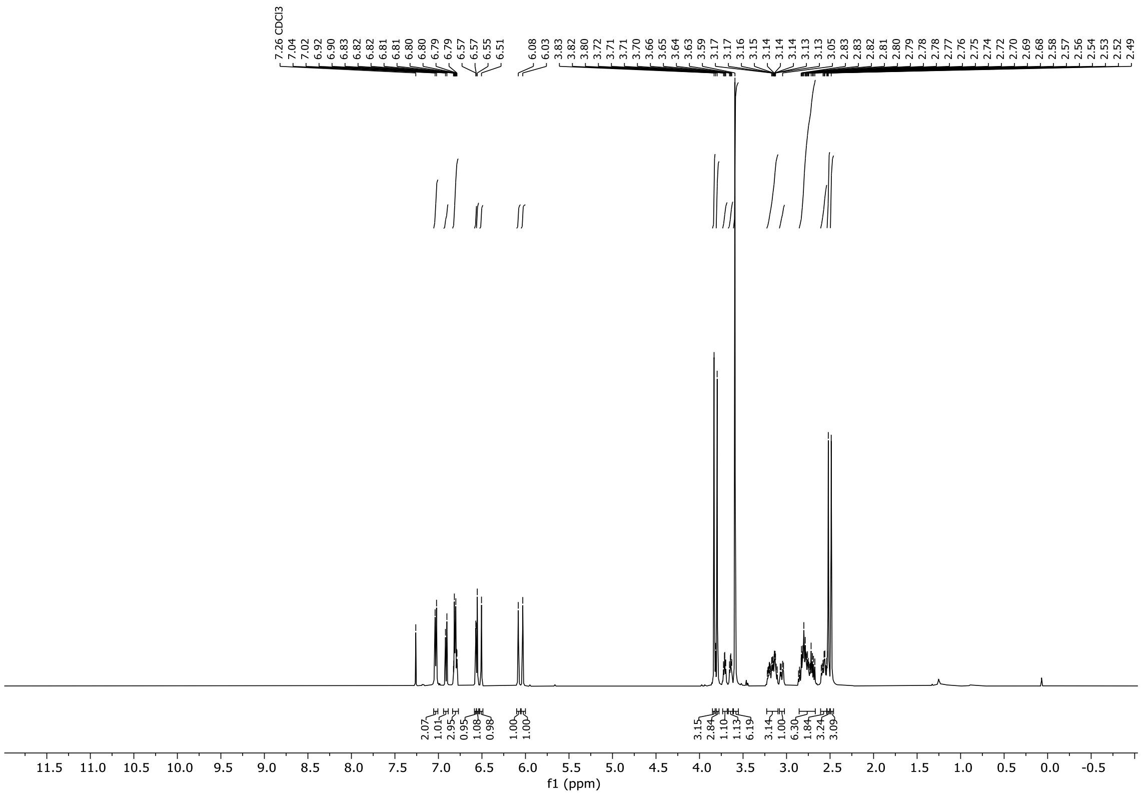

^{13}C NMR (126 MHz, $CDCl_3$) of **(*R*,*R*)-13**

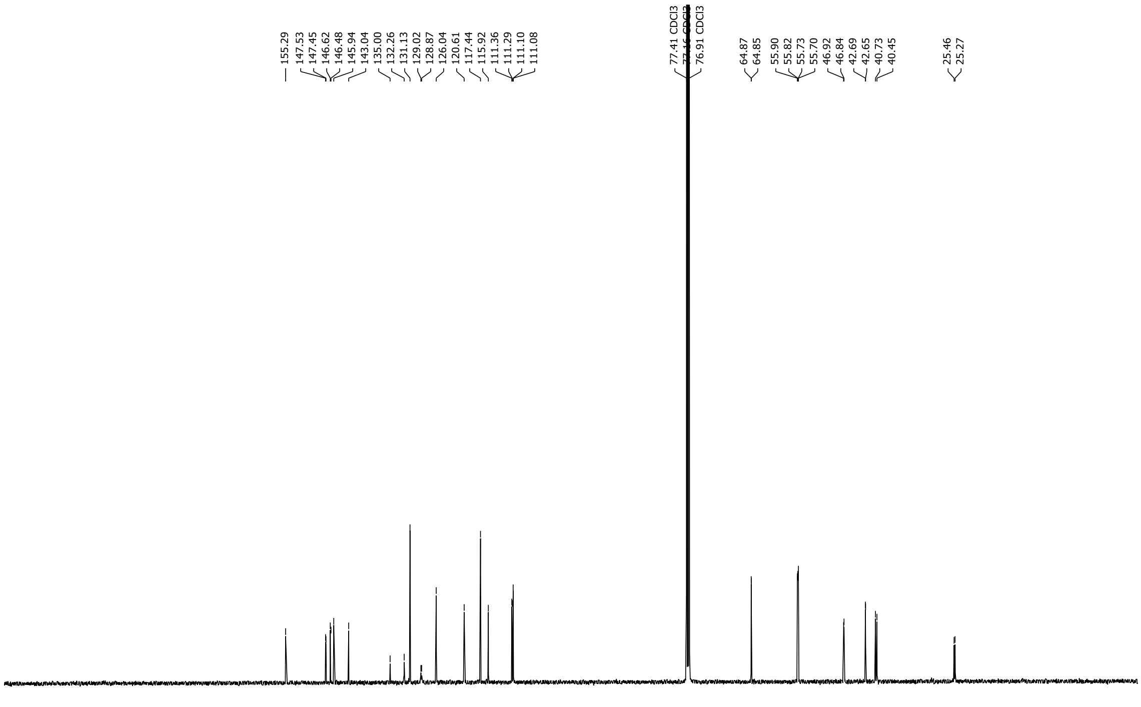

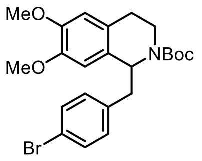

^{1}H NMR (400 MHz, $CDCl_3$, 2 rotamers) of **rac-S1**

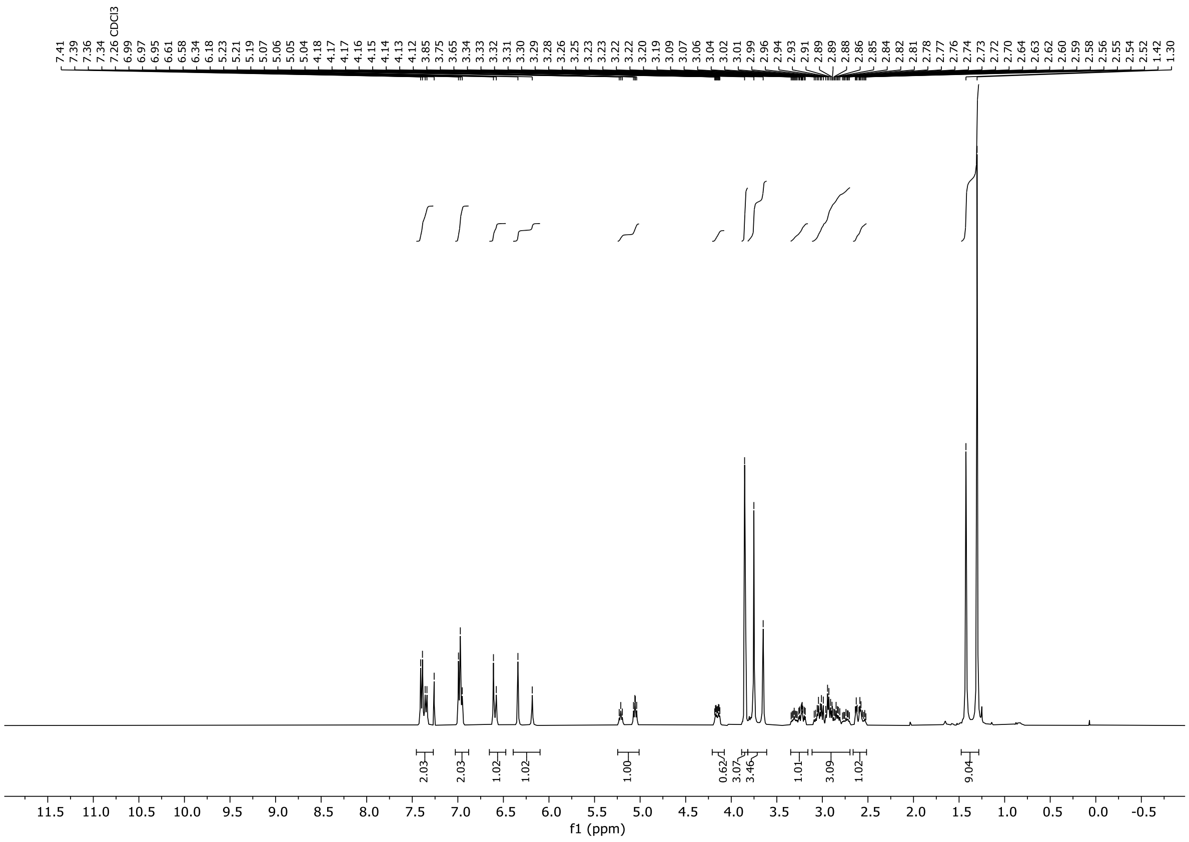

^{13}C NMR (126 MHz, $CDCl_3$, rotamers) of **rac-S1**

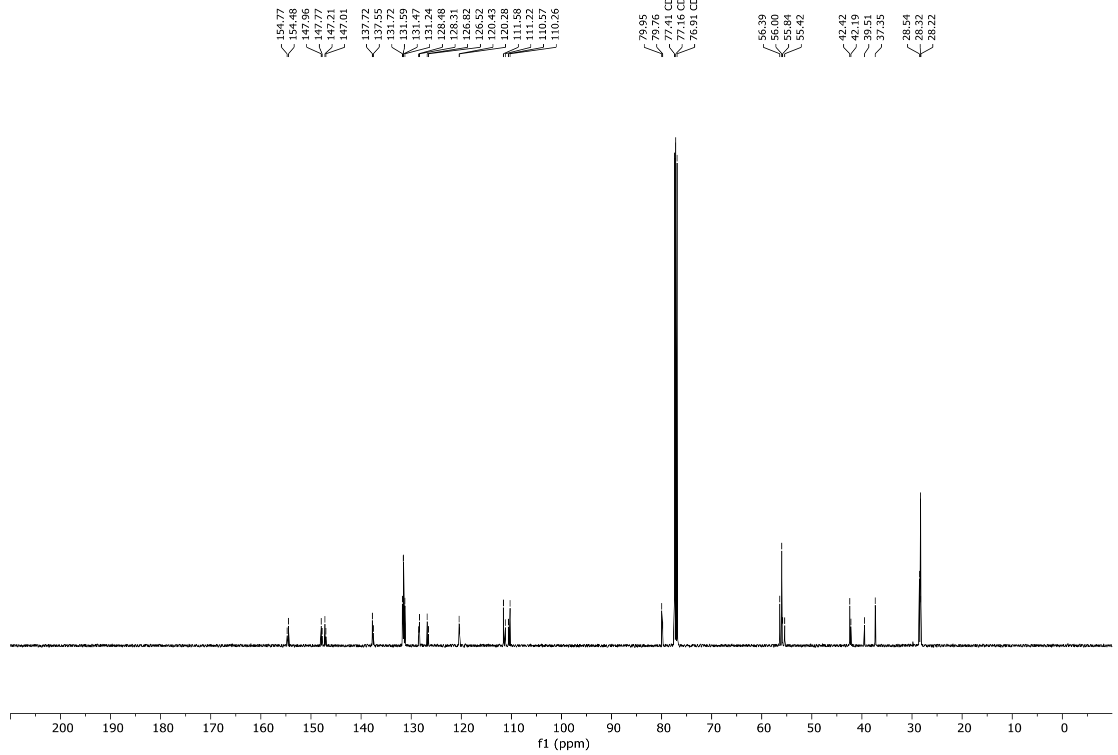

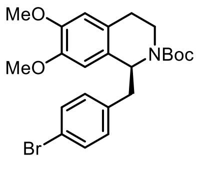

^{1}H NMR (400 MHz, $CDCl_3$, 2 rotamers) of ***(S)*-S1**

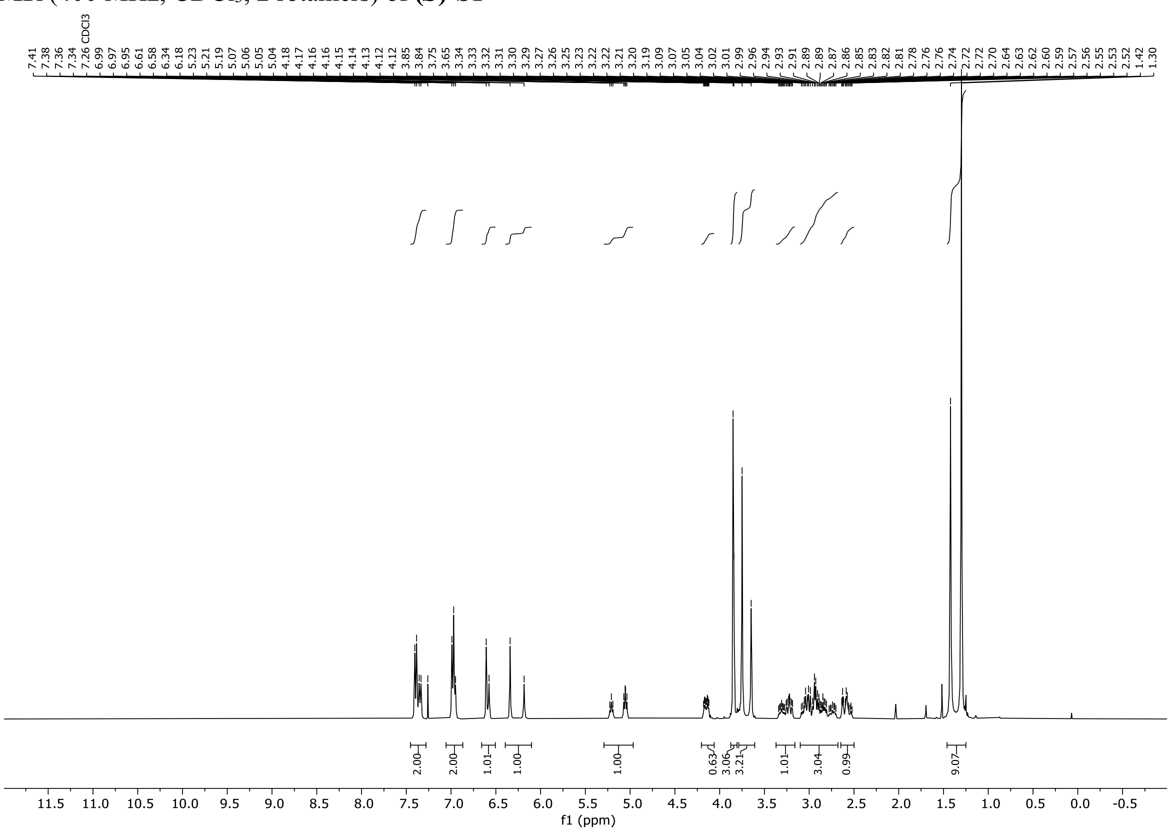

^{13}C NMR (101 MHz, $CDCl_3$, rotamers) of ***(S)*-S1**

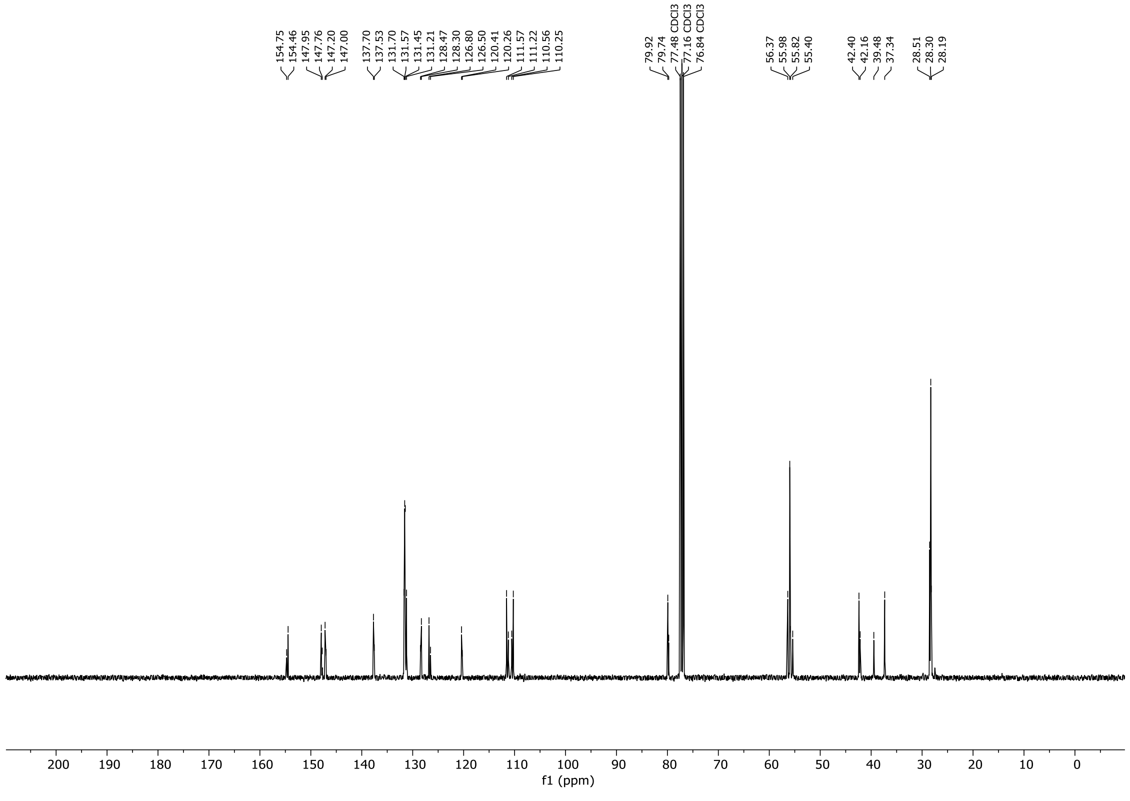

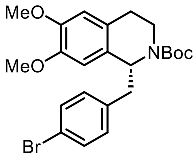

^{1}H NMR (500 MHz, $CDCl_3$, 2 rotamers) of ***(R)*-S1**

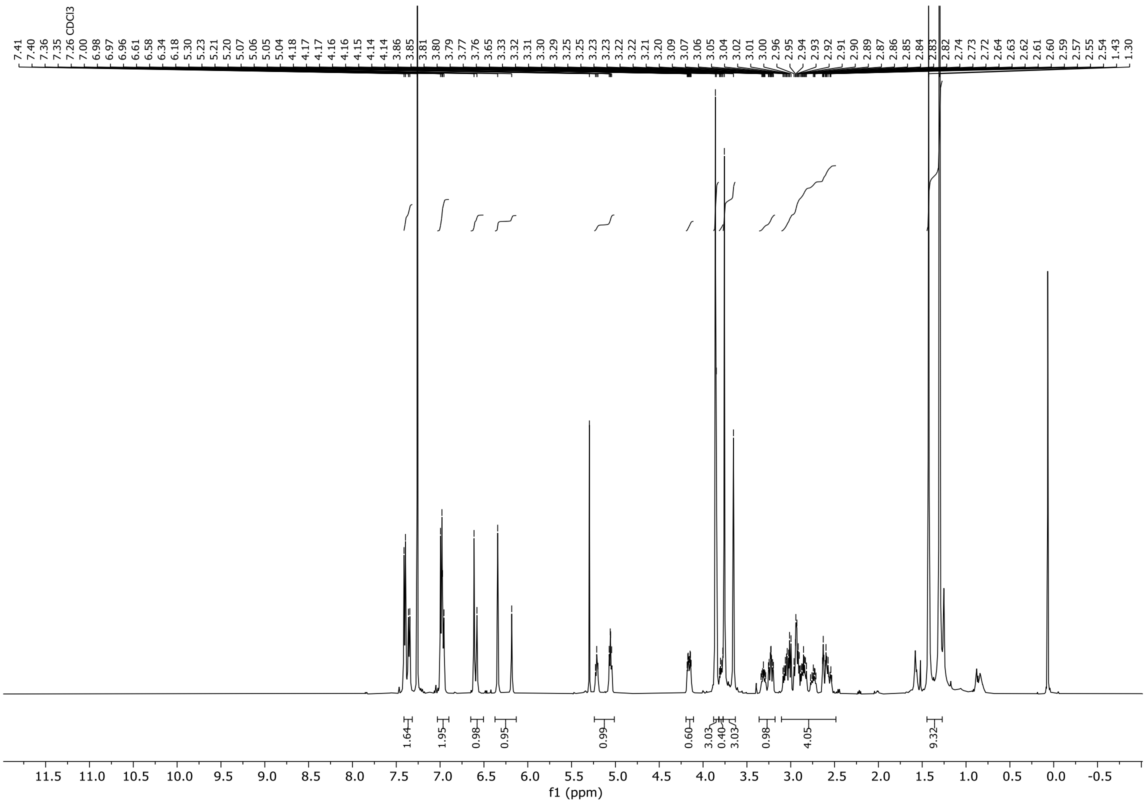

^{13}C NMR (126 MHz, $CDCl_3$, rotamers) ***(R)*-S1**

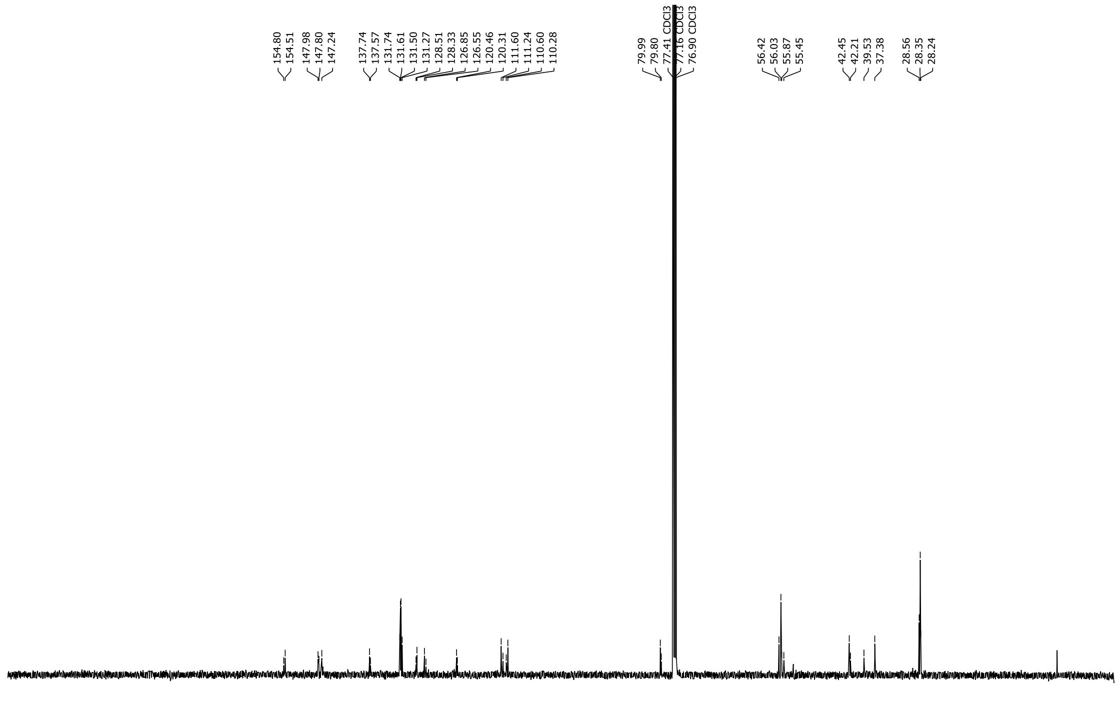

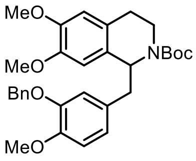

^{1}H NMR (400 MHz, $CDCl_3$, 2 rotamers) of **rac-S2**

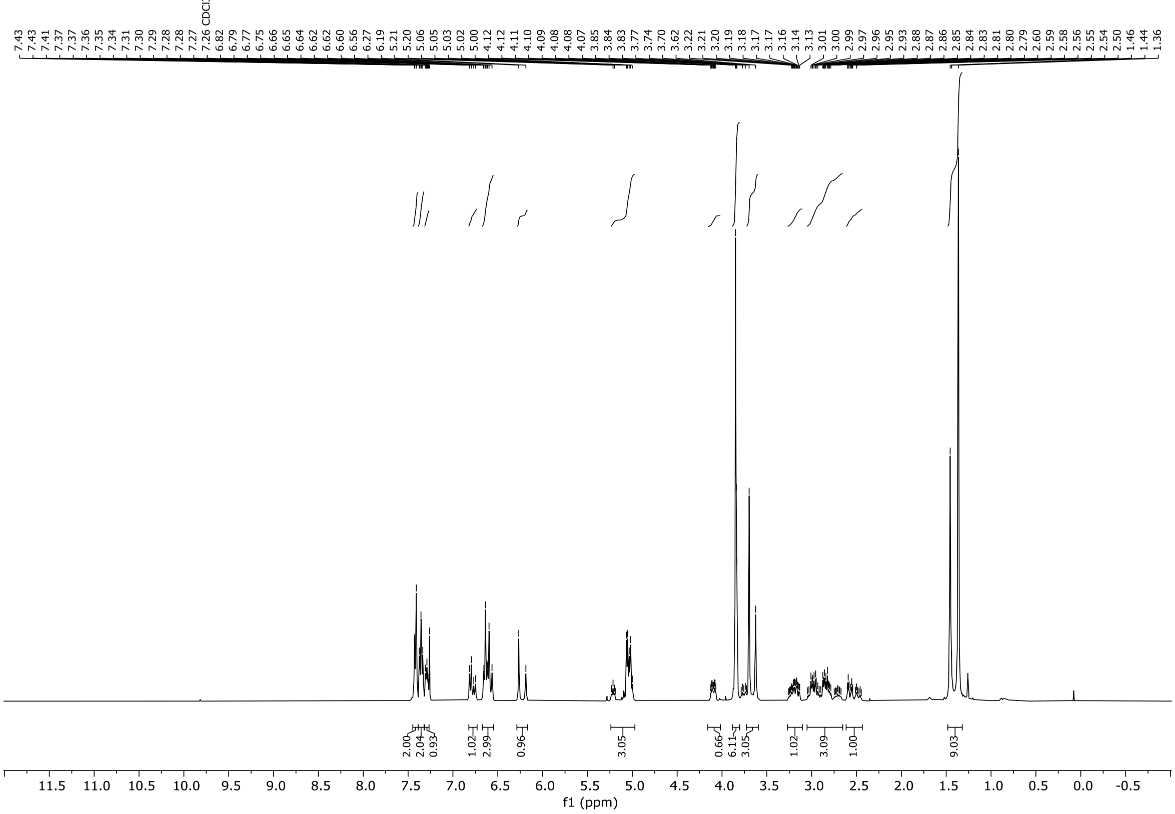

^{13}C NMR (101 MHz, $CDCl_3$, rotamers) of **rac-S2**

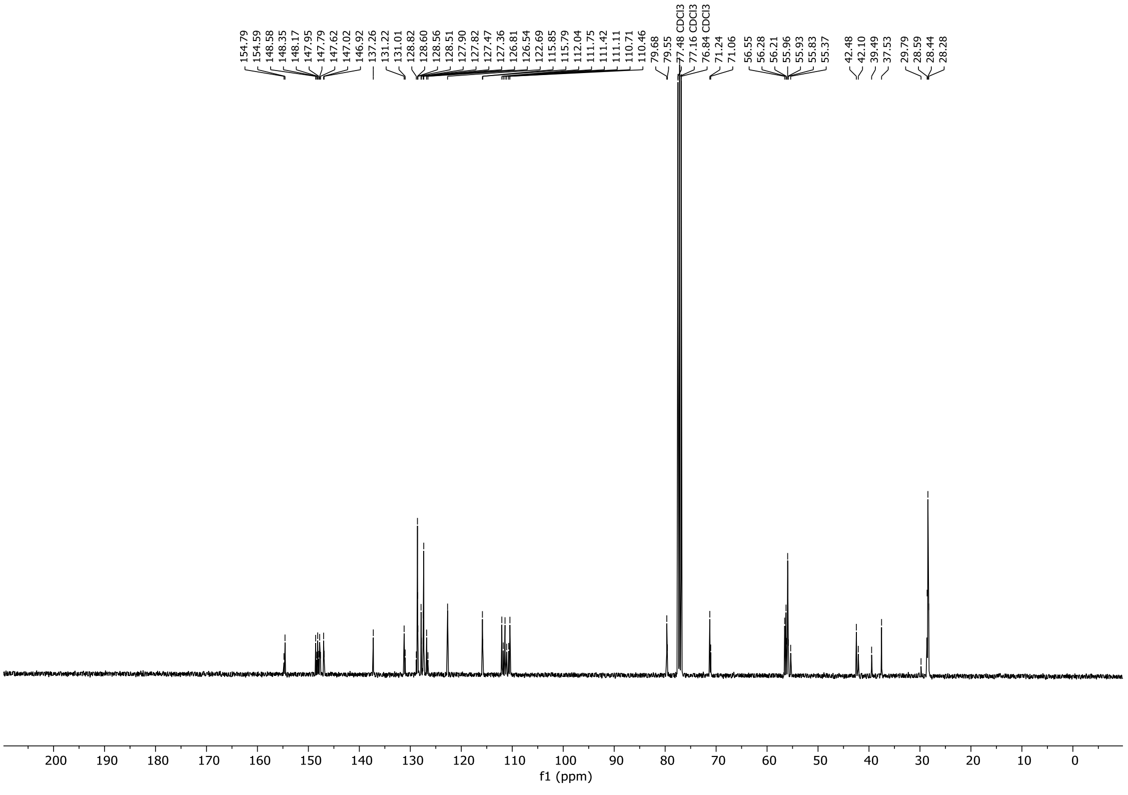

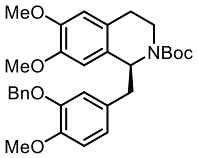

^{1}H NMR (600 MHz, $CDCl_3$, 2 rotamers) of **(*S*)-S2**

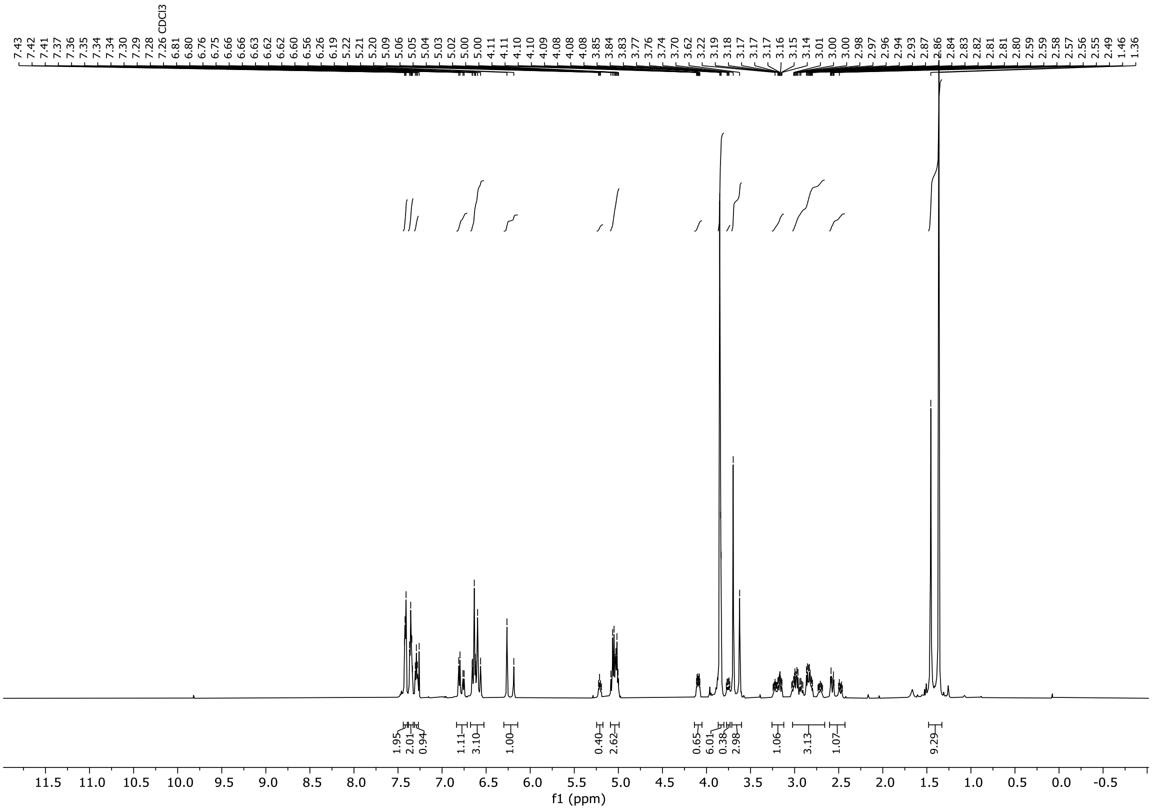

^{13}C NMR (151 MHz, $CDCl_3$, rotamers) of **(*S*)-S2**

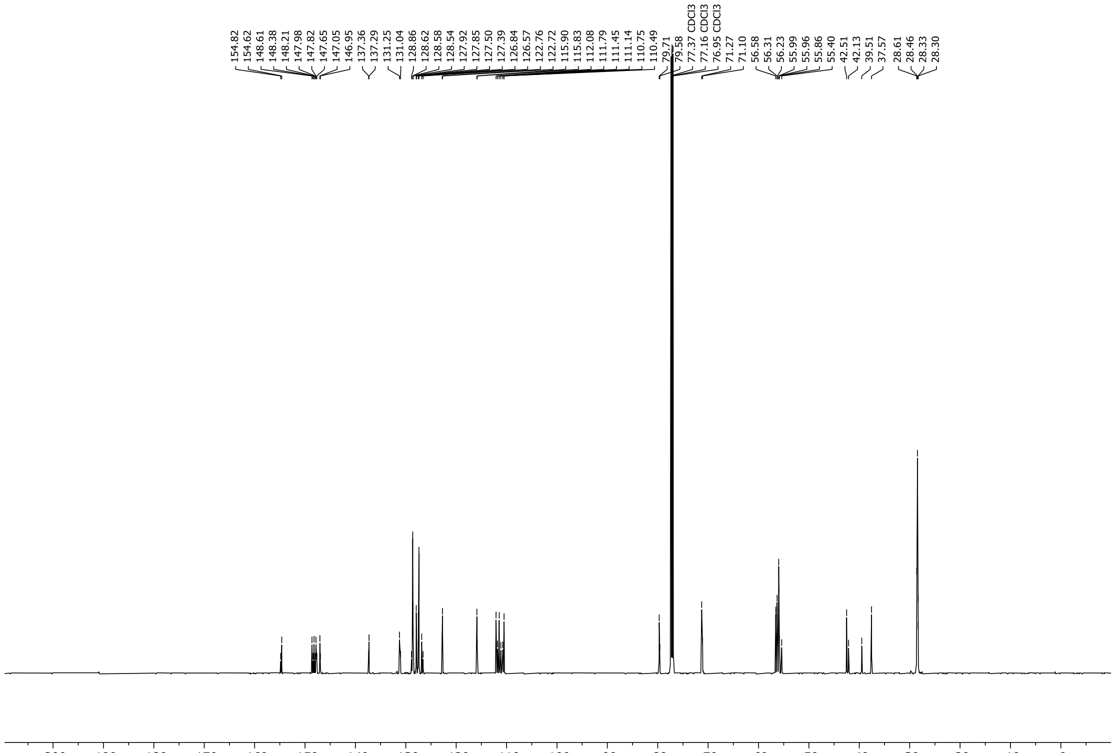

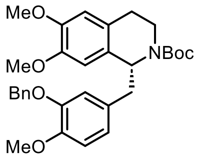

^{1}H NMR (600 MHz, $CDCl_3$, 2 rotamers) of ***(R)*-S2**

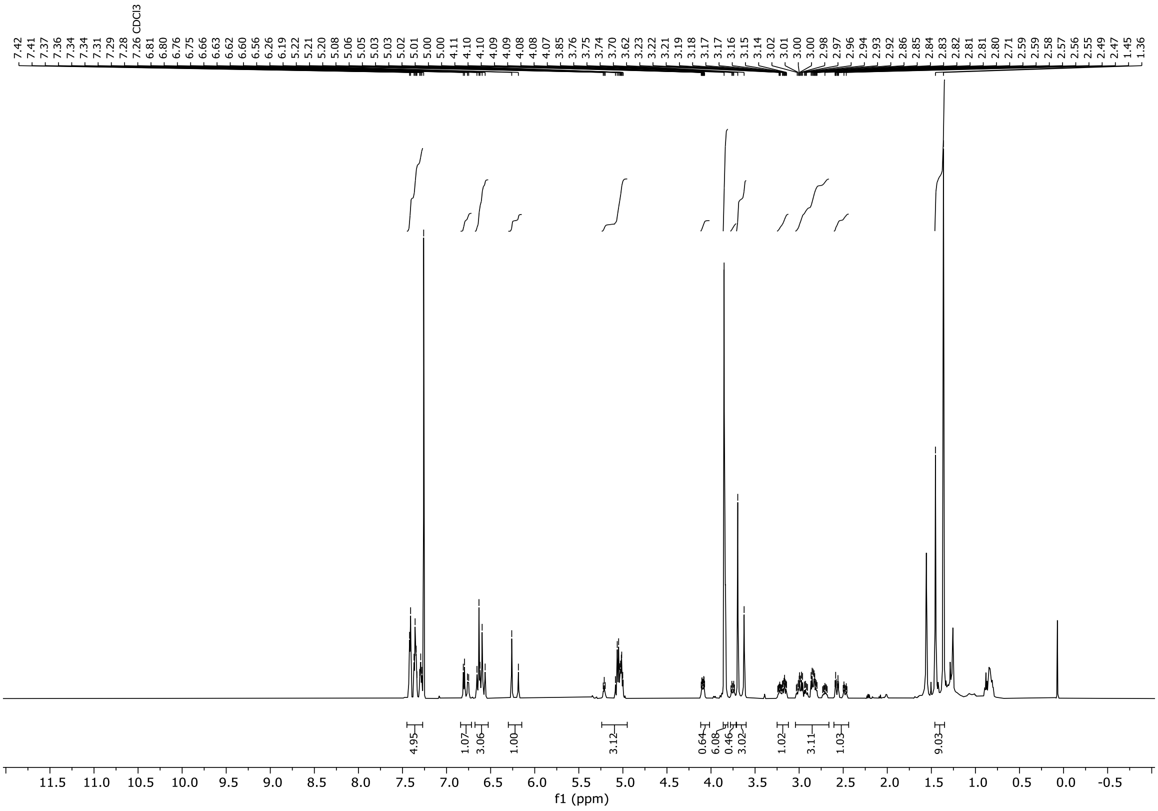

^{13}C NMR (151 MHz, $CDCl_3$, rotamers) of ***(R)*-S2**

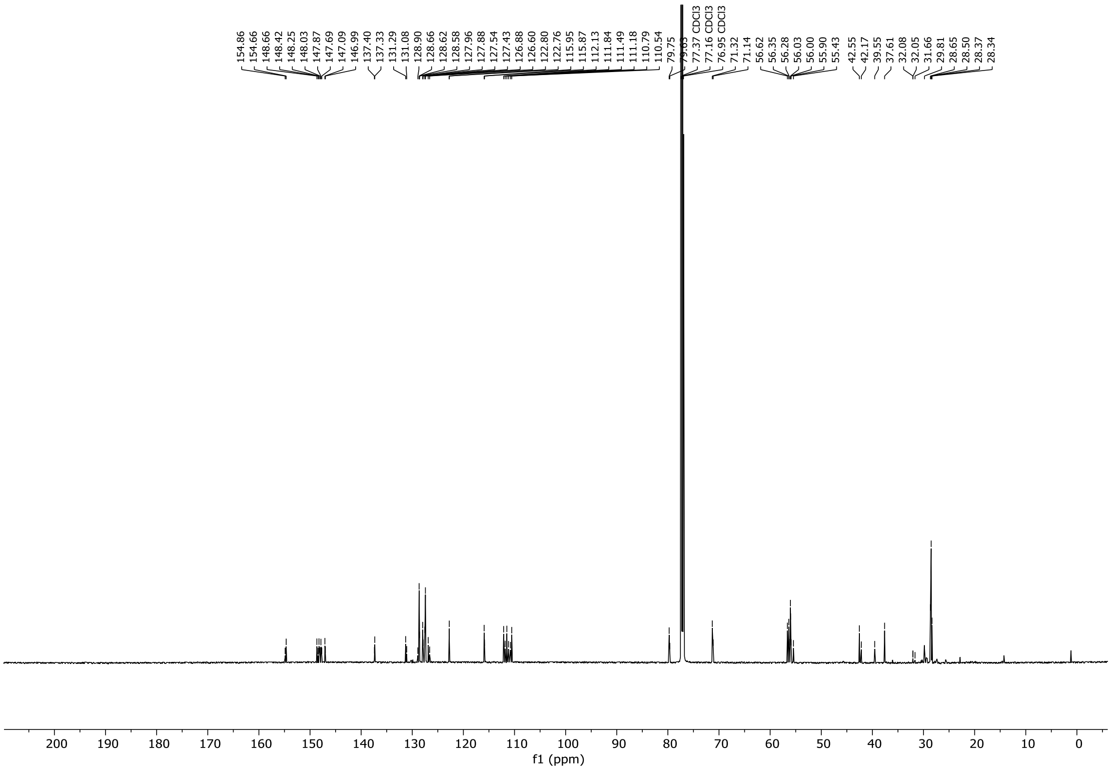

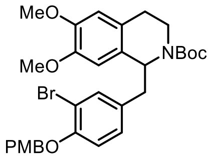

^{1}H NMR (500 MHz, $CDCl_3$, 2 rotamers) of **rac-S3**

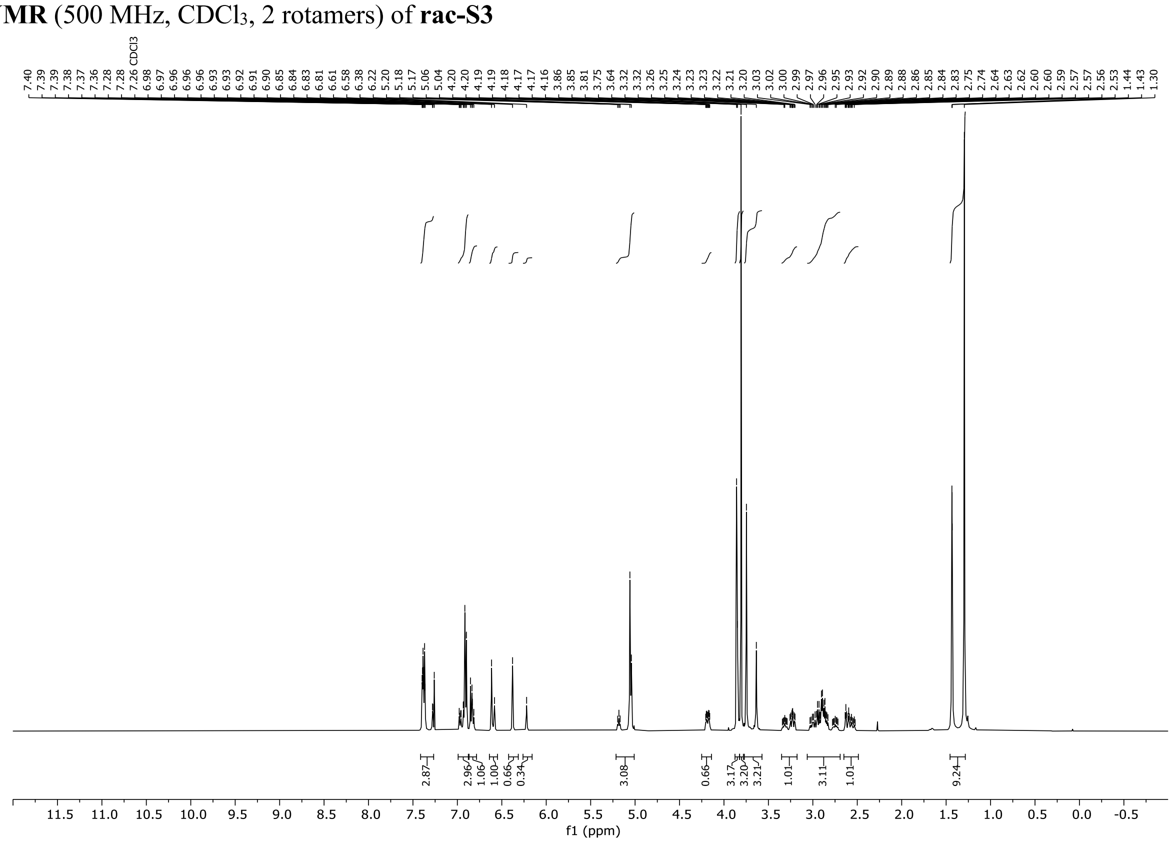

^{13}C NMR (126 MHz, $CDCl_3$, rotamers) of **rac-S3**

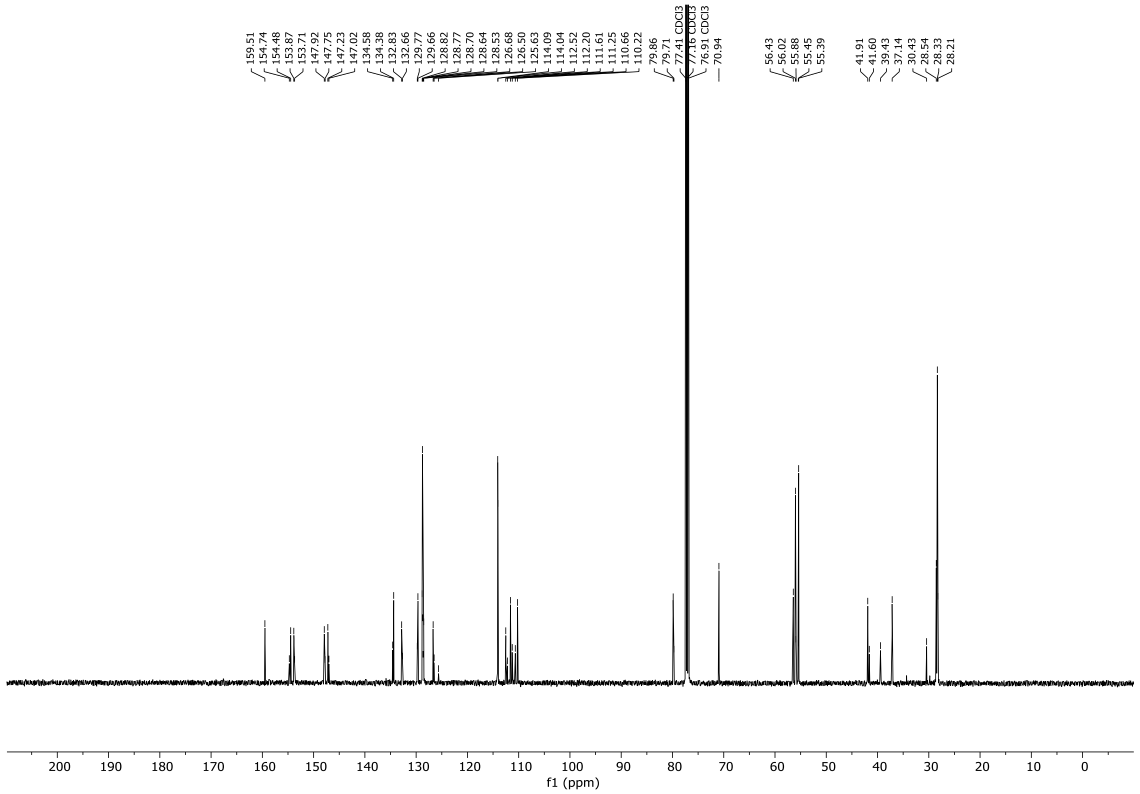

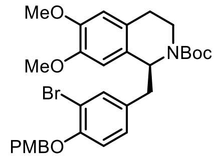

^{1}H NMR (500 MHz, $CDCl_3$, 2 rotamers) of ***(S)*-S3**

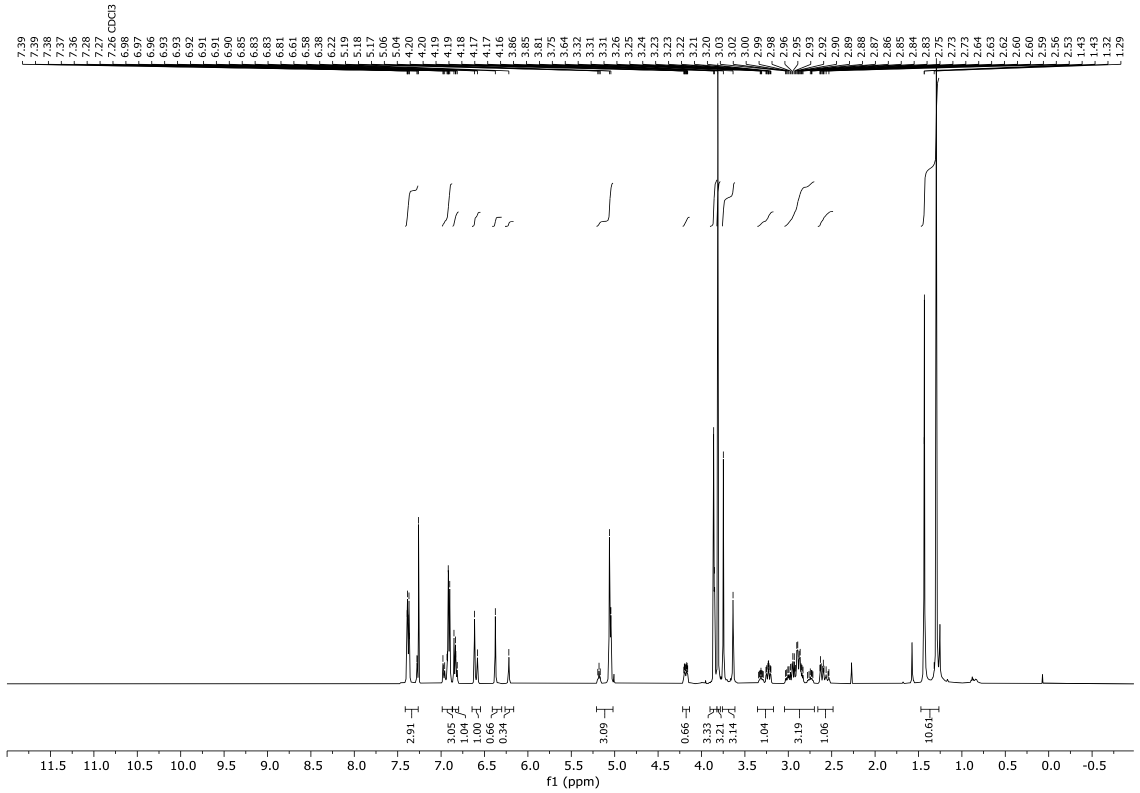

^{13}C NMR (126 MHz, $CDCl_3$, rotamers) of ***(S)*-S3**

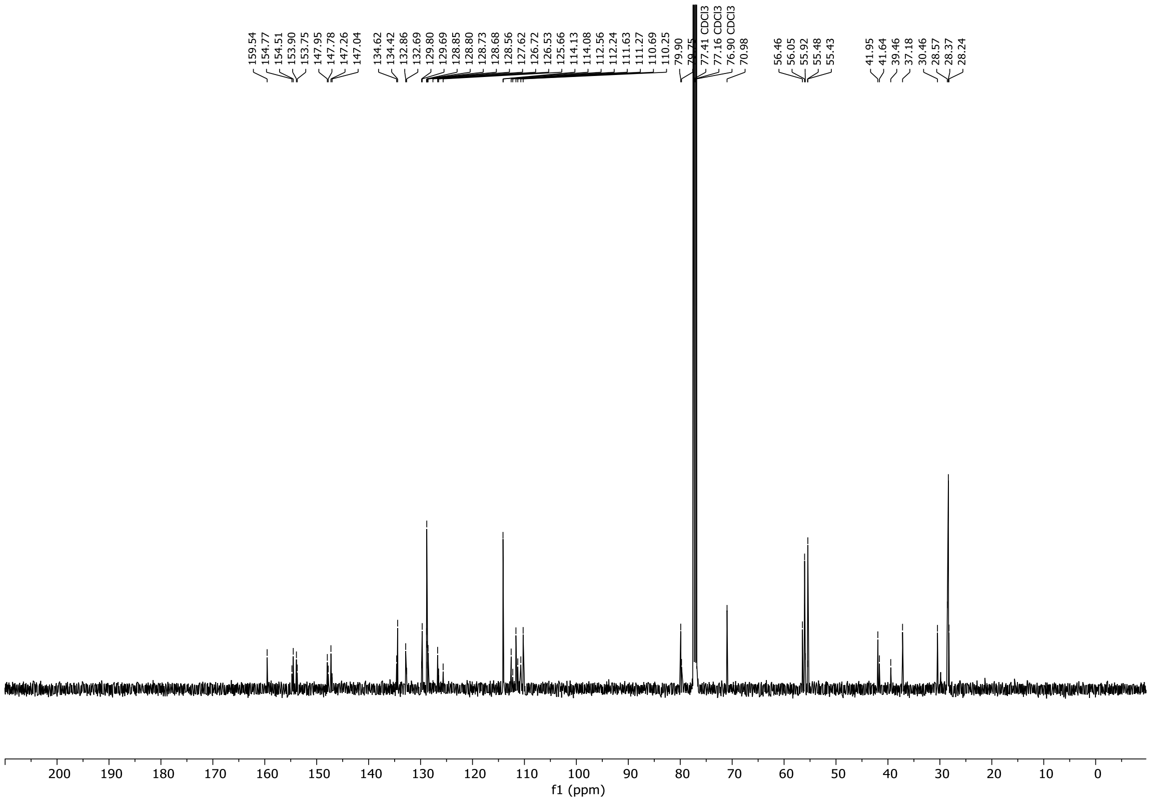

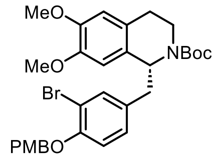

^{1}H NMR (500 MHz, $CDCl_3$, 2 rotamers) of **(*R*)-S3**

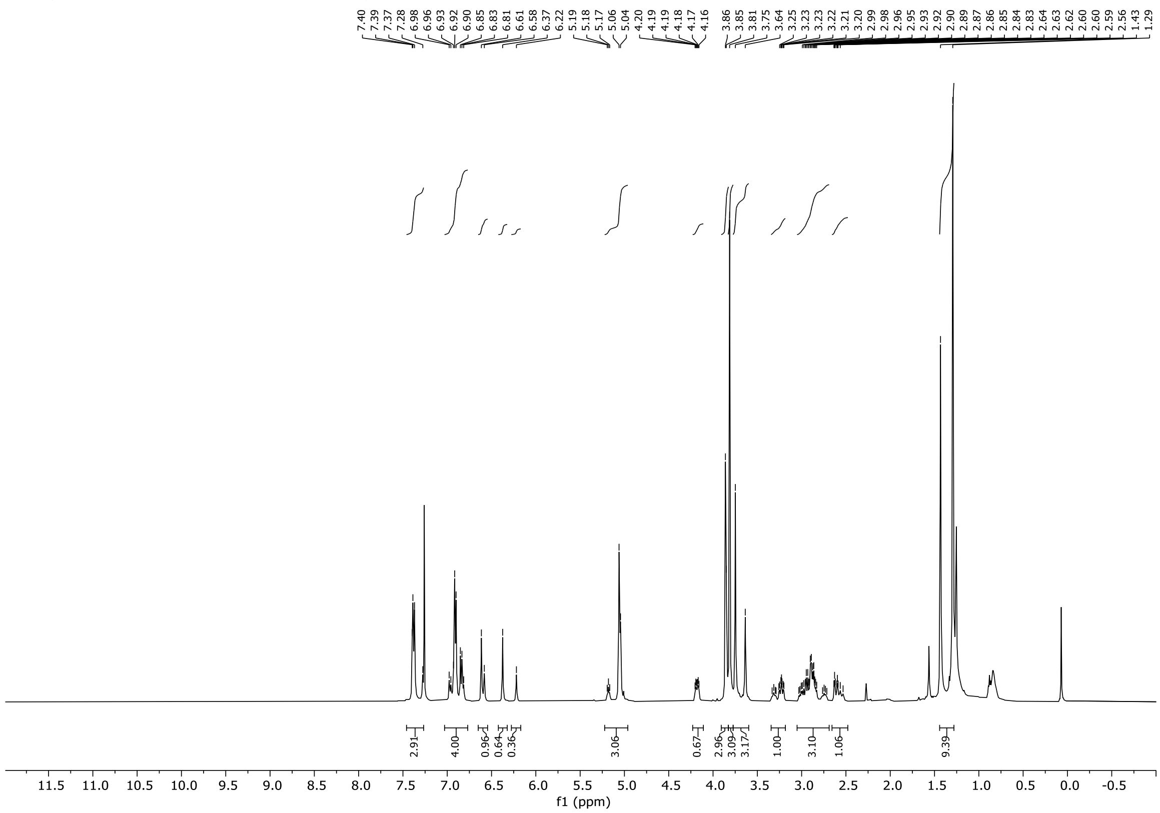

^{13}C NMR (126 MHz, $CDCl_3$, rotamers) of **(*R*)-S3**

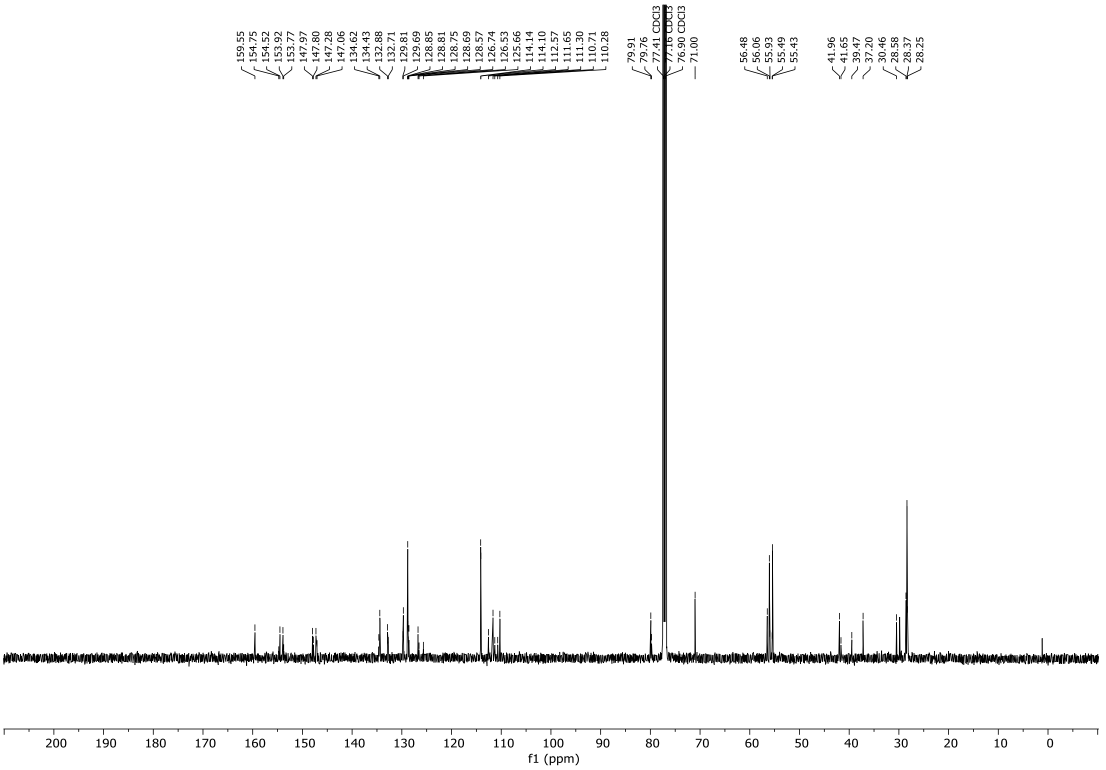

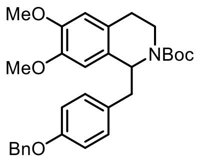

^{1}H NMR (500 MHz, $CDCl_3$, 2 rotamers) of **rac-S4**

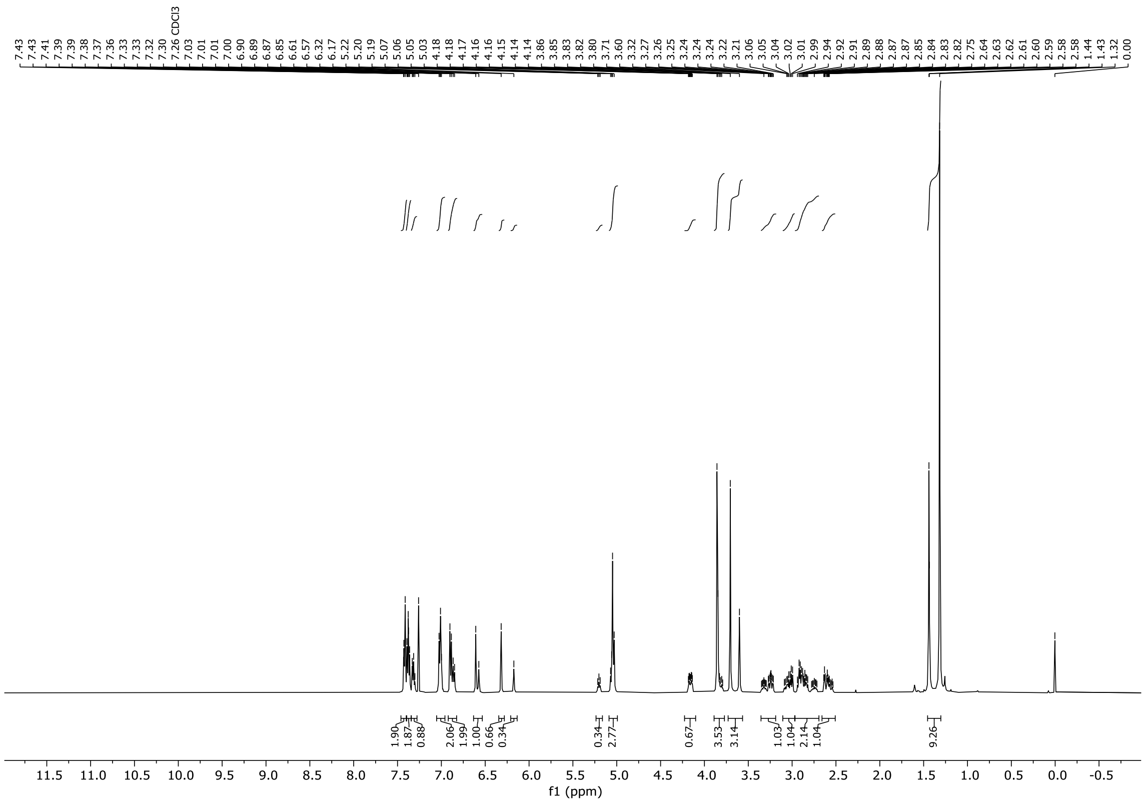

^{13}C NMR (126 MHz, $CDCl_3$, rotamers) of **rac-S4**

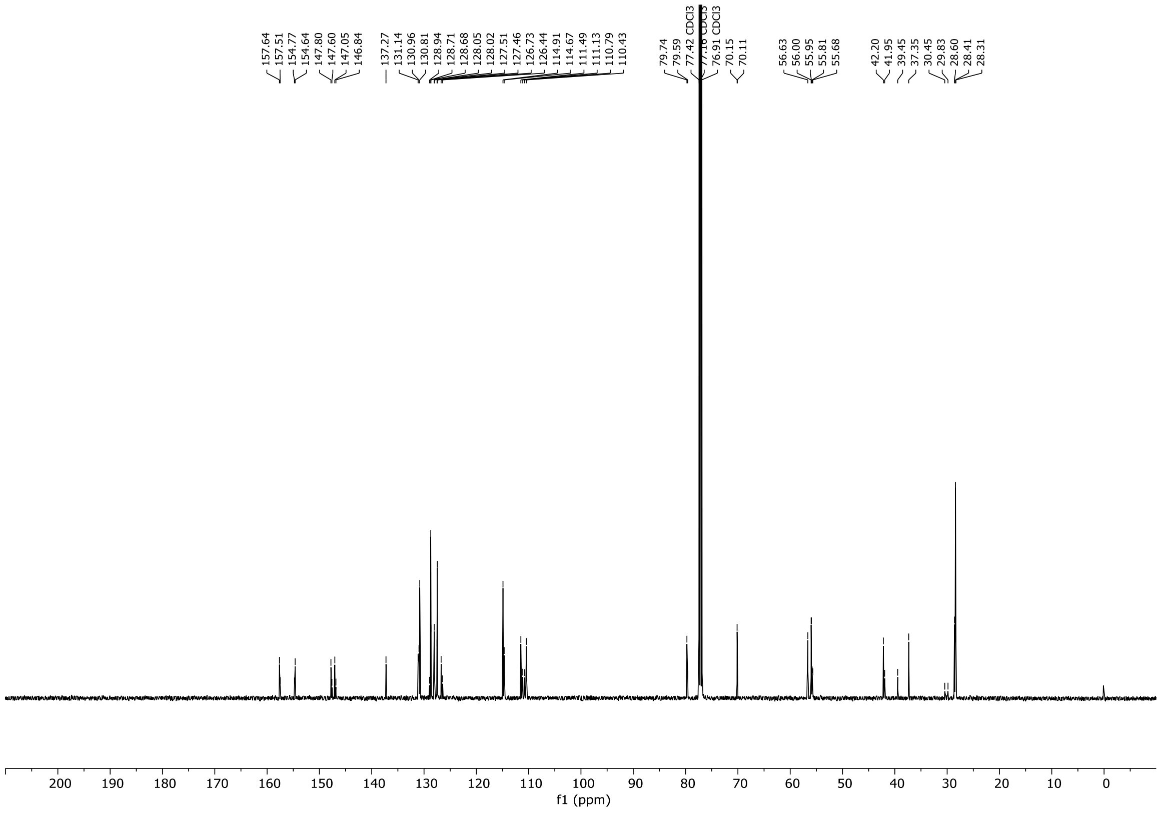

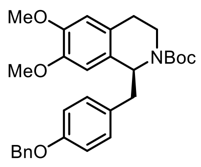

^{1}H NMR (500 MHz, $CDCl_3$, 2 rotamers) of **(*S*)-S4**

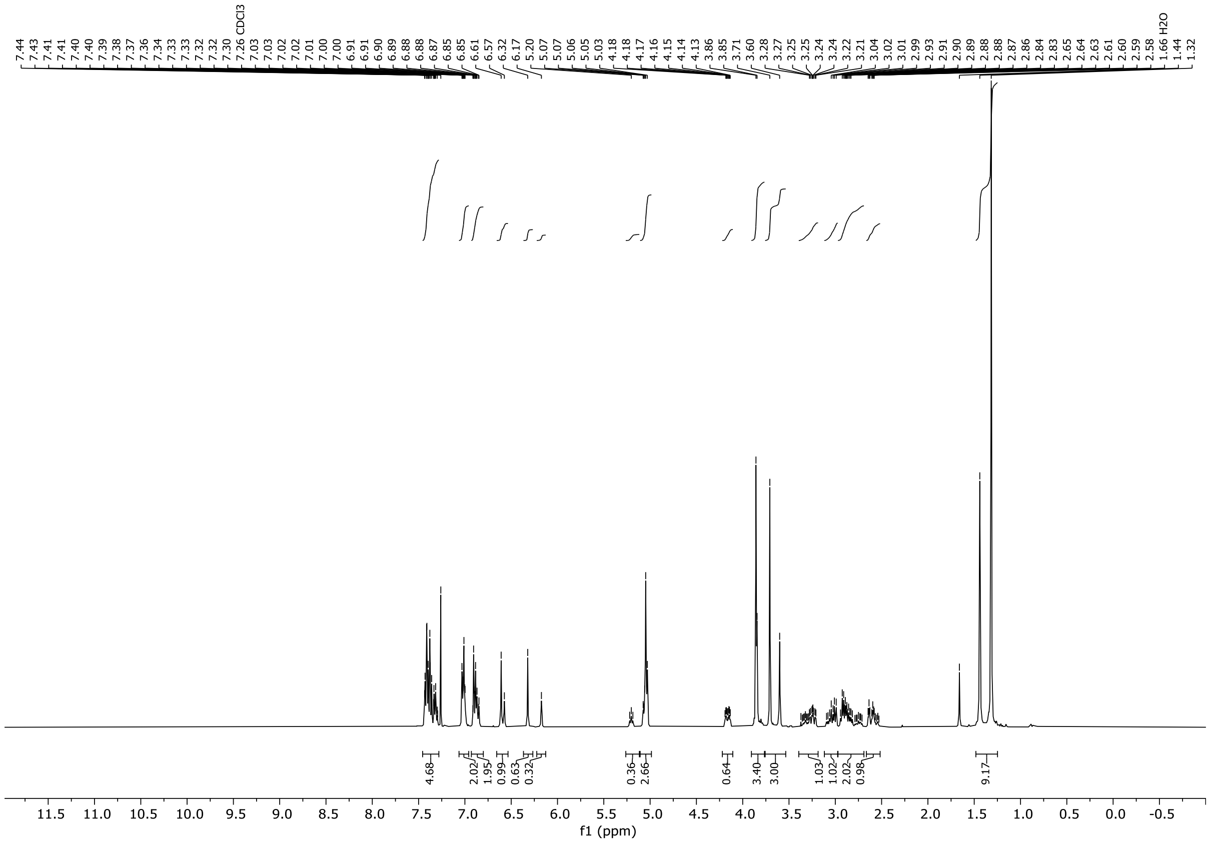

^{13}C NMR (126 MHz, $CDCl_3$, rotamers) of **(*S*)-S4**

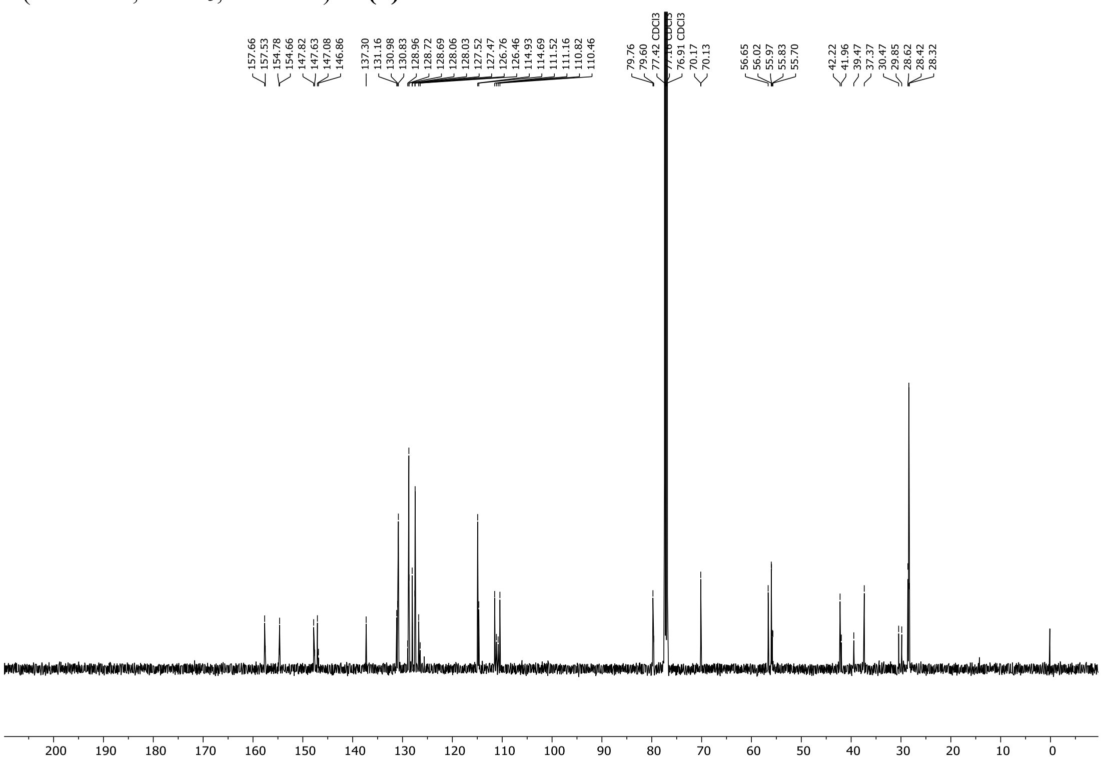

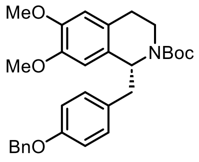

¹H NMR (500 MHz, $CDCl_3$, 2 rotamers) of ***(R)*-S4**

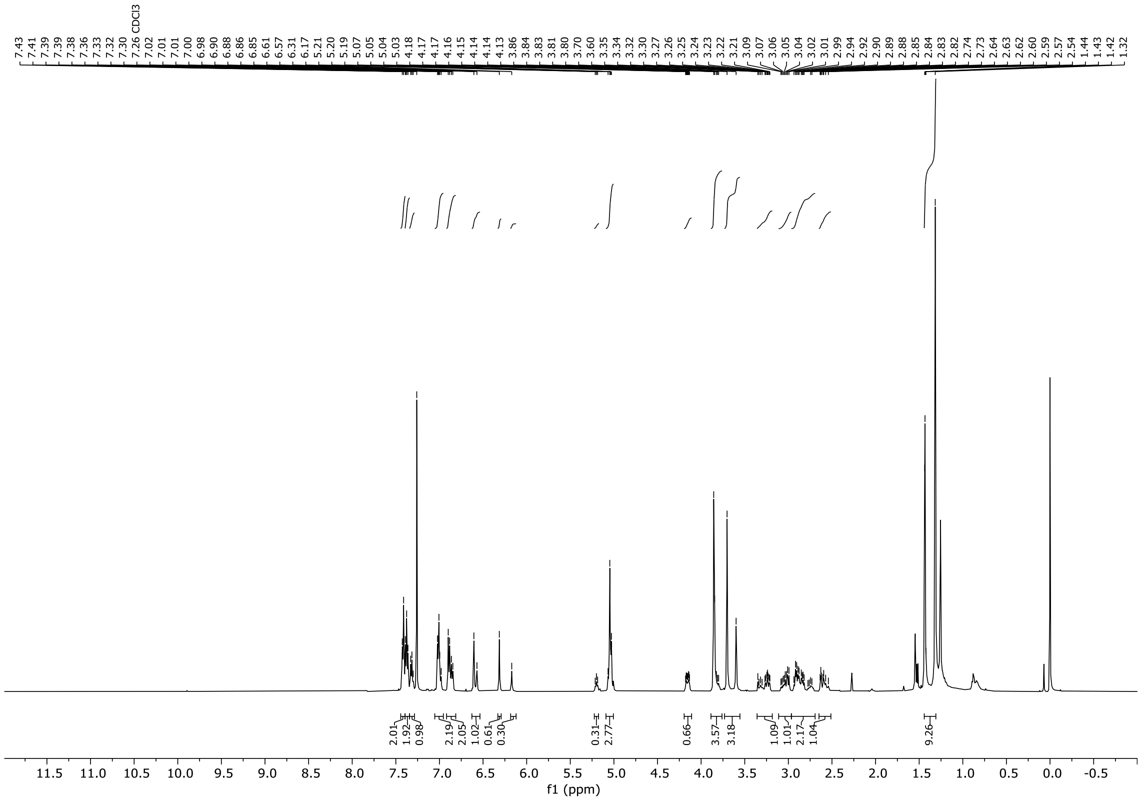

¹³C NMR (126 MHz, $CDCl_3$, rotamers) of ***(R)*-S4**

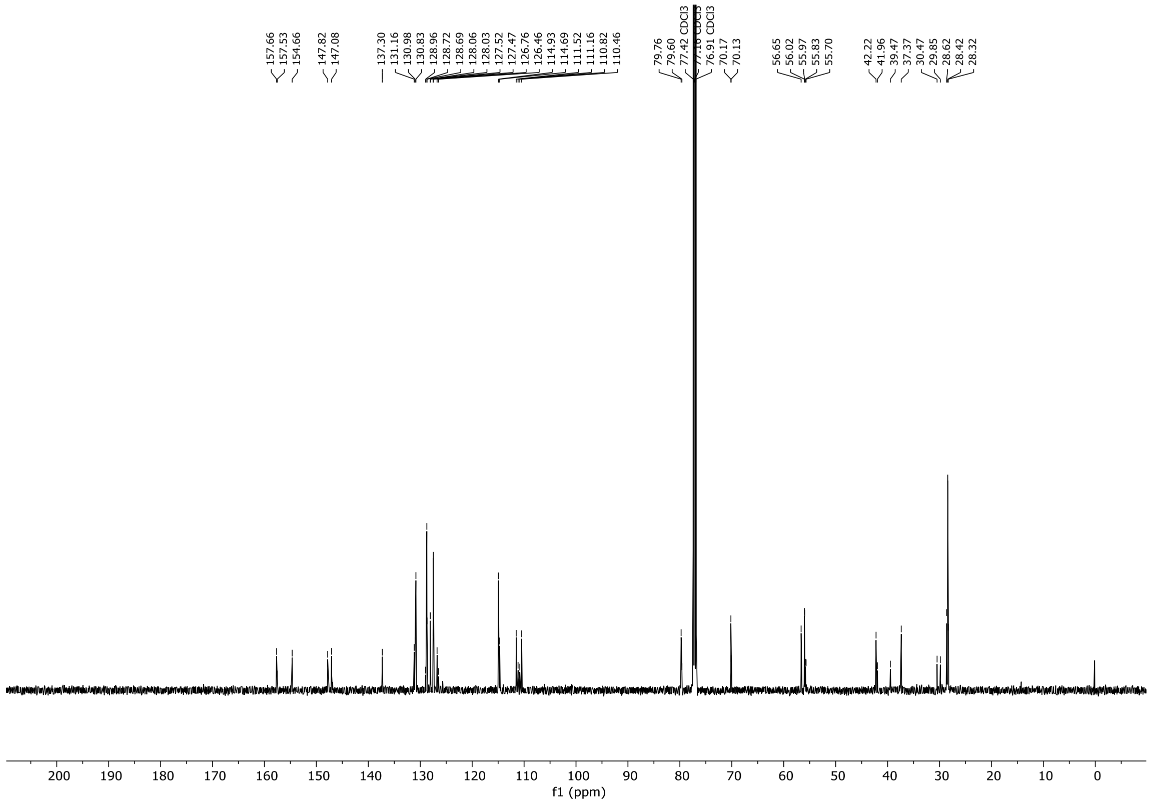

7. References

(1) Daina, A.; Michielin, O.; Zoete, V. SwissADME: a free web tool to evaluate pharmacokinetics, drug-likeness and medicinal chemistry friendliness of small molecules. *Sci. Rep.* **2017**, *7*, 42717

(2) Delaney, J. S. ESOL: Estimating Aqueous Solubility Directly from Molecular Structure. *J. CHem. Inf. Comput. Sci.* **2004**, *44*, 1000-1005

(3) Lipinski, C. A.; Lombardo, F.; Dominy, B. W.; Feeney, P. J. Experimental and computational approaches to estimate solubility and permeability in drug discovery and development settings. *Adv. Drug Deliv. Rev.* **2001**, *46*, 3-26

(4) Ghose, A. K.; Viswanadhan, V. N.; Wendoloski, J. J. A Knowledge-Based Approach in Designing Combinatorial or Medicinal Chemistry Libraries for Drug Discovery. 1. A Qualitative and Quantitative Characterization of Known Drug Databases. *J. Comb. Chem.* **1999**, *1*, 55-69

(5) Veber, D. F.; Johnson, S. R.; Cheng, H. Y.; Smith, B. R.; Ward, K. W.; Kopple, K. D. Molecular Properties That Influence the Oral Bioavailability of Drug Candidates. *J. Med. Chem.* **2002**, *45*, 2615-2623

(6) Egan, W. J.; Merz, K. M. J.; Baldwin, J. J. Prediction of Drug Absorption Using Multivariate Statistics. *J. Med. Chem.* **2000**, *2000*, 3867-3877

(7) Muegge, I.; Heald, S.; Brittelli, D. Simple Selection Criteria for Drug-like Chemical Matter. *J. Med. Chem.* **2001**, *44*, 1841-1846

(8) Baell, J. B.; Holloway, G. A. New substructure filters for removal of pan assay interference compounds (PAINS) from screening libraries and for their exclusion in bioassays. *J. Med. Chem.* **2010**, *53* (7), 2719-2740

(9) Brenk, R.; Schipani, A.; James, D.; Krasowski, A.; Gilbert, I. H.; Frearson, J.; Wyatt, P. G. Lessons learnt from assembling screening libraries for drug discovery for neglected diseases. *ChemMedChem* **2008**, *3* (3), 435-444

(10) Teague, S. J.; Davis, A. M.; Leeson, P. D.; Oprea, T. The Design of Leadlike Combinatorial Libraries. *Angew. Chem. Int. Ed.* **1999**, *38* (24), 3743-3748

(11) Beneke, T.; Madden, R.; Makin, L.; Valli, J.; Sunter, J.; Gluenz, E. A CRISPR Cas9 high-throughput genome editing toolkit for kinetoplastids. *R. Soc. Open Sci.* **2017**, *4* (5), 170095

(12) Stauber, L. A.; Franchino, E. M.; Grun, J. An Eight-day Method for Screening Compounds againstLeishmania donovaniin the Golden Hamster*. *J. Protozoology* **1958**, *5* (4), 269-273

(13) Tempone, A. G.; de Souza, C. M. M.; Prado, O. F.; Motoie, G.; Hiramoto, M. R.; Antoniazzi, M. M.; Baptista Haddad, F. C.; Carlos, J. Amphibian Secretions for Drug Discovery Studies: A Search for New Antiparasitic and Antifungal Compounds. *Lett. Drug Des. Discov.* **2007**, *4* (7), 67-73

(14) K. P. Chang; C. A. Nancy; Pearson, R. D. Intracellular Parasitism of Macrophages in Leishmaniasis: In Vitro Systems and Their Applications. *Methods Enzymol.* **1986**, *132*, 603-626

(15) Tada, H.; Shiho, O.; Kuroshima, K.-I.; Koyama, M.; Tsukamoto, K. An improved colorimetric assay for interleukin 2. *J. Immunol. Methods* **1986**, *93*, 157-165

(16) Halliday, C.; Billington, K.; Wang, Z.; Madden, R.; Dean, S.; Sunter, J. D.; Wheeler, R. J. Cellular landmarks of Trypanosoma brucei and Leishmania mexicana. *Mol. Biochem. Parasitol.* **2019**, *230*, 24-36

(17) Dean, S.; Sunter, J.; Wheeler, R. J.; Hodkinson, I.; Gluenz, E.; Gull, K. A toolkit enabling efficient, scalable and reproducible gene tagging in trypanosomatids. *Open Biol.* **2015**, *5* (1), 140197

(18) Mader, P.; Bartholomaus, R.; Nicolussi, S.; Baumann, A.; Weis, M.; Chicca, A.; Rau, M.; Simao, A. C.; Gertsch, J.; Altmann, K. H. Synthesis and Biological Evaluation of Endocannabinoid Uptake Inhibitors Derived from WOBE437. *ChemMedChem.* **2021**, *16* (1), 145-154

(19) Mohammah Movassaghi, M. D. H. A Versatile Cyclodehydration Reaction for the Synthesis of Isoquinoline and Beta-Carboline Derivatives. *Org. Lett.* **2008**, *10*, 3845-3488

(20) Uematsu, N.; Fujii, A.; Hashiguchi, S.; Ikariya, T.; Noyori, R. Asymmetric Transfer Hydrogenation of Imines. *J. Am. Chem. Soc.* **1996**, *118* (20), 4916-4917

(21) Blank, N.; Opatz, T. Enantioselective synthesis of tetrahydroprotoberberines and bisbenzylisoquinoline alkaloids from a deprotonated α-aminonitrile. *J. Org. Chem.* **2011**, *76* (23), 9777-9784

(22) Weicheng, Y.; Mingyang, Z.; Qingping, J.; Yu, C.; Zhenzhen, G.; Yanqun, D.; Yan, J.; Haoyu, W. Novel total synthesis method of racemic tetrandrine. CN113045578A, 2021.

(23) Horling, A.; Muller, C.; Barthel, R.; Bracher, F.; Imming, P. A new class of selective and potent 7-dehydrocholesterol reductase inhibitors. *J. Med. Chem.* **2012**, *55* (17), 7614-7622

(24) Fujitani, K.; Aoyagi, Y.; Masaki, Y. Studies on the Alkaloids of Menispermaceous Plants CCXXIV. Syntheses of Dauricine Type Bases. Synthesis of 2-Hydroxy-5,4'-bis(2-methyl-6,7-dimethoxy-1,2,3,4-tetrahydroisoquinolin-1-ylmethyl)-diphenyl ether. *J. Pharm. Soc. Japan* **1966**, *86*, 654-659

(25) Xu, Z. C.; Wang, X. B.; Yu, W. Y.; Xie, S. S.; Li, S. Y.; Kong, L. Y. Design, synthesis and biological evaluation of benzylisoquinoline derivatives as multifunctional agents against Alzheimer's disease. *Bioorg. Med. Chem. Lett.* **2014**, *24* (10), 2368-2373

(26) Blank, N.; Straub, B. F.; Opatz, T. 1,3-Benzyl Migration in Iminium Ions: Evidence for a Fast Free-Radical Chain Reaction. *Eur. J. Org. Chem.* **2011**, *2011* (36), 7355-7365

(27) Kikkawa, I. dl-Laurotetanine. *J. Pharm. Soc. Japan* **1959**, *79* (4), 425-428

(28) Orejarena Pacheco, J. C.; Lahm, G.; Opatz, T. Synthesis of alkaloids by Stevens rearrangement of nitrile-stabilized ammonium ylides: (+/-)-laudanosine, (+/-)-laudanidine, (+/-)-armepavine, (+/-)-7-methoxycryptopleurine, and (+/-)-xylopinine. *J. Org. Chem.* **2013**, *78* (10), 4985-4992

(29) Frydman, B.; Bendisch, R.; Deulofeu, V. A synthesis of laudanidine and (±)-pseudo-codamine: resolution into the optial isomers. *Tetrahedron* **1958**, *4*, 342-350

(30) Ruiz-Olalla, A.; Wurdemann, M. A.; Wanner, M. J.; Ingemann, S.; van Maarseveen, J. H.; Hiemstra, H. Organocatalytic enantioselective Pictet-Spengler approach to biologically relevant 1-benzyl-1,2,3,4-tetrahydroisoquinoline alkaloids. *J. Org. Chem.* **2015**, *80* (10), 5125-5132

(31) Kametani, T.; Sakurai, K.; Iida, H. Total Syntheses of Gerbamunine and Its Diastereomer (Studies on the Syntheses of Heterocyclic compounds CCLIV). *J. Pharm. Soc. Japan* **1970**, *90*, 1182-1185

(32) Ahmad, R.; Cava, M. P. Grisabine and Grisabutine, New Bisbenzyiisoquinoline Alkaloids from Abuta grisebachii. *J. Org. Chem.* **1977**, *42*, 2271-2273

(33) Falco, M. R.; de Vries, J. X.; Maccio, Z.; Bick, I. R. C. Alkaloids ofBerberis laurina Billb. II. Two new phenolic biscoclaurine alkaloids. *Experientia* **1969**, *25* (12), 1236-1237

(34) Ma, H.; Liang, H.; Cai, S.; O'Keefe, B. R.; Mooberry, S. L.; Cichewicz, R. H. An Integrated Strategy for the Detection, Dereplication, and Identification of DNA-Binding Biomolecules from Complex Natural Product Mixtures. *J. Nat. Prod.* **2021**, *84* (3), 750-761

(35) Manske, R. H. F.; Tomita, M.; Fujitani, K.; Okamoto, Y. Studies on the Alkaloids of Menispermaceous Plants. CCXIX. Dauricine from Menispermum canadense L. *Chem. Pharm. Bull.* **1965**, *13* (12), 1476-1477
